# Effects of Belt Accelerations During Push-Off on Propulsion Mechanics in Individuals Post-Stroke

**DOI:** 10.1109/TNSRE.2026.3675477

**Published:** 2026

**Authors:** Hannah N. Cohen, Tamara Wright, GilHwan Kim, Henry Wright, Darcy S. Reisman, Fabrizio Sergi

**Affiliations:** Department of Biomedical Engineering, University of Delaware, Newark, DE 19713 USA; Department of Physical Therapy, University of Delaware, Newark, DE 19716 USA; Department of Mechanical Engineering, University of Delaware, Newark, DE 19713 USA; Department of Physical Therapy, University of Delaware, Newark, DE 19716 USA; Department of Physical Therapy, University of Delaware, Newark, DE 19716 USA; Department of Biomedical Engineering, University of Delaware, Newark, DE 19713 USA, and also with the Department of Mechanical Engineering, University of Delaware, Newark, DE 19713 USA

**Keywords:** Legged locomotion, muscles, propulsion, rehabilitation, stroke (medical condition)

## Abstract

Stroke often causes hemiparesis, affecting balance and walking ability. Propulsion, a major subtask of walking, has two components: trailing limb posture and propulsive force generated by plantarflexor muscles. Our group previously developed a method to challenge propulsion by accelerating the belt supporting the trailing limb during push off. In this study, we test the efficacy of a similar paradigm in 34 post-stroke individuals, and compare the effects of posterior belt accelerations applied to both legs (symmetric condition), and only to the paretic leg (asymmetric condition). We hypothesized that the two conditions would elicit changes in propulsion mechanics during and after exposure. Results indicate that belt accelerations induced measurable effects in paretic propulsion mechanics during exposure, and some of these effects persisted over a 1-3 minute post-exposure session conducted at self-selected speed. Specifically, by the end of the exposure session, participants increased their paretic TLA by 7.2 ± 0.9%, and their plantarflexor muscle activation by 6.6±2.2% in the soleus and 7.8 ± 2.3% in the lateral gastrocnemius, compared to their baseline. Changes in propulsion mechanics led to a small but statistically significant (0.024 m/s or 3.4 ± 1.4%) post-exposure increase in selfselected walking speed. Effects were primarily induced on metrics of propulsion mechanics of the leg directly exposed to belt acceleration; therefore, differential effects as a function of acceleration condition were only observed for the non-paretic leg. A responder analysis indicated that individuals with greater impairment exhibited larger relative changes in plantarflexor muscle activation after exposure.

## I. Introduction

EVERY year, approximately 795,000 individuals in the United States suffer a stroke [1]. Following a stroke, damage to the brain and central nervous system often leads to a loss of voluntary movement on one side of the body, known as hemiparesis. Around 75% of stroke survivors face challenges with walking, most commonly characterized by hemiparetic gait [2]. Hemiparetic gait typically results in slower walking speeds, shorter stride lengths, and reduced cadence [2], [3], [4]. This type of gait is less efficient than normal walking, resulting in higher metabolic costs [5], [6]. For many individuals recovering from a stroke, improving their walking ability is a key goal, with a particular emphasis on achieving walking independence over aspects such as distance, speed, and gait appearance [7], [8]. To support individuals towards these goals, current rehabilitation methods often prioritize quick recovery over effective gait restoration. These approaches encourage patients to compensate for their impairments to regain independence rapidly, rather than retraining for a more energy-efficient walking style [6]. Innovative rehabilitation strategies are needed to enhance quality of life and promote greater independence through improved walking.

Propulsion is one of three major subtasks of walking including body support and swing initiation [9], [10]. Propulsion is closely associated with walking speed, as propulsive force increases with gait speed [10], [11], [12], [13]. Propulsion consists of two components: the kinematic component (determined by the posture of the trailing limb at push-off, usually quantified as the trailing limb angle - TLA) and the kinetic component (resulting from the force generated by plantarflexor muscles in late stance). In individuals post-stroke, propulsion is asymmetric and weaker in the paretic limb compared to normal walking [2], [5]. Hemiparetic severity is strongly correlated with paretic propulsion; as hemiparetic severity increases, the percentage of total propulsion generated by the paretic leg decreases [11]. Enhancing either TLA or plantarflexor muscle force can lead to improvements in propulsion and increased walking speed [12].

Previous propulsion training methods can be categorized along two dimensions. The first dimension reflects the primary learning mechanism targeted during practice [14]. At one end of this spectrum are explicit paradigms, in which users are instructed to modify movement based on external feedback. At the other end are implicit paradigms, driven by sensorimotor prediction errors induced through perturbations or distortions of task dynamics or goals, with participants typically instructed to walk as naturally as possible. Some training methods also employ a mechanical manipulation of propulsion mechanics, defining the second dimension: whether propulsion is facilitated or challenged during practice.

Biofeedback is a common method targeting explicit learning, and has been used to train anterior ground reaction force (AGRF), TLA, and muscle activation via audio and visual methods [15], [16], [17], [18], [19], [20]. In contrast, implicit learning paradigms are based on manipulations of propulsion mechanics, induced by tethers, hip or ankle powered devices [21], [22], [23], [24], [25], requiring participants to modify their motor coordination to maintain steady-state walking. Although explicit learning may occur more rapidly (often resulting in larger immediate post-training effects), it requires a significantly greater cognitive load and relies on voluntary recall for later use. In contrast, learning retained without conscious effort reflects use-dependent or implicit processes [14], which may be more suitable for post-stroke rehabilitation.

Along the second dimension, facilitatory interventions directly assist propulsion. These include functional electrical stimulation, which electrically stimulates the plantarflexor muscles to enhance push-off [26], [27], [28], [29], [30], [31] and exosuits that generate assistive ankle plantar/dorsiflexor forces throughout the gait cycle [25]. Anterior treadmill belt accelerations have also been used to extend ground contact time, giving participants more time to generate propulsive force, or to provide an external assistive push-off force [20]. Conversely, challenge-based paradigms, such as those based on resistive forces applied via tethers or exoskeletons, aim to elicit improved propulsion by drawing on an underutilized propulsion reserve [21]. Because this capacity exists beyond laboratory and clinical settings, challenge-based approaches may offer greater potential for translation to real-world walking.

Our lab previously developed AccelBelt, a novel implicit training method to challenge propulsive force generation based on posterior accelerations of the trailing limb during push-off. In a previous study, our group showed that AccelBelt affects both the kinematic and kinetic components of propulsion during and after ten minutes of exposure in healthy young adults [32]. While our previous work demonstrated the effectiveness of AccelBelt in healthy individuals, it is unknown whether this approach can be used to modulate propulsion in individuals post-stroke, an important step toward developing rehabilitation strategies that specifically target deficits in paretic propulsion.

The primary purpose of this study was to establish the feasibility of using belt accelerations to modulate paretic propulsion in individuals post-stroke. We evaluated two methods: delivering accelerations to both legs (symmetric) or only to the paretic leg (asymmetric). To test for changes in propulsion, we used force plates to quantify peak AGRF and propulsive impulse of both legs during and after exposure to belt accelerations. To quantify effects in each of the two components of propulsion, we used EMG data from four ankle plantar- and dorsi-flexor muscles and a motion-capture set-up to measure TLA and stride length. We tested the following hypotheses: (1) propulsion will change relative to baseline during and after exposure as a result of exposure to belt accelerations, (2) there will be differential effects in propulsion between symmetric and asymmetric conditions, and (3) baseline impairment will have an effect on the exposure and post-exposure effects of belt accelerations on propulsion mechanics.

## II. Methods

41 individuals post-stoke were recruited to participate in this study. Of these, one was excluded due to treadmill malfunction, one for excessive cross-over between treadmill belts, two for being unable to sustain walking with belt accelerations for 5 minutes, and three for exceeding safe heart rate levels (see protocols below). This resulted in a total of 34 participants who completed the study (18 female, age mean ± std: 65.2 ± 9.8 years, 87.6 ± 59.3 months post-stroke). Inclusion criteria for this study were: i) age between 18-85 years old; ii) imaging-confirmed diagnosis of a single, unilateral chronic stroke at least six months prior to participating in the experiment; iii) able to walk at a self-selected speed for at least 15 minutes without assistance; iv) weight less than 250 lbs (due to treadmill constraints); v) free of neurological conditions (other than stroke) that affect walking function; vi) free of musculoskeletal pain or conditions that limit walking function; vii) free of any severe respiratory problems; viii) resting heart rate between 50-110 bpm, and ix) resting blood pressure between 90/60 to 160/90 mmHg. Potential participants were excluded if they reported a coronary artery bypass graft or myocardial infarction within 3 months prior to participating in the experiment, unexplained dizziness, a visual field cut, or spatial neglect. The study was approved by the Institutional Review Board of the University of Delaware (IRBNet ID: 1835998, IRBNet ID: 2068278). This study was a component of the clinical study, Model-informed Patient-specific Rehabilitation Using Robotics and Neuromuscular Modeling, which is registered on Clinical-Trials.gov (NCT06008743). Each participant provided written informed consent and received compensation for participation.

### A. Experimental Set-Up

Participants walked on an instrumented dual-belt treadmill (Bertec Corp., Columbus OH, USA) while wearing eight bipolar EMG sensors (soleus - SOL, lateral and medial gastrocnemius - LGAS and MGAS, and tibialis anterior - TA, on both legs) and four reflective markers (one on each greater trochanter and lateral malleolus). Participants were also fitted with a chest strap heart rate monitor (Polar Electro, Kempele, Finland) to allow us to monitor their heart rate during the experiment. A ten-camera Vicon T40-S passive motion capture system (Oxford Metrics, Oxford, UK) was used to capture marker data at 500 Hz and a Delsys Trigno system (Delsys Inc., Natick MA, USA) was used to record EMG data at 1925 Hz. Force/torque data from the force plates were recorded in Simulink (MathWorks Inc., Natick MA, USA) at 500 Hz. A 40-inch screen placed at eye level about 2 m in front of the treadmill provided a visual target for the participants to redirect their focus away from the treadmill belts. The screen displayed nature images that changed every 90 s. Participants also wore a safety harness attached to a rolling ceiling-mounted platform to prevent falls.

### B. Experimental Protocol

The overall experiment consisted of two sessions. At the beginning of the first session (before placing the EMG sensors and reflective markers), we administered a clinical examination consisting of the lower extremity portion of the Fugl-Meyer test (FMLE) [33] and the 10-meter walk test [34]. Over the two sessions, the participants completed the procedures described below twice, once in the symmetric condition (belt accelerations applied to both legs), and once in the asymmetric condition (belt accelerations applied only to the paretic leg), in a randomized order.

We first found the participant’s self-selected speed on the treadmill by starting at a slow speed (0.3 m/s) and increasing the velocity in increments of 0.02 m/s until the participant and/or the physical therapist verbally indicated that the treadmill appeared to reach the participant’s comfortable speed. Participants were allowed a “light touch” on the handrails of the treadmill to keep a sense of stability. We then ran a 1-2 minute practice with the adaptive treadmill controller. The adaptive treadmill is based on that described by [35]; treadmill velocity is defined based on the deviation between desired and estimated position/velocity on the treadmill. The estimate is provided by a Kalman filter, which uses antero-posterior ground reaction forces and the antero-posterior location of the center of pressure at heel strike (HS). The velocity of the belt increases if the center of mass is located anteriorly relative to the baseline, and if the estimated velocity is positive. The velocity decreases if the opposite is true. If the participant had a hard time maintaining a constant speed, we adjusted the gains on the controller to make the controller less sensitive and ran the practice again.

**Fig. 1.**
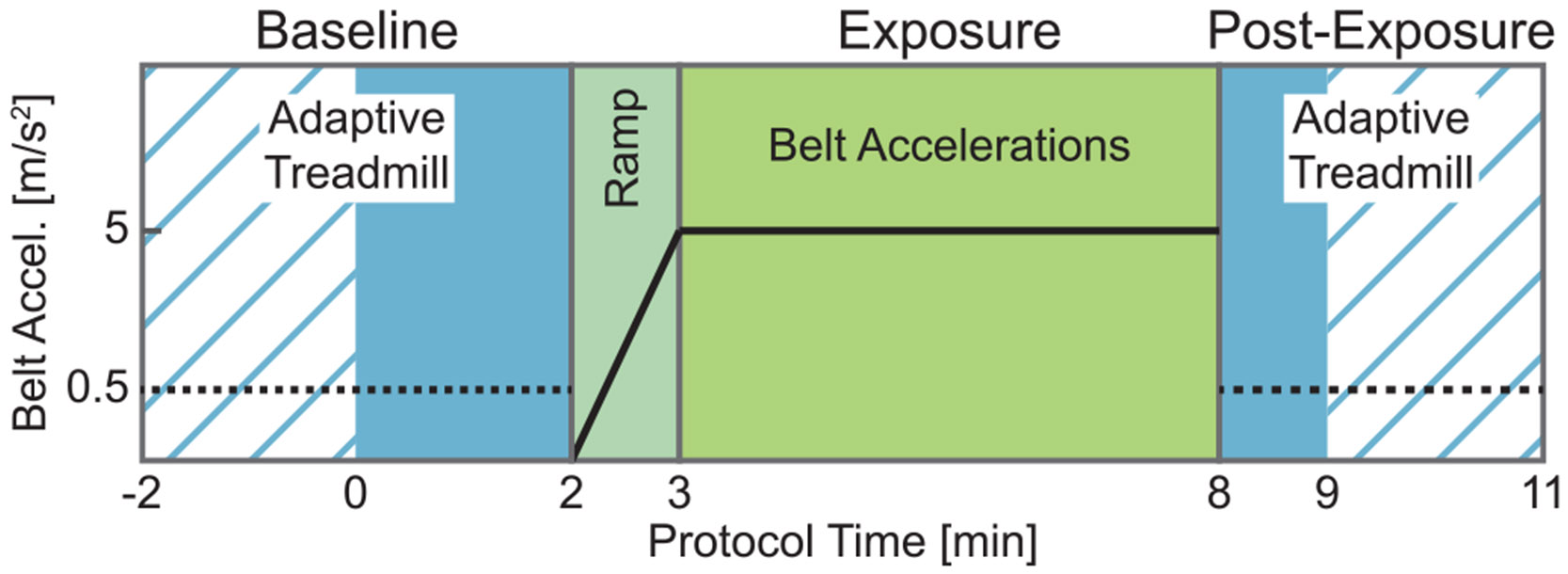
Timing diagram showing the progression of the experimental protocol. All participants walked for at least 2 minutes during Baseline (solid blue, 0-2 min). If needed to achieve steady speed, up to 2 additional minutes were included (striped blue, −2-0 min). During Exposure, accelerations are gradually increased to the target value of 5 m/s^2^, then maintained for 5 minutes. Post-Exposure, all participants walked for at least 1 minute (solid blue, 8-9 min), with some continuing up to 3 minutes total (striped blue, 9-11 min). The dotted black line during Baseline and Post-Exposure indicates the maximum rate of change in velocity allowed by the adaptive treadmill controller.

The walking trial began with participants walking for two minutes at their self-selected speed on the adaptive treadmill. If they maintained their speed within 0.2 m/s during the last minute (Baseline), they proceeded to the acceleration ramp phase, where posterior belt accelerations gradually increased over one minute. Participants unable to maintain a steady speed continued walking until they met this goal or 4 minutes had passed, after which the treadmill was stopped, they rested, and the trial restarted. After the one-minute acceleration ramp, the participants walked for five minutes (Exposure) with the belt accelerations at their maximum magnitude (5 m/s^2^) with a base speed equal to the average speed of the last minute of Baseline. This was followed by 1-3 minutes of walking with the adaptive treadmill, depending on the participant’s level of exhaustion (Post-Exposure) (Fig. 1).

Throughout the experiment, the participant’s heart rate was continuously monitored by a physical therapist. An age-specific maximum heart rate was calculated as

(1) $${\text{HR}}_{\max}=220-\text{age}.$$

If the participant’s heart rate exceeded 85% of ${\text{HR}}_{\max}$, the participant was asked to take a break. If this occurred multiple times in a session, the participant was either rescheduled for a later session or withdrawn from the protocol due to cardiovascular safety concerns.

### C. Belt Acceleration Controller

In general, AccelBelt controllers aim to accelerate the belt supporting the trailing limb during late stance to modulate propulsive force, preferably during double support for safety. Due to inherent delays in treadmill acceleration (~75 ms), and the short duration of double support at normal walking speeds (~125 ms), the previous AccelBelt controller [32] sent an acceleration signal in anticipation of contralateral HS, based on predictions from the previous gait cycle. At slower walking speeds, where double support duration is naturally longer, this predictive approach resulted in longer acceleration durations, making it difficult to control the “dose” of exposure to belt accelerations. Additionally, high variability in gait event timing, as observed in post-stroke individuals, reduces the reliability of event predictions and may compromise the safety of the protocol.

To improve accuracy and safety at low speeds (ν≤0.5m∕s), we modified the logic of our previous controller. The new controller is primarily based on predicting toe off (TO) events, and accelerates the belt for a fixed duration ($T_{\text{TOT}}$) before the predicted TO event ($T_{\text{TO},\text{pred}}$) if contralateral HS has been detected ($HS_{\text{cont},\text{evt}}$) (Algorithm 1, lines 8, 14, 20, 21). The total duration $T_{\text{TOT}}$ depends on the desired acceleration duration ($T_{\text{des}}$) and the intrinsic actuation delays of the treadmill ($T_{\text{del}}$). In our set-up, $T_{\text{des}}$ = 180 ms and $T_{\text{del}}$ = 40 ms, resulting in $T_{\text{TOT}}$ = 220 ms.

At higher speeds (0.5 < ν < 1 m/s), where the duration of double support is comparable with $T_{\text{des}}$, it is important to send the acceleration command based on predicted contralateral HS ($T_{\text{HS},\text{pred}}$), and not after detection, to minimize the effect of actuation delays. As such, the new controller implements additional logic at higher velocities. Rather than waiting to detect HS, the controller sends the acceleration signal $T_{\text{ant}}$ seconds before $T_{\text{HS},\text{pred}}$ (Algorithm 1, line 9). Due to the negative association between double support duration and gait speed, we scaled parameter $T_{\text{ant}}$ with velocity to achieve consistent timing across speed (Algorithm 1, lines 1-7).

Additional logic was put into place to pause accelerations for three steps if the participant steps onto the other belt or if their gait timing is highly variable (Algorithm 1, lines 17-18). Every stride, the controller follows the logic described above. However, if contralateral HS is not detected within 70 ms of $T_{\text{HS},\text{pred}}$, the acceleration will stop, and the controller will wait three steps to reset the gait event predictions before resuming accelerations. Similarly, if the contralateral foot is in stance ($HS_{\text{cont}}$), but TO of the accelerated foot ($TO_{\text{detected}}$) does not occur within 250 ms of the start of the acceleration command ($t_{\text{accel}}$), the acceleration is stopped and the controller will pause accelerations for three steps, preventing excessively long accelerations.

### D. Data Processing and Analysis

Force plate and motion capture data were synchronized using QUARC real-time control software (Quanser, Markham, ON, Canada) in Simulink, which was used to read data in real time from an NI PCIe 6321 card and from Vicon Tracker (Oxford Metrics, Oxford, UK). A square wave signal was sent every second by the real-time model and fed into EMGworks Acquisition (Delsys Inc., Natick MA, USA) via a Trigno analog input adapter to synchronize the force plate and motion capture data with EMG data. Force plate data were low-pass filtered at 25 Hz with a 4th-order zero-shift Butterworth filter. EMG data were bandpass filtered at 20-500 Hz, rectified, and the envelope was taken via a lowpass 4th-order zero-shift Butterworth filter with a cutoff frequency of 10 Hz. Motion capture marker data gaps less than 25 samples (50 ms) were linearly interpolated.

HS events were defined in post-processing as the instants at which vertical ground reaction force exceeded 50 N and remained above 50 N for at least 200 ms. TO events were defined as the instants when vertical ground reaction force dropped below 50 N and remained below 50 N for at least 200 ms. EMG data were segmented and resampled into 0-100% of stance and swing for each leg based on HS and TO events (resulting in 200 data points per stride). For the TA, EMG data was also resampled into 0-100% of double support, 0-100% between TO of the two feet, and 0-100% of swing (resulting in 300 data points per stride).

Velocity was defined as the average over the gait cycle. To evaluate changes in push-off force, we calculated both peak AGRF and propulsive impulse (PI). Peak AGRF was calculated as the maximum value of the anterior (positive) component of ground reaction force within a gait cycle. PI was calculated as the area under the positive portion of the AGRF. We normalized both outcomes by bodyweight.

**Fig. 2.**
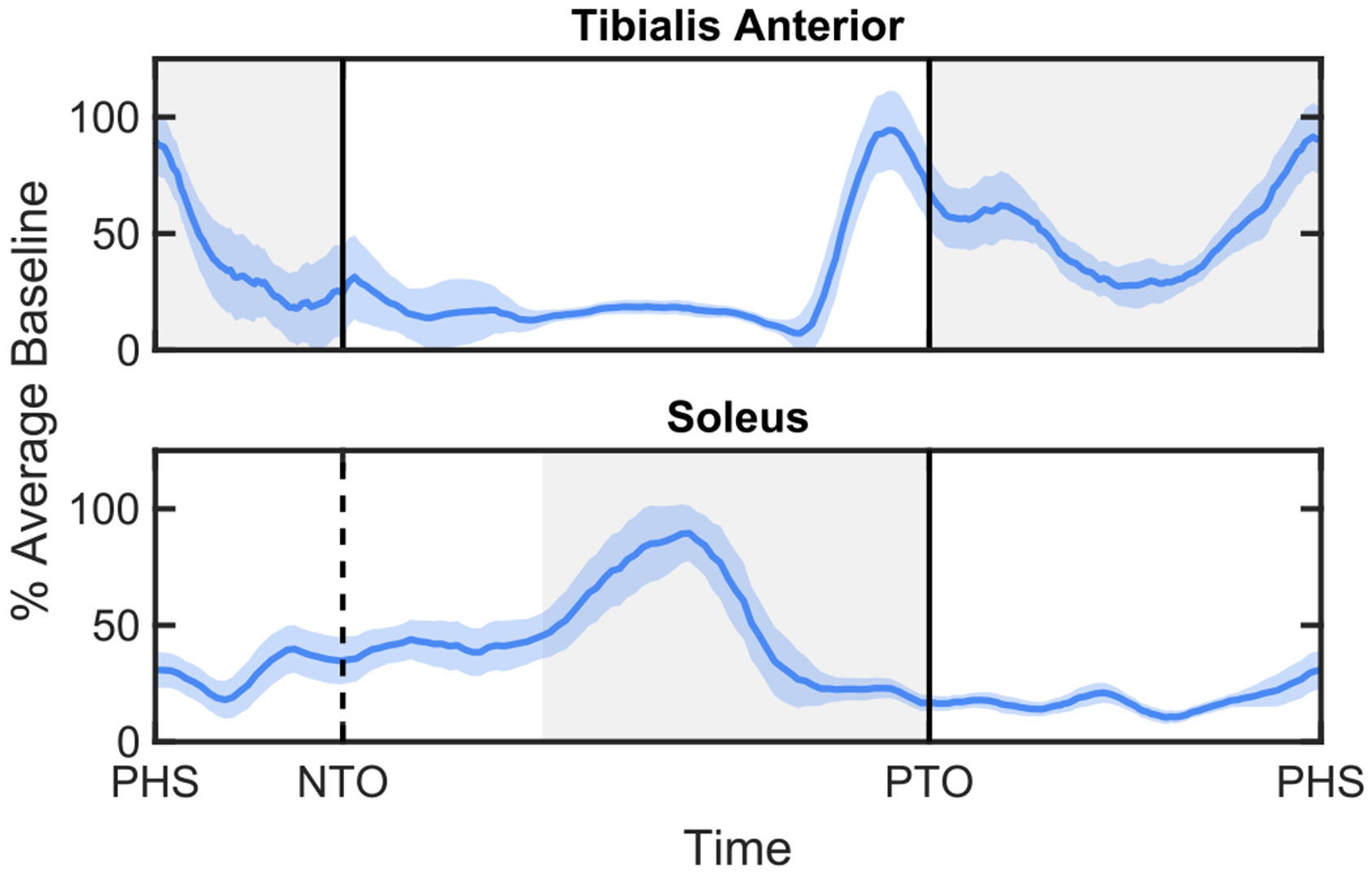
Resampling of Tibialis Anterior (TA) and plantarflexor (eg. soleus) muscle activations based on gait events (paretic heel strike - PHS, non-paretic toe-off - NHS, and paretic toe-off - PHS). The gray area indicates the region(s) used to measure average muscle activation for each gait cycle of that muscle.

EMG activation was measured as the average EMG linear envelope during the respective region of interest for each muscle. EMG activation for the TA was measured as the average signal surrounding HS (i.e., 0-100% of swing + 0-100% of double support after HS of the same leg). For the other three muscles, activation was measured as the average during the second half of stance (Fig. 2). TA activation was also measured during the second half of stance to measure co-contraction during push-off, referred to as TA co-contraction (TACC). The EMG was normalized by the average value of the peak EMG activation during the region of interest over the last minute of baseline.

**Table:** 

Algorithm 1 Belt Acceleration Control Logic
$$\begin{array}{cc} \; & \;\;\;\;\mathrm{Constants}:\\ \; & \;\;\;\;T_{\text{antBase}}\leftarrow 175\;\text{ms}\\ \; & \;\;\;\;T_{\text{TOT}}\leftarrow 220\;\text{ms}\\ \; & \;\;\;\;T_{\text{HSdelay}}\leftarrow 70\;\text{ms}\\ \; & \;\;\;\;T_{\max\text{Dur}}\leftarrow 250\;\text{ms}\\ \; & \;\;\;\;\mathrm{Define}\;\mathrm{Anticipation}\;\mathrm{Factor}:\\ \; & \;1:\;\mathrm{if}\;\nu \leq 0.5\;m∕s\;\mathrm{then}\\ \; & \;2:\;\;\;T_{\text{ant}}\leftarrow 0\\ \; & \;3:\;\mathrm{else}\;\mathrm{if}\;0.5<\nu <m∕s\;\mathrm{then}\\ \; & \;4:\;\;\;T_{\text{ant}}\leftarrow T_{\text{antBase}}(2\nu -1)\\ \; & \;5:\;\mathrm{else}\\ \; & \;6:\;\;\;T_{\text{ant}}\leftarrow T_{\text{antBase}}\\ \; & \;7:\;\mathrm{end}\;\mathrm{if}\\ \; & \;\;\;\mathrm{Belt}\;\mathrm{Acceleration}\;\mathrm{Control}\;\mathrm{Logic}:\\ \; & \;8:\;\mathrm{if}\;t>(T_{\text{TO},\text{pred}}-T_{\text{TOT}})\;\mathrm{then}\\ \; & \;9:\;\;\;\mathrm{if}\;(\nu >0.5\;m∕s)\;\mathrm{and}\;(T_{\text{HS},\text{pred}}-T_{\text{ant}}\leq t<T_{\text{HS},\text{pred}}+\\ \; & \;\;\;\;\;T_{\text{HS},\text{pred}})\;\mathrm{then}\\ \; & \;10:\;\;\;\;\;\mathrm{if}\;t=(T_{\text{HS},\text{pred}}-T_{\text{ant}})\;\mathrm{then}\\ \; & \;11:\;\;\;\;\;\;\;t_{\text{accel}}\leftarrow t\\ \; & \;12:\;\;\;\;\;\mathrm{end}\;\mathrm{if}\\ \; & \;13:\;\;\;\;\;\text{Accelerate belt}\\ \; & \;14:\;\;\;\mathrm{else}\;\mathrm{if}\;[(\nu <0.5\;m∕s)\;\mathrm{and}\;HS_{\text{cont}}]\;\mathrm{or}\\ \; & \;\;\;\left[(\nu >0.5\;m∕s)\;\text{and}\;\left(t\geq T_{\text{HS},\text{pred}}+T_{\text{HSdelay}}\right)\right]\\ \; & \;\;\;\mathrm{Then}\\ \; & \;15:\;\;\;\;\;\mathrm{if}\;TO_{\text{detected}}\;\mathrm{then}\\ \; & \;16:\;\;\;\;\;\;\;\text{End belt acceleration}\\ \; & \;17:\;\;\;\;\;\mathrm{else}\;\mathrm{if}\;\mathrm{not}\;HS_{\text{cont}}\;\mathrm{or}\;(t>t_{\text{accel}}+T_{\max\text{Dur}})\;\mathrm{then}\\ \; & \;18:\;\;\;\;\;\;\;\text{Pause accelerations for three steps}\\ \; & \;19:\;\;\;\;\;\mathrm{else}\\ \; & \;20:\;\;\;\;\;\;\;\mathrm{if}\;(\nu \leq 0.5\;m∕s)\;\mathrm{and}\;HS_{\text{cont},\text{evt}}\;\mathrm{then}\\ \; & \;21:\;\;\;\;\;\;\;\;\;t_{\text{accel}}\leftarrow t\\ \; & \;22:\;\;\;\;\;\;\;\mathrm{end}\;\mathrm{if}\\ \; & \;23:\;\;\;\;\;\;\;\text{Accelerate belt}\\ \; & \;24:\;\;\;\;\;\mathrm{end}\;\mathrm{if}\\ \; & \;25:\;\;\;\mathrm{else}\\ \; & \;26:\;\;\;\;\;\text{Do not accelerate belt}\\ \; & \;27:\;\;\;\mathrm{end}\;\mathrm{if}\\ \; & \;28:\;\mathrm{else}\\ \; & \;29:\;\;\;\text{Do not accelerate belt}\\ \; & \;30:\;\mathrm{end}\;\mathrm{if} \end{array}$$

Post-processed EMG profiles were subject to visual screening and automatic outlier removal to only retain measurements not affected by common EMG artifacts, such as loss of electrode-skin contact due to sweat, improper EMG electrode placement, impact artifacts, and lack of any measurable electromyographic signal in heavily impaired participants. EMG activation across all strides was overlaid for visual inspection. If the data exhibited excessive noise throughout the entire stride (characterized by numerous irregular peaks or a complete absence of peaks) the corresponding muscle was excluded from analysis for that session. The number of complete sessions removed from each condition for the four muscles, based on visual screening, is summarized by leg in Table I. The remaining sessions were then processed using an automated outlier removal method to identify individual strides with excessive noise and remove them from EMG activation calculations. The procedure was as follows: first, the maximum activation value within the region of interest was extracted for each stride. Then, the mean and standard deviation of maximum activation across the trial was calculated. This operation was conducted after removing the top and bottom 5% of these values to reduce the influence of extreme outliers that would distort the distribution. Based on the calculated means and standard deviations, we identified and removed strides where the maximum activation was above or below 10 standard deviations from the mean. This process was repeated using the average activation value during the region of interest, and the value at each data point within the stride (300 points for TA; 200 points for SOL, LGAS, MGAS, and TACC) to remove strides with impact artifacts. On a stride-by-stride basis, the automatic outlier removal process removed 7.33% of strides across all participants.

To evaluate changes in push-off posture, we calculated TLA as the angle between the vertical and the line between the hip and ankle markers at TO [32]. Stride length (SL) is known to be related to TLA [36], and can be measured via the distance between the start and end location of one’s stride on a treadmill. We calculated SL using the following equation:

(2) $$SL=\Delta P_{\text{HS}}+\int_{t_{\text{HS},\text{prev}}}^{t_{\text{HS},\text{curr}}}v\;dt$$

where $\Delta P_{\text{HS}}$ is the difference in antero-posterior position of the ankle marker between the current HS and the previous HS, $t_{\text{HS},\text{prev}}$ is the time of the previous HS, $t_{\text{HS},\text{curr}}$ is the time of the current HS, and ν is the velocity of the treadmill belt between HS events. Motion capture trajectories from all strides were overlaid to visually identify sessions with excessive marker jumps or missing data. Sessions with such issues were entirely excluded from the analysis and are reported in Table I. After visual outlier removal, the remaining sessions were then visually screened on a stride-by-stride basis to remove any individual strides affected by missing markers or marker jumps that could impact the calculation of TLA or SL. On a stride-by-stride basis, the visual screening of motion capture trajectories removed 3.82% of strides across all participants.

Because the duration of Post-Exposure was different across participants, we resampled the Post-Exposure data for all outcome measurements into 0-100% of Post-Exposure to compare effects between participants.

### E. Statistical Analysis

We used JMP Pro Version 17 (SAS Institute Inc., Cary, NC, USA) to run a linear mixed model analysis with condition, time point, and their interaction as fixed effects. Random effects included participant, the interaction between participant and condition, and the interaction between participant and timing. Outcomes were averaged across all strides within specific bins, referred to as “time points”: Baseline (BL, defined as the last minute of baseline), Early Exposure (EE, including the first 20 strides of exposure), Late Exposure (LE, including the last 20 strides of exposure), Early Post-Exposure (EPE, including the first 25% of strides of post-exposure data), and Late Post-Exposure (LPE, including the last 25% of strides of post-exposure data). Separate models were run for the paretic and non-paretic legs.

If the interaction between condition and timing was significant (*p* < 0.05), we evaluated simple main effects using Dunnett’s test to compare each time point to BL within each condition. To interpret the interaction, we ran statistical contrasts to determine if the change from baseline was different between conditions at each time point. We used a Bonferroni correction for four contrasts (outcomes at 4 time points compared to baseline) resulting in a threshold of *p_th_* = 0.0125 (0.05/4) to mantain a false-positive rate of α = 0.05. In absence of a significant interaction, within-condition changes from BL were identified using Dunnett’s test. If only the main effect of timing was significant, the Dunnett’s test was used on the pooled data to compare the outcome at each time point to the pooled BL.

To determine if baseline impairment affected differences in propulsion between conditions, we ran a linear mixed model analysis with condition, FMLE score, and their interaction as fixed effects, and subject as a random effect. For each leg, this model was run twice per outcome: once for the difference between LE and BL, and once for the difference between LPE and BL. If the interaction between condition and FMLE was significant, we calculated regression slopes between FMLE and the change in outcome for each condition and compared the slopes using pairwise tests. For outcomes where only FMLE was significant, we reported the average regression slope across both conditions.

## III. Results

### A. Velocity

**Fig. 3.**
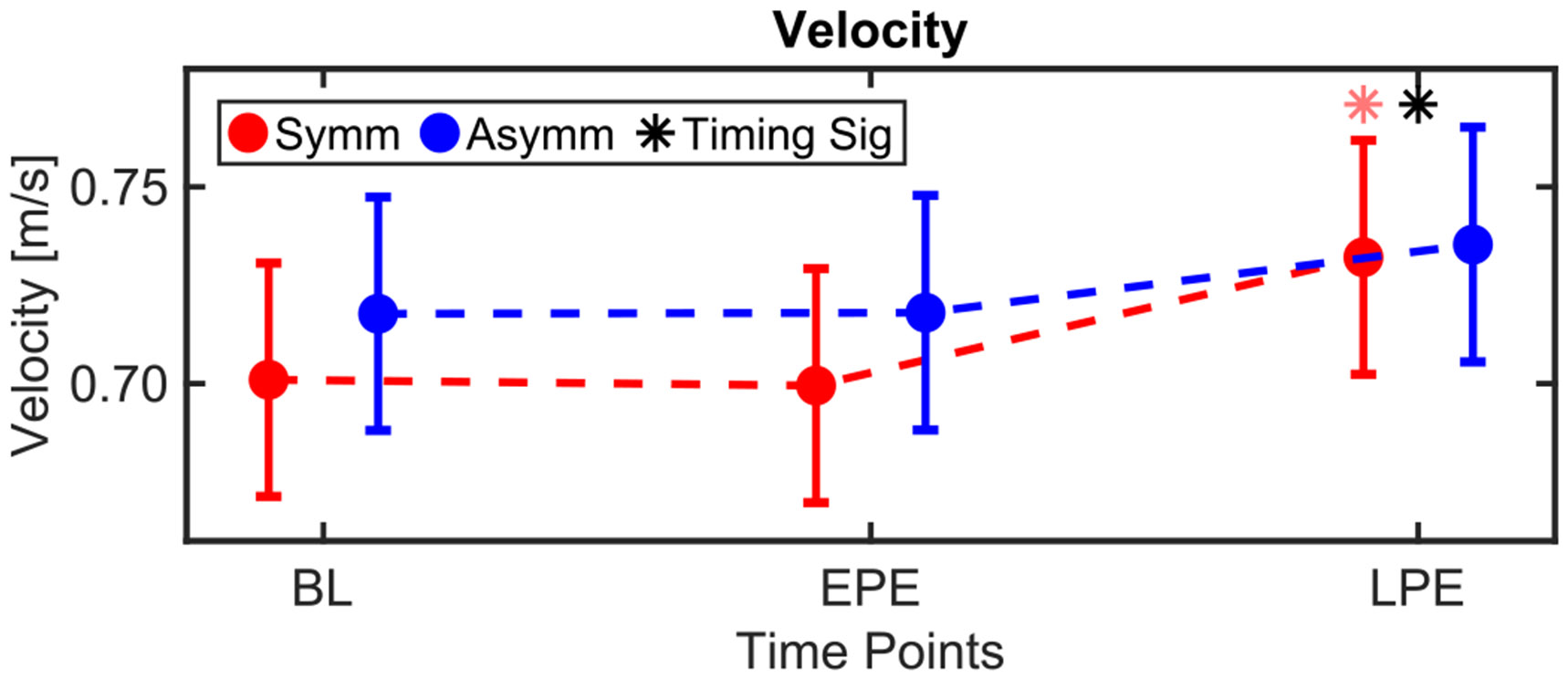
Model-estimated least-square means of gait speed. Dots indicate the group means, and whiskers extend to one s.e.m. Black asterisks indicate statistically significant changes from BL according to Dunnett’s post hoc test (only run if timing was a significant main effect in the mixed model). Red/blue asterisks indicate statistically significant changes from BL within the respective condition. The asterisk is dark/light in presence/absence of a significant interaction between condition and timing.

Because velocity was imposed during Exposure, we only evaluated changes during Post-Exposure relative to BL. Timing was a significant main effect for velocity (*p* = 0.016), as the average velocity across both conditions increased during LPE relative to BL (mean ± s.e.m.: 2.44 ± 0.96 cm/s, *p* = 0.025), Fig. 3. A similar change was observed in the symmetric condition (3.07 ± 1.32 cm/s, *p* = 0.043).

### B. Propulsion

#### 1) Peak AGRF:

**Fig. 4.**
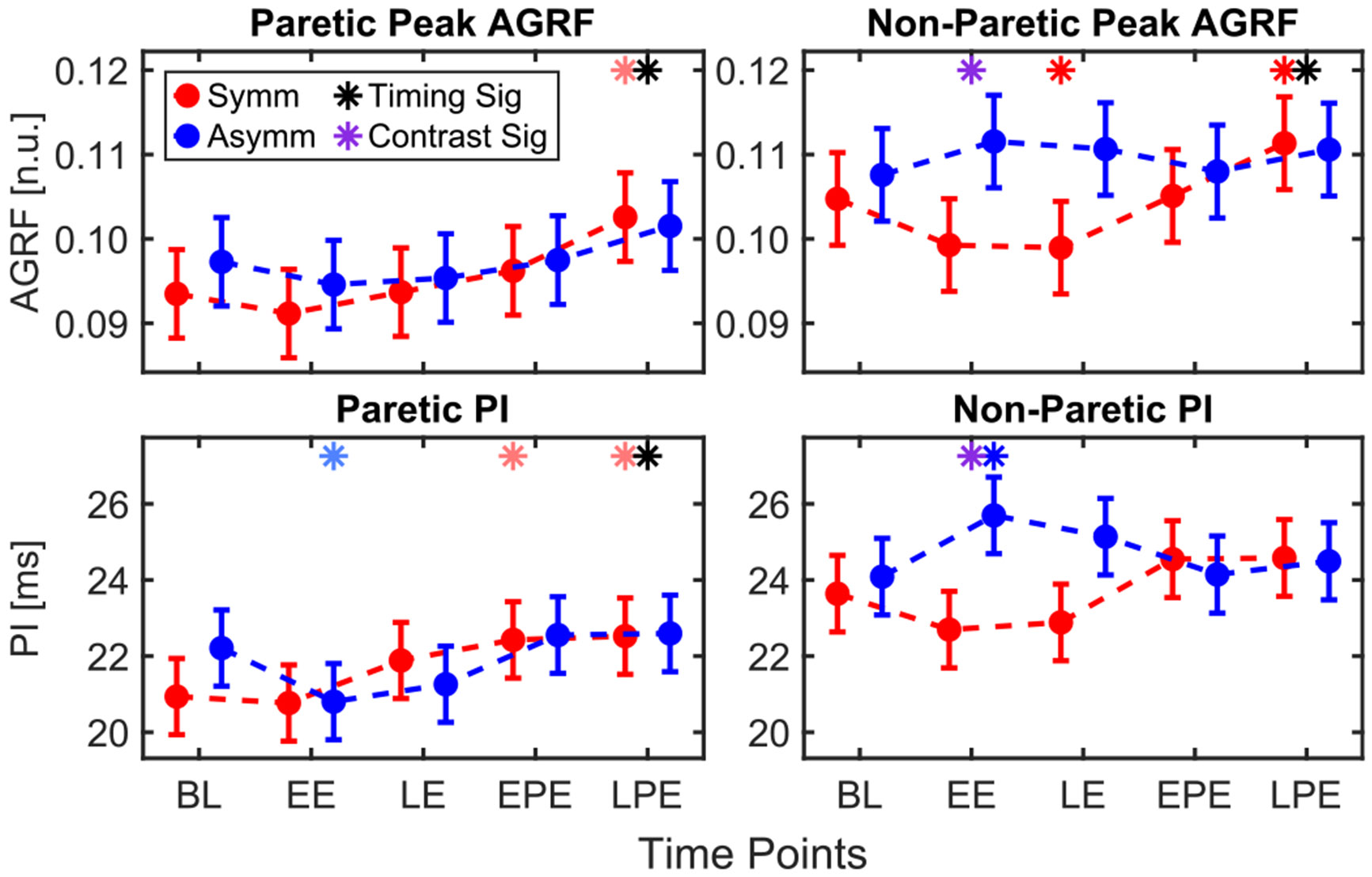
Model-estimated least-square means for peak AGRF and PI for paretic and non-paretic legs. Purple asterisks indicate time points where the statistical contrast between change from BL between the two conditions was significant (only run in presence of a significant interaction).

All changes in AGRF values are reported in units of ×10^−3^. Timing had a significant effect on peak AGRF in the paretic leg (*p* < 0.001), with increases observed across both conditions during LPE (6.66 ± 1.51, *p* < 0.001), relative to BL (Fig. 4). Within the symmetric condition, AGRF also increased significantly during LPE relative to BL (9.03 ± 2.10, *p* < 0.001).

In the non-paretic leg, timing (*p* = 0.001) and the interaction between condition and timing (*p* = 0.001) were significant factors. Within the symmetric condition, there was a significant decrease in peak AGRF during LE (−5.78 ± 2.26, *p* = 0.040); however, there was a significant increase during LPE (6.58 ± 2.28, *p* = 0.016). The change in non-paretic peak AGRF at EE relative to BL was greater in the asymmetric condition than in the symmetric condition (4.71 ± 1.84 ms, *p* = 0.011).

#### 2) Propulsive Impulse:

In the paretic leg, timing was the only significant factor affecting PI (*p* < 0.001). Dunnett’s post hoc test revealed a significant increase during LPE relative to BL (0.99 ± 0.38 ms, *p* = 0.035) across conditions. Within the symmetric condition, PI increased during Post-Exposure relative to BL (EPE: 1.48 ± 0.52 ms, *p* = 0.019; LPE: 1.58 ± 0.52 ms, *p* = 0.011). Within the asymmetric condition; however, there was a significant decrease during EE relative to BL (−1.41 ± 0.52 ms, *p* = 0.028).

In the non-paretic leg, there was a significant effect of condition (*p* = 0.040) and the interaction between condition and timing (*p* < 0.001). In the symmetric condition, PI did not significantly change from BL at any time point; however, in the asymmetric condition, PI increased during EE (1.61 ± 0.54 ms, *p* = 0.013). The change in PI at EE relative to BL was greater in the asymmetric condition than in the symmetric condition (1.28 ± 0.39 ms, *p* = 0.001).

### C. Muscle Activation

#### 1) Soleus:

**Fig. 5.**
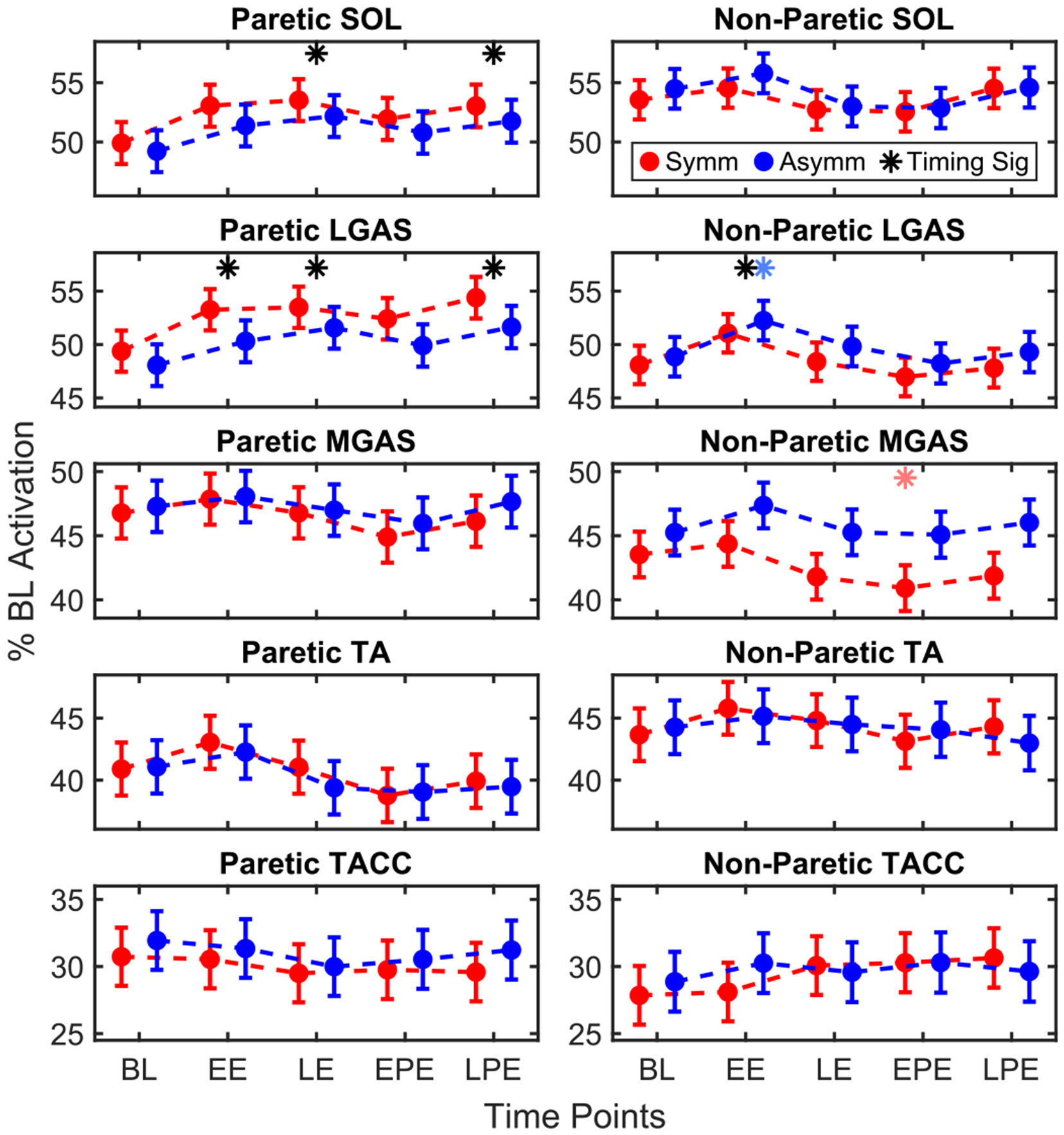
Model-estimated least square means for paretic and non-paretic muscle activation.

Timing was a significant factor for SOL activation in the paretic (*p* = 0.025) and non-paretic legs (*p* = 0.033). In the paretic leg, SOL activation increased during LE (3.29 ± 1.08, *p* = 0.010) and LPE (2.83 ± 1.11, *p* = 0.041) relative to BL, Fig. 5. In the non-paretic leg, Dunnett’s post hoc did not indicate any significant changes from BL.

#### 2) Lateral Gastrocnemius:

Timing significantly affected LGAS activation in both the paretic (*p* = 0.002) and nonparetic legs (*p* < 0.001). In the paretic leg, LGAS increased during Exposure (EE: 3.06 ± 1.11, *p* = 0.025; LE: 3.81 ± 1.11, *p* = 0.003) and LPE (4.29 ± 1.13, *p* < 0.001), relative to BL. In the non-paretic leg, there was an increase during EE (3.18 ± 0.95, *p* = 0.004), driven by the asymmetric condition (3.39 ± 1.11, *p* = 0.010).

#### 3) Medial Gastrocnemius:

Timing significantly affected MGAS activation in the non-paretic leg (*p* = 0.008). The post hoc Dunnett’s test did not reveal any significant changes from BL; however, there was a notable decrease during EPE in the symmetric condition relative to BL (−2.62 ± 1.03, *p* = 0.042).

#### 4) Tibialis Anterior:

Timing was a significant factor for paretic TA activation (*p* < 0.001); however, no significant changes relative to BL were observed at any time point. There were no significant effects in the non-paretic leg.

#### 5) Tibialis Anterior Co-Contraction:

There were no significant effects on TACC in either leg.

### D. Push-Off Posture

#### 1) Trailing Limb Angle:

**Fig. 6.**
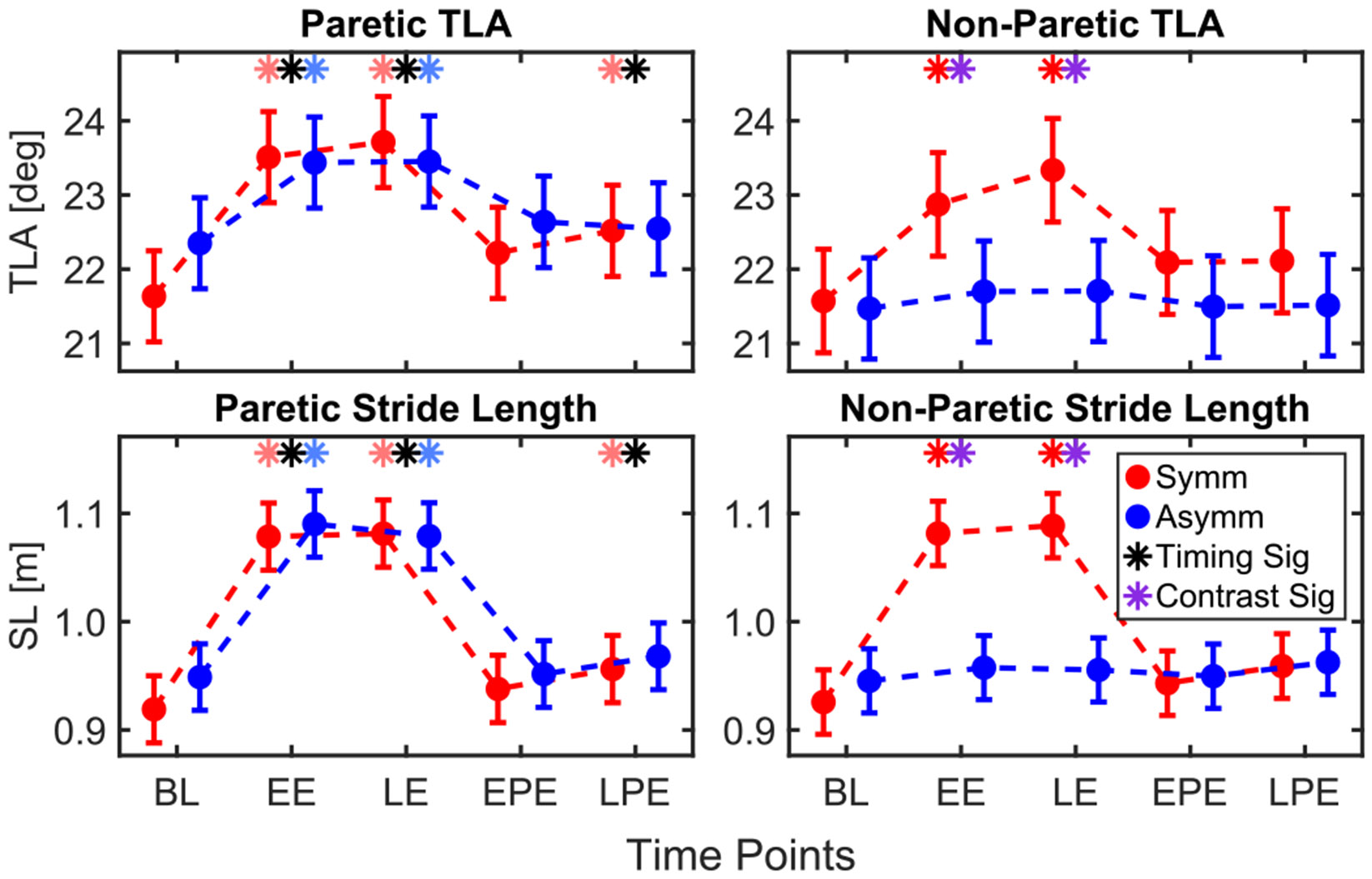
Model-estimated least square means for metrics of push-off posture for paretic and non-paretic legs.

Timing had a significant effect on paretic TLA (*p* < 0.001). TLA increased significantly during Exposure (EE: 1.48 ± 0.20 deg, *p* < 0.001; LE: 1.59 ± 0.20 deg, *p* < 0.001) and LPE (0.54 ± 0.21 deg, *p* = 0.036), Fig. 6. Within the symmetric condition, TLA was larger during Exposure and LPE (EE: 1.88 ± 0.28, *p* < 0.001; LE: 2.08 ± 0.28, *p* < 0.001; LPE: 0.88 ± 0.28, *p* = 0.008). Within the asymmetric condition, TLA also increased during Exposure (EE: 1.09 ± 0.30, *p* = 0.002; LE: 1.10 ± 0.30, *p* = 0.001).

In the non-paretic leg, both timing (*p* < 0.001) and the interaction between condition and timing (*p* = 0.002) were significant factors. Increases in TLA were identified during Exposure in the symmetric condition (EE: 1.30 ± 0.26 deg, *p* < 0.001; LE: 1.76 ± 0.26 deg, *p* < 0.001). The change in TLA relative to BL was greater in the symmetric condition than the asymmetric condition during EE (0.54 ± 0.19 deg, *p* = 0.007) and LE (0.76 ± 0.19 deg, *p* < 0.001).

#### 2) Stride Length:

Timing was the only significant factor that affected paretic SL (*p* < 0.001). SL increased during EE relative to BL (15.04 ± 1.05 cm, *p* < 0.001), LE (14.63 ± 1.05 cm, *p* < 0.001), and LPE (2.82 ± 1.07 cm, *p* = 0.32). Within the symmetric condition, there were increases in SL relative to BL at the same time points (EE: 15.95 ± 1.45 cm, *p* < 0.001; LE: 16.20 ± 1.45 cm, *p* < 0.001; LPE: 3.66 ± 1.46 cm, *p* = 0.46). In the asymmetric condition, increases relative to BL were observed during Exposure (EE: 14.14 ± 1.41 cm, *p* < 0.001; LE: 13.03 ± 1.41 cm, *p* < 0.001).

For the non-paretic leg, condition (*p* = 0.008), timing (*p* < 0.001), and their interaction (*p* < 0.001) were all significant factors. SL increased relative to BL only in the symmetric condition during EE (15.58 ± 1.43 cm, *p* < 0.001) and LE (16.30 ± 1.43 cm, *p* < 0.001). The change in SL relative to BL was greater in the symmetric condition than the asymmetric condition during both EE (7.17 ± 1.02 cm, *p* < 0.001) and LE (7.64 ± 1.02 cm, *p* < 0.001).

### E. Stride & Stance Duration

#### 1) Stride Duration:

**Fig. 7.**
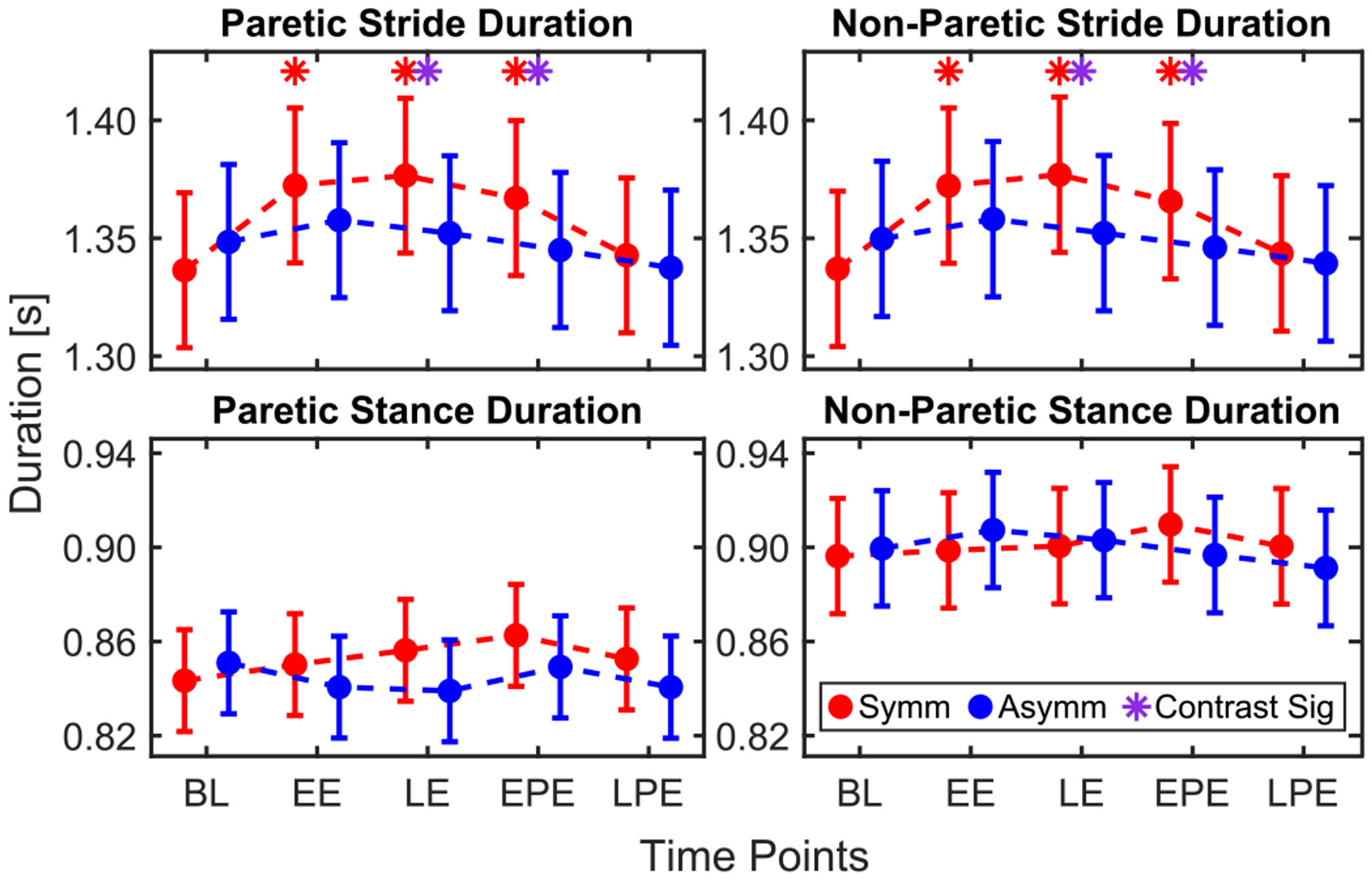
Model-estimated means for stride and stance duration for paretic and non-paretic legs.

For the paretic leg, timing (*p* = 0.003) and the interaction between condition and timing (*p* = 0.019) significantly affected stride duration. In the symmetric condition, stride duration increased relative to BL during EE (35.97 ± 10.57 ms, *p* = 0.003), LE (40.10 ± 10.57 ms, *p* < 0.001), and EPE (30.66 ± 10.68 ms, *p* = 0.017), Fig. 7. The increase in stride duration relative to BL was greater in the symmetric condition than in the asymmetric condition during LE (18.22 ± 5.96 ms, *p* = 0.003) and EPE (17.01 ± 6.06 ms, *p* = 0.006).

For the non-paretic leg, timing (*p* = 0.006) and the interaction between condition and timing (*p* = 0.023) were also significant. Increases in stride duration relative to BL were observed in the symmetric condition during EE (35.26 ± 10.81 ms, *p* = 0.005), LE (39.93 ± 10.81, *p* = 0.001), and EPE (28.76 ± 10.92 ms, *p* = 0.033). The increase in stride duration relative to BL was greater in the symmetric condition during LE (18.74 ± 6.09 ms, *p* = 0.003) and EPE (16.18 ± 6.19 ms, *p* = 0.010).

#### 2) Stance Duration:

The initial model did not report any significant effects for stance duration of either leg.

### F. Effects of Baseline Impairment

**Fig. 8.**
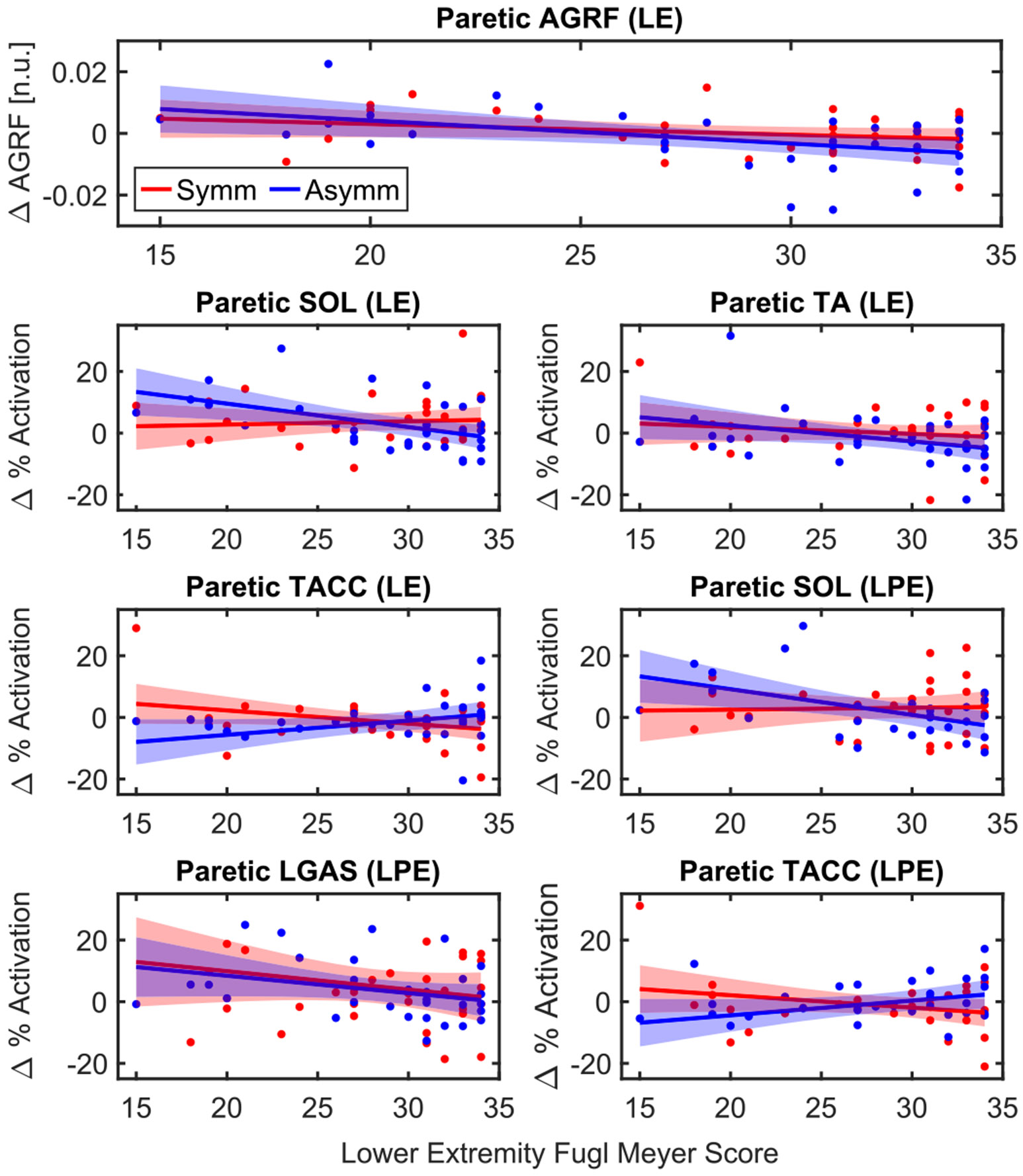
Relationships between FMLE score and change in outcome measurement from BL to either LE or LPE for both conditions. Outcomes/time points shown had a significant effect of either FMLE or the interaction between condition and FMLE in the paretic leg.

In the paretic leg, the interaction between FMLE score and condition was significant in SOL activation during LE (*p* = 0.010) and LPE (*p* = 0.028). During LE, the linear relationship between FMLE score and change in SOL activation in the symmetric condition was not significant, with a slope of 0.11 ± 0.26 units of muscle activation change per unit of baseline FMLE score (*p* = 0.679). In the asymmetric condition, the slope of −0.76 ± 0.26 was significant (*p* = 0.005). The slopes of the fitted lines for the two conditions were significantly different (0.87 ± 0.32, *p* = 0.010). Similarly, during LPE, the linear relationship FMLE score and change in SOL activation in the symmetric condition was not significant, with a slope of 0.09 ± 0.33 units of muscle activation change per unit of baseline FMLE score (*p* = 0.77). The slope of this relationship in the asymmetric condition was significant (−0.79 ± 0.30, *p* = 0.011). The two slopes for the different conditions were also significantly different from each other (0.89 ± 0.38, *p* = 0.028). This indicates that both during and after exposure, there was not much of an effect of baseline FMLE score on change in SOL activation in the symmetric condition; however, in the asymmetric condition, more impaired participants (participants with lower FMLE scores) exhibited larger increases than less impaired (higher FMLE score) participants exhibited larger increases than less impaired participants (Fig. 8).

The interaction between FMLE score and condition was also significant in TACC activation during LE (*p* = 0.003) and LPE (*p* = 0.009). During exposure, TACC activation was not significantly related to FMLE score in either condition (symm: −0.43 ± 0.24, *p* = 0.076; asymm: 0.46 ± 0.24, *p* = 0.059), although the slopes differed significantly between conditions (0.88 ± 0.27, *p* = 0.003). After exposure, neither condition resulted in a significant relationship between TACC activation and FMLE score (symm: −0.40 ± 0.27, *p* = 0.137; asymm: 0.48 ± 0.27, *p* = 0.085), but the slopes of the two lines were significantly different (0.88 ± 0.31, *p* = 0.009).

FMLE score had a significant effect on the change in peak AGRF during LE in the paretic leg (average slope = −0.55 ± 0.19 × 10^−3^, *p* = 0.006). This was also true for the change in activation of TA during LE (−0.37 ± 0.18, *p* = 0.044) and LGAS activation during LPE (−0.60 ± 0.25, *p* = 0.026). In all of these relationships, more impaired participants produced greater increases in muscle activation than less impaired participants across both conditions.

## IV. Discussion

We evaluated a novel paradigm to increase paretic propulsion in individuals post-stroke via posterior belt accelerations applied during push-off. In the two versions of the paradigm, one delivered belt accelerations to both legs (symmetric condition), and the other only delivered belt accelerations to the paretic leg (asymmetric condition). We hypothesized that (1) propulsion will change relative to baseline during and after exposure as a result of exposure to belt accelerations, (2) there will be differential effects in propulsion between symmetric and asymmetric conditions, and (3) baseline impairment will have an effect on the exposure and post-exposure effects of belt accelerations on propulsion mechanics.

In the paretic leg, there were significant changes in both components of propulsion during Exposure when conditions were grouped. TLA increased by (1.5 deg (7.2%)), as did SOL activation (6.6% of baseline value), and LGAS activation (7.8%). While meaningful, these responses are generally smaller than those reported in other interventions targeting paretic propulsion. For example, Kesar et al. reported a 40% increase in AGRF and a 3.8 deg (20.5%) increase in TLA using functional electrical stimulation at fast walking speeds [37]. In a biofeedback-based intervention, Santucci et al. increased TLA by 4–6.5 deg (32–52%) and ankle moment by 13% [19].

Both Kesar and Santucci included participants with greater baseline impairment than those in our cohort. This is important because individuals with more severe impairments tend to exhibit larger relative increases in propulsion. Additionally, baseline walking trials in prior studies were relatively brief, meaning the calculated baseline values may have included non-steady-state strides. Kesar et al. averaged multiple separate baseline trials to mitigate this issue [37]; however, Santucci et al. utilized data from all strides during a single baseline trial lasting between 20-60 seconds [19]. In our study, baseline metrics were based only on strides from the final minute of the Baseline phase, after steady-state walking was achieved. Alternatively, Swaminathan et al. reported gains comparable to ours using a similar task-specific resistance paradigm targeting plantarflexor muscles with an ankle exoskeleton, with AGRF increasing 8–14%, TLA increasing by 0.5–0.6 deg (2.5–3%) depending on resistance level, and SOL activation increasing by 7% in the high-resistance condition [24]. Their participants had impairment levels similar to ours due to the endurance needed to complete their experiment, and they only used data from the second half of a one-minute baseline period to ensure steady-state comparisons.

Immediately after Exposure, no outcome changed significantly from baseline, except for an increase in stride duration in the symmetric condition, likely contributing to the rise in paretic PI observed in the symmetric condition. The increase in paretic propulsion during Early Post-Exposure across conditions (4.27%) is smaller than other propulsion training protocols have been able to achieve in individuals post-stroke immediately after intervention such as Lewek et al. (25%) and Swaminathan et al. (8.8-14%) [21], [24]. Both the change in paretic AGRF (0.28% BW) and PI (0.15% BWs) are less than within-session minimal detectable changes (MDC) for individuals post-stroke (0.80% BW and 0.24% BWs, respectively) [27]. Due to the velocity change limits imposed by the adaptive treadmill controller, we did not expect to see immediate (i.e. within the first 5 strides) changes in propulsion post-exposure. Once enabled, the controller introduced fluctuations in speed that required multiple strides before participants were able to stabilize at their new comfortable walking speed.

Toward the end of the Post-Exposure phase, increases in plantarflexor activation (SOL: 5.7%, LGAS: 8.8%), TLA (0.5 deg or 2.5%), and overall propulsion (AGRF: 7.0%, PI: 4.6%) in the paretic leg were observed across both conditions. Most of these outcomes also increased during the Exposure phase, and their persistence suggests short-term adaptation or retention of the modified gait strategy utilized during Exposure. While the magnitude of these after-effects was smaller than those reported in previous studies, such as Genthe et al. (25% increase in plantarflexor moment, 3 deg or 21% increase in TLA, and 50% increase in AGRF) [16], and Park et al. 2023 (16% increase in AGRF after a 10-minute break) [23], and Park et al. 2025 (25% increase in SOL activation, 25-26% increase in MGAS activation, and 33-38% increase in AGRF) [20], our results still demonstrate significant improvements in propulsion. Specifically, paretic AGRF in the symmetric condition increased by 0.92% BW, which is greater than the 0.80% BW within-session MDC for individuals post-stroke [27]. The discrepancy in the magnitude of measured effects may be attributed to the fact that these other studies involve explicit learning or apply large forces to challenge propulsion during training, whereas our intervention is an implicit paradigm based on subtle changes in propulsion mechanics during push-off.

Similar to other measurements, the increase in velocity during LPE across both conditions (2.45 cm/s or 3.5%) is mainly driven by the symmetric condition. This increase is less than the MDC for overground walking speed in individuals post-stroke reported by Hosoi et al.(11 cm/s for moderate speeds, 0.4–0.8 m/s) [38]. It is also less than the 6 cm/s (8.5%) improvement reported by Park et al. [23]; however, it aligns with previous studies demonstrating a positive association between increased propulsion and walking velocity [10], [11], [12], [13].

In partial support of our second hypothesis, we observed different effects on propulsion mechanics between conditions, primarily during exposure. In the paretic leg, effects in the kinematic measures of propulsion were observed consistently in both conditions. In the non-paretic leg, however, the differences between conditions were more apparent. For example, AGRF and PI increased in the asymmetric condition but decreased in the symmetric condition during Early Exposure. This suggests that participants initially relied on their non-paretic leg when the belt accelerations were introduced in the asymmetric condition (both because it is already the more stable leg, and because the non-paretic leg is not being accelerated and is hence steadier than their accelerated, paretic leg). Unfortunately, this type of compensation via the non-paretic leg is undesirable in post-stroke gait rehabilitation, as it is metabolically inefficient and puts additional stress on other joints [5], [6]. The decreases in AGRF and PI between Early and Late Exposure in the asymmetric condition suggest that participants became more comfortable walking with belt accelerations and relied less on their non-paretic leg as the protocol progressed, but not enough to return at least to their baseline propulsion levels until after exposure.

There is a clear contrast in the effect of different conditions on the kinematic component of non-paretic propulsion. During the symmetric condition, both TLA and SL increased during Exposure, whereas no significant changes occurred in the asymmetric condition. Alternatively, the paretic leg (which was accelerated during Exposure in both conditions) showed large kinematic increases in both conditions. These results suggest that condition influences non-paretic kinematics due to the presence or absence of belt acceleration, which directly affects limb movement. Conversely, condition did not have a significant effect on the propulsion mechanics of the paretic leg. This similarity is likely due to the fact that the paretic leg was accelerated in both conditions, although the responses were often larger in the symmetric condition.

Due to the heterogeneity in the post-stroke population, we investigated the relationships between FMLE scores and changes in metrics of propulsion mechanics relative to baseline. In general, this analysis highlighted that more impaired individuals will exhibit greater positive changes in paretic propulsion, specifically in plantarflexor muscle activation, during and after exposure to belt accelerations. This suggests that belt accelerations may effectively target an individual’s muscle activation based on their level of impairment, promoting larger improvements in those with greater deficits. During LPE, these effects were most evident in the plantarflexors, which could be mediated by the increased walking velocity observed at this time, following exposure to belt accelerations. The current study design does not allow us to determine whether the increase in muscle activation led to the increase in velocity, or whether velocity increased due to greater TLA, which then required more muscle activation to maintain speed.

We then sought to quantify whether individuals with different baseline impairment would respond more to one condition or the other. We observed that more impaired participants had greater increases in SOL activation during LE and LPE in the asymmetric condition than in the symmetric condition. The opposite is true for TACC, where more impaired participants had less activation in TA activation during push-off in the asymmetric condition, suggesting inhibition of the dorsiflexors to generate greater plantarflexor activation.

Our previous work exposed healthy young adults to belt accelerations at a high acceleration (7 m/s^2^) [32]. Similar to the current study, there was a significant increase in velocity during Late Post-Exposure (7.3 ± 1.3 cm/s). In the present study, however, the observed increase in velocity (2.45 ± 0.96 cm/s) was smaller and falls below the threshold for clinical significance [39]. This small increase may be partially explained by participants’ discomfort with the adaptive treadmill controller. While the changes in gait speed are small, they raise the possibility that with repeated exposure, more meaningful changes than observed here could be achieved.

Our study with healthy young adults demonstrated a significant increase in TLA during Exposure in the high acceleration group (EE: 0.80 ± 0.14 deg; LE: 0.99 ± 0.14 deg) [32]. In contrast, individuals post-stroke in the symmetric condition exhibited larger changes (EE: 1.88 ± 0.28 deg, LE: 2.08 ± 0.28 deg). This difference likely indicates that the training paradigm drew more heavily on the kinematic reserve for propulsion in individuals post-stroke compared to healthy subjects [21]. Because the training paradigm challenges force production but assists the kinematic component, and since individuals post-stroke may have greater difficulty in increasing propulsion via the kinetic component (difficulty increasing ankle plantarflexor activation), participants likely only generated the minimal additional force needed, and instead relied on increased TLA to enhance propulsion. Future iterations of this study will attempt to more specifically modulate the effects on the two components of propulsion during exposure.

There are a few limitations of this study that must be taken into consideration. As stated above, many participants found it challenging to find a consistent walking speed on the adaptive treadmill, especially in the Post-Exposure phase. When tuning the gains of the controller, we focused on ensuring that the treadmill felt responsive when walking at slower speeds (~0.4 m/s), which we achieved by increasing the sensitivity. It is possible that this increased sensitivity was ideal for steady-state walking conditions, but not for the transition between belt accelerations and adaptive speed control Post-Exposure. While the user-driven adaptive treadmill paradigm used here offers advantages during immediate post-training evaluation, such as ecological validity and temporal resolution, it also introduces limitations in the late post-training assessment. In particular, difficulty achieving steady-state walking post-training likely increased the error with which self-selected walking speed was measured. The outcome may have been biased downward due to fatigue-related reductions in preferred speed, or upward if delayed changes in belt velocity resulted in brief instability, forcing participants to temporarily exceed their preferred speed before they asked to terminate the trial. Future studies may mitigate these effects by decoupling training and evaluation (e.g., allowing rest before repeating baseline assessments), by more extensively tuning the adaptive controller gains in the target population for a variety of stationary and non-stationary walking conditions, or by evaluating both baseline and post-training performance during overground walking.

Also, in this study, the participant population consisted of individuals mostly in the mild-medium level of impairment; 59% had mild impairment (FMLE score ≥ 29), and 29% had medium impairment (FMLE score between 20 and 28). Given the observed negative relationships between baseline impairment and training effects, it is possible that a future study targeting a more impaired population would demonstrate larger training effects. Due to the duration of the protocol, and the requirement for participants to walk continuously for 15 minutes, we were not able to recruit and retain as many participants with lower FMLE scores. Many of our participants walked at faster speeds than expected (0.6-0.8 m/s) and may not have had a large propulsion reserve, yielding to an underestimate of the effects of this training paradigm (see how the effects on walking mechanics scale with baseline impairment). We also had not considered that participants would compensate with their non-paretic leg during the asymmetric condition when designing the study. Given our observation, a hypothetical training mode where we only accelerated the non-paretic leg may also have resulted in greater use of the paretic leg due to a reduction in stability of the non-paretic leg.

## V. Conclusion

Belt accelerations required individuals post-stroke to increase paretic propulsion during walking practice. The changes in propulsion mechanics of the paretic leg were similar in both conditions (symmetric and asymmetric), likely because the paretic leg was accelerated in both conditions. The non-paretic leg did show evidence of significant differences between conditions, particularly in the kinematic components of propulsion, suggesting that accelerating the leg directly affects outcomes such as trailing limb angle and stride length. The asymmetric condition encouraged participants to utilize their non-paretic leg instead of solely targeting the paretic leg as intended, meaning that the symmetric condition may be a better training paradigm, suitable for challenging participant to produce more metabolically efficient walking and dissuade compensation via the non-paretic leg. Effects on the kinetic component of propulsion were greater in more impaired participants, suggesting that this training method may be more effective in tapping into the kinetic component of propulsion in individuals with greater baseline impairment.

## Supplementary Material

supp2-3675477

supp1-3675477

## Figures and Tables

**TABLE I:** Full Sessions Removed From Each Condition/Leg Mixed Model By Outcome Measure

	SOL	LGAS	MGAS	TA	TLA	SL
**Symmetric**						
Paretic	2	1	4	0	3	2
Non-Paretic	0	0	0	0	6	2
Both	0	0	0	0	2	1
**Asymmetric**						
Paretic	2	2	5	1	3	0
Non-Paretic	1	2	0	2	3	1
Both	1	1	0	1	2	0
