# Supplementary material for: Effects of Belt Accelerations During Push-Off on Propulsion Mechanics in Individuals Post-Stroke
Source: IEEE Trans Neural Syst Rehabil Eng. Author manuscript; Available in PMC 2026 Apr 27. (PMC13120362; doi:10.1109/TNSRE.2026.3675477)
Supplement: supp2-3675477 [file NIHMS2162889-supplement-supp2-3675477.pdf]

EMG Timeseries For All Participants

Effects of Belt Accelerations During Push-Off on Propulsion Mechanics in Individuals Post-Stroke

Hannah N. Cohen, Tamara Wright, GilHwan Kim, Henry Wright,
Darcy S. Reisman, and Fabrizio Sergi

Soleus

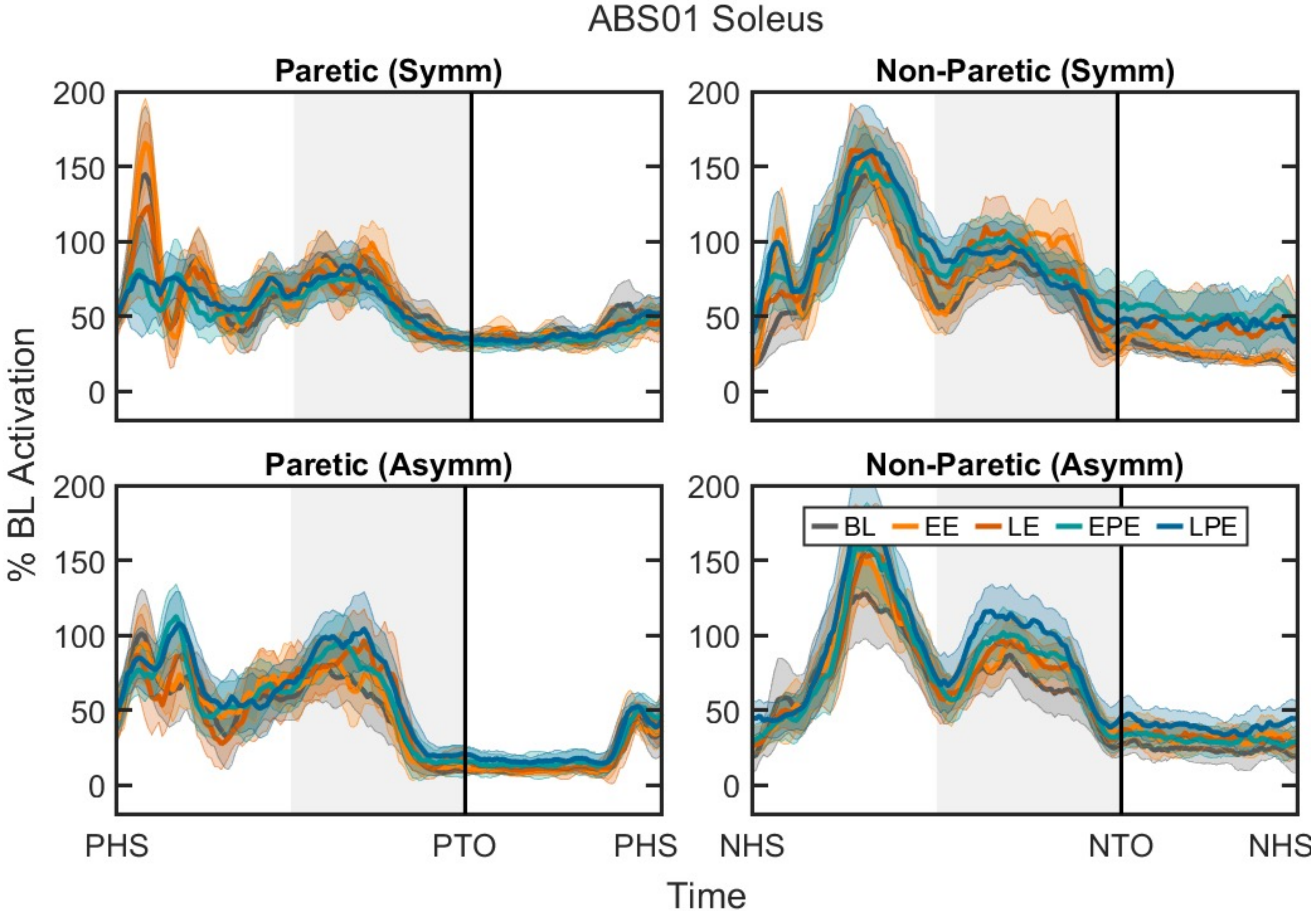
ABS01 Soleus
Paretic (Symm)
Non-Paretic (Symm)
Paretic (Asymm)
Non-Paretic (Asymm)
% BL Activation
200
150
100
50
0
BL
EE
LE
EPE
LPE
PHS
PTO
PHS
NHS
NTO
NHS
Time

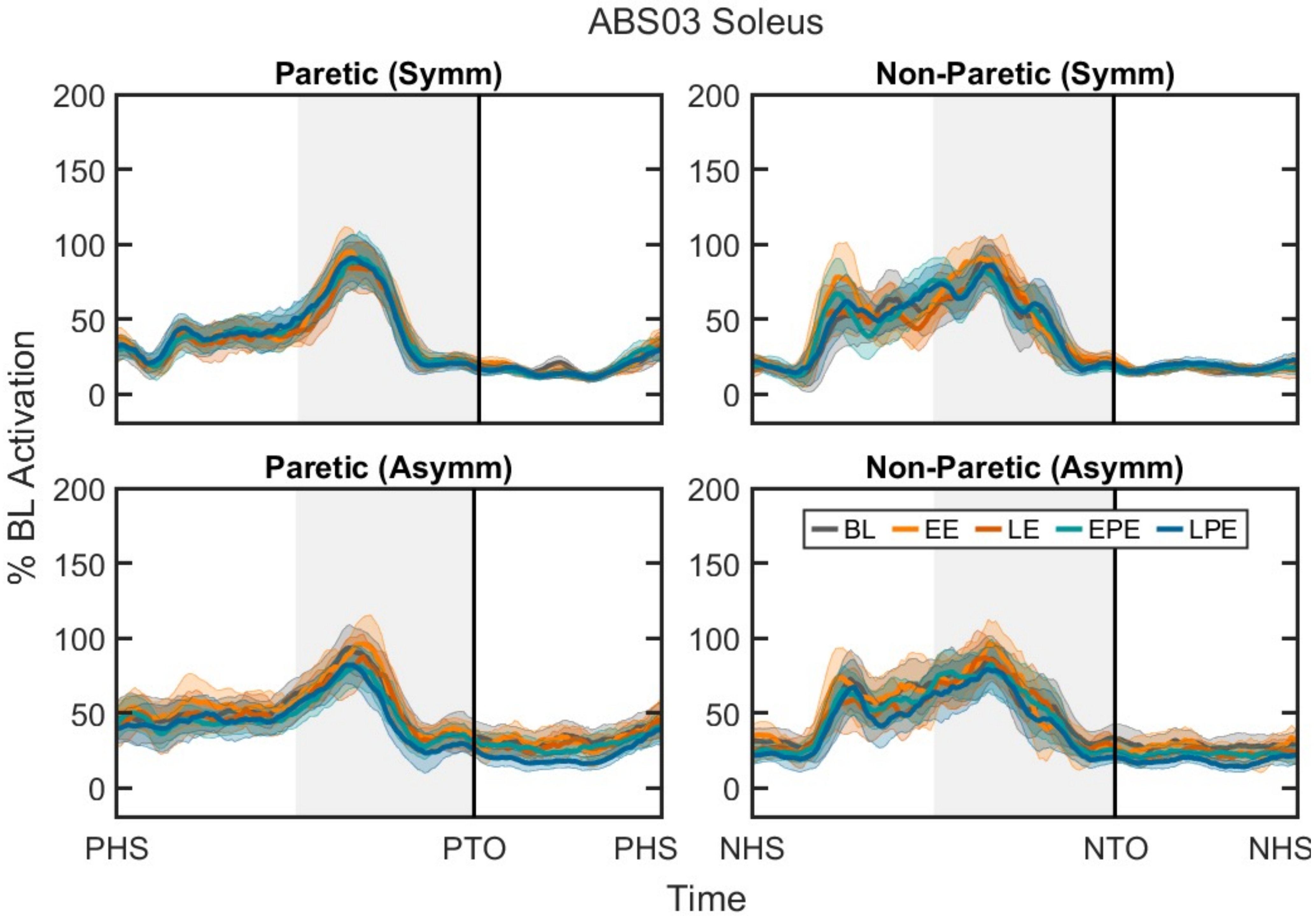
ABS03 Soleus
Paretic (Symm)
Non-Paretic (Symm)
Paretic (Asymm)
Non-Paretic (Asymm)
% BL Activation
200
150
100
50
0
BL
EE
LE
EPE
LPE
PHS
PTO
PHS
NHS
NTO
NHS
Time

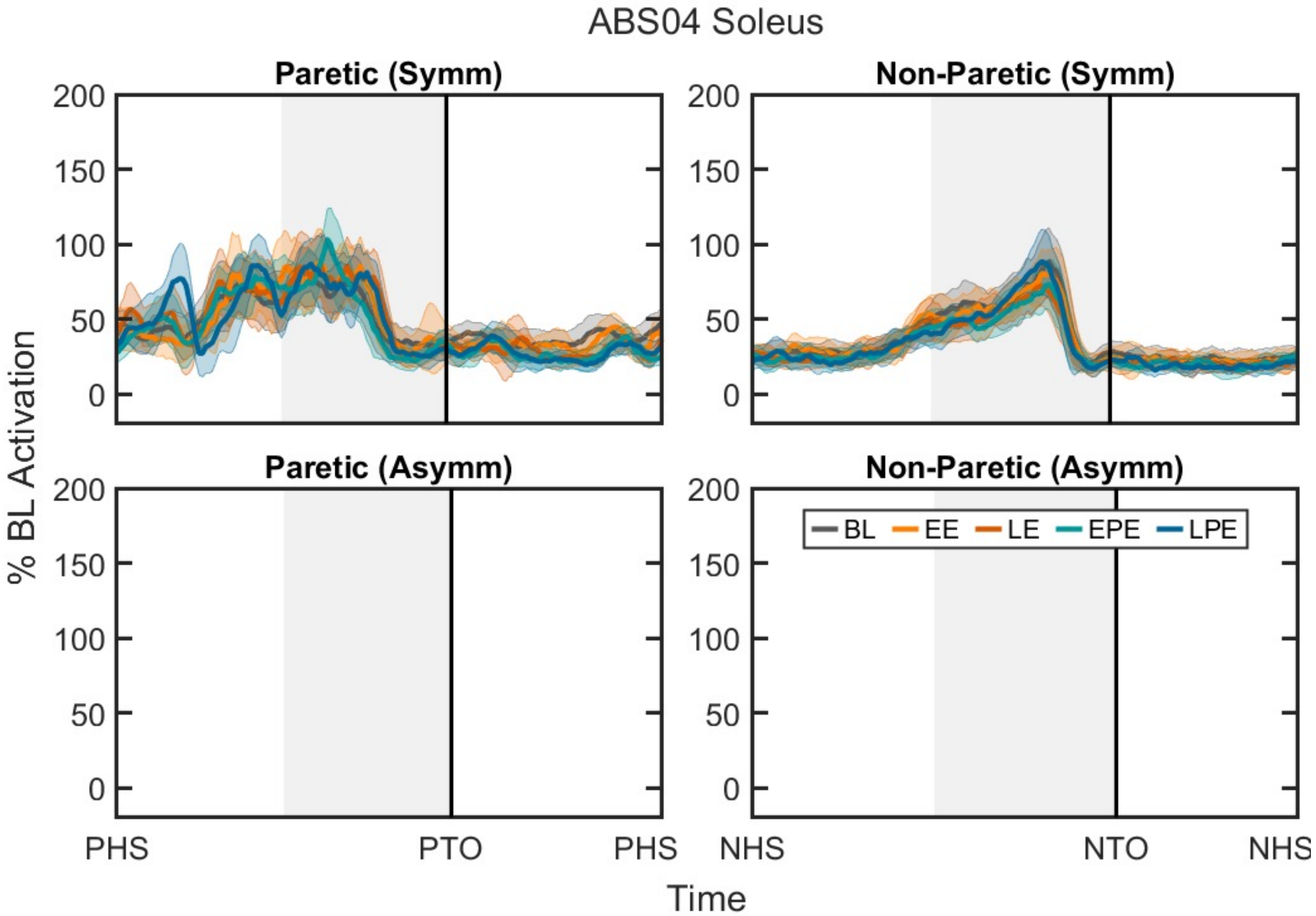
ABS04 Soleus
Paretic (Symm)
Non-Paretic (Symm)
Paretic (Asymm)
Non-Paretic (Asymm)
% BL Activation
200
150
100
50
0
BL
EE
LE
EPE
LPE
PHS
PTO
PHS
NHS
NTO
NHS
Time

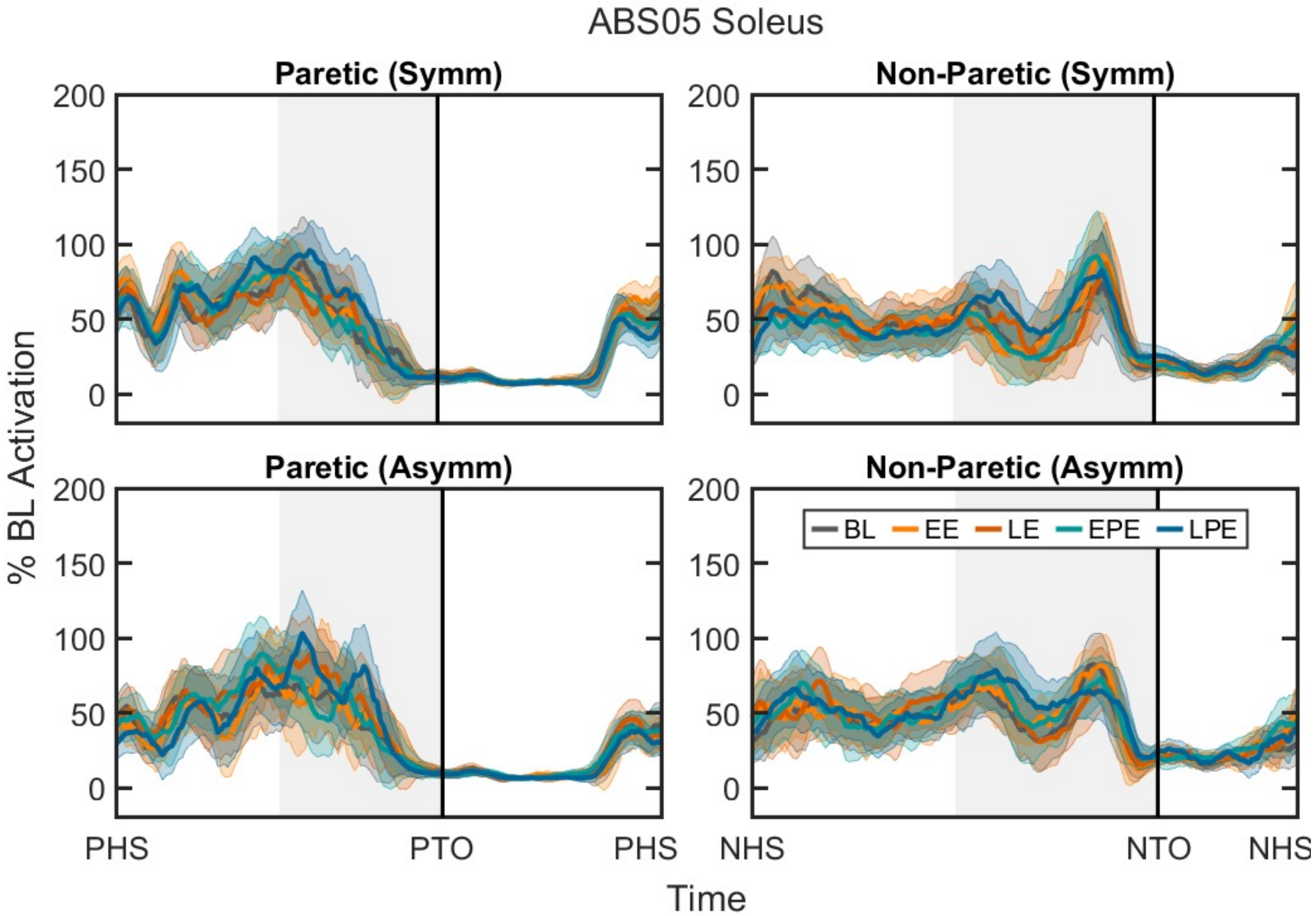

ABS05 Soleus
Paretic (Symm)
Non-Paretic (Symm)
Paretic (Asymm)
Non-Paretic (Asymm)
% BL Activation
200
150
100
50
0
BL
EE
LE
EPE
LPE
PHS
PTO
PHS
NHS
NTO
NHS
Time

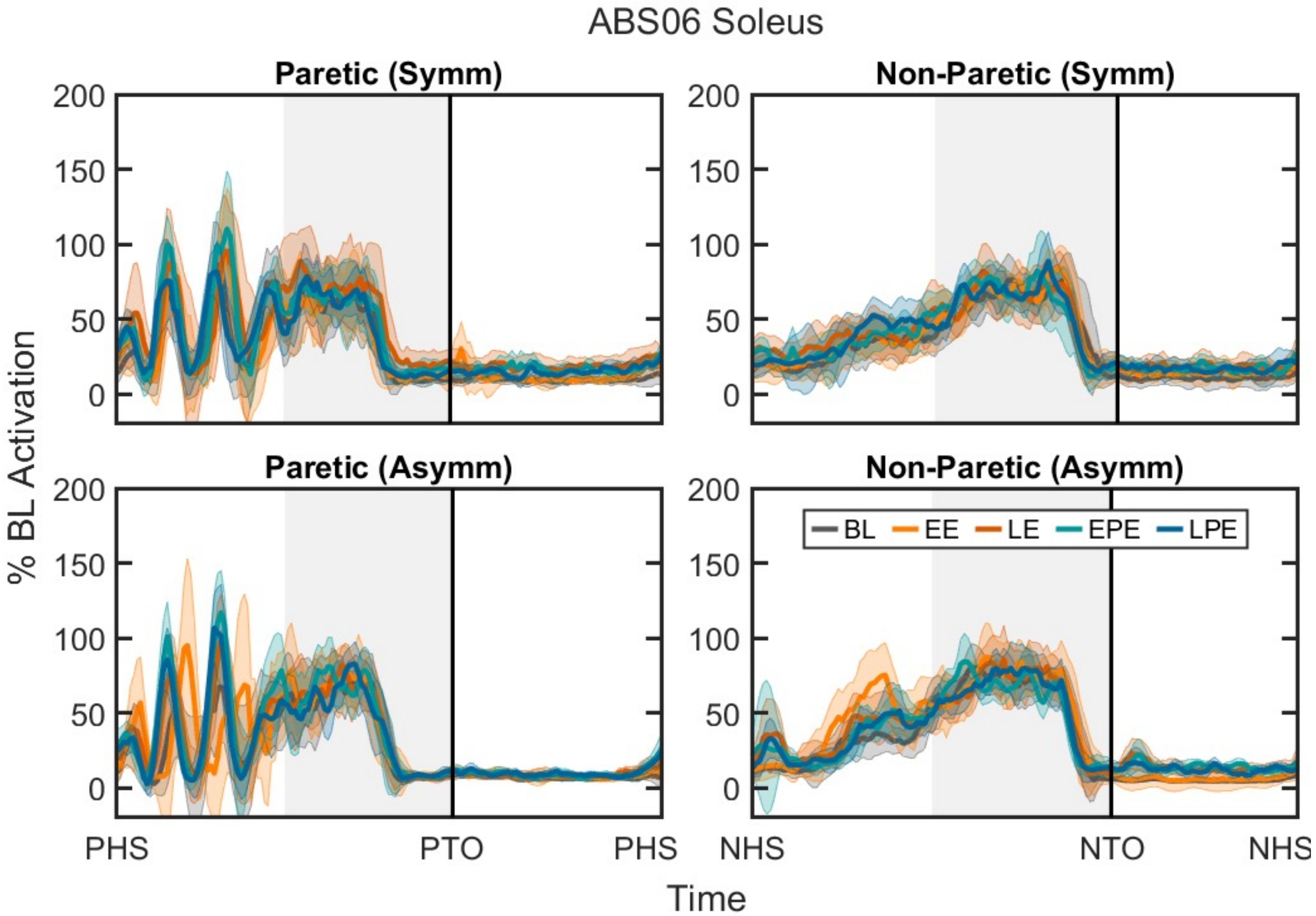
ABS06 Soleus
Paretic (Symm)
Non-Paretic (Symm)
Paretic (Asymm)
Non-Paretic (Asymm)
% BL Activation
200
150
100
50
0
BL
EE
LE
EPE
LPE
PHS
PTO
PHS
NHS
NTO
NHS
Time

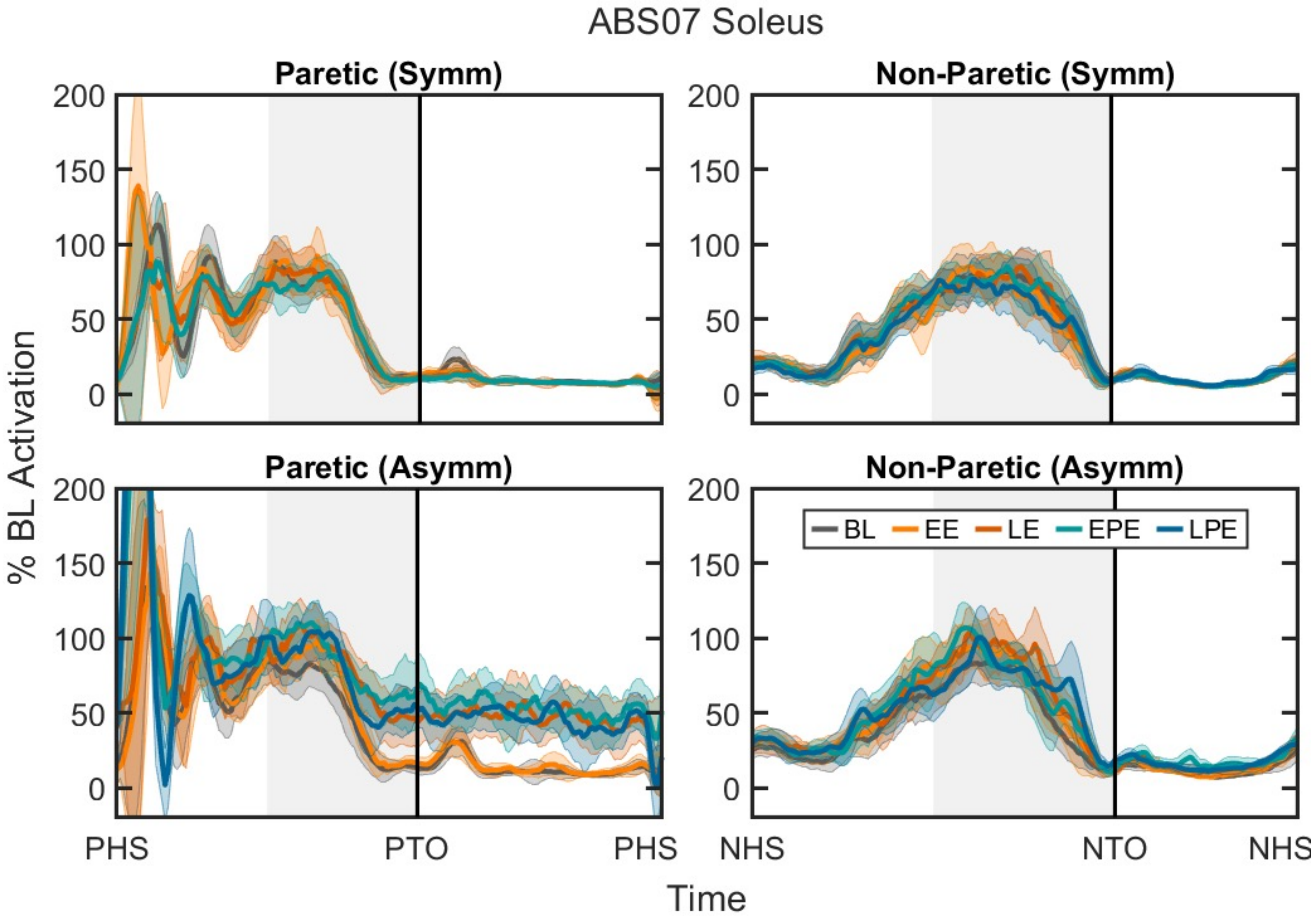
ABS07 Soleus
Paretic (Symm)
Non-Paretic (Symm)
Paretic (Asymm)
Non-Paretic (Asymm)
% BL Activation
200
150
100
50
0
BL
EE
LE
EPE
LPE
PHS
PTO
PHS
NHS
NTO
NHS
Time

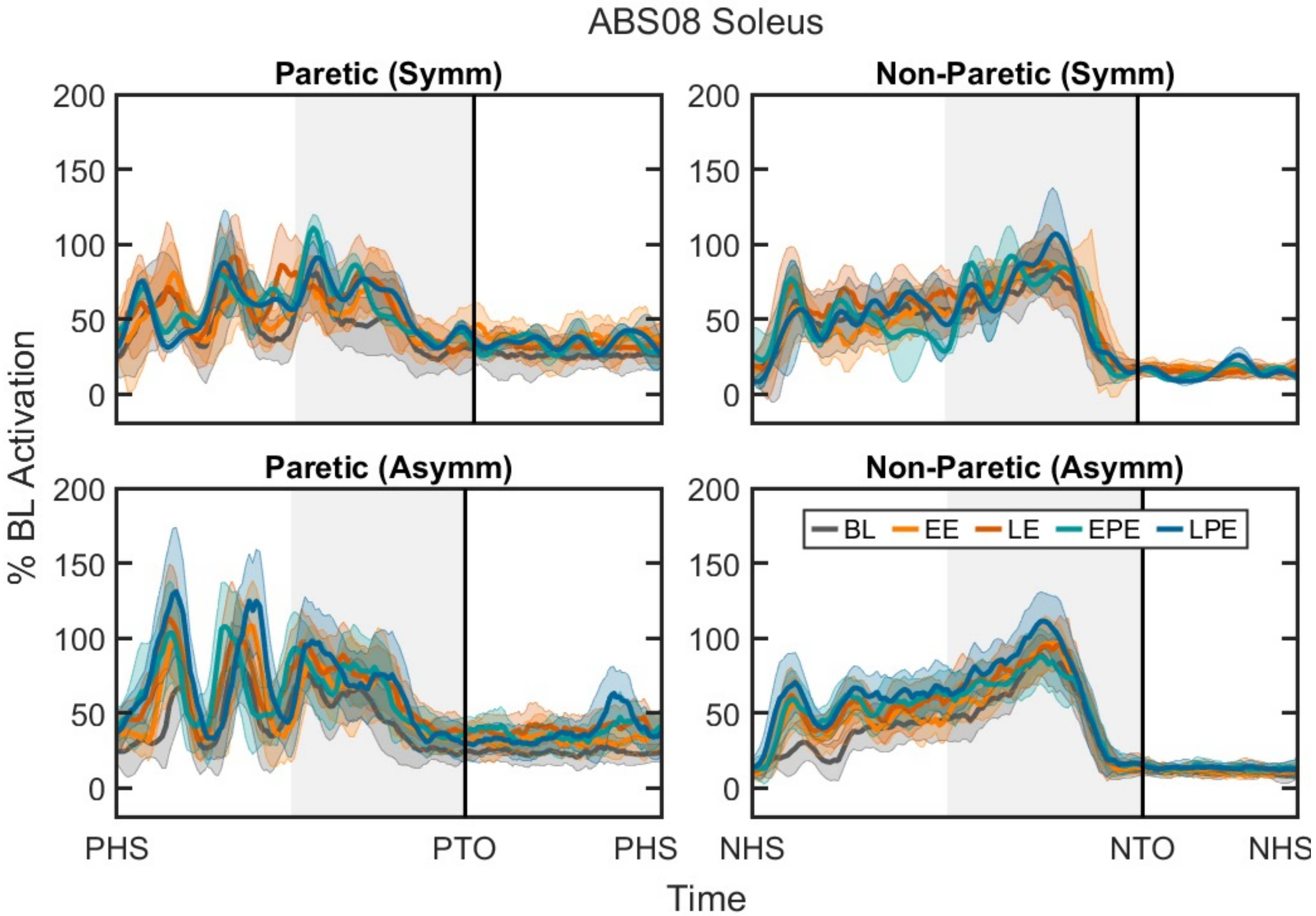
ABS08 Soleus
Paretic (Symm)
Non-Paretic (Symm)
Paretic (Asymm)
Non-Paretic (Asymm)
% BL Activation
200
150
100
50
0
BL
EE
LE
EPE
LPE
PHS
PTO
PHS
NHS
NTO
NHS
Time

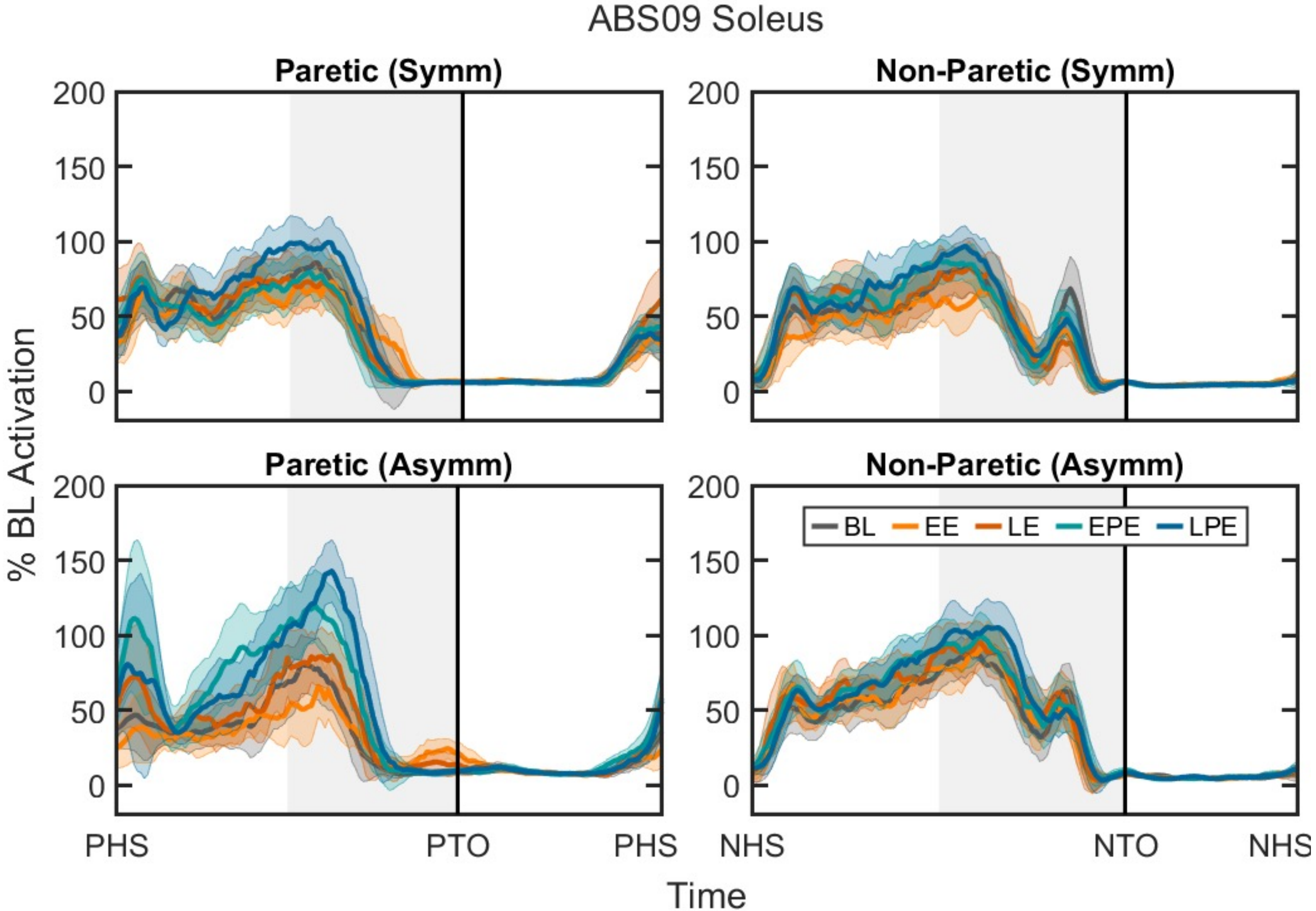
ABS09 Soleus
Paretic (Symm)
Non-Paretic (Symm)
Paretic (Asymm)
Non-Paretic (Asymm)
% BL Activation
200
150
100
50
0
BL
EE
LE
EPE
LPE
PHS
PTO
PHS
NHS
NTO
NHS
Time

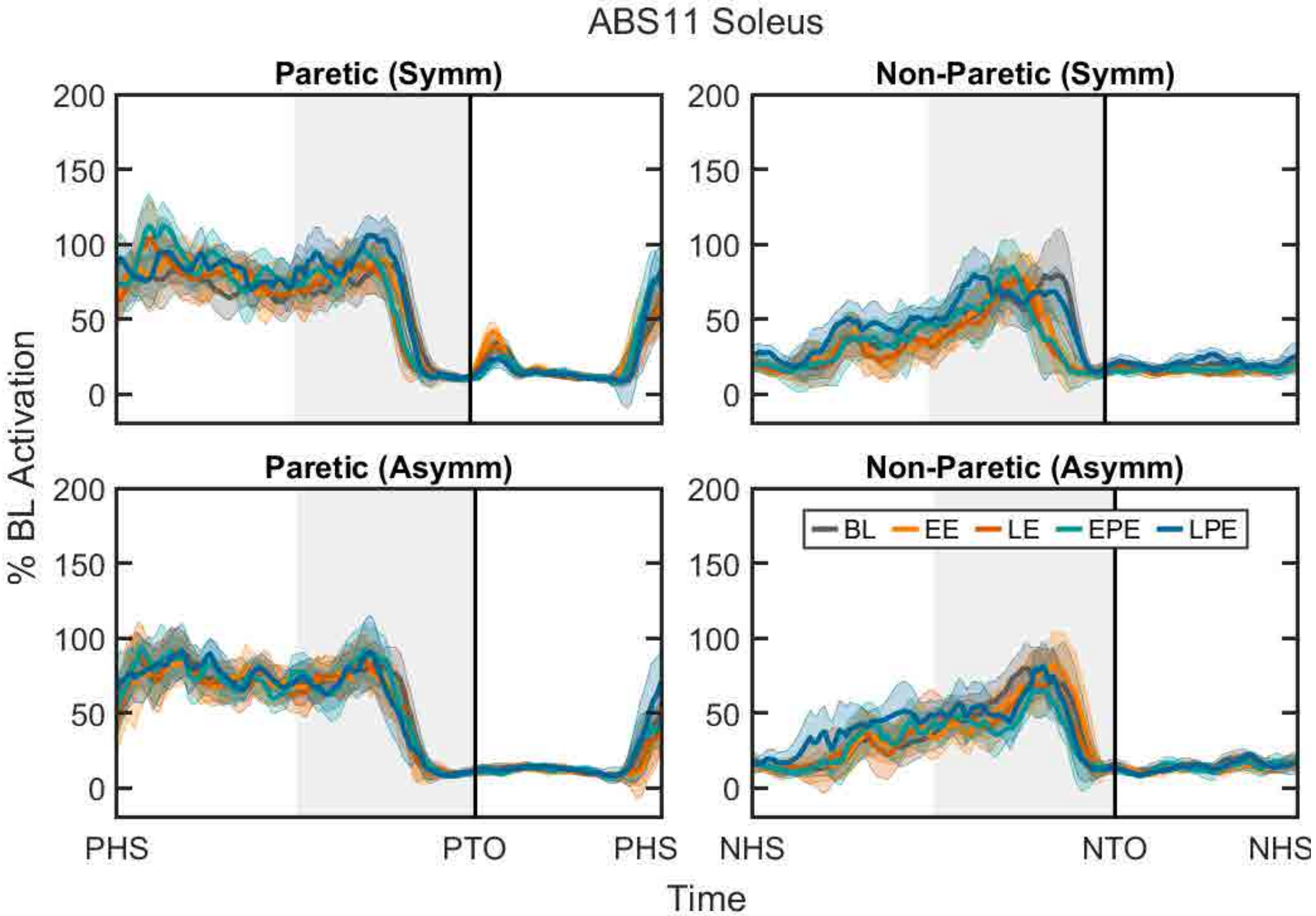
ABS11 Soleus
Paretic (Symm)
Non-Paretic (Symm)
Paretic (Asymm)
Non-Paretic (Asymm)
% BL Activation
200
150
100
50
0
BL
EE
LE
EPE
LPE
PHS
PTO
PHS
NHS
NTO
NHS
Time

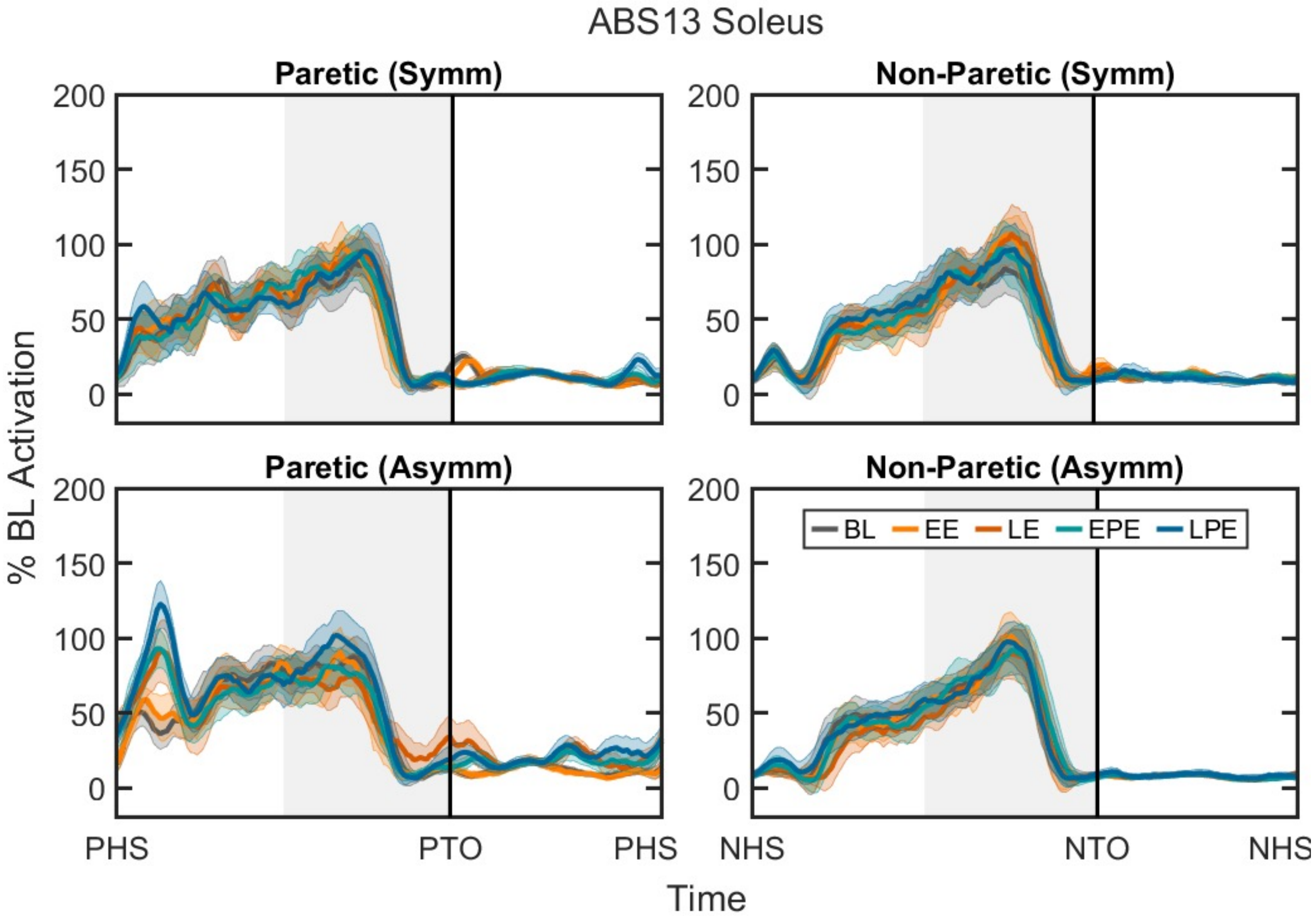

ABS13 Soleus
Paretic (Symm)
Non-Paretic (Symm)
Paretic (Asymm)
Non-Paretic (Asymm)
% BL Activation
200
150
100
50
0
BL
EE
LE
EPE
LPE
PHS
PTO
PHS
NHS
NTO
NHS
Time

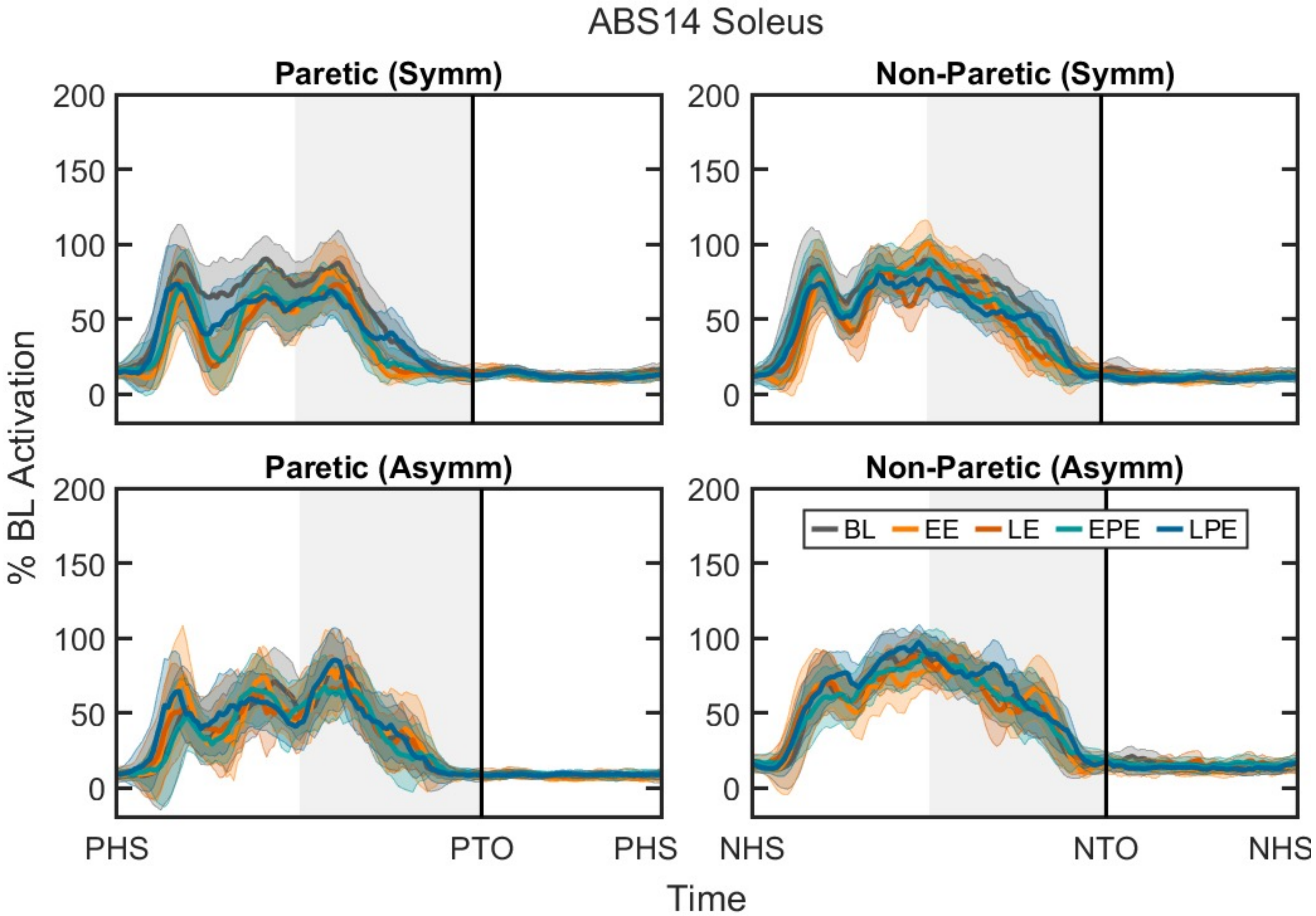

ABS14 Soleus
Paretic (Symm)
Non-Paretic (Symm)
Paretic (Asymm)
Non-Paretic (Asymm)
% BL Activation
200
150
100
50
0
BL
EE
LE
EPE
LPE
PHS
PTO
PHS
NHS
NTO
NHS
Time

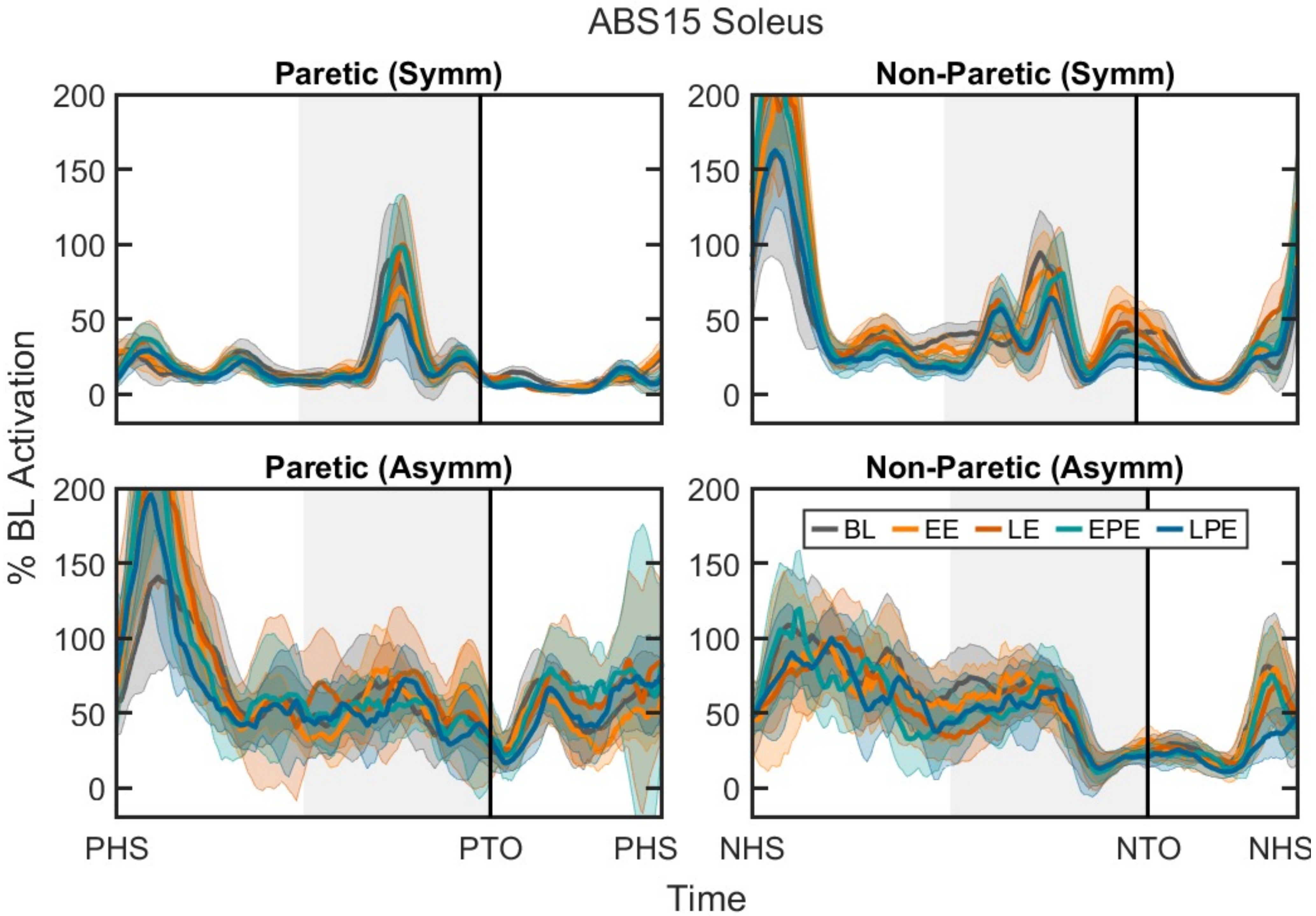
ABS15 Soleus
Paretic (Symm)
Non-Paretic (Symm)
Paretic (Asymm)
Non-Paretic (Asymm)
% BL Activation
200
150
100
50
0
BL
EE
LE
EPE
LPE
PHS
PTO
PHS
NHS
NTO
NHS
Time

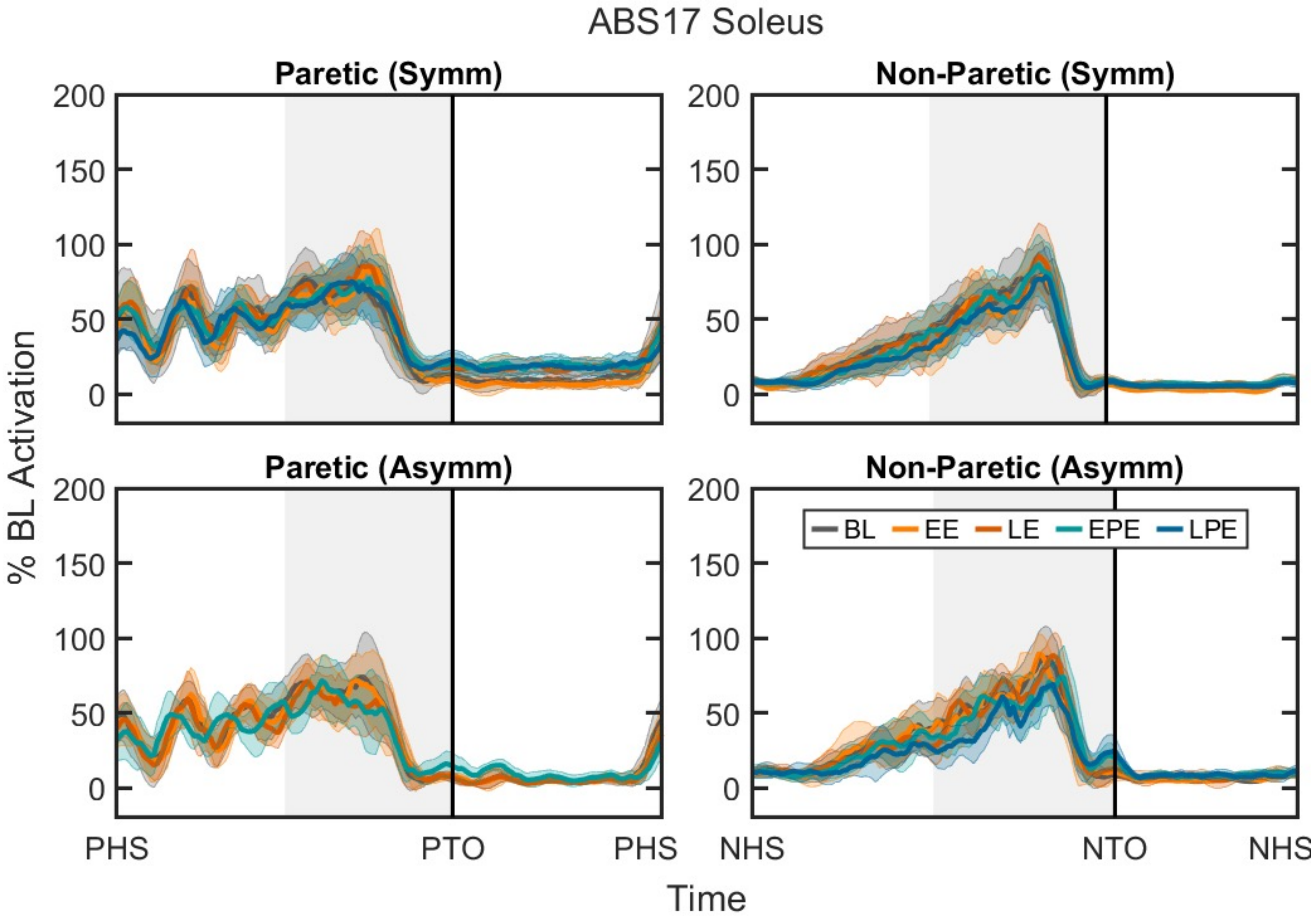
ABS17 Soleus
Paretic (Symm)
Non-Paretic (Symm)
Paretic (Asymm)
Non-Paretic (Asymm)
% BL Activation
200
150
100
50
0
BL
EE
LE
EPE
LPE
PHS
PTO
PHS
NHS
NTO
NHS
Time

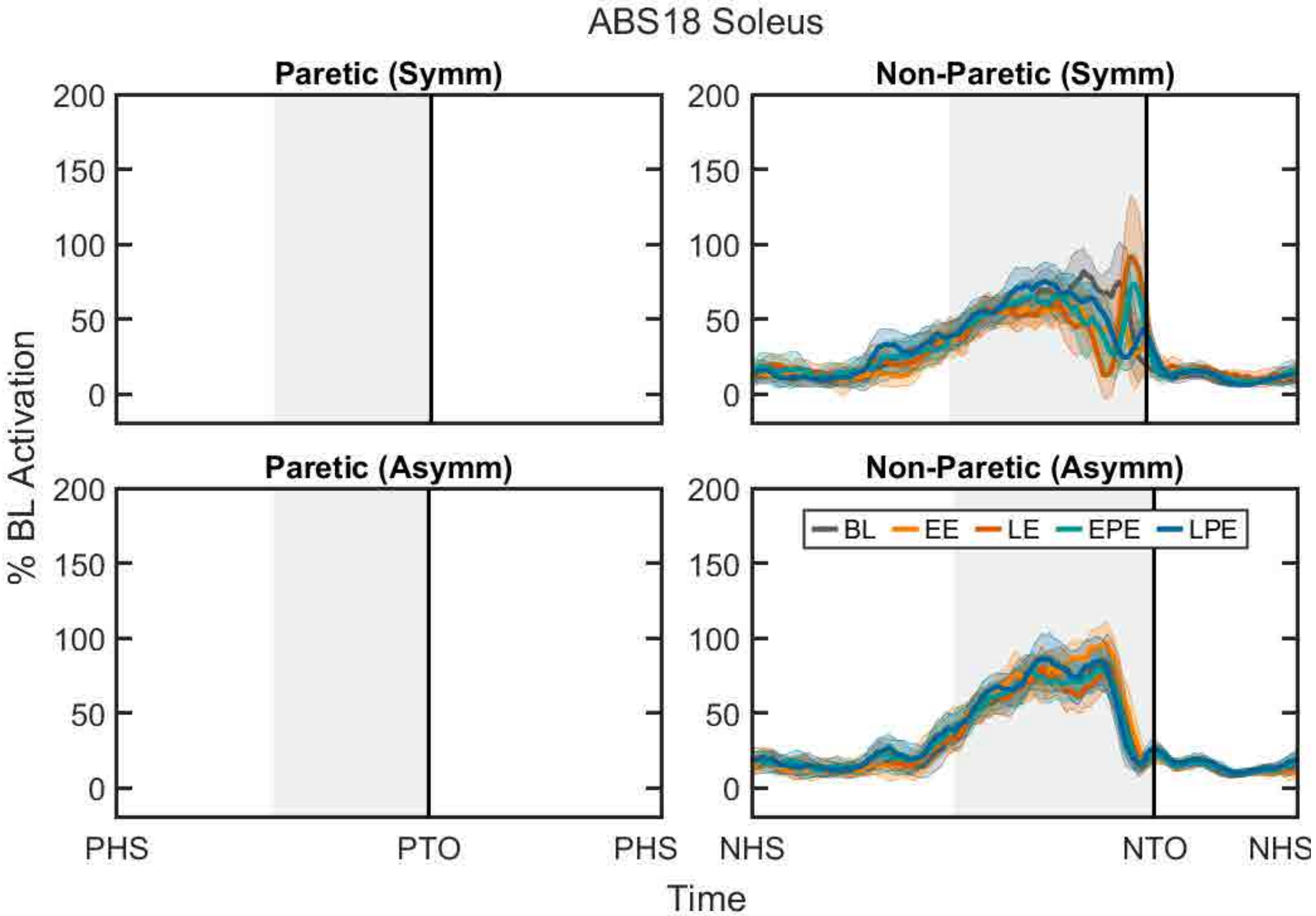

ABS18 Soleus
Paretic (Symm)
Non-Paretic (Symm)
Paretic (Asymm)
Non-Paretic (Asymm)
% BL Activation
200
150
100
50
0
BL
EE
LE
EPE
LPE
PHS
PTO
PHS
NHS
NTO
NHS
Time

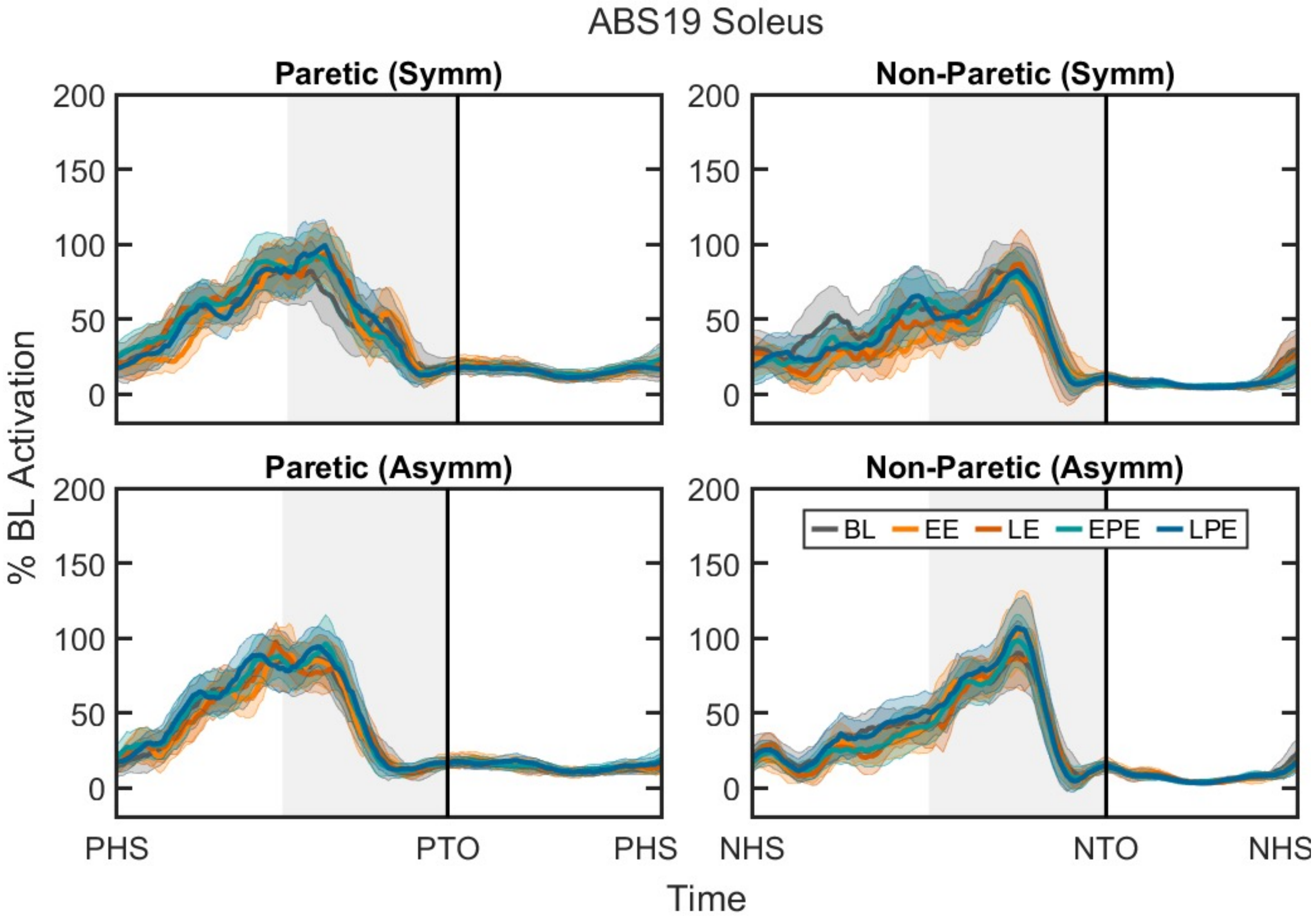
ABS19 Soleus
Paretic (Symm)
Non-Paretic (Symm)
Paretic (Asymm)
Non-Paretic (Asymm)
% BL Activation
200
150
100
50
0
BL
EE
LE
EPE
LPE
PHS
PTO
PHS
NHS
NTO
NHS
Time

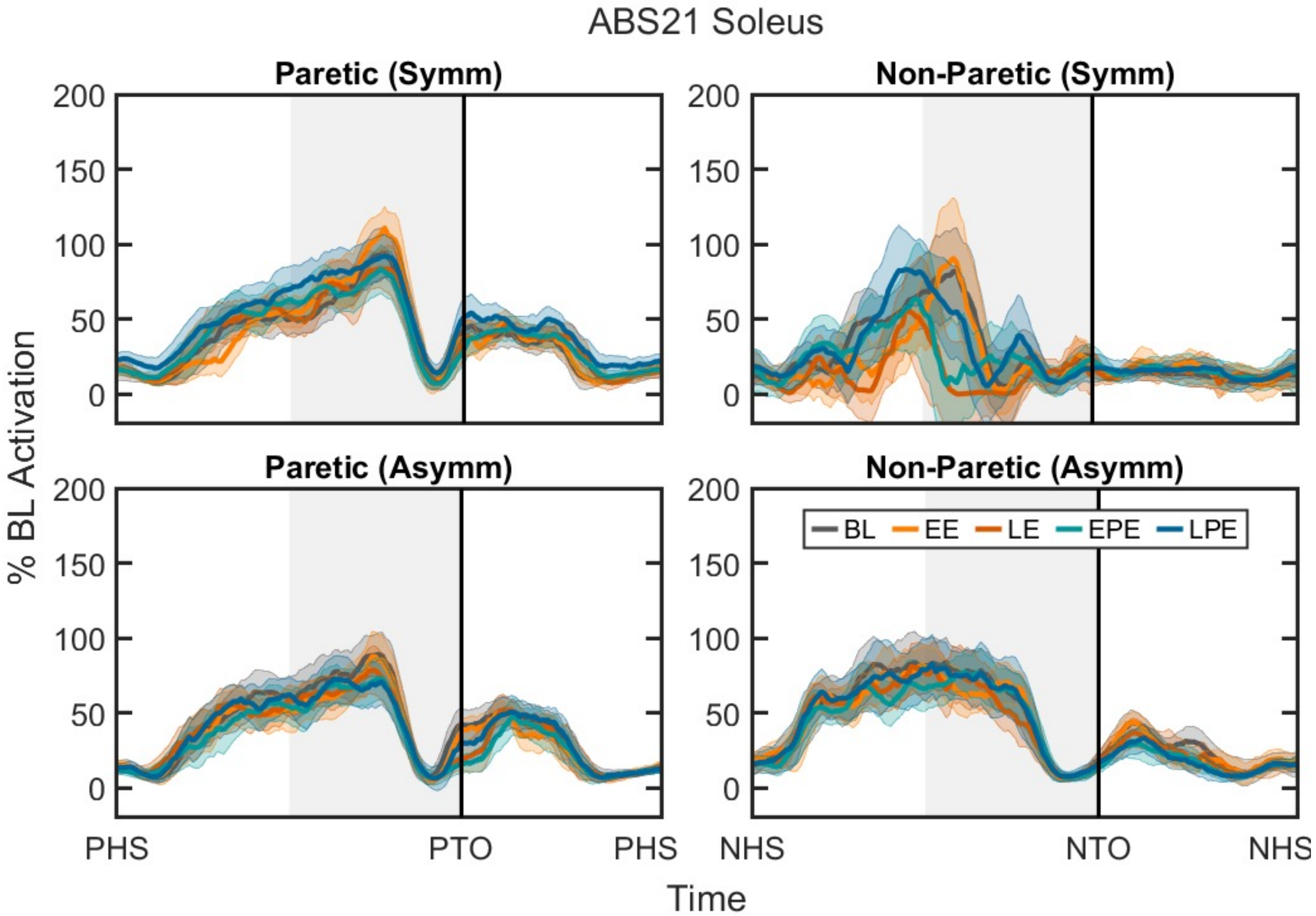

ABS21 Soleus
Paretic (Symm)
Non-Paretic (Symm)
Paretic (Asymm)
Non-Paretic (Asymm)
% BL Activation
200
150
100
50
0
BL
EE
LE
EPE
LPE
PHS
PTO
PHS
NHS
NTO
NHS
Time

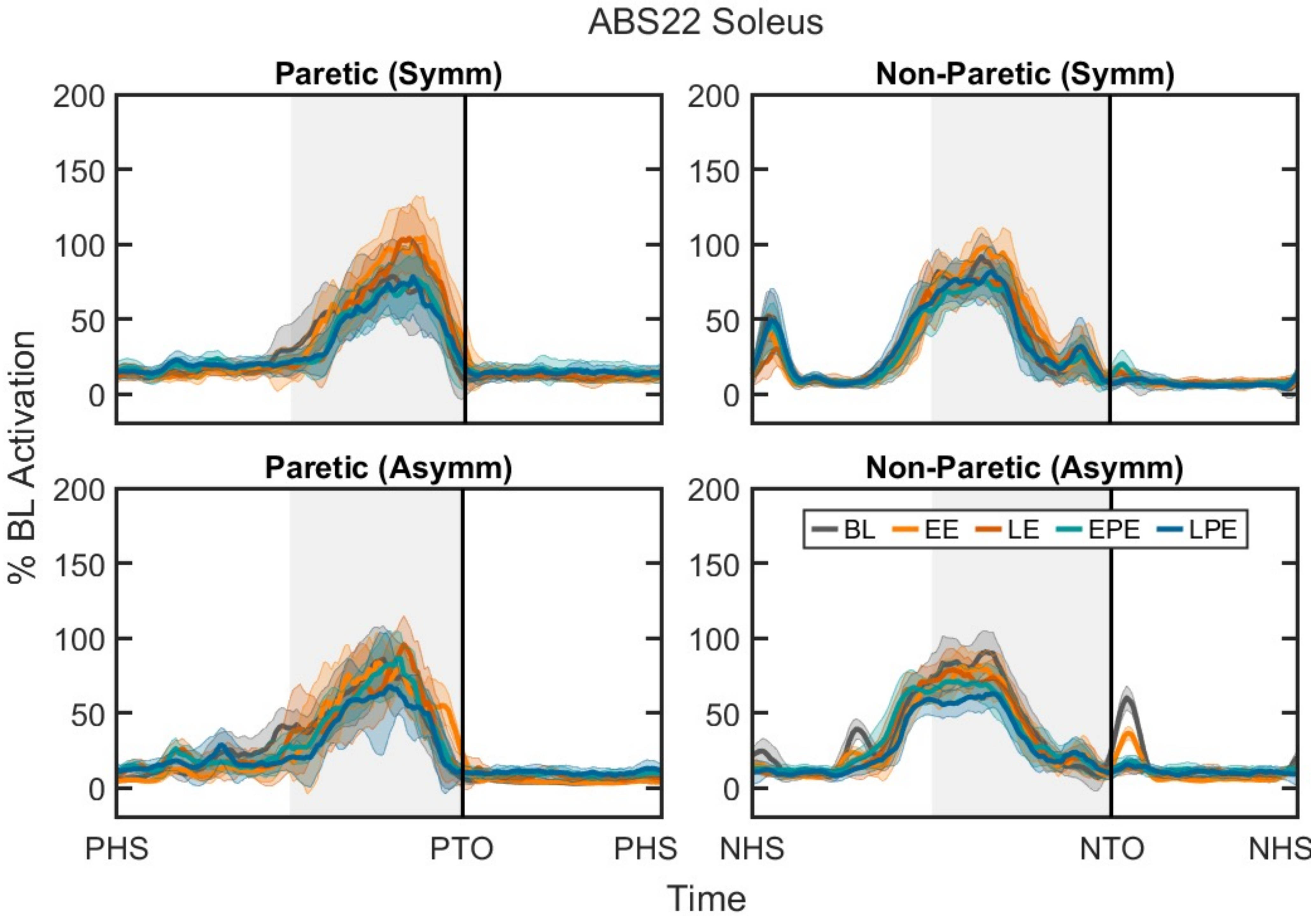
ABS22 Soleus
Paretic (Symm)
Non-Paretic (Symm)
Paretic (Asymm)
Non-Paretic (Asymm)
BL
EE
LE
EPE
LPE
200
150
100
50
0
% BL Activation
PHS
PTO
PHS
NHS
NTO
NHS
Time

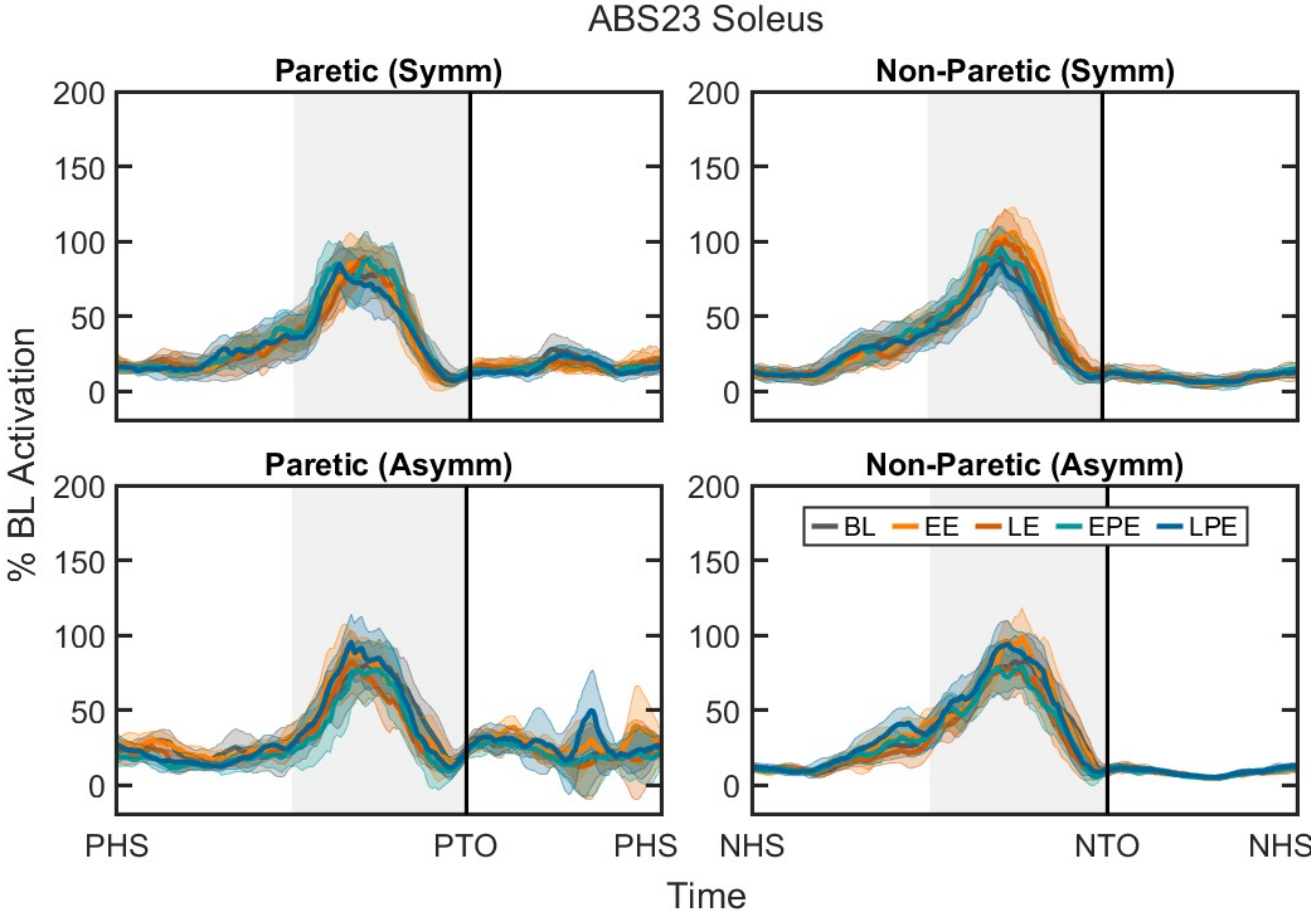
ABS23 Soleus
Paretic (Symm)
Non-Paretic (Symm)
Paretic (Asymm)
Non-Paretic (Asymm)
% BL Activation
200
150
100
50
0
BL
EE
LE
EPE
LPE
PHS
PTO
PHS
NHS
NTO
NHS
Time

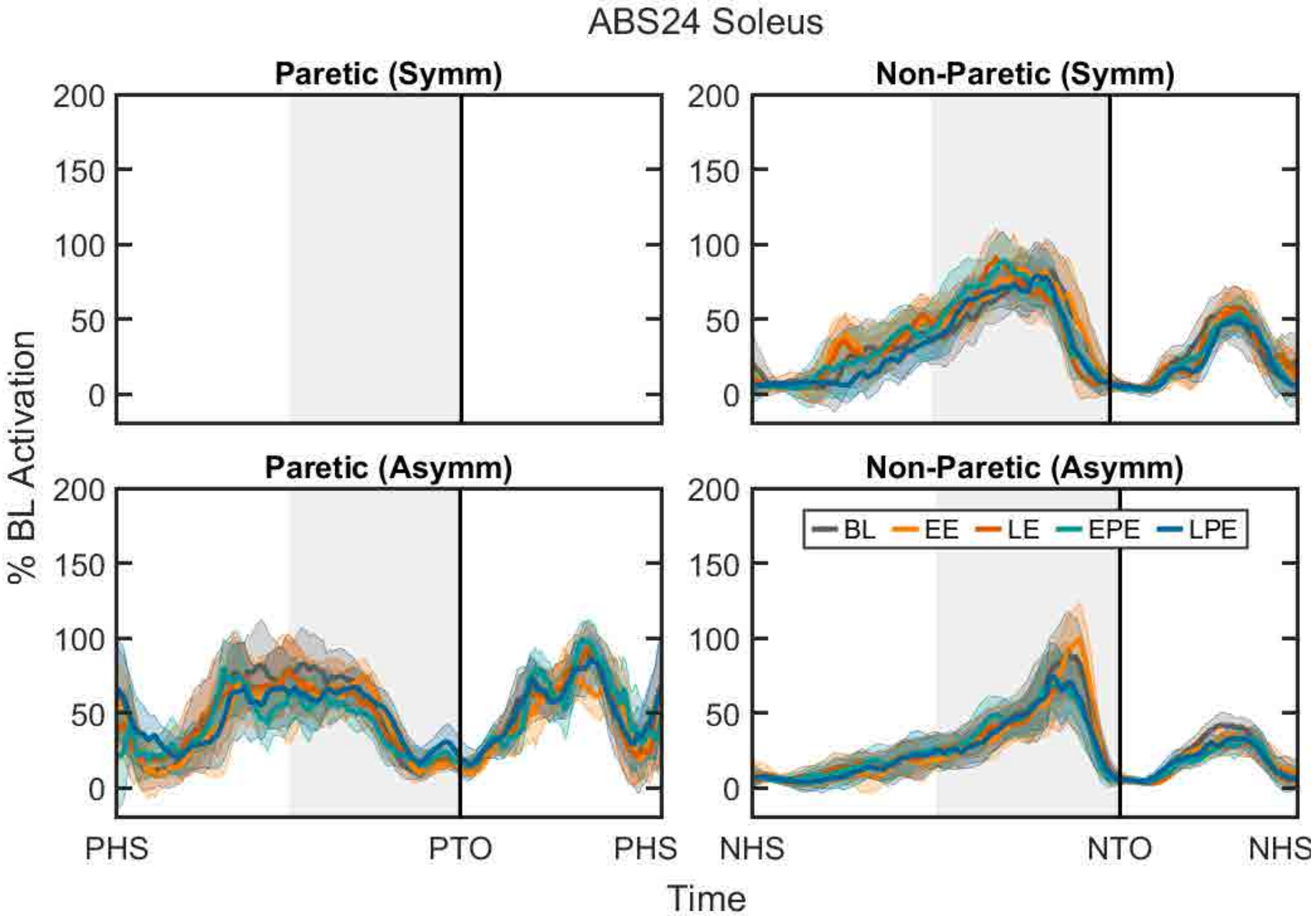
ABS24 Soleus
Paretic (Symm)
Non-Paretic (Symm)
Paretic (Asymm)
Non-Paretic (Asymm)
% BL Activation
200
150
100
50
0
BL
EE
LE
EPE
LPE
PHS
PTO
PHS
NHS
NTO
NHS
Time

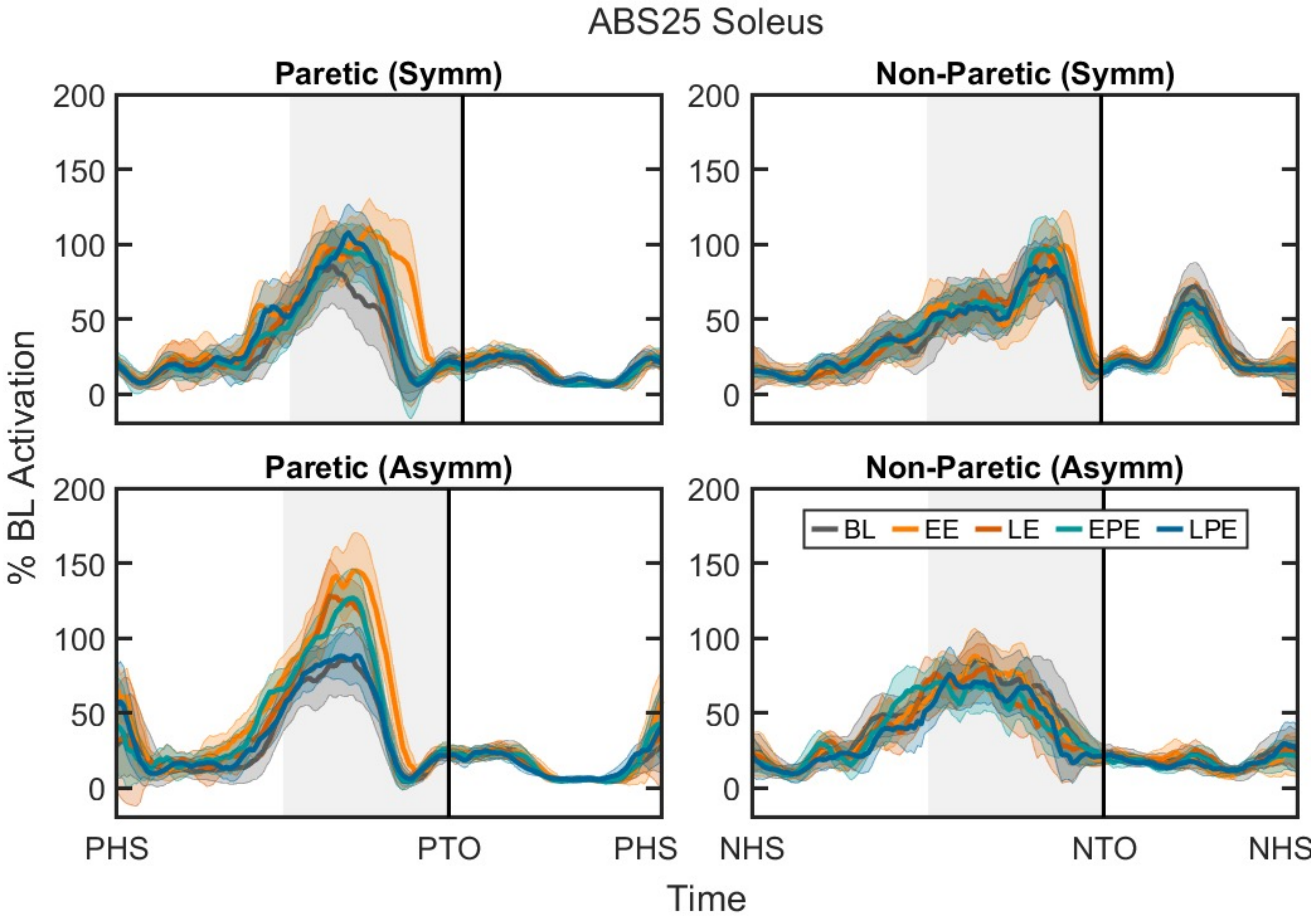
ABS25 Soleus
Paretic (Symm)
Non-Paretic (Symm)
Paretic (Asymm)
Non-Paretic (Asymm)
% BL Activation
200
150
100
50
0
BL
EE
LE
EPE
LPE
PHS
PTO
PHS
NHS
NTO
NHS
Time

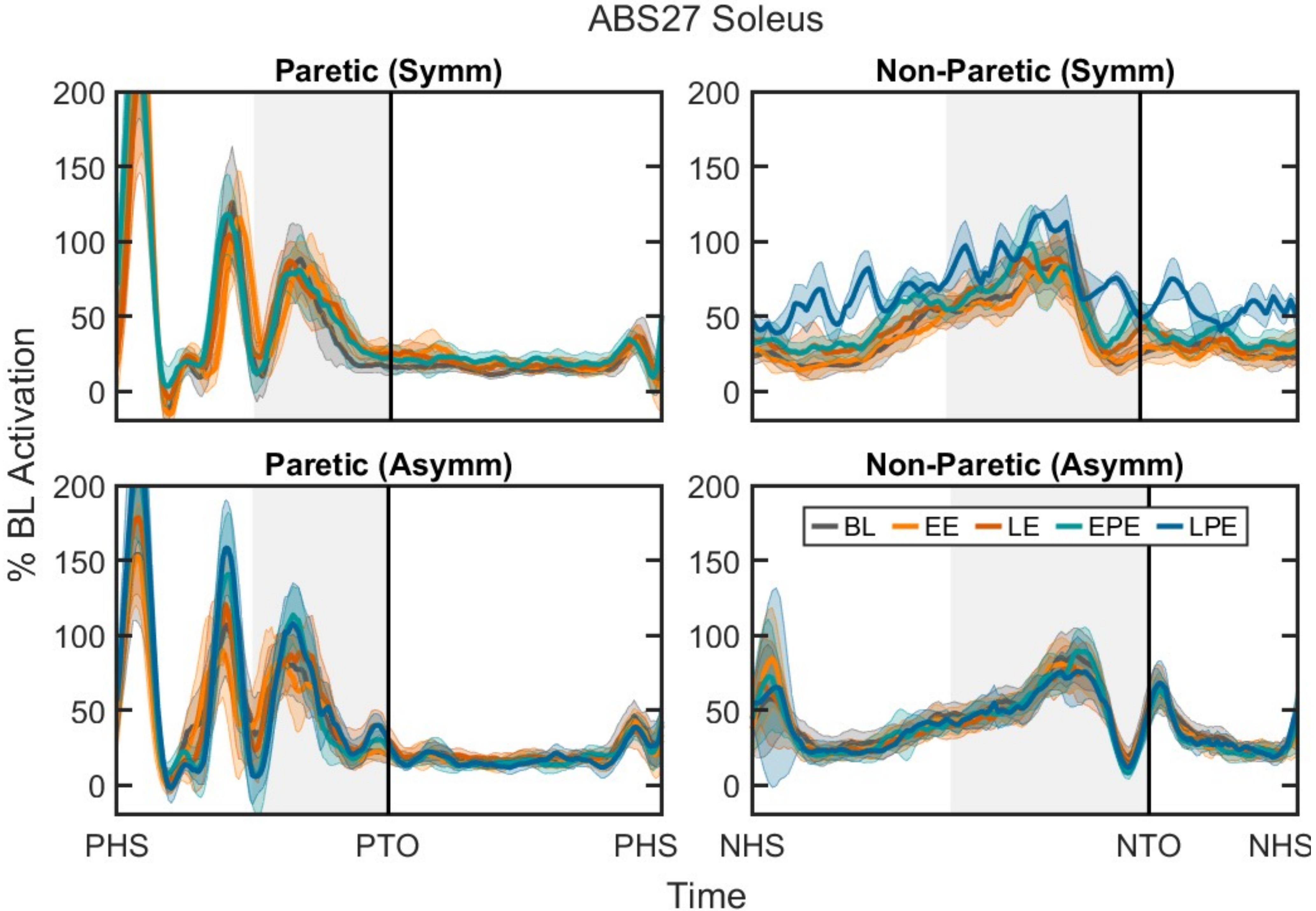
ABS27 Soleus
Paretic (Symm)
Non-Paretic (Symm)
Paretic (Asymm)
Non-Paretic (Asymm)
% BL Activation
200
150
100
50
0
BL
EE
LE
EPE
LPE
PHS
PTO
PHS
NHS
NTO
NHS
Time

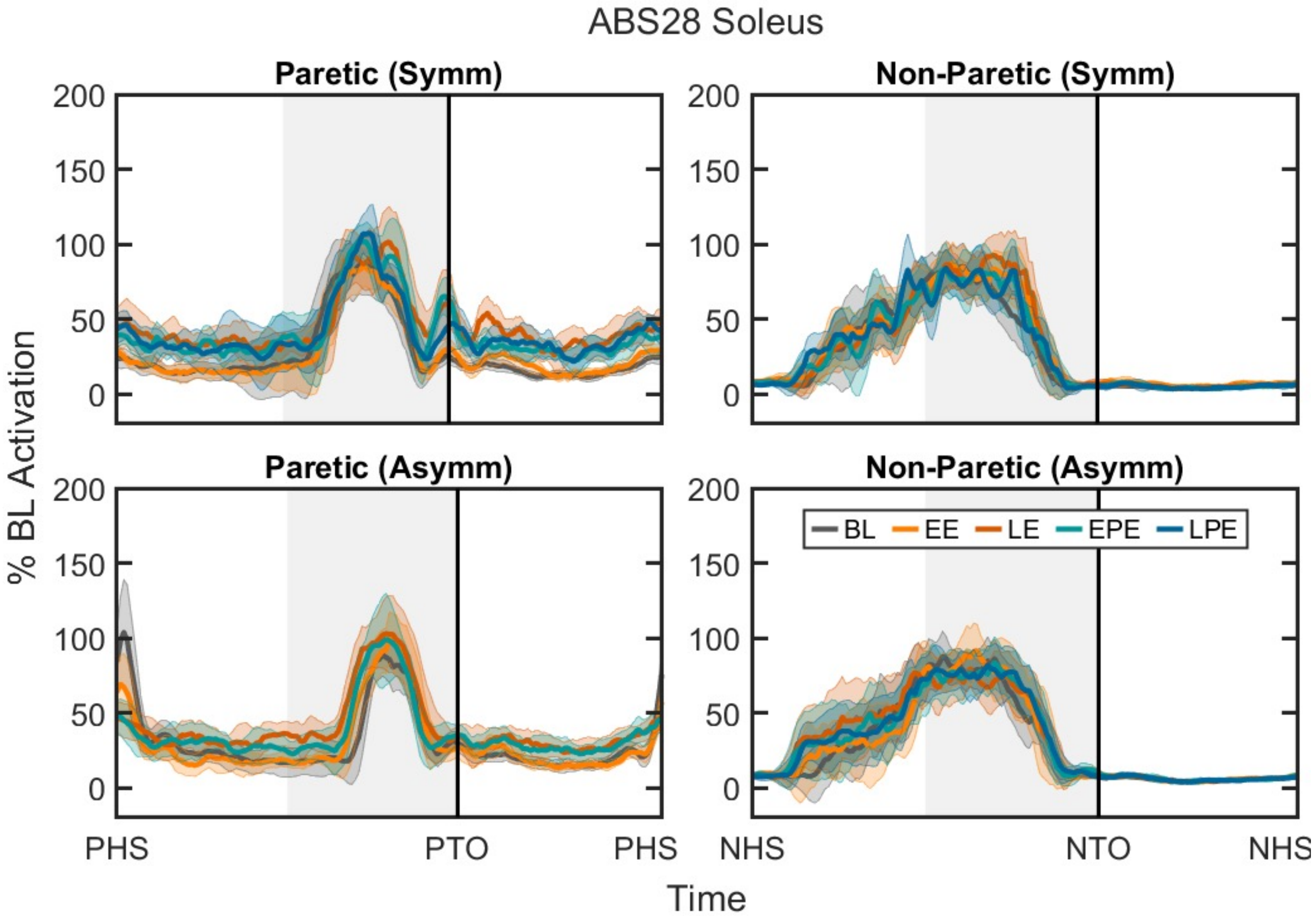
ABS28 Soleus
Paretic (Symm)
Non-Paretic (Symm)
Paretic (Asymm)
Non-Paretic (Asymm)
% BL Activation
200
150
100
50
0
BL
EE
LE
EPE
LPE
PHS
PTO
PHS
NHS
NTO
NHS
Time

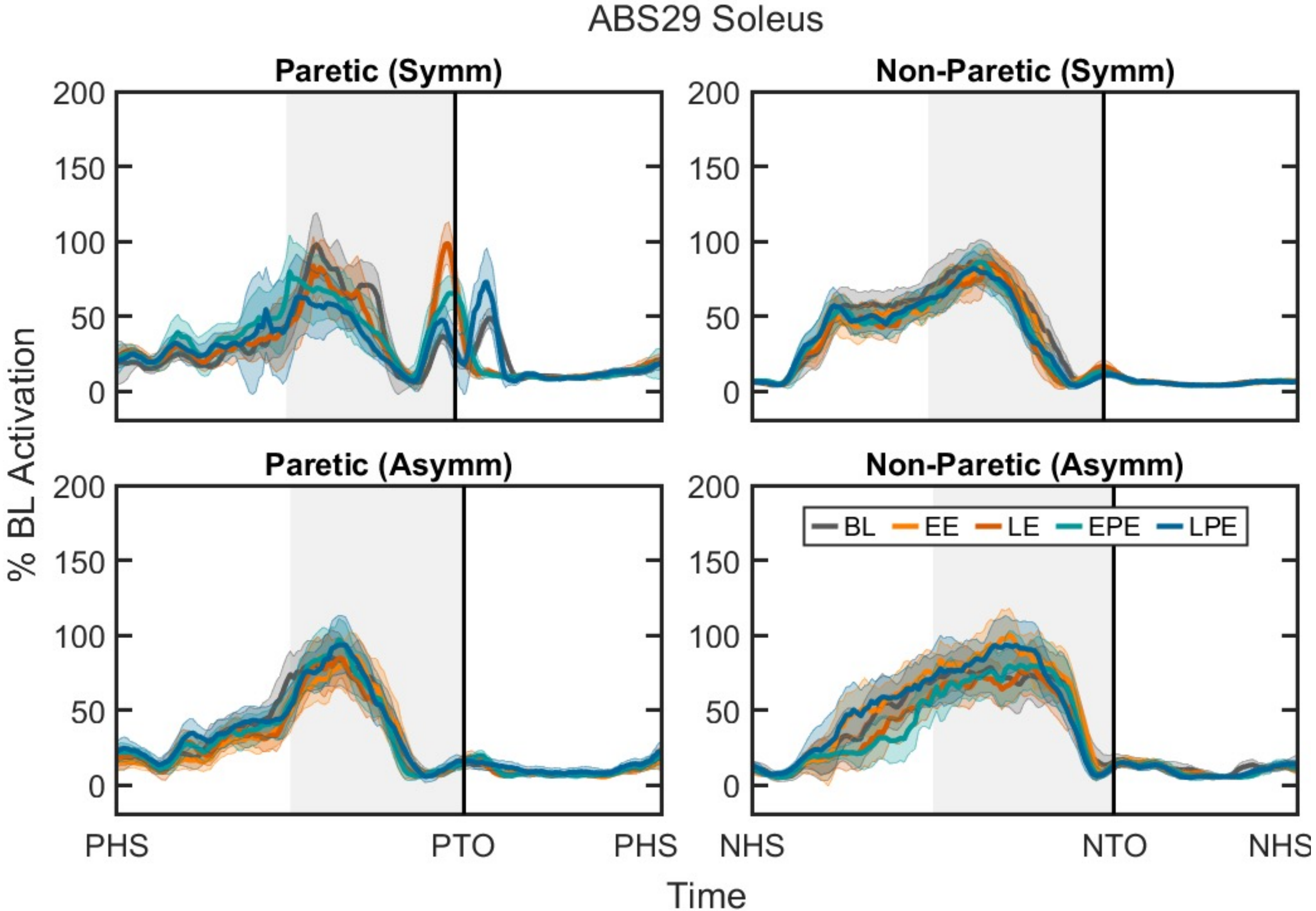
ABS29 Soleus
Paretic (Symm)
Non-Paretic (Symm)
Paretic (Asymm)
Non-Paretic (Asymm)
BL
EE
LE
EPE
LPE
% BL Activation
200
150
100
50
0
PHS
PTO
PHS
NHS
NTO
NHS
Time

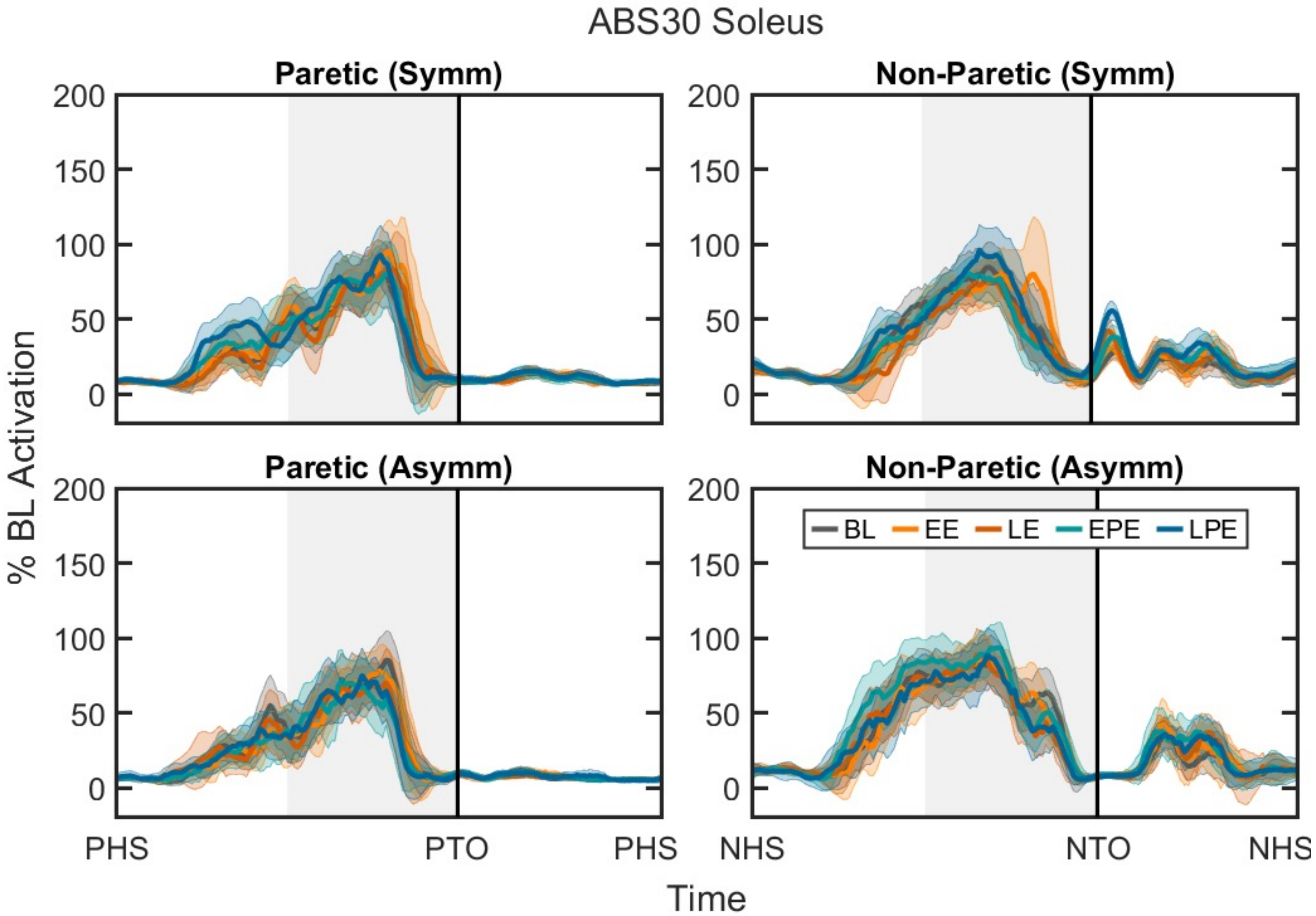
ABS30 Soleus
Paretic (Symm)
Non-Paretic (Symm)
Paretic (Asymm)
Non-Paretic (Asymm)
% BL Activation
200
150
100
50
0
BL
EE
LE
EPE
LPE
PHS
PTO
PHS
NHS
NTO
NHS
Time

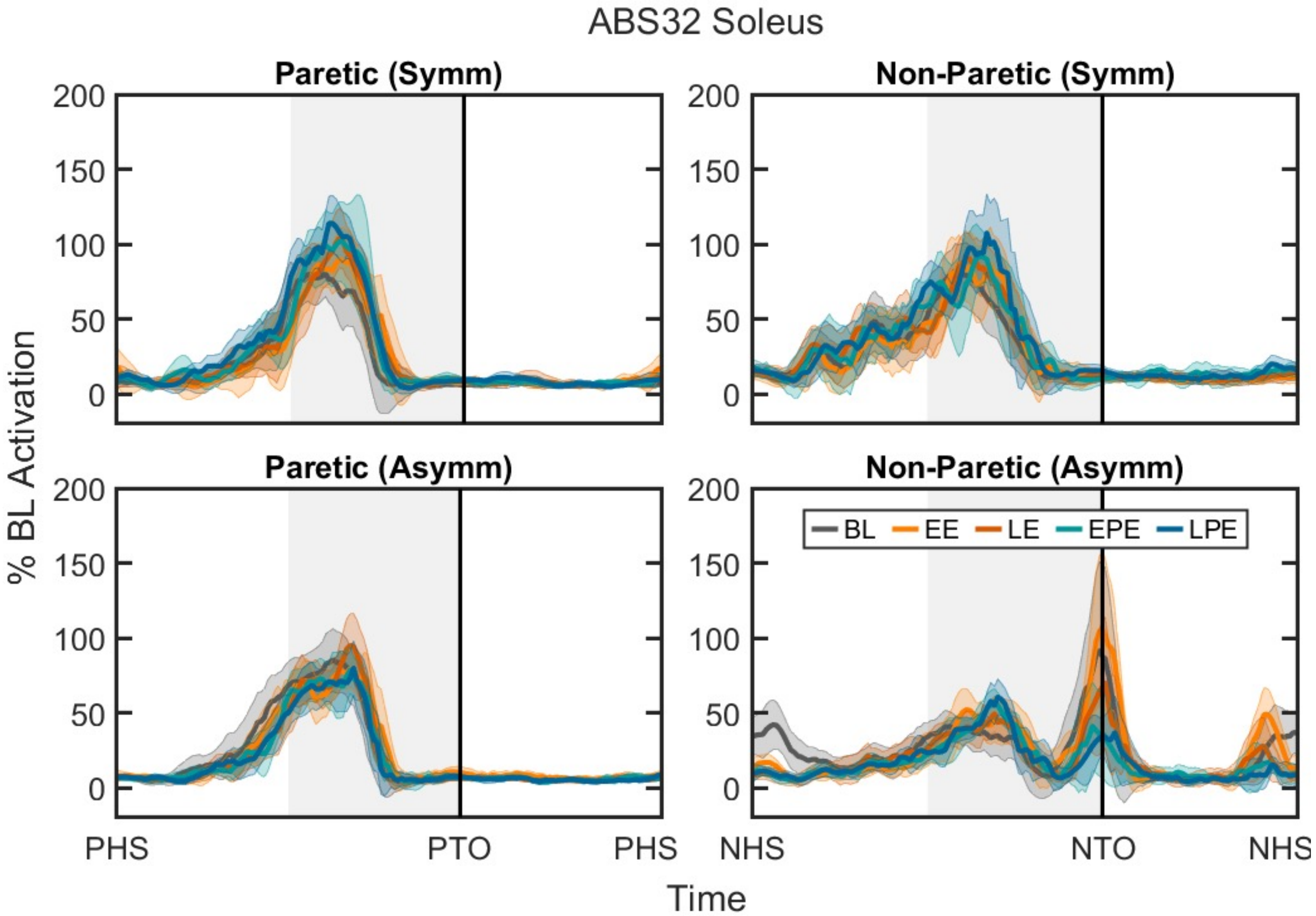
ABS32 Soleus
Paretic (Symm)
Non-Paretic (Symm)
Paretic (Asymm)
Non-Paretic (Asymm)
% BL Activation
200
150
100
50
0
BL
EE
LE
EPE
LPE
PHS
PTO
PHS
NHS
NTO
NHS
Time

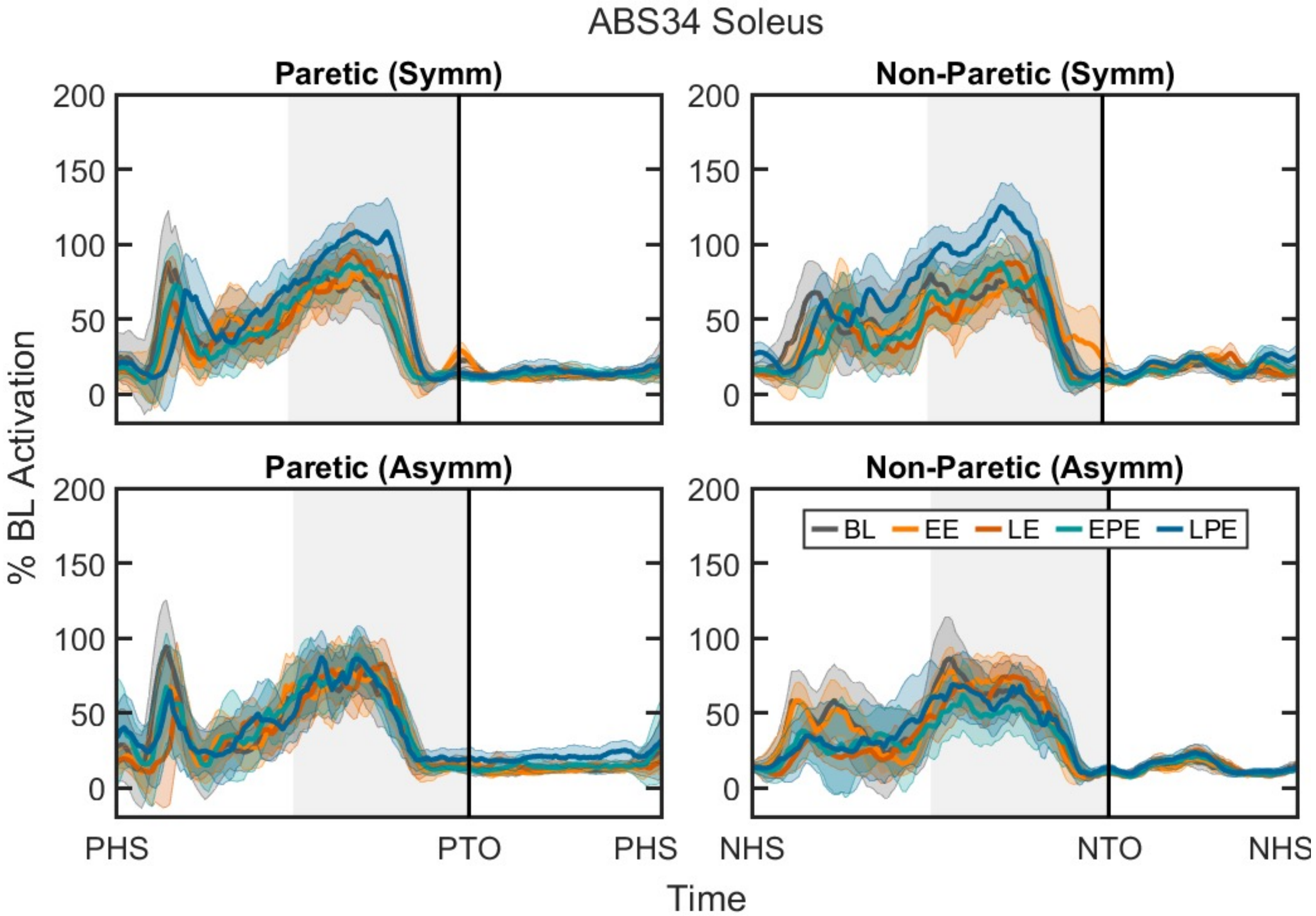
ABS34 Soleus
Paretic (Symm)
Non-Paretic (Symm)
Paretic (Asymm)
Non-Paretic (Asymm)
% BL Activation
200
150
100
50
0
BL
EE
LE
EPE
LPE
PHS
PTO
PHS
NHS
NTO
NHS
Time

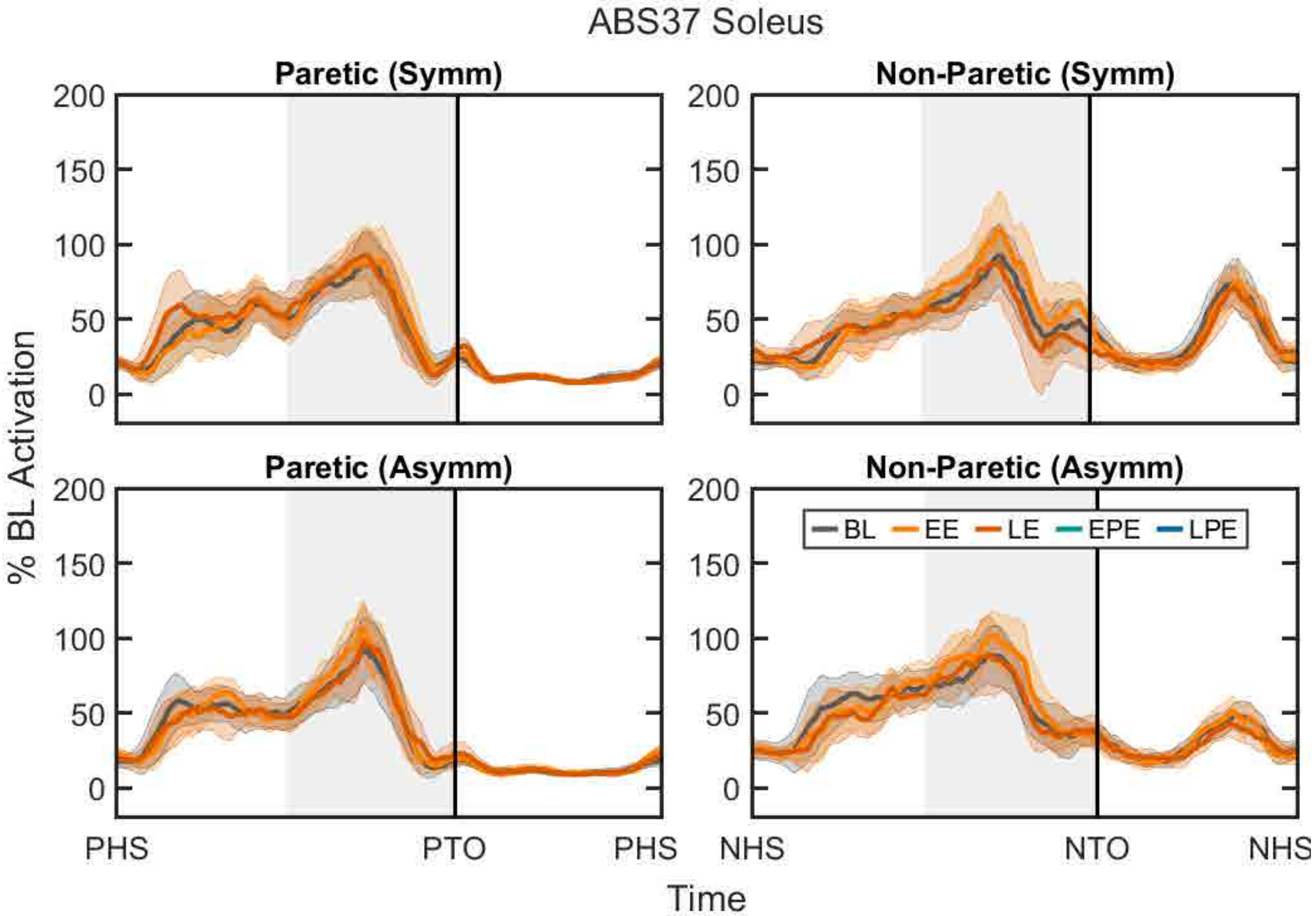

ABS37 Soleus
Paretic (Symm)
Non-Paretic (Symm)
Paretic (Asymm)
Non-Paretic (Asymm)
% BL Activation
200
150
100
50
0
BL
EE
LE
EPE
LPE
PHS
PTO
PHS
NHS
NTO
NHS
Time

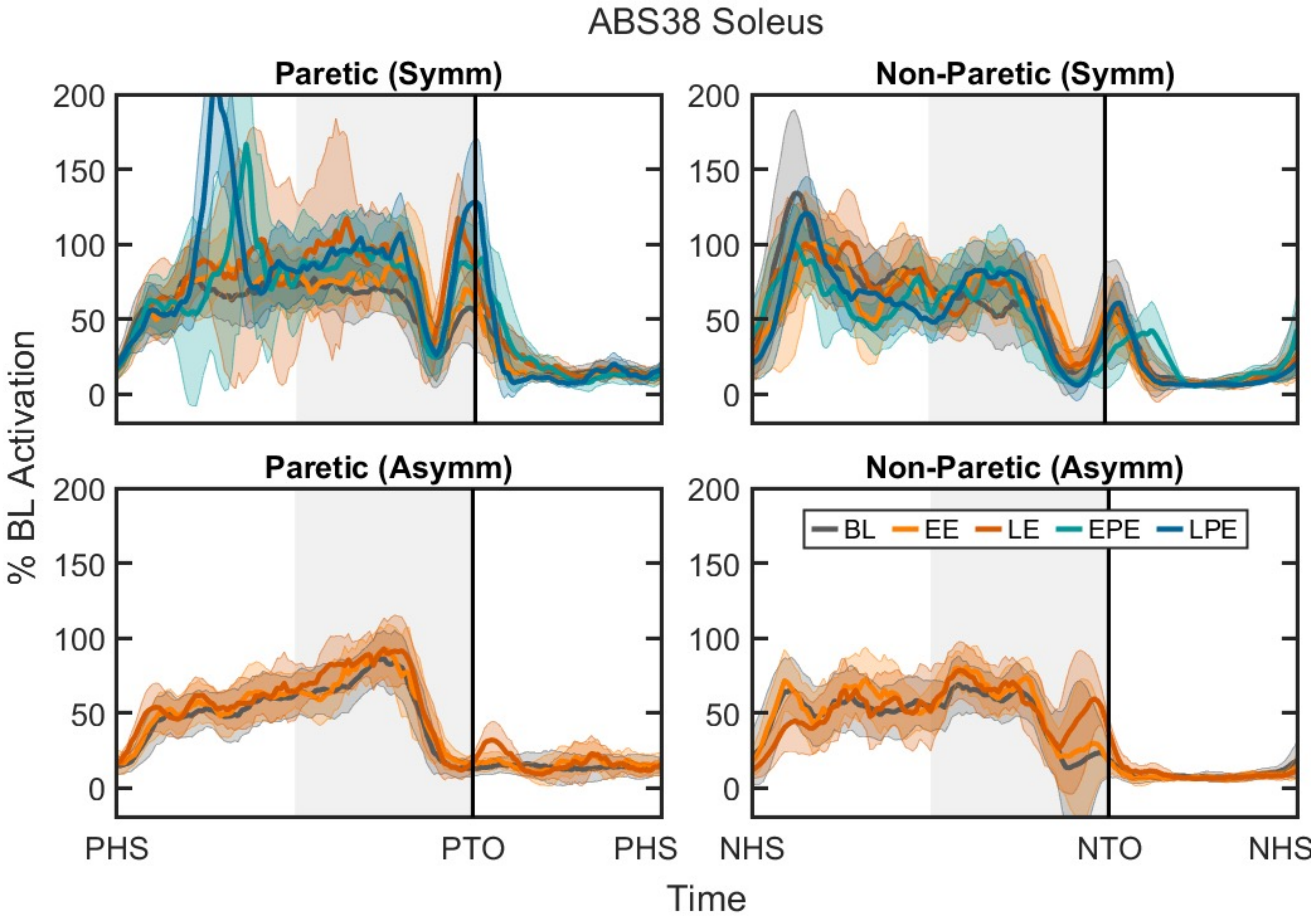
ABS38 Soleus
Paretic (Symm)
Non-Paretic (Symm)
Paretic (Asymm)
Non-Paretic (Asymm)
% BL Activation
200
150
100
50
0
BL
EE
LE
EPE
LPE
PHS
PTO
PHS
NHS
NTO
NHS
Time

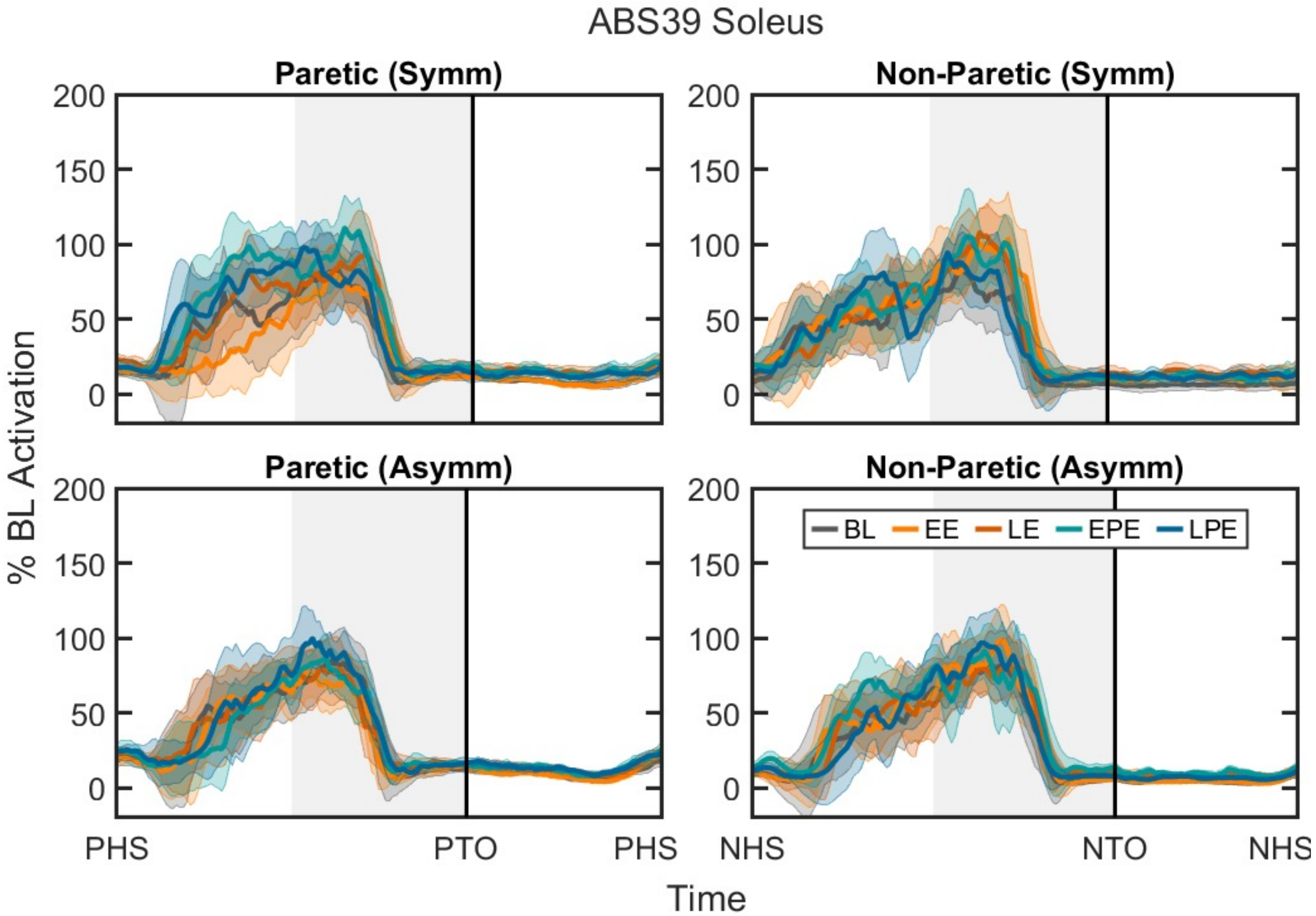
ABS39 Soleus
Paretic (Symm)
Non-Paretic (Symm)
Paretic (Asymm)
Non-Paretic (Asymm)
% BL Activation
200
150
100
50
0
BL
EE
LE
EPE
LPE
PHS
PTO
PHS
NHS
NTO
NHS
Time

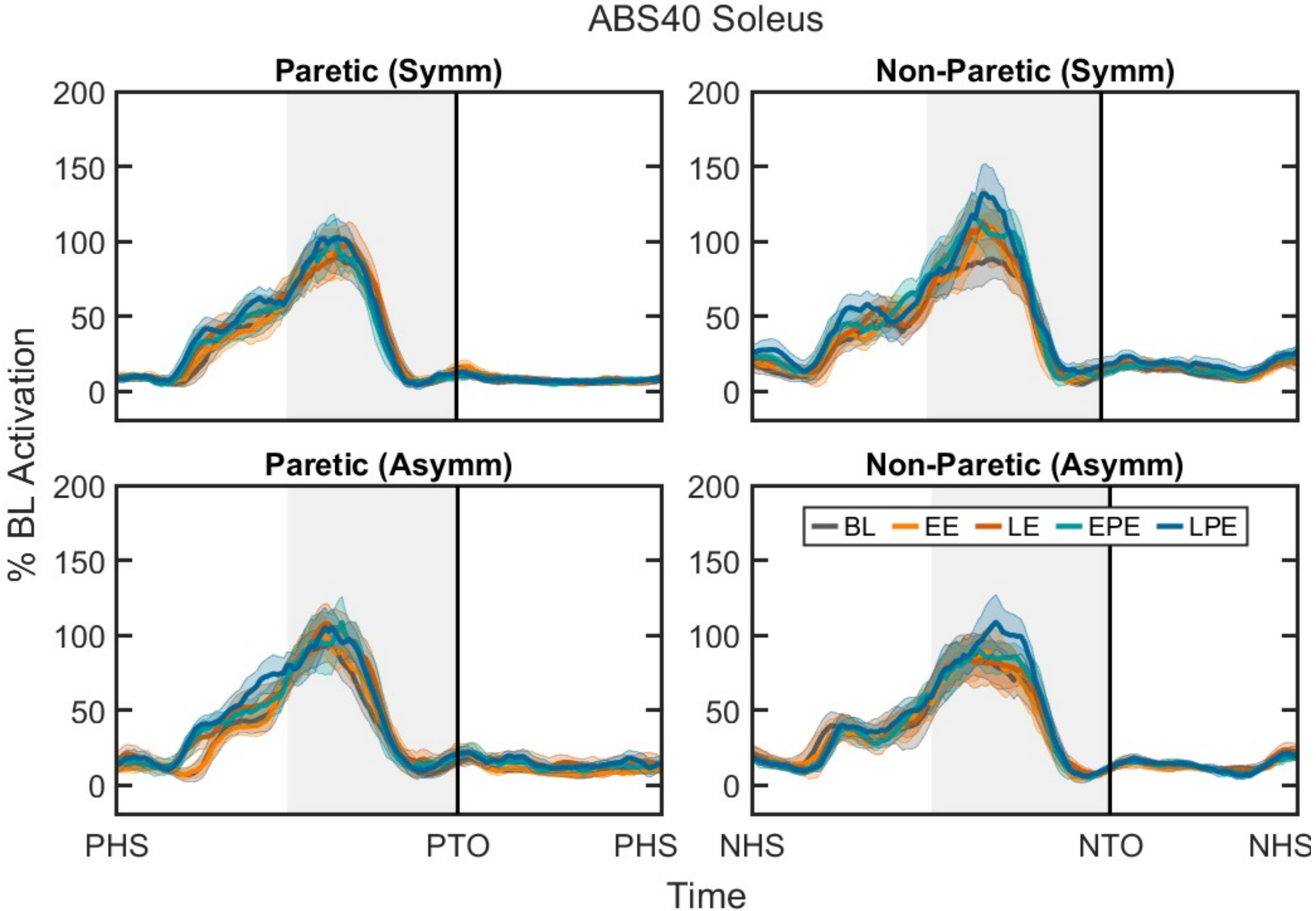

ABS40 Soleus
Paretic (Symm)
Non-Paretic (Symm)
Paretic (Asymm)
Non-Paretic (Asymm)
% BL Activation
200
150
100
50
0
BL
EE
LE
EPE
LPE
PHS
PTO
PHS
NHS
NTO
NHS
Time

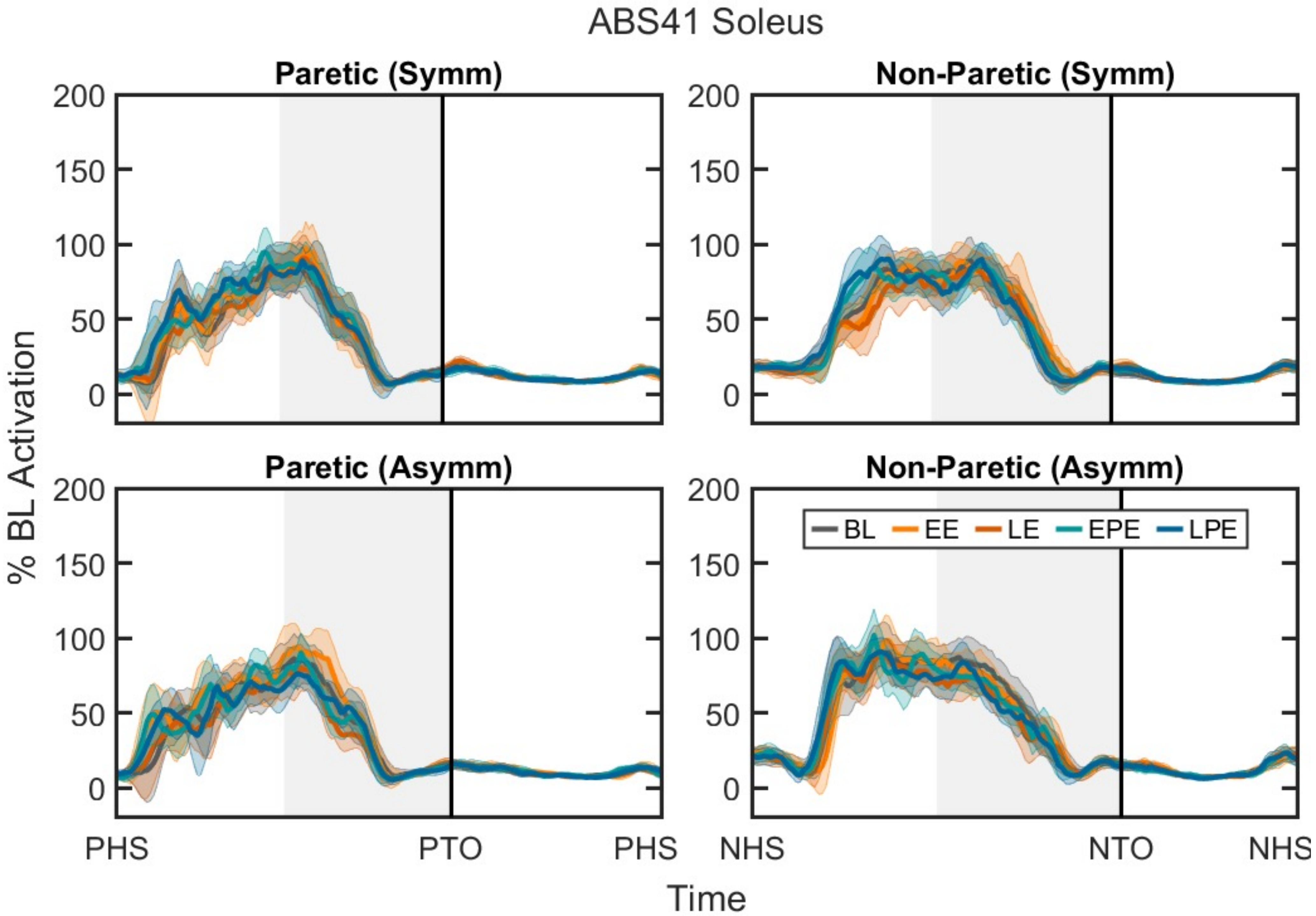
ABS41 Soleus
Paretic (Symm)
Non-Paretic (Symm)
Paretic (Asymm)
Non-Paretic (Asymm)
% BL Activation
200
150
100
50
0
BL
EE
LE
EPE
LPE
PHS
PTO
PHS
NHS
NTO
NHS
Time

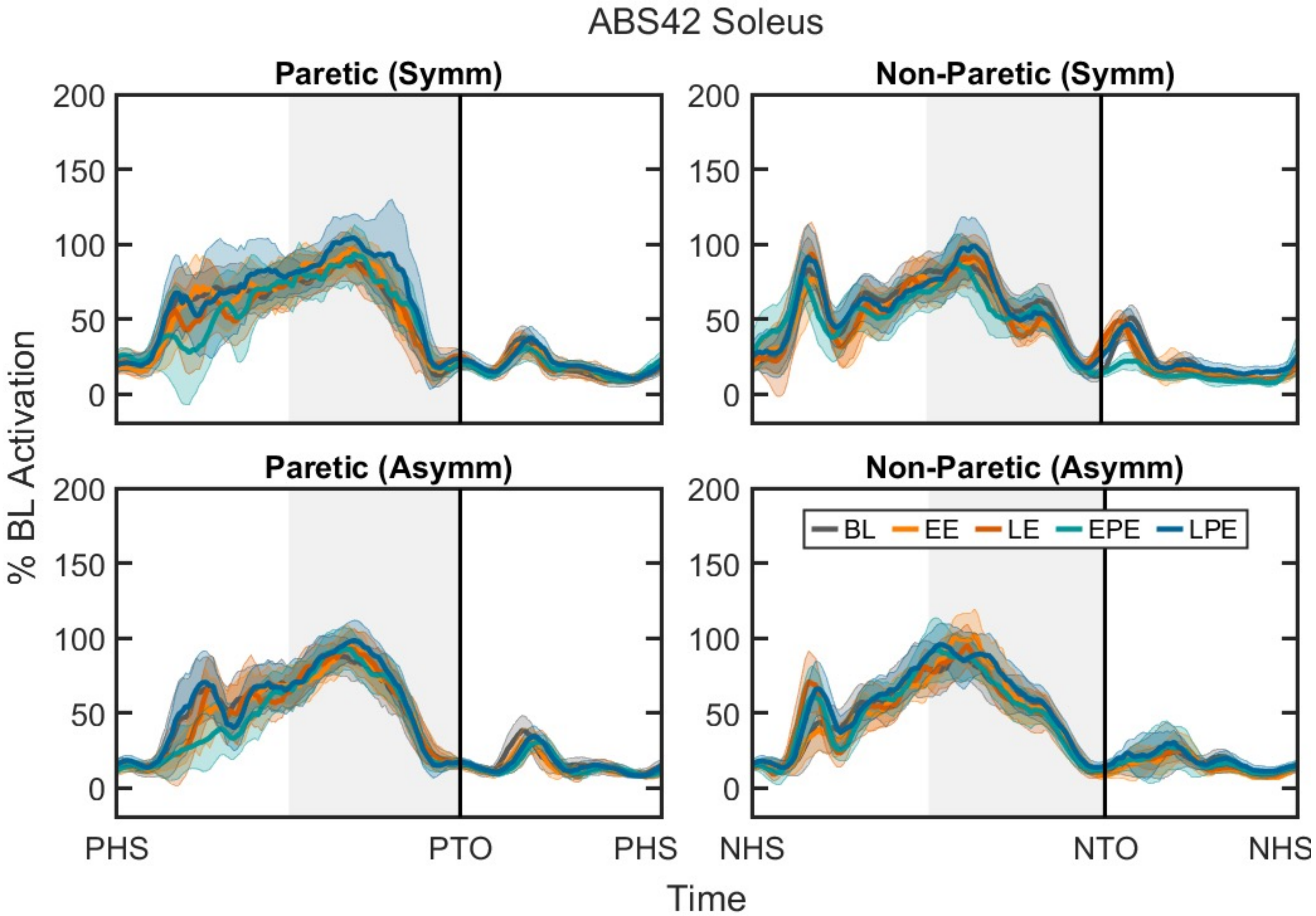
ABS42 Soleus
Paretic (Symm)
Non-Paretic (Symm)
Paretic (Asymm)
Non-Paretic (Asymm)
% BL Activation
200
150
100
50
0
BL
EE
LE
EPE
LPE
PHS
PTO
PHS
NHS
NTO
NHS
Time

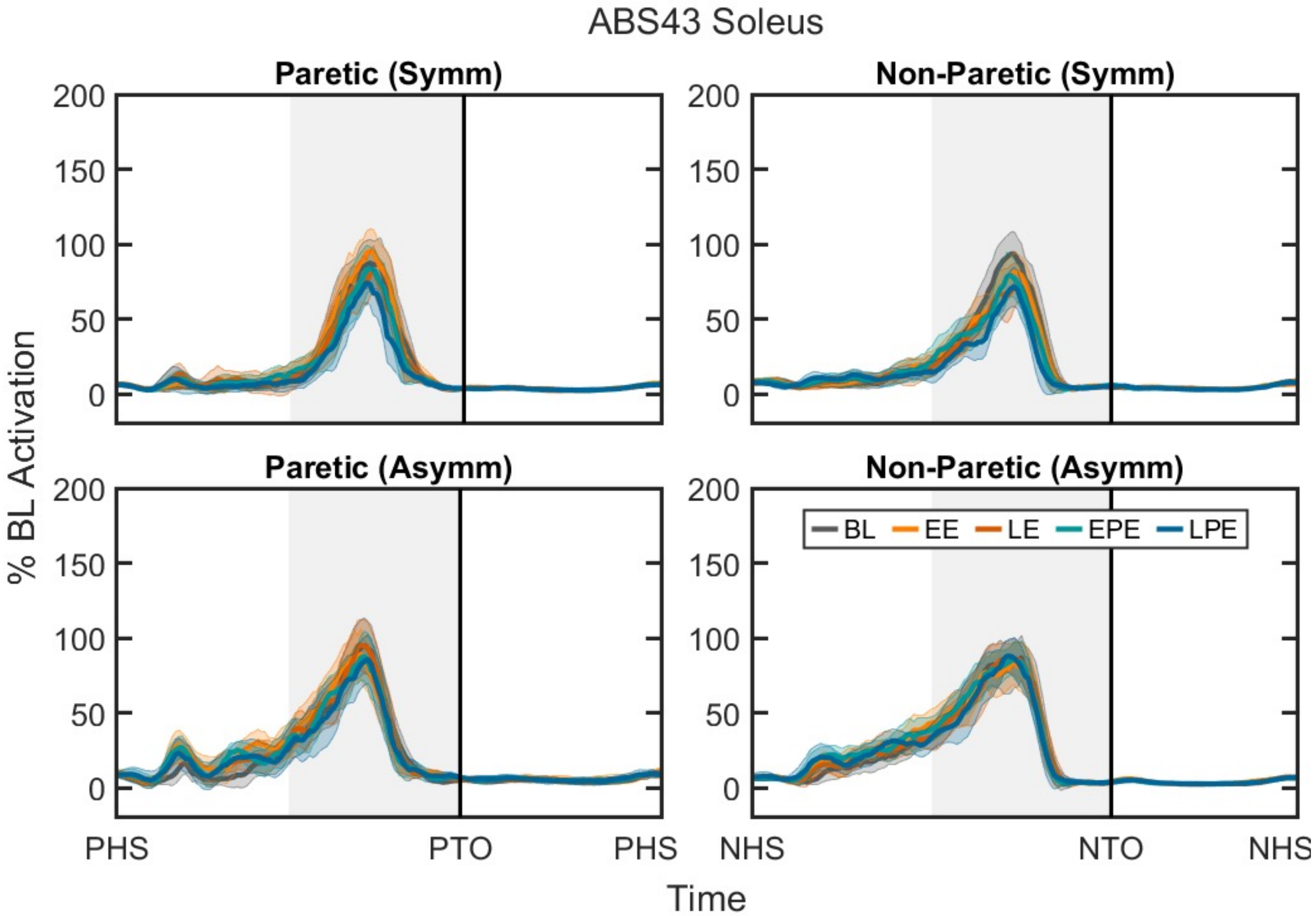
ABS43 Soleus
Paretic (Symm)
Non-Paretic (Symm)
Paretic (Asymm)
Non-Paretic (Asymm)
% BL Activation
200
150
100
50
0
BL
EE
LE
EPE
LPE
PHS
PTO
PHS
NHS
NTO
NHS
Time

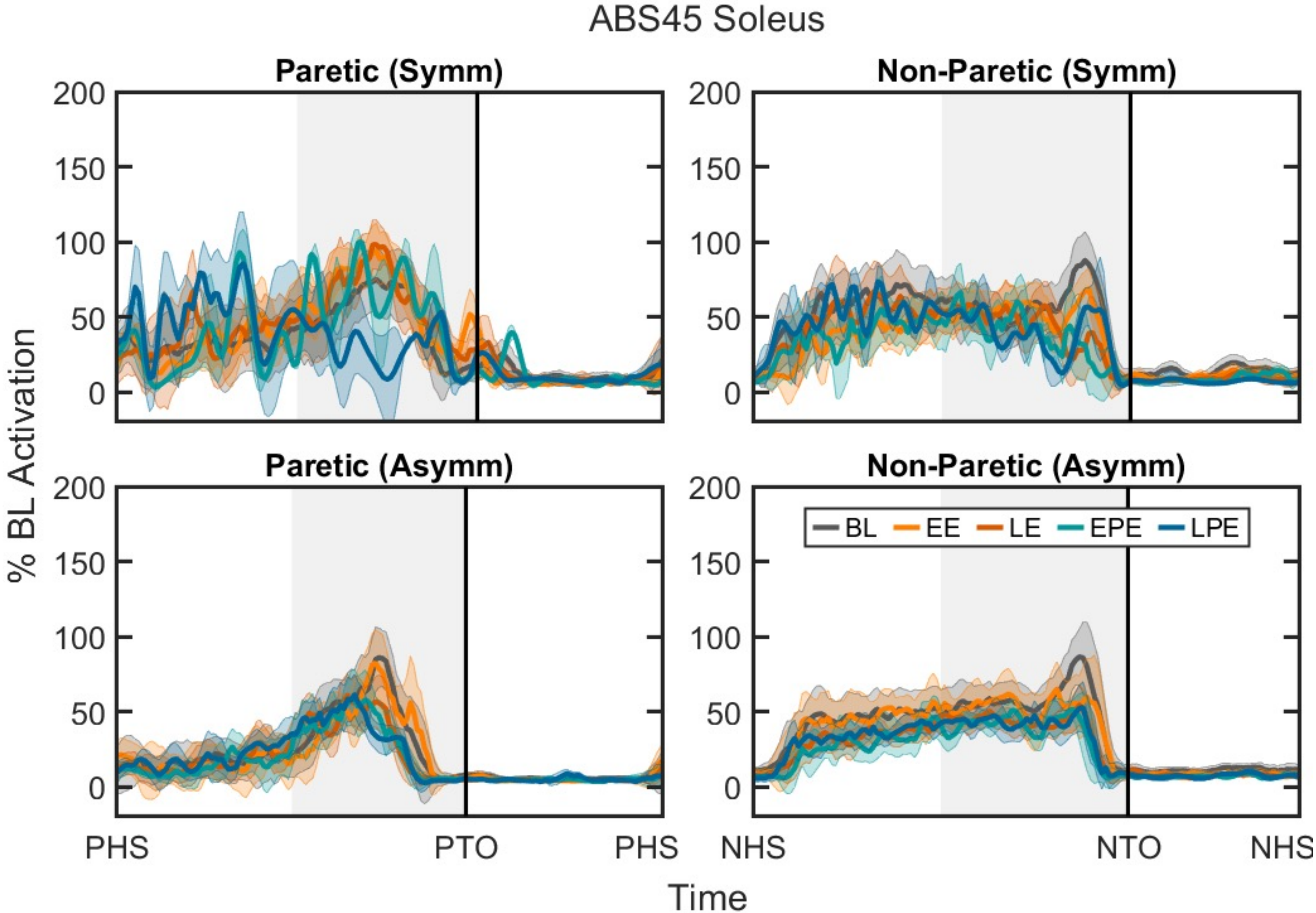
ABS45 Soleus
Paretic (Symm)
Non-Paretic (Symm)
Paretic (Asymm)
Non-Paretic (Asymm)
% BL Activation
200
150
100
50
0
BL
EE
LE
EPE
LPE
PHS
PTO
PHS
NHS
NTO
NHS
Time

Lateral Gastrocnemius

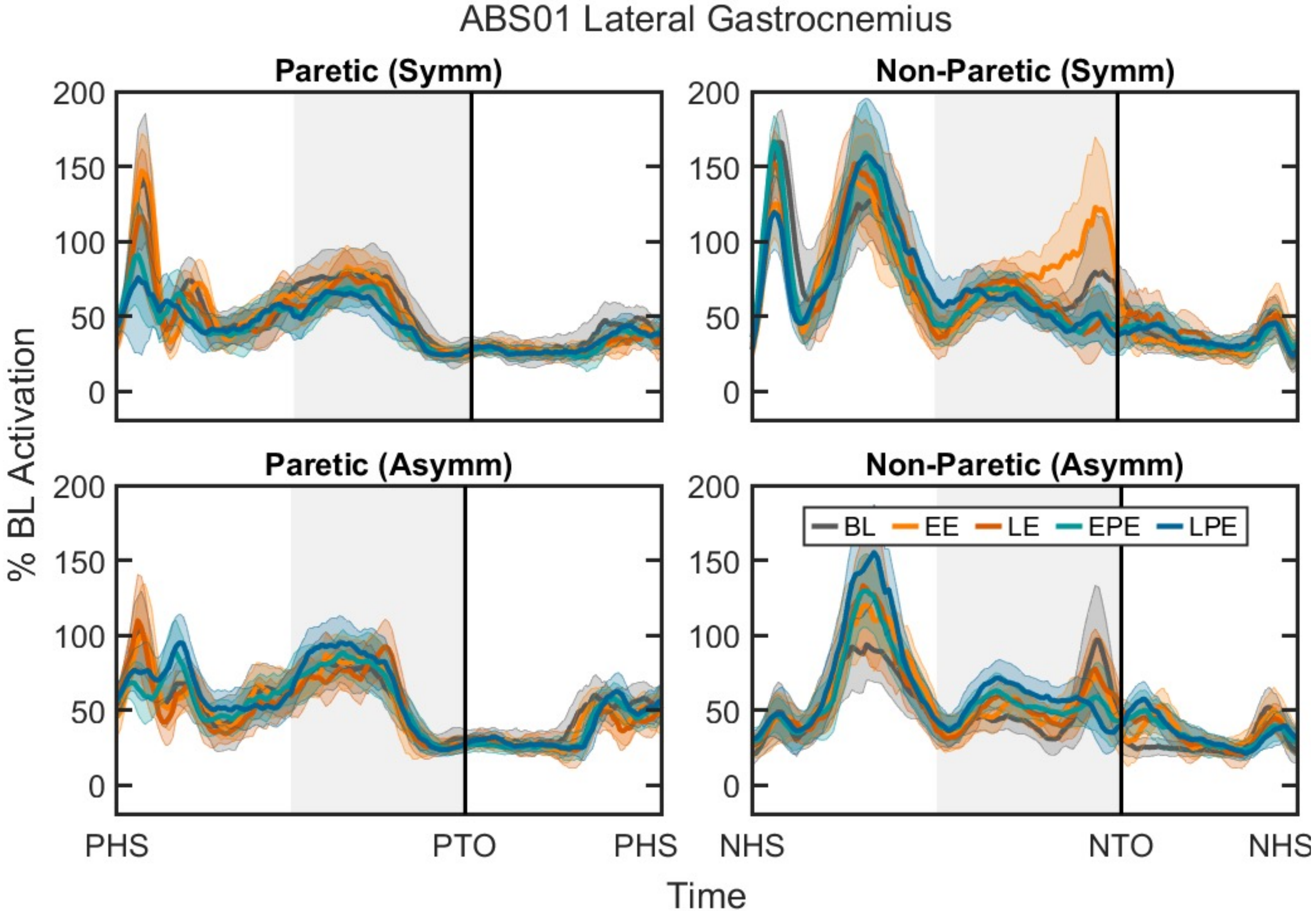
ABS01 Lateral Gastrocnemius
Paretic (Symm)
Non-Paretic (Symm)
Paretic (Asymm)
Non-Paretic (Asymm)
% BL Activation
200
150
100
50
0
BL
EE
LE
EPE
LPE
PHS
PTO
PHS
NHS
NTO
NHS
Time

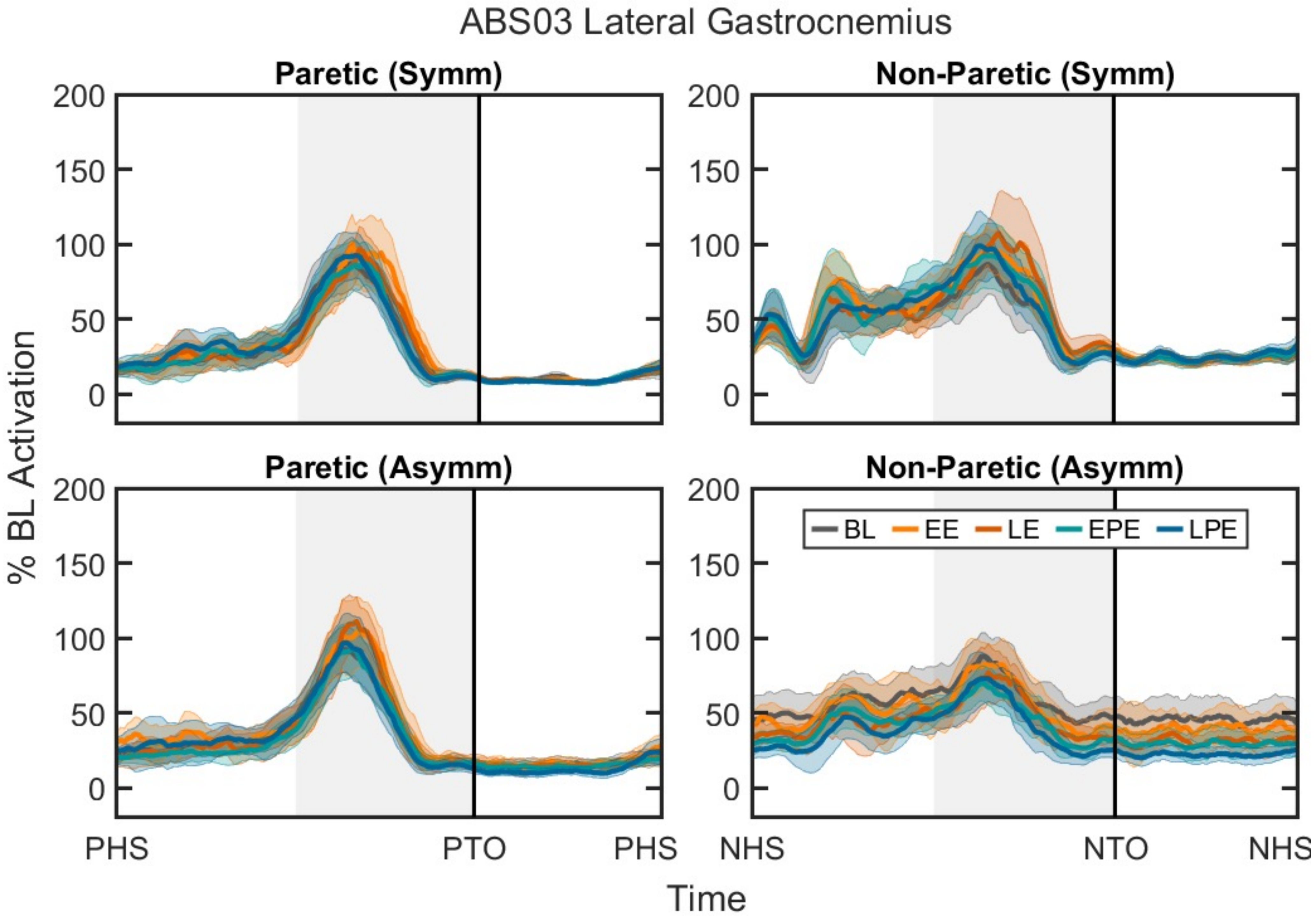
ABS03 Lateral Gastrocnemius
Paretic (Symm)
Non-Paretic (Symm)
Paretic (Asymm)
Non-Paretic (Asymm)
% BL Activation
200
150
100
50
0
BL
EE
LE
EPE
LPE
PHS
PTO
PHS
NHS
NTO
NHS
Time

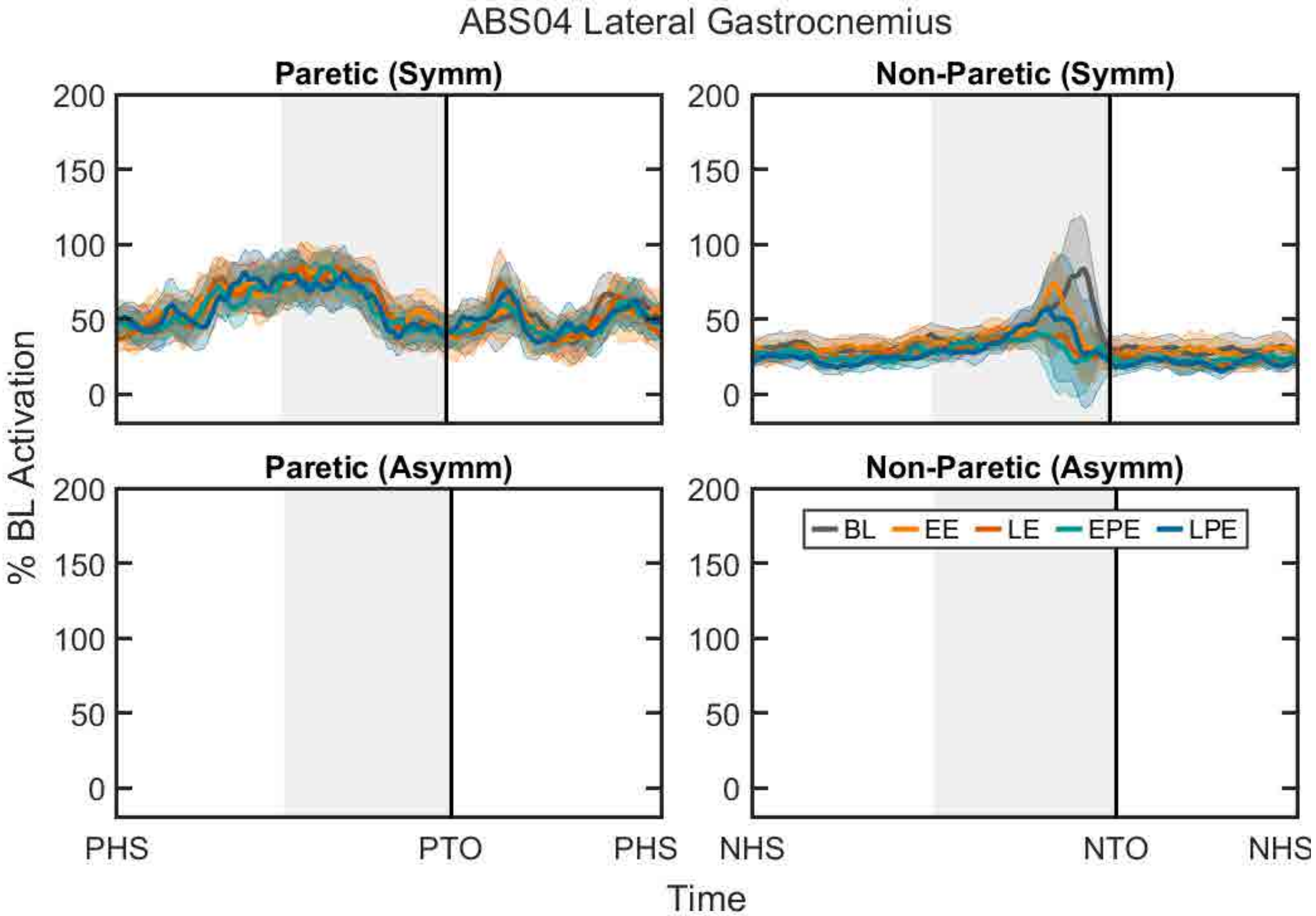
ABS04 Lateral Gastrocnemius
Paretic (Symm)
Non-Paretic (Symm)
Paretic (Asymm)
Non-Paretic (Asymm)
% BL Activation
200
150
100
50
0
BL
EE
LE
EPE
LPE
PHS
PTO
PHS
NHS
NTO
NHS
Time

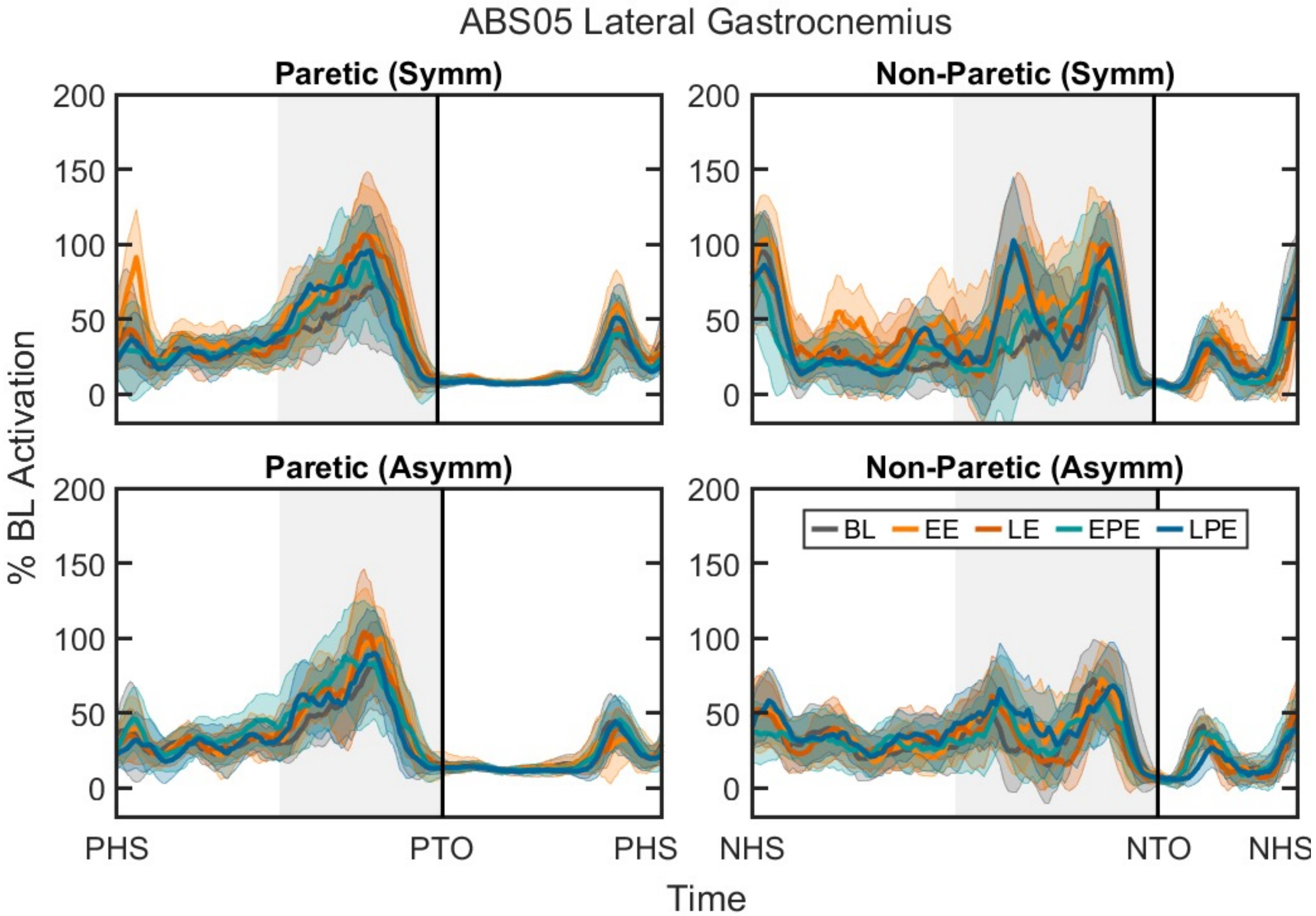
ABS05 Lateral Gastrocnemius
Paretic (Symm)
Non-Paretic (Symm)
Paretic (Asymm)
Non-Paretic (Asymm)
% BL Activation
200
150
100
50
0
BL
EE
LE
EPE
LPE
PHS
PTO
PHS
NHS
NTO
NHS
Time

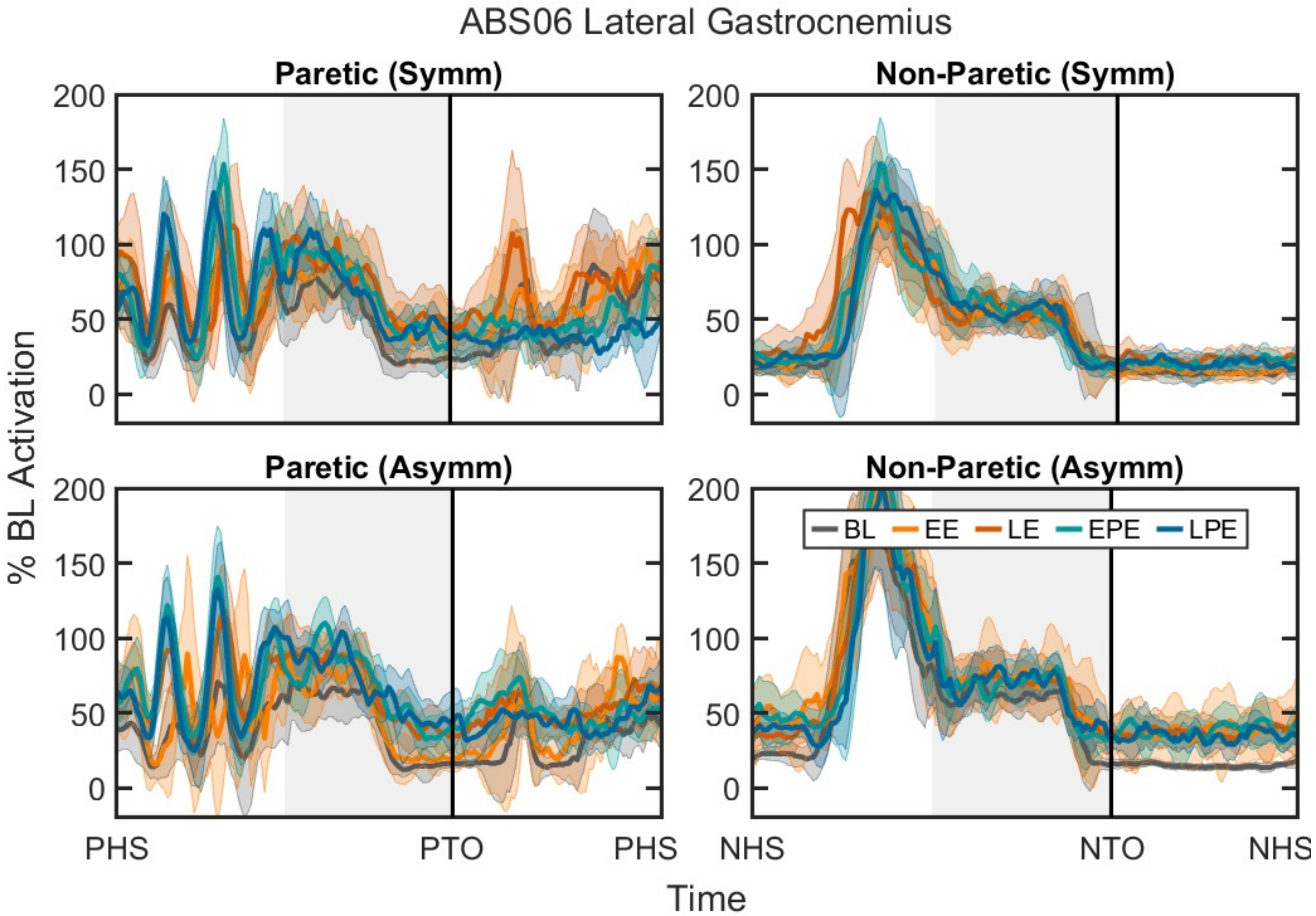

ABS06 Lateral Gastrocnemius
Paretic (Symm)
Non-Paretic (Symm)
Paretic (Asymm)
Non-Paretic (Asymm)
% BL Activation
200
150
100
50
0
BL
EE
LE
EPE
LPE
PHS
PTO
PHS
NHS
NTO
NHS
Time

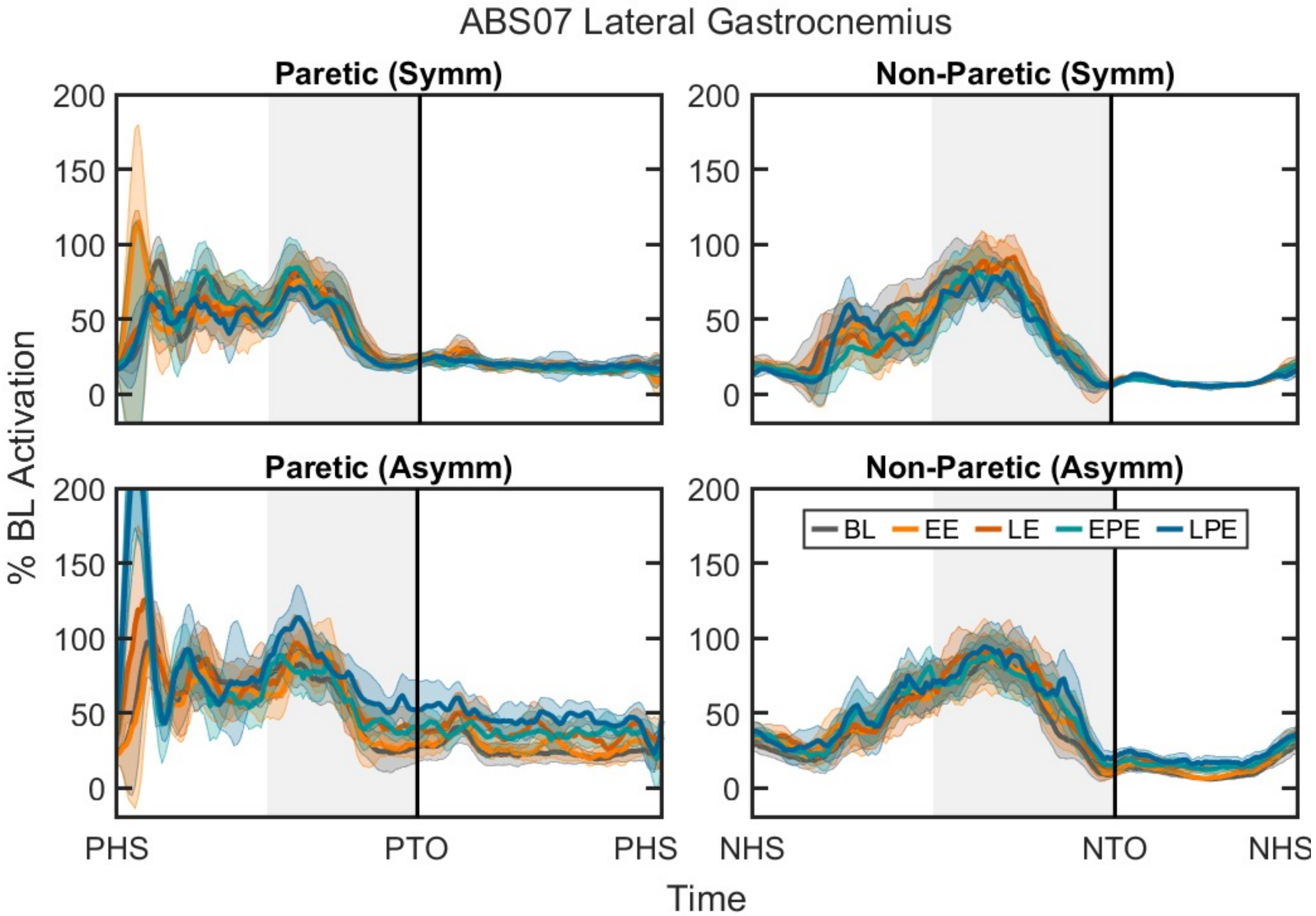
ABS07 Lateral Gastrocnemius
Paretic (Symm)
Non-Paretic (Symm)
Paretic (Asymm)
Non-Paretic (Asymm)
% BL Activation
200
150
100
50
0
BL
EE
LE
EPE
LPE
PHS
PTO
PHS
NHS
NTO
NHS
Time

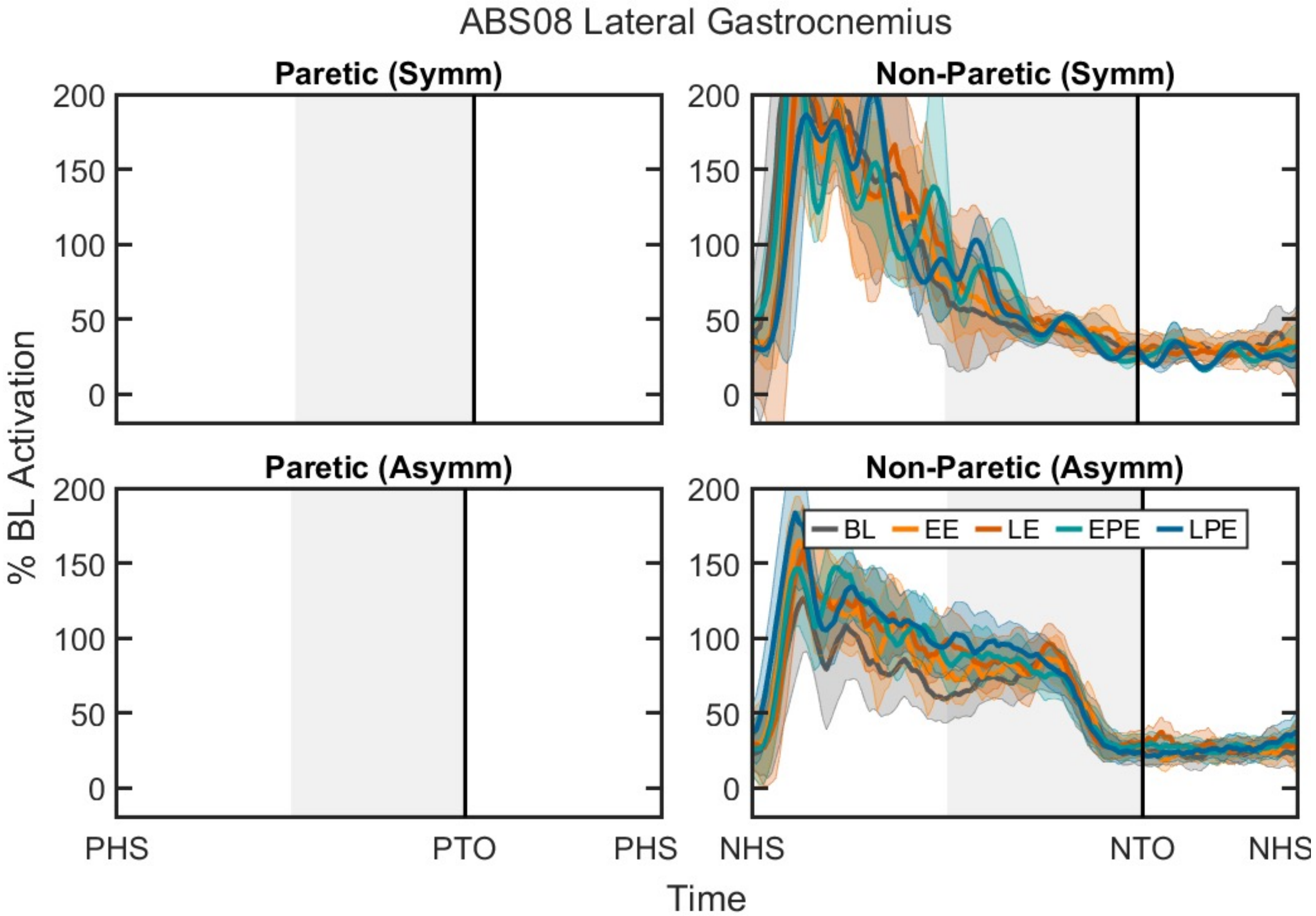
ABS08 Lateral Gastrocnemius
Paretic (Symm)
Non-Paretic (Symm)
Paretic (Asymm)
Non-Paretic (Asymm)
% BL Activation
200
150
100
50
0
BL
EE
LE
EPE
LPE
PHS
PTO
PHS
NHS
NTO
NHS
Time

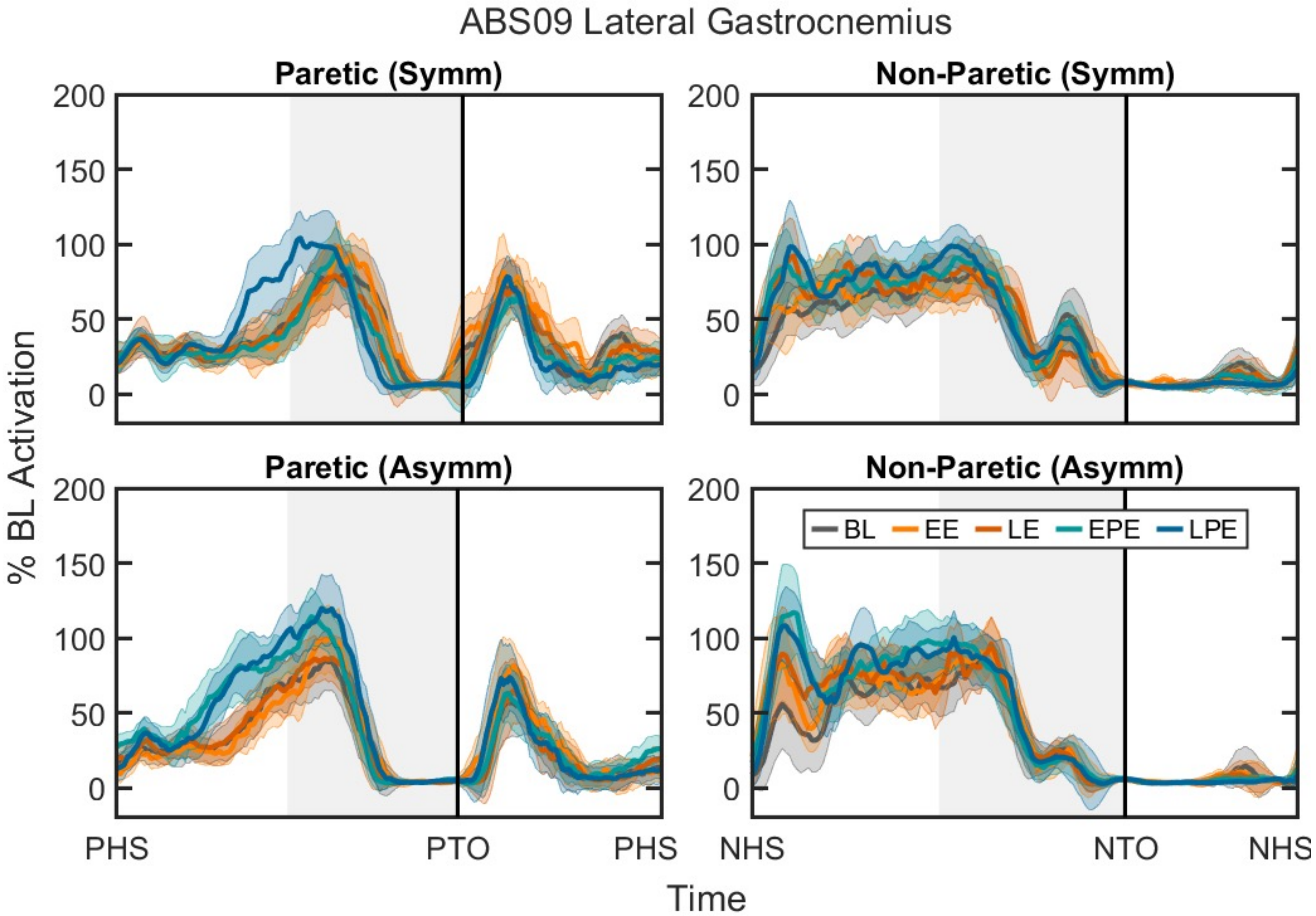
ABS09 Lateral Gastrocnemius
Paretic (Symm)
Non-Paretic (Symm)
Paretic (Asymm)
Non-Paretic (Asymm)
% BL Activation
200
150
100
50
0
BL
EE
LE
EPE
LPE
PHS
PTO
PHS
NHS
NTO
NHS
Time

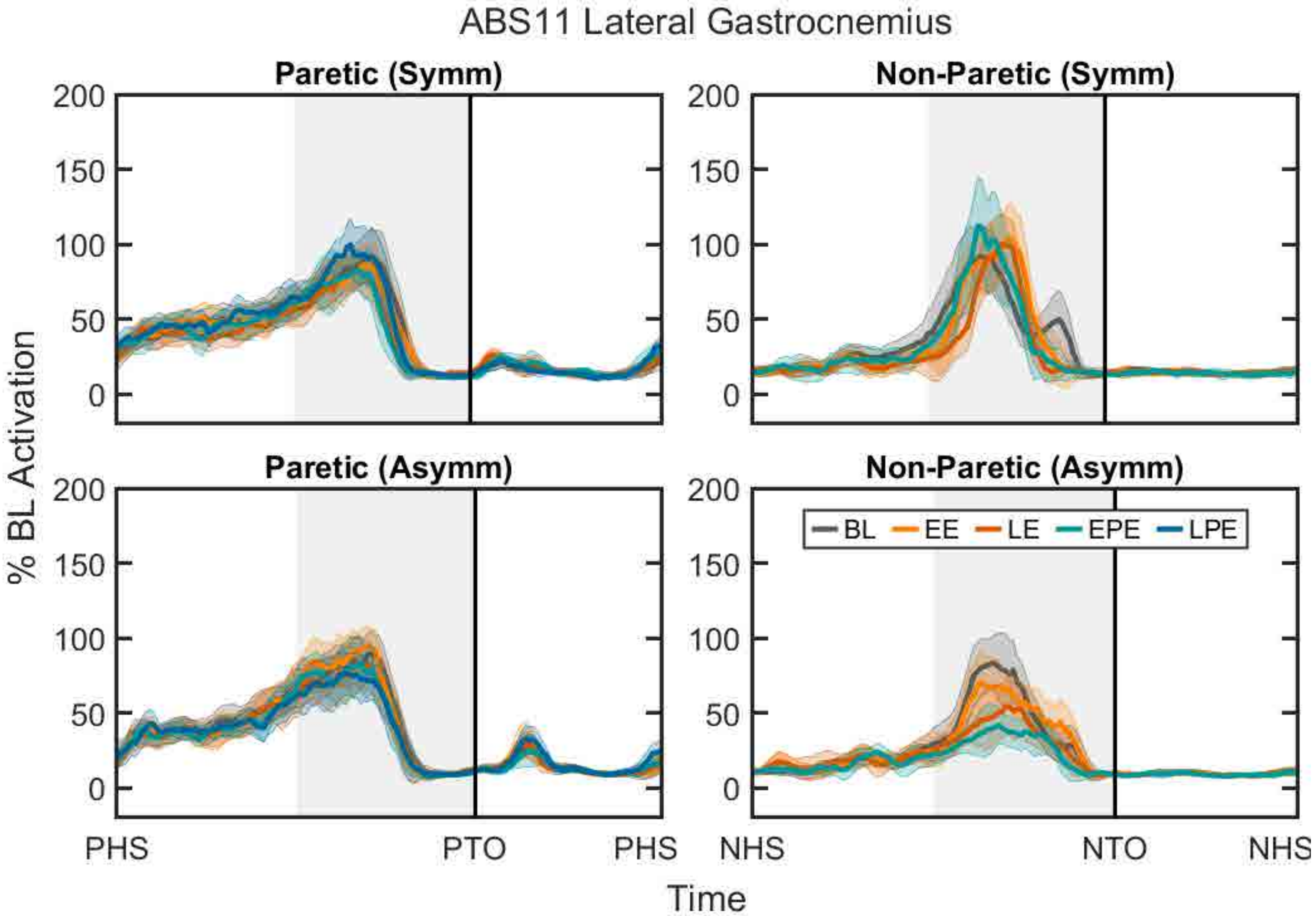

ABS11 Lateral Gastrocnemius
Paretic (Symm)
Non-Paretic (Symm)
Paretic (Asymm)
Non-Paretic (Asymm)
% BL Activation
200
150
100
50
0
BL
EE
LE
EPE
LPE
PHS
PTO
PHS
NHS
NTO
NHS
Time

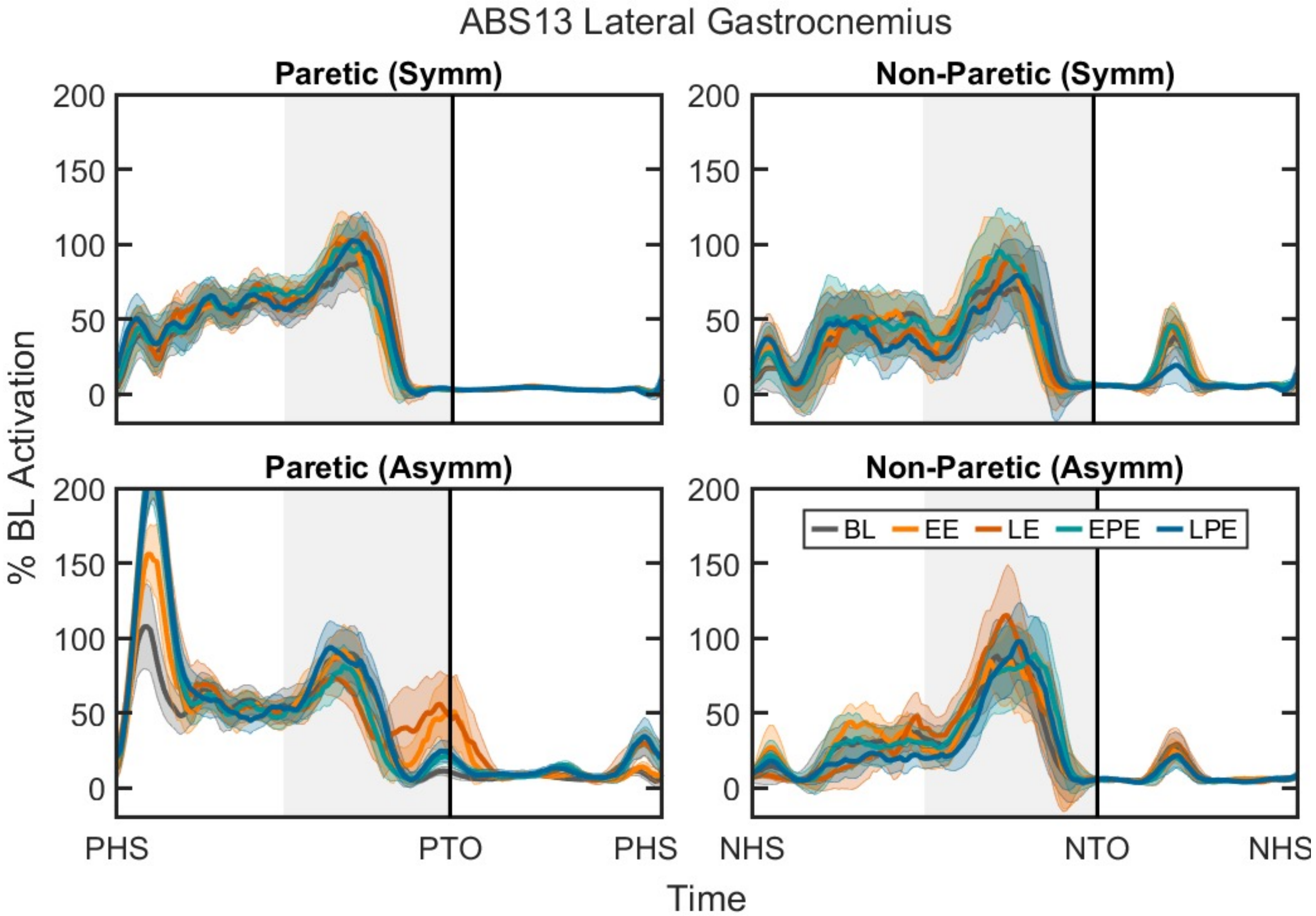

ABS13 Lateral Gastrocnemius
Paretic (Symm)
Non-Paretic (Symm)
Paretic (Asymm)
Non-Paretic (Asymm)
% BL Activation
200
150
100
50
0
BL
EE
LE
EPE
LPE
PHS
PTO
PHS
NHS
NTO
NHS
Time

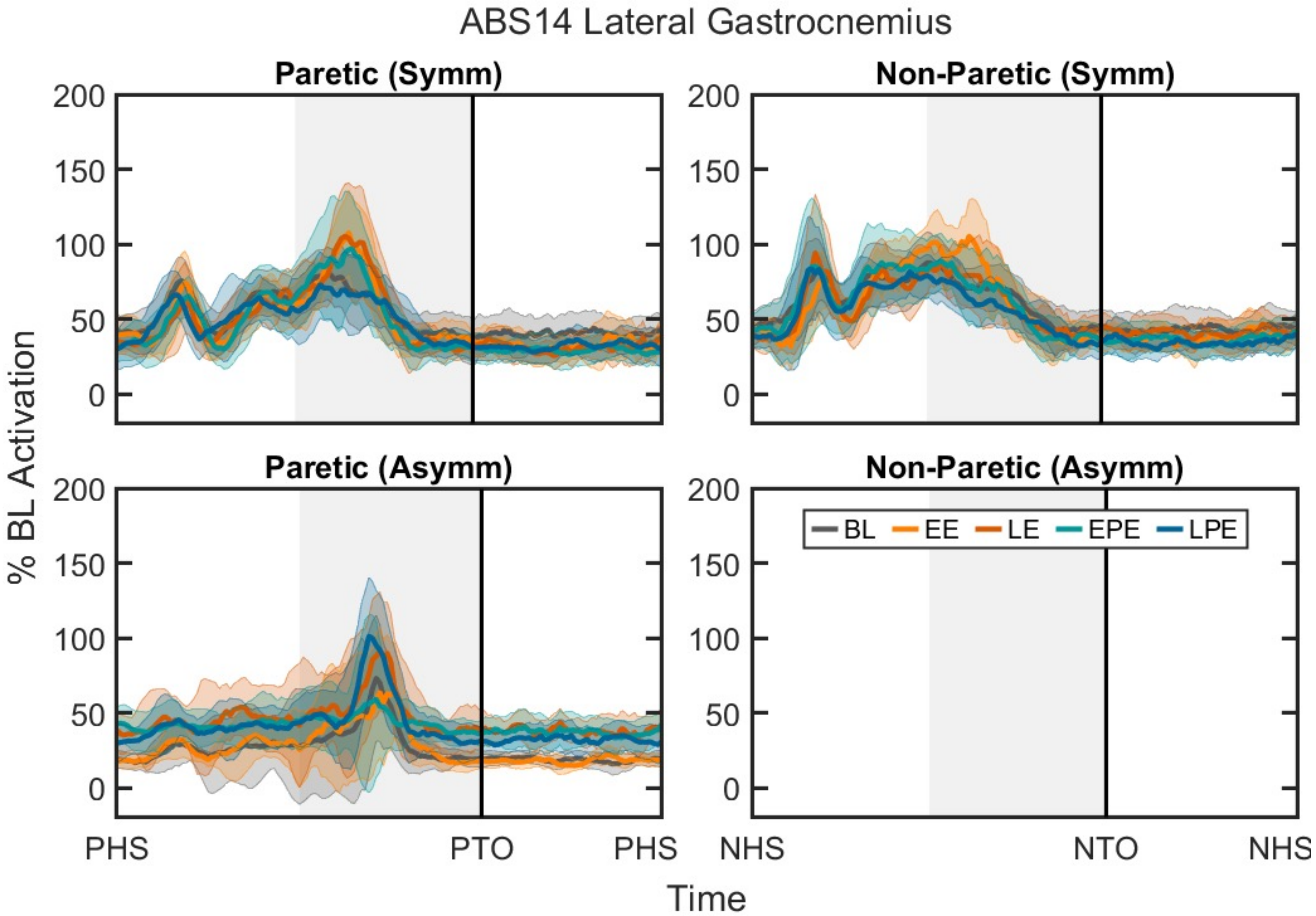
ABS14 Lateral Gastrocnemius
Paretic (Symm)
Non-Paretic (Symm)
Paretic (Asymm)
Non-Paretic (Asymm)
% BL Activation
200
150
100
50
0
BL
EE
LE
EPE
LPE
PHS
PTO
PHS
NHS
NTO
NHS
Time

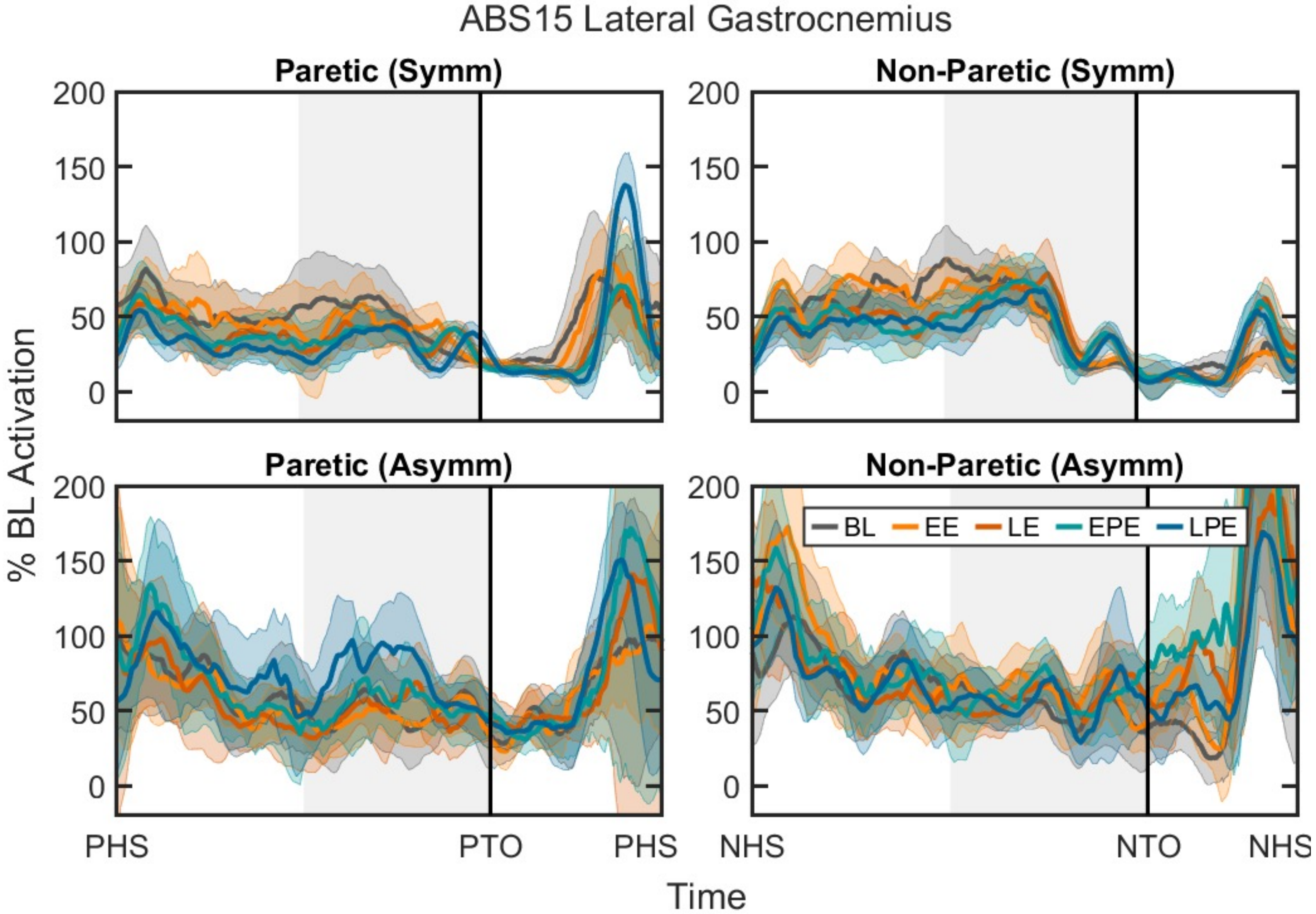

ABS15 Lateral Gastrocnemius
Paretic (Symm)
Non-Paretic (Symm)
Paretic (Asymm)
Non-Paretic (Asymm)
% BL Activation
200
150
100
50
0
BL
EE
LE
EPE
LPE
PHS
PTO
PHS
NHS
NTO
NHS
Time

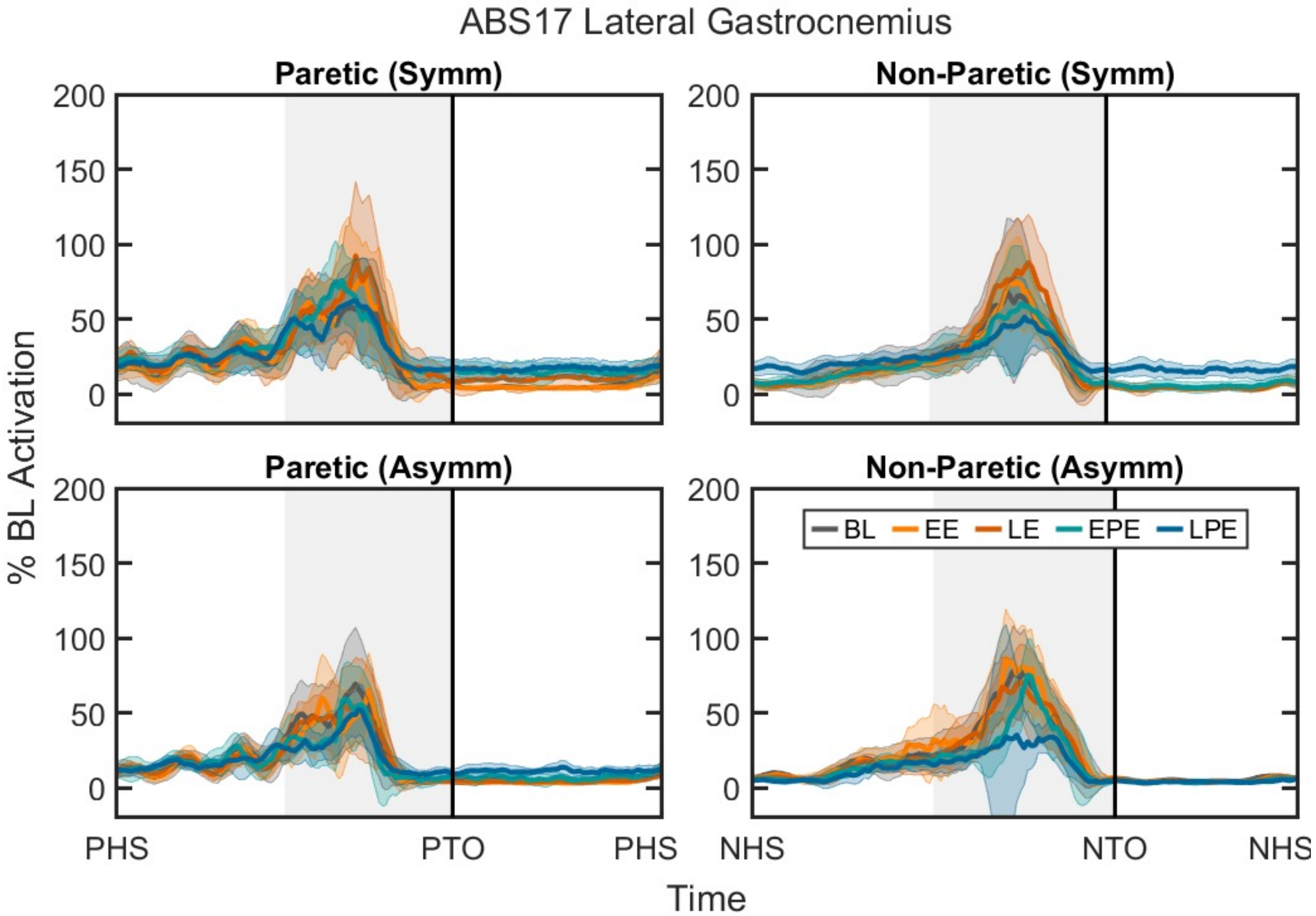
ABS17 Lateral Gastrocnemius
Paretic (Symm)
Non-Paretic (Symm)
Paretic (Asymm)
Non-Paretic (Asymm)
BL
EE
LE
EPE
LPE
% BL Activation
200
150
100
50
0
PHS
PTO
PHS
NHS
NTO
NHS
Time

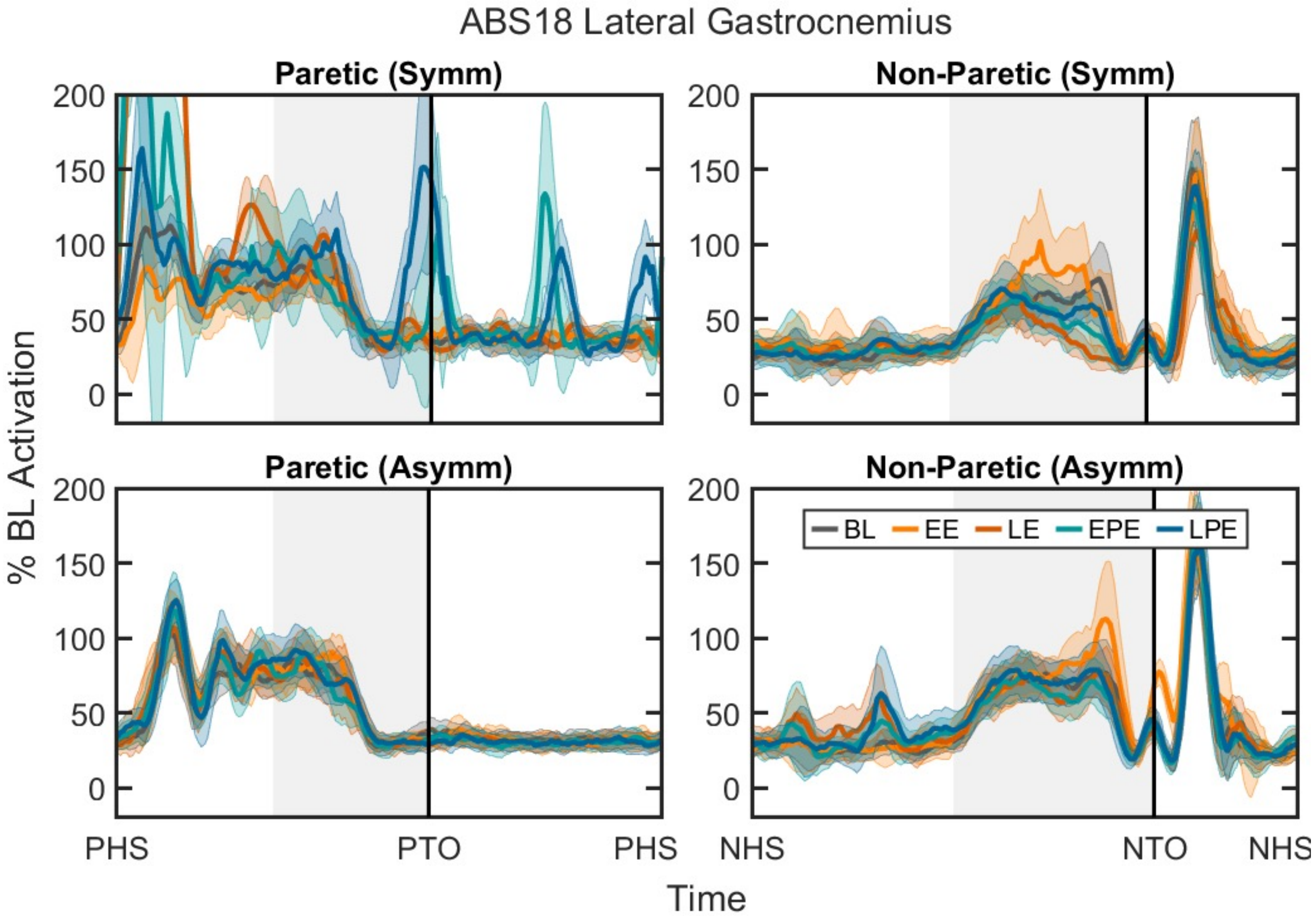
ABS18 Lateral Gastrocnemius
Paretic (Symm)
Non-Paretic (Symm)
Paretic (Asymm)
Non-Paretic (Asymm)
% BL Activation
200
150
100
50
0
BL
EE
LE
EPE
LPE
PHS
PTO
PHS
NHS
NTO
NHS
Time

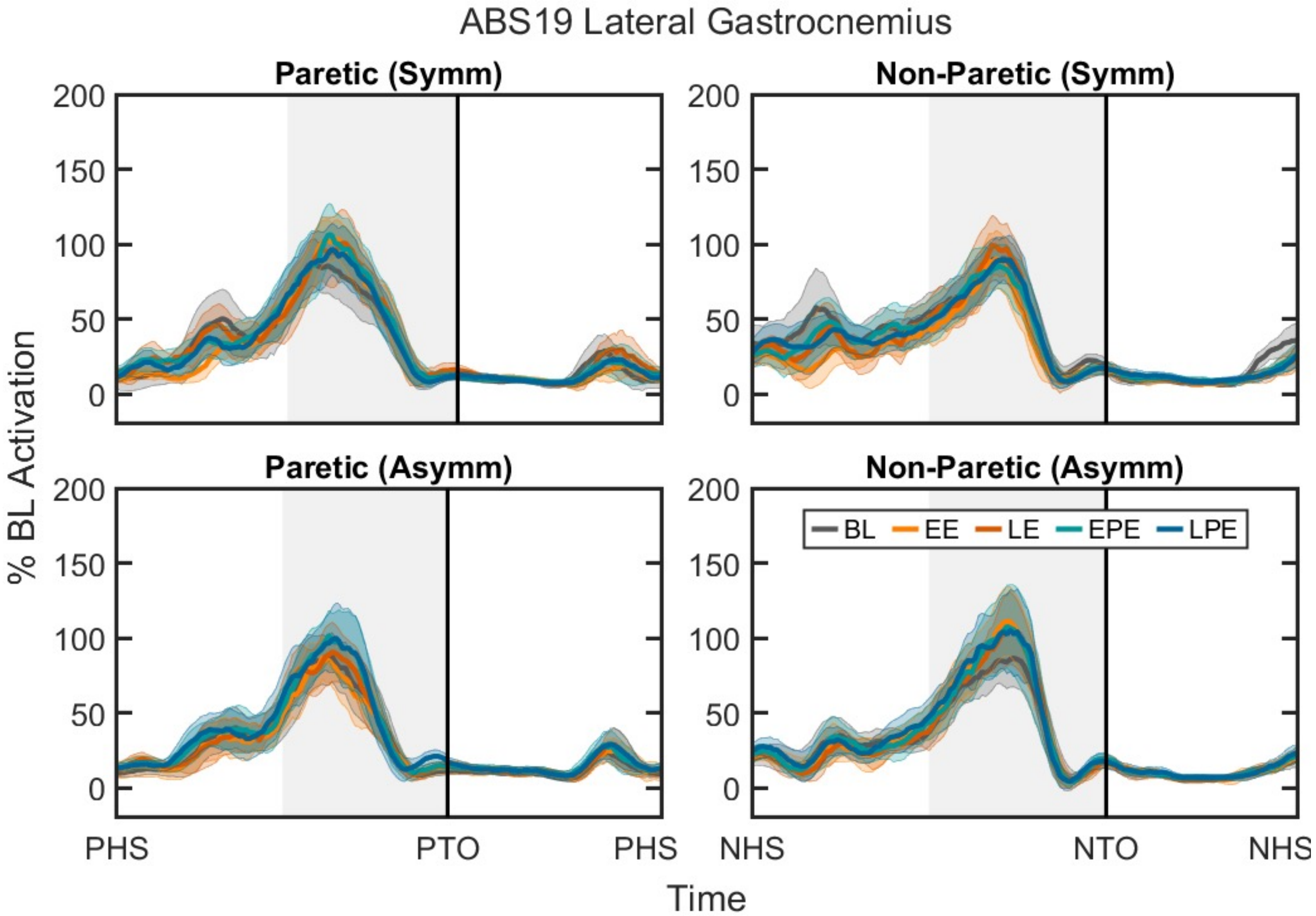
ABS19 Lateral Gastrocnemius
Paretic (Symm)
Non-Paretic (Symm)
Paretic (Asymm)
Non-Paretic (Asymm)
% BL Activation
200
150
100
50
0
BL
EE
LE
EPE
LPE
PHS
PTO
PHS
NHS
NTO
NHS
Time

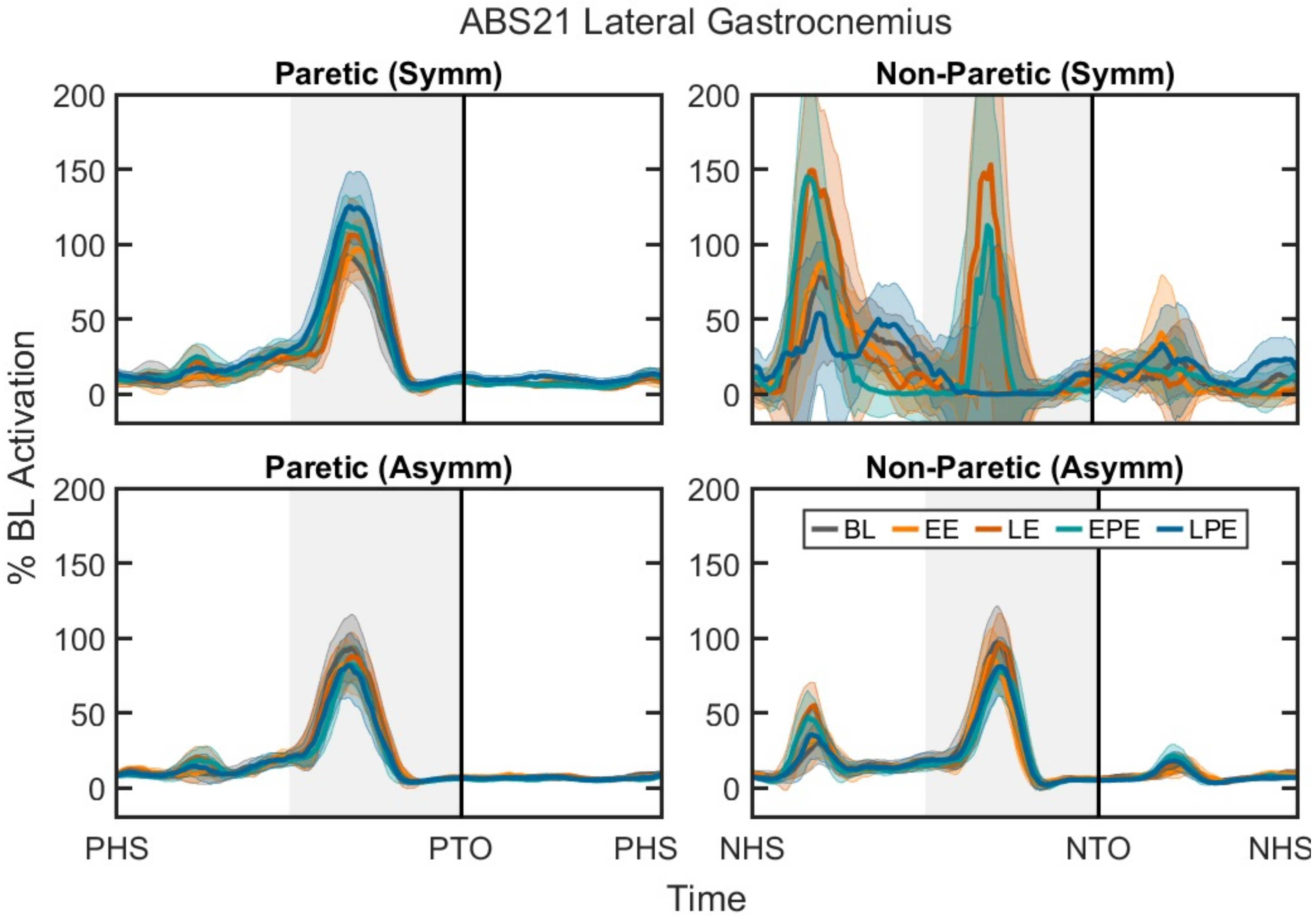

ABS21 Lateral Gastrocnemius
Paretic (Symm)
Non-Paretic (Symm)
Paretic (Asymm)
Non-Paretic (Asymm)
% BL Activation
200
150
100
50
0
BL
EE
LE
EPE
LPE
PHS
PTO
PHS
NHS
NTO
NHS
Time

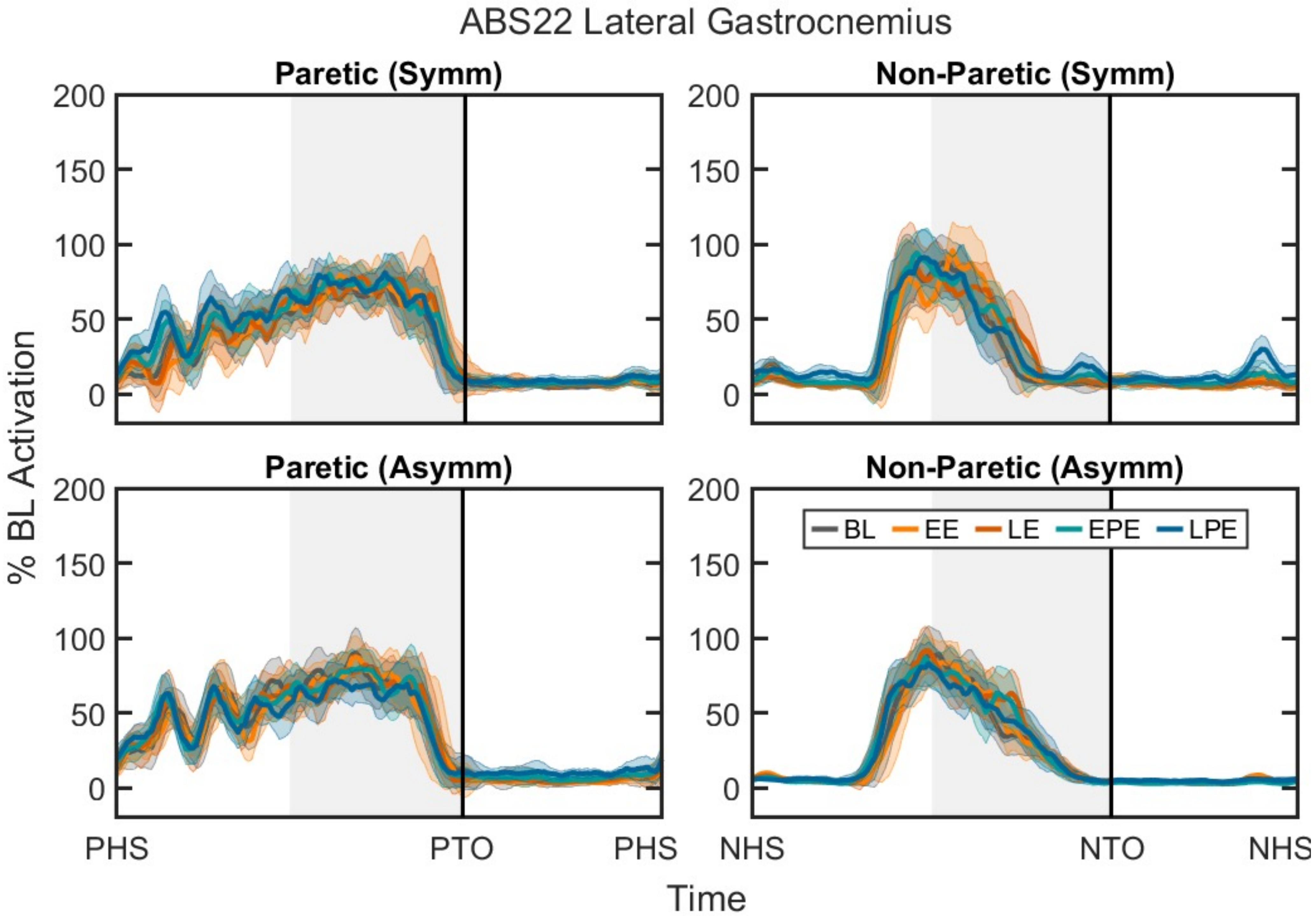
ABS22 Lateral Gastrocnemius
Paretic (Symm)
Non-Paretic (Symm)
Paretic (Asymm)
Non-Paretic (Asymm)
% BL Activation
200
150
100
50
0
BL
EE
LE
EPE
LPE
PHS
PTO
PHS
NHS
NTO
NHS
Time

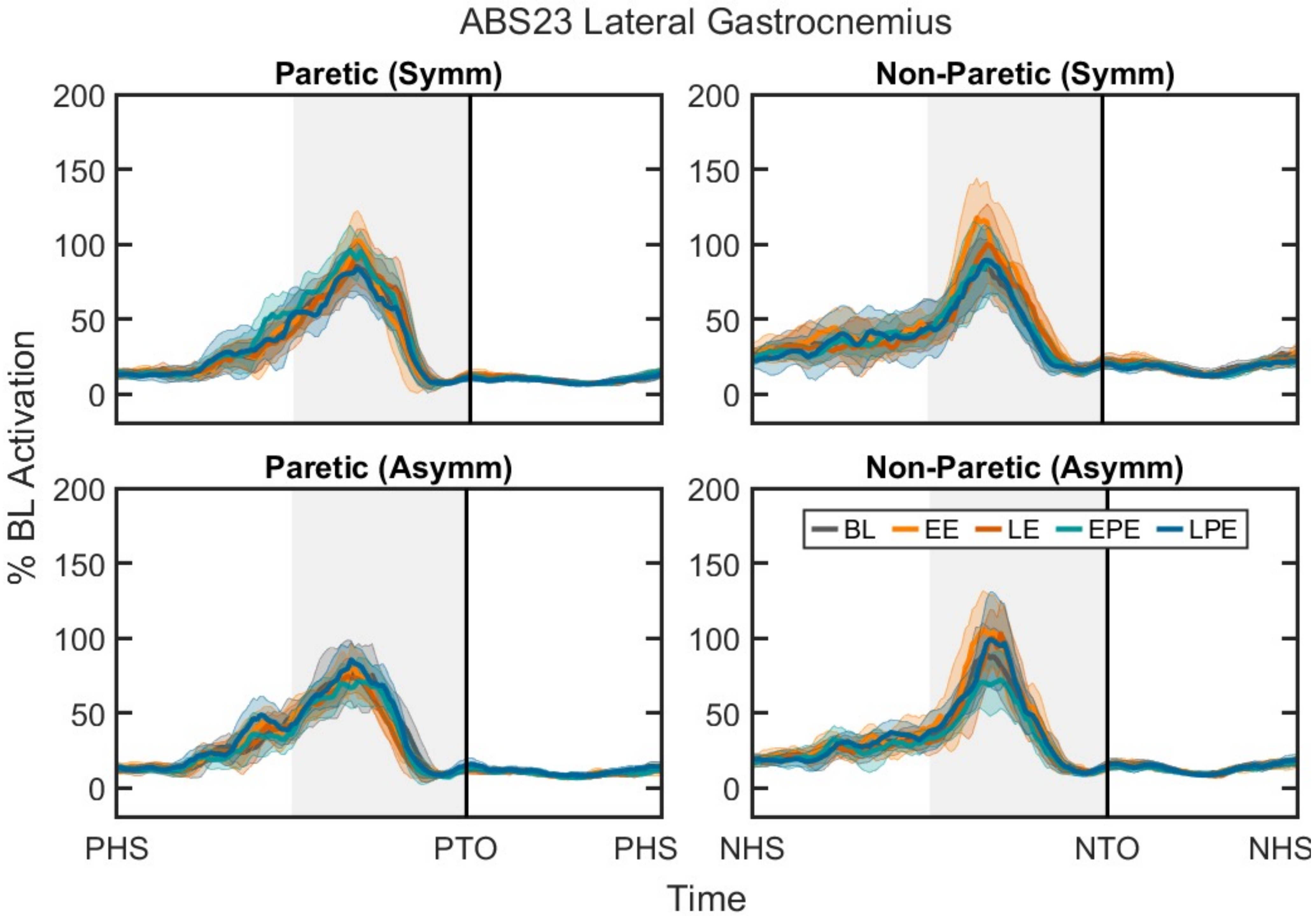

ABS23 Lateral Gastrocnemius
Paretic (Symm)
Non-Paretic (Symm)
Paretic (Asymm)
Non-Paretic (Asymm)
% BL Activation
200
150
100
50
0
BL
EE
LE
EPE
LPE
PHS
PTO
PHS
NHS
NTO
NHS
Time

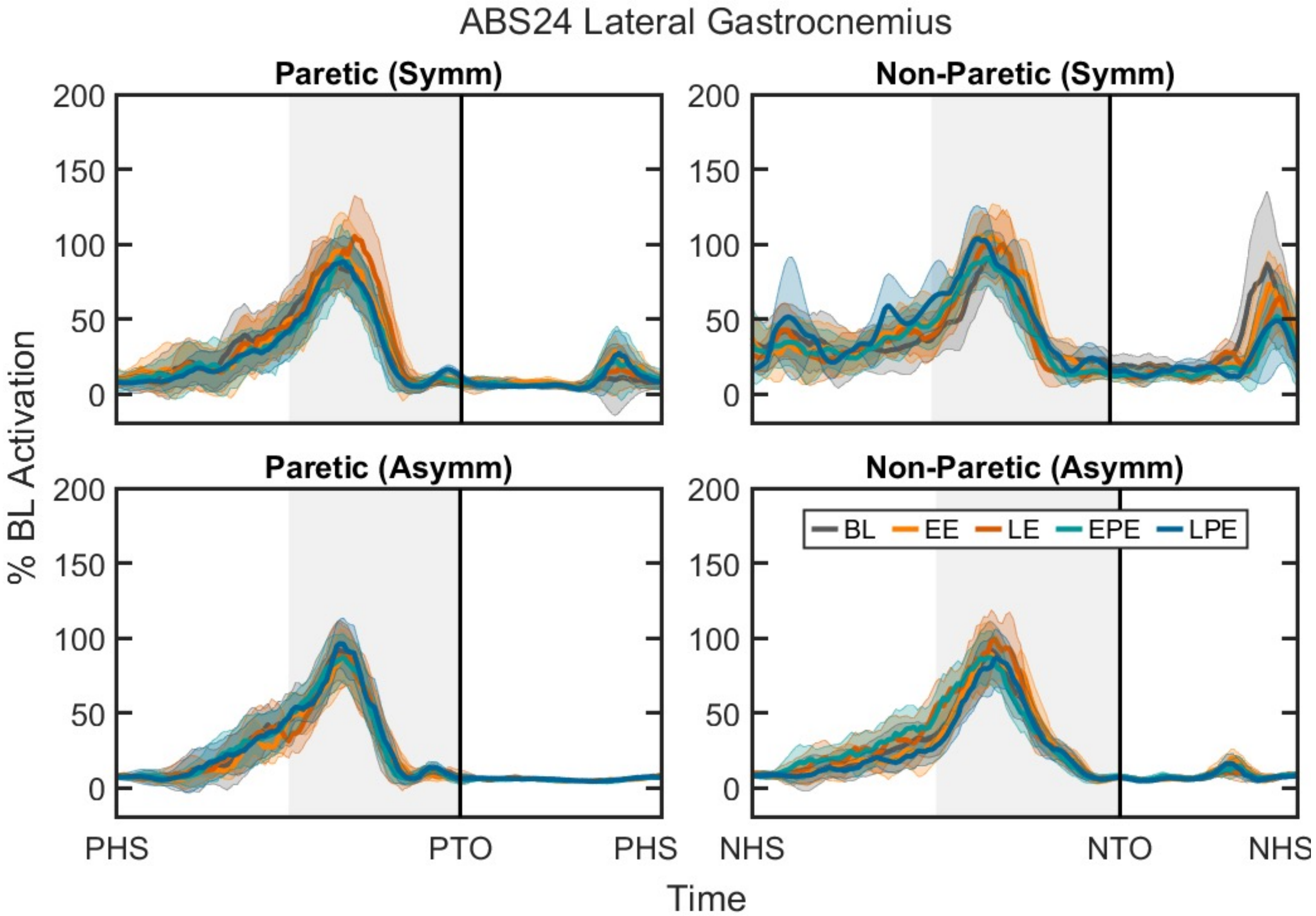
ABS24 Lateral Gastrocnemius
Paretic (Symm)
Non-Paretic (Symm)
Paretic (Asymm)
Non-Paretic (Asymm)
% BL Activation
200
150
100
50
0
BL
EE
LE
EPE
LPE
PHS
PTO
PHS
NHS
NTO
NHS
Time

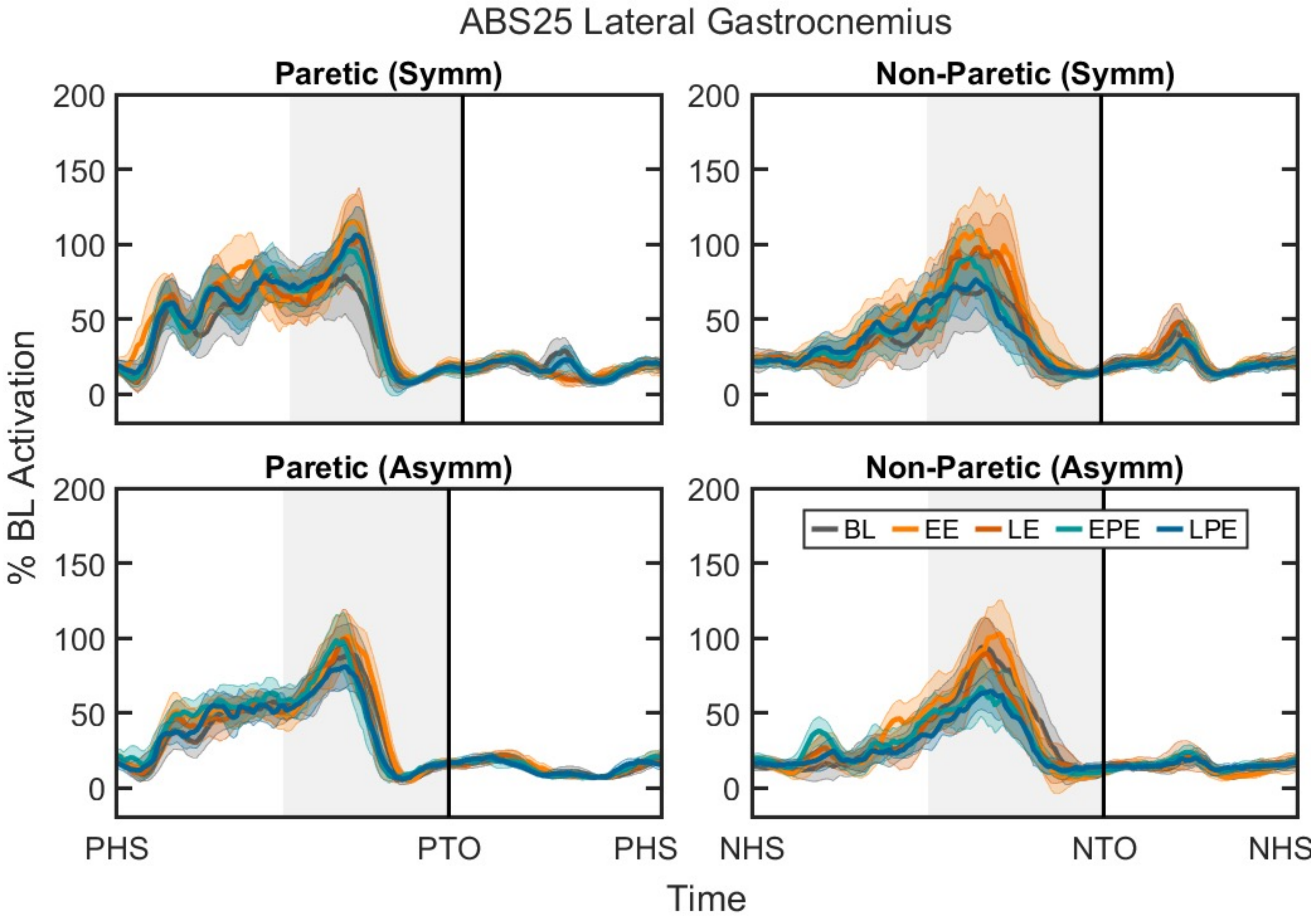
ABS25 Lateral Gastrocnemius
Paretic (Symm)
Non-Paretic (Symm)
Paretic (Asymm)
Non-Paretic (Asymm)
% BL Activation
200
150
100
50
0
BL
EE
LE
EPE
LPE
PHS
PTO
PHS
NHS
NTO
NHS
Time

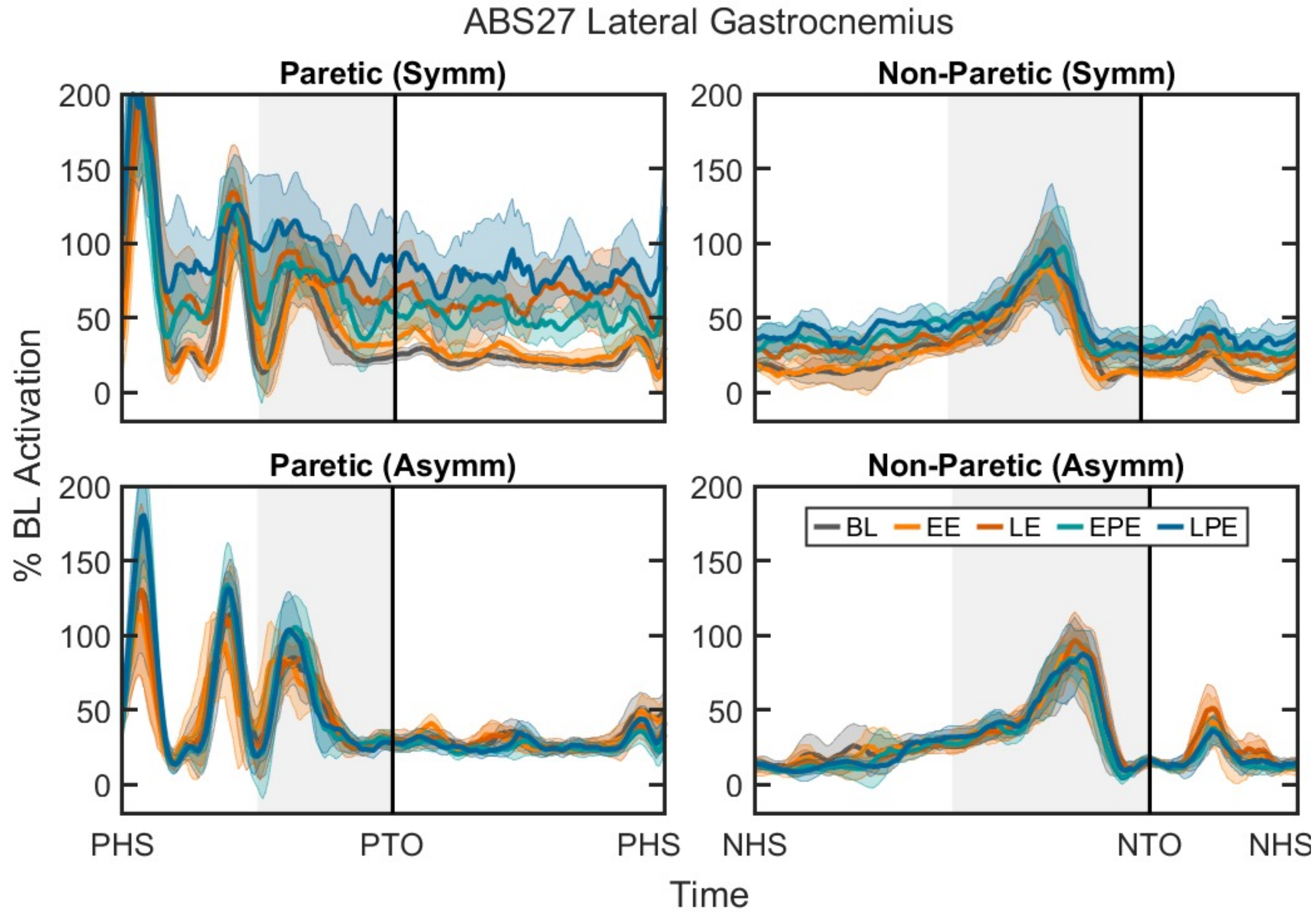
ABS27 Lateral Gastrocnemius
Paretic (Symm)
Non-Paretic (Symm)
Paretic (Asymm)
Non-Paretic (Asymm)
% BL Activation
200
150
100
50
0
BL
EE
LE
EPE
LPE
PHS
PTO
PHS
NHS
NTO
NHS
Time

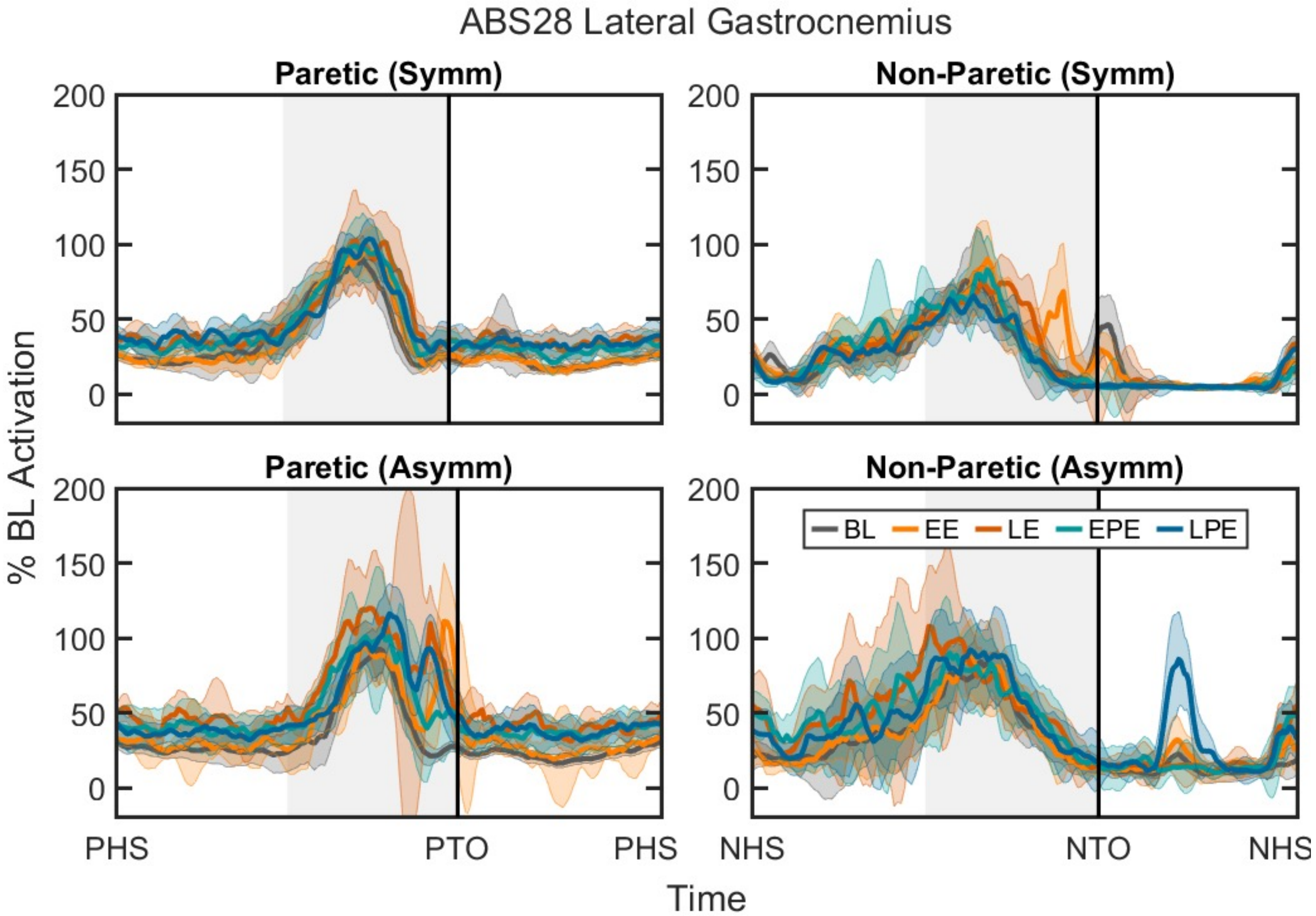
ABS28 Lateral Gastrocnemius
Paretic (Symm)
Non-Paretic (Symm)
Paretic (Asymm)
Non-Paretic (Asymm)
% BL Activation
200
150
100
50
0
BL
EE
LE
EPE
LPE
PHS
PTO
PHS
NHS
NTO
NHS
Time

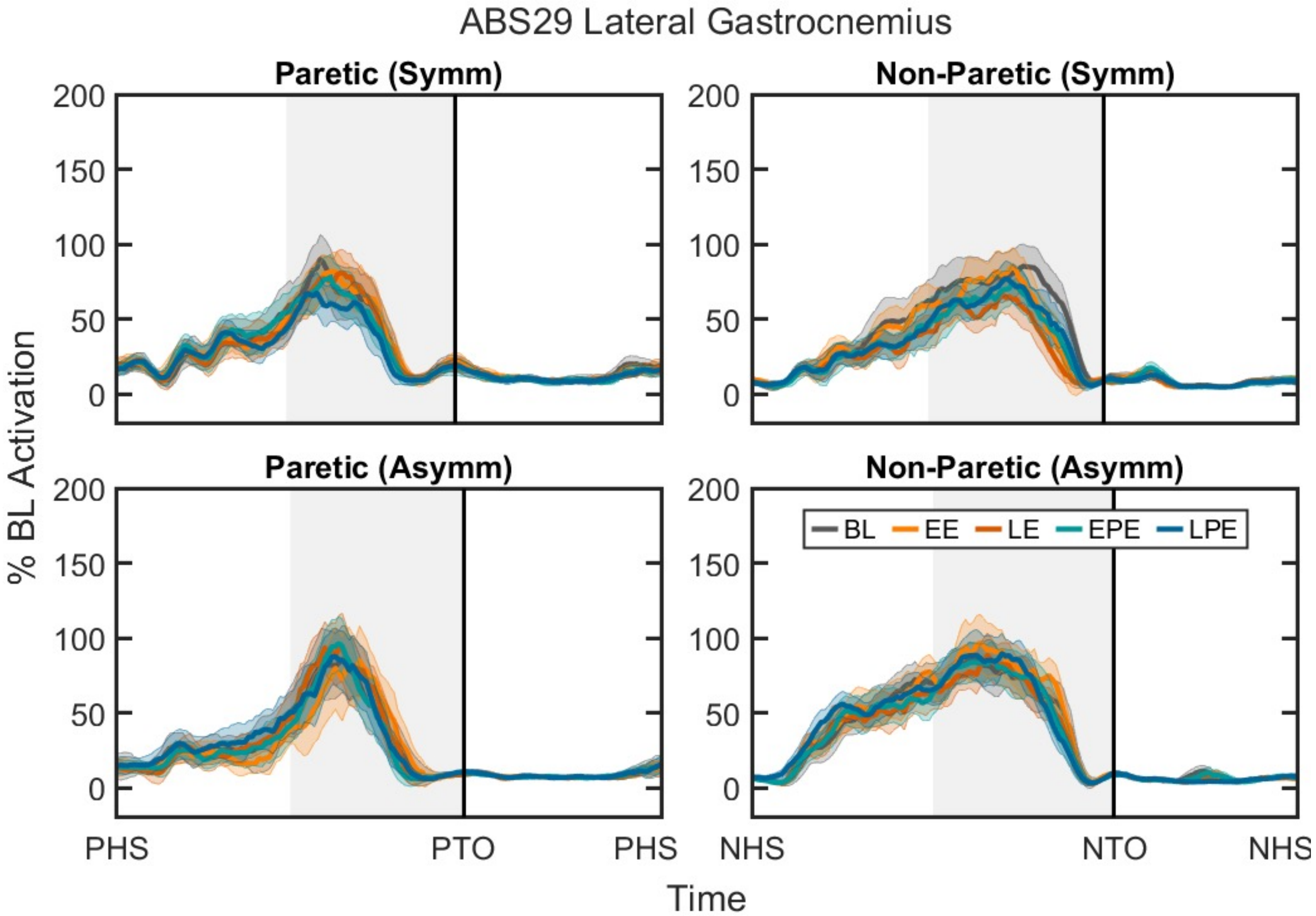
ABS29 Lateral Gastrocnemius
Paretic (Symm)
Non-Paretic (Symm)
Paretic (Asymm)
Non-Paretic (Asymm)
% BL Activation
200
150
100
50
0
BL
EE
LE
EPE
LPE
PHS
PTO
PHS
NHS
NTO
NHS
Time

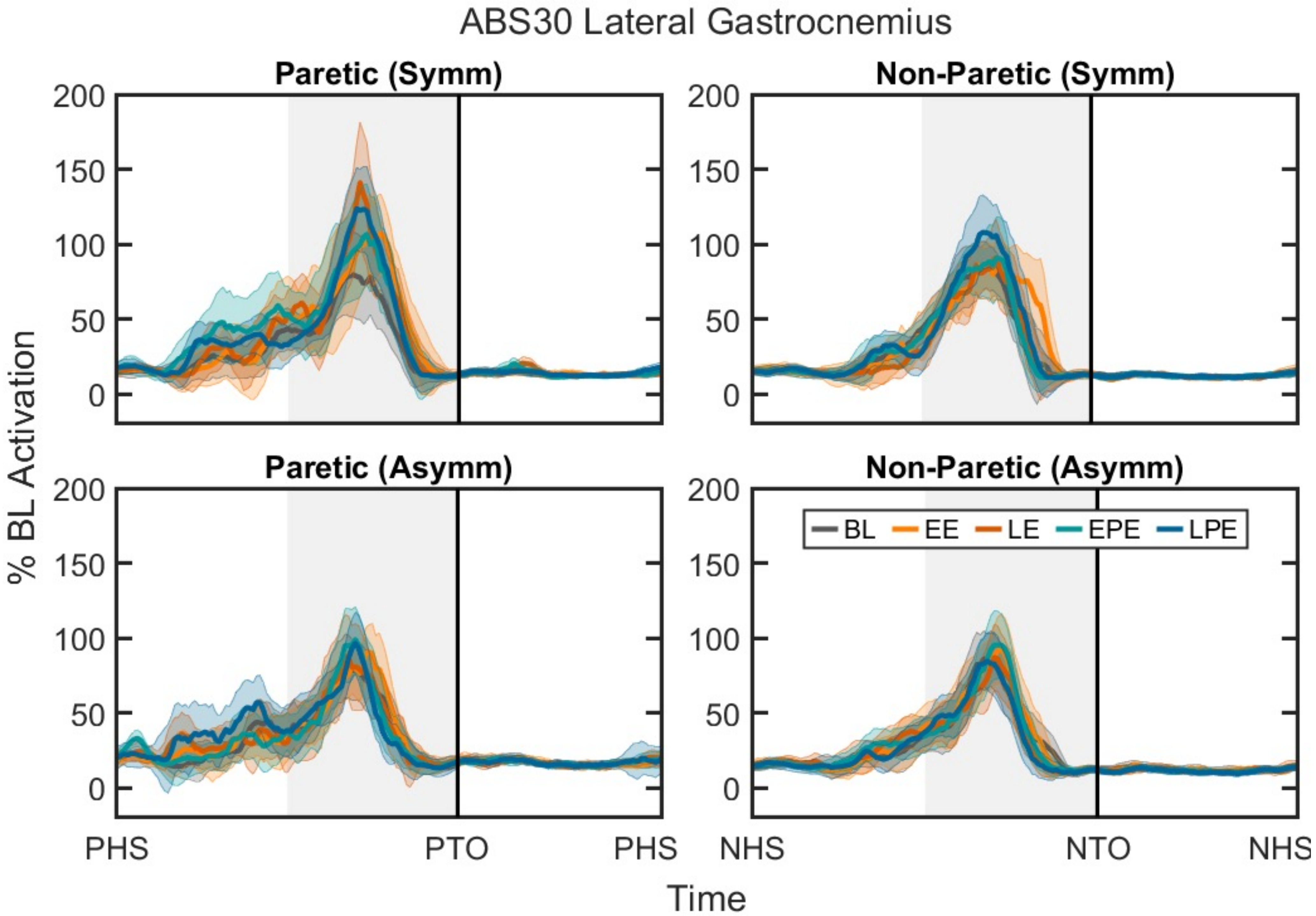

ABS30 Lateral Gastrocnemius
Paretic (Symm)
Non-Paretic (Symm)
Paretic (Asymm)
Non-Paretic (Asymm)
% BL Activation
200
150
100
50
0
BL
EE
LE
EPE
LPE
PHS
PTO
PHS
NHS
NTO
NHS
Time

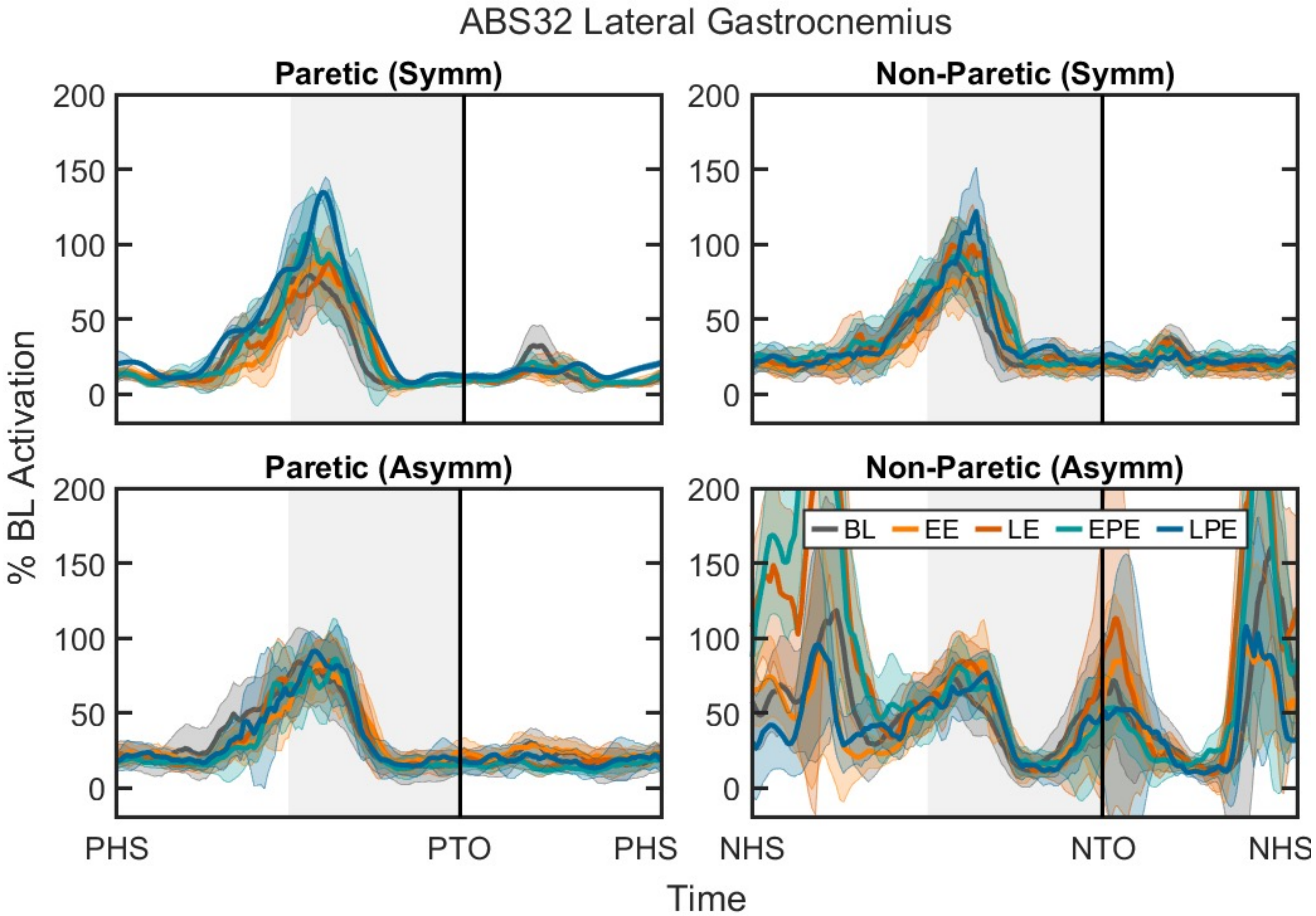
ABS32 Lateral Gastrocnemius
Paretic (Symm)
Non-Paretic (Symm)
Paretic (Asymm)
Non-Paretic (Asymm)
% BL Activation
200
150
100
50
0
BL
EE
LE
EPE
LPE
PHS
PTO
PHS
NHS
NTO
NHS
Time

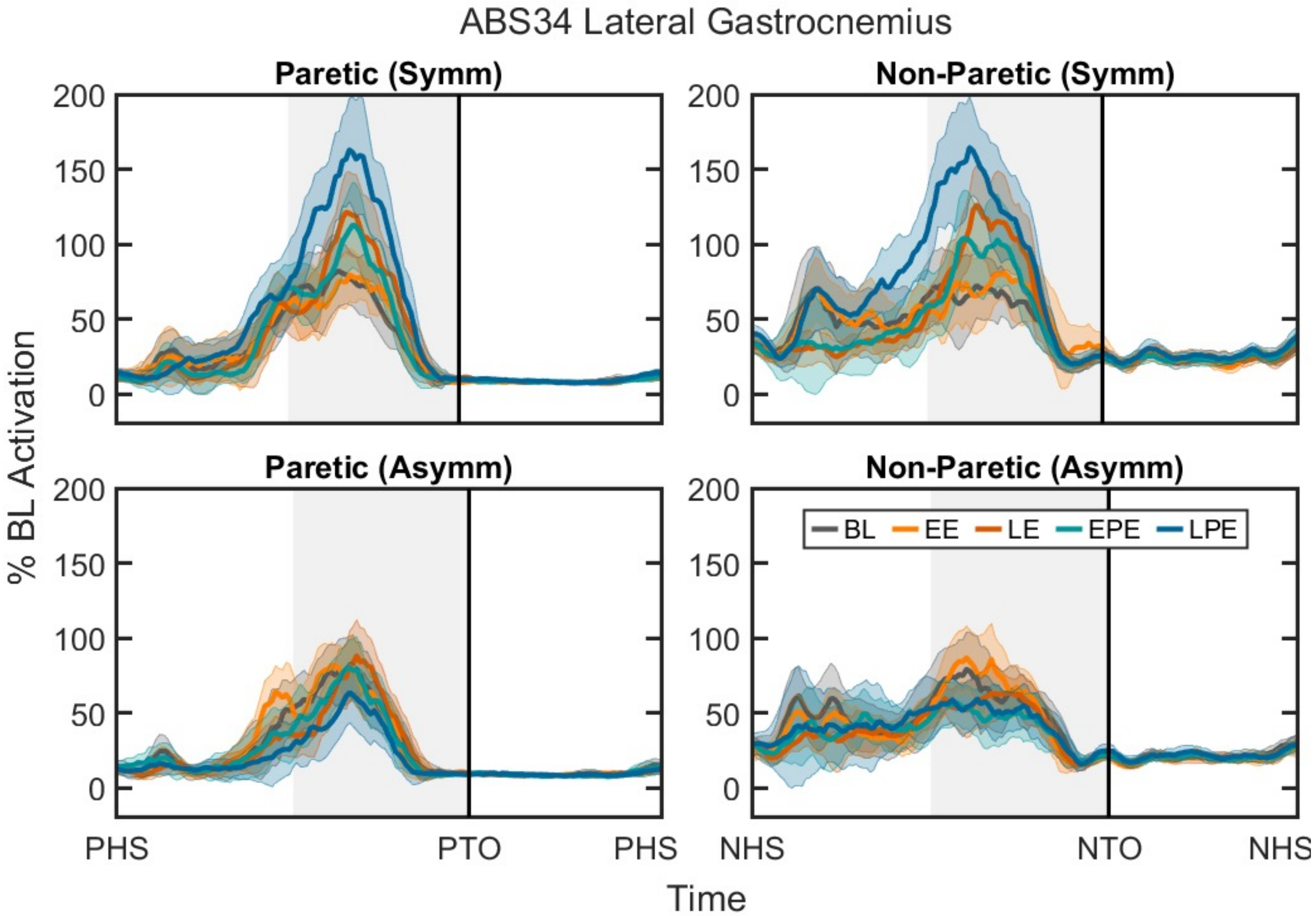
ABS34 Lateral Gastrocnemius
Paretic (Symm)
Non-Paretic (Symm)
Paretic (Asymm)
Non-Paretic (Asymm)
% BL Activation
200
150
100
50
0
BL
EE
LE
EPE
LPE
PHS
PTO
PHS
NHS
NTO
NHS
Time

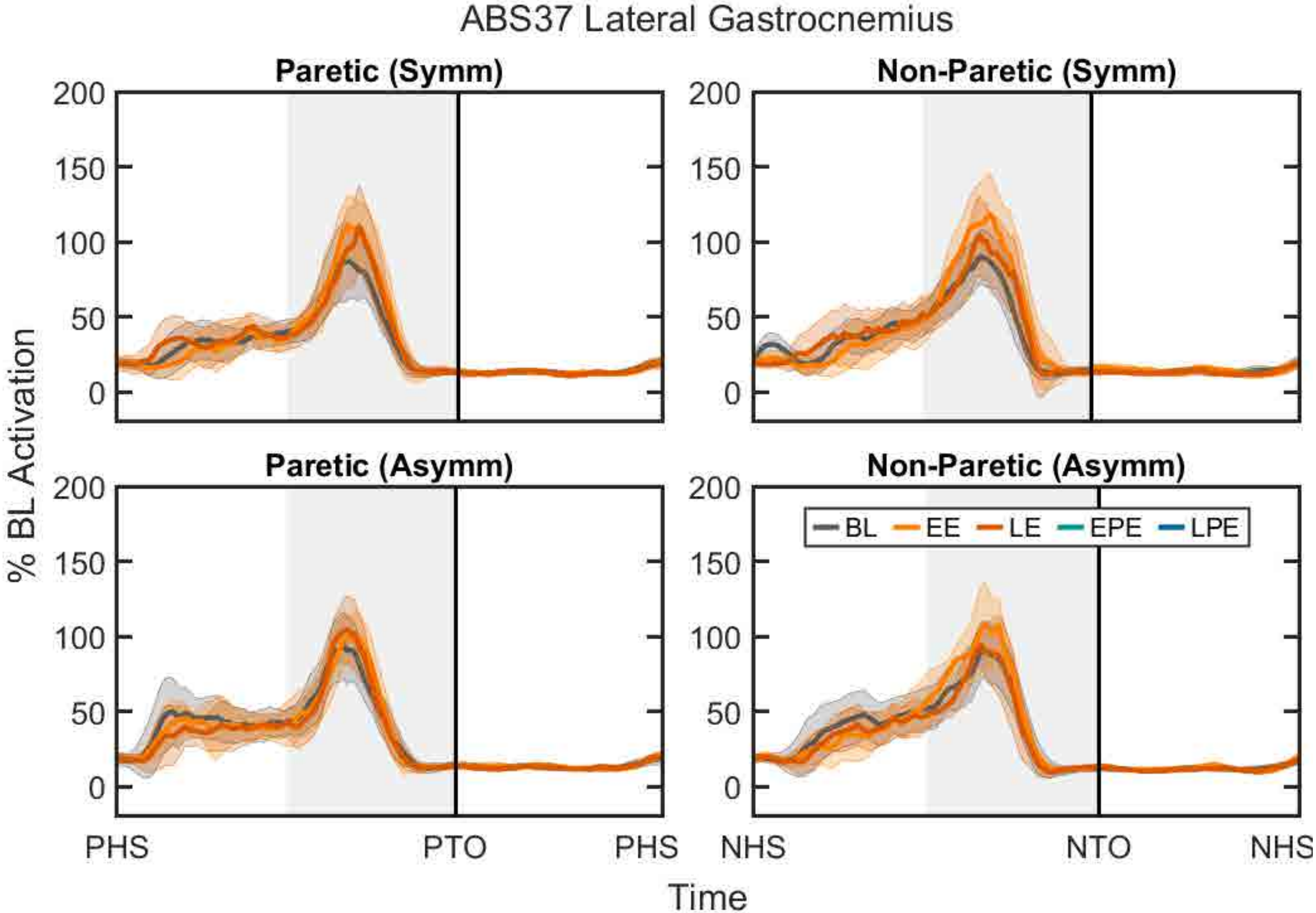
ABS37 Lateral Gastrocnemius
Paretic (Symm)
Non-Paretic (Symm)
Paretic (Asymm)
Non-Paretic (Asymm)
% BL Activation
200
150
100
50
0
BL
EE
LE
EPE
LPE
PHS
PTO
PHS
NHS
NTO
NHS
Time

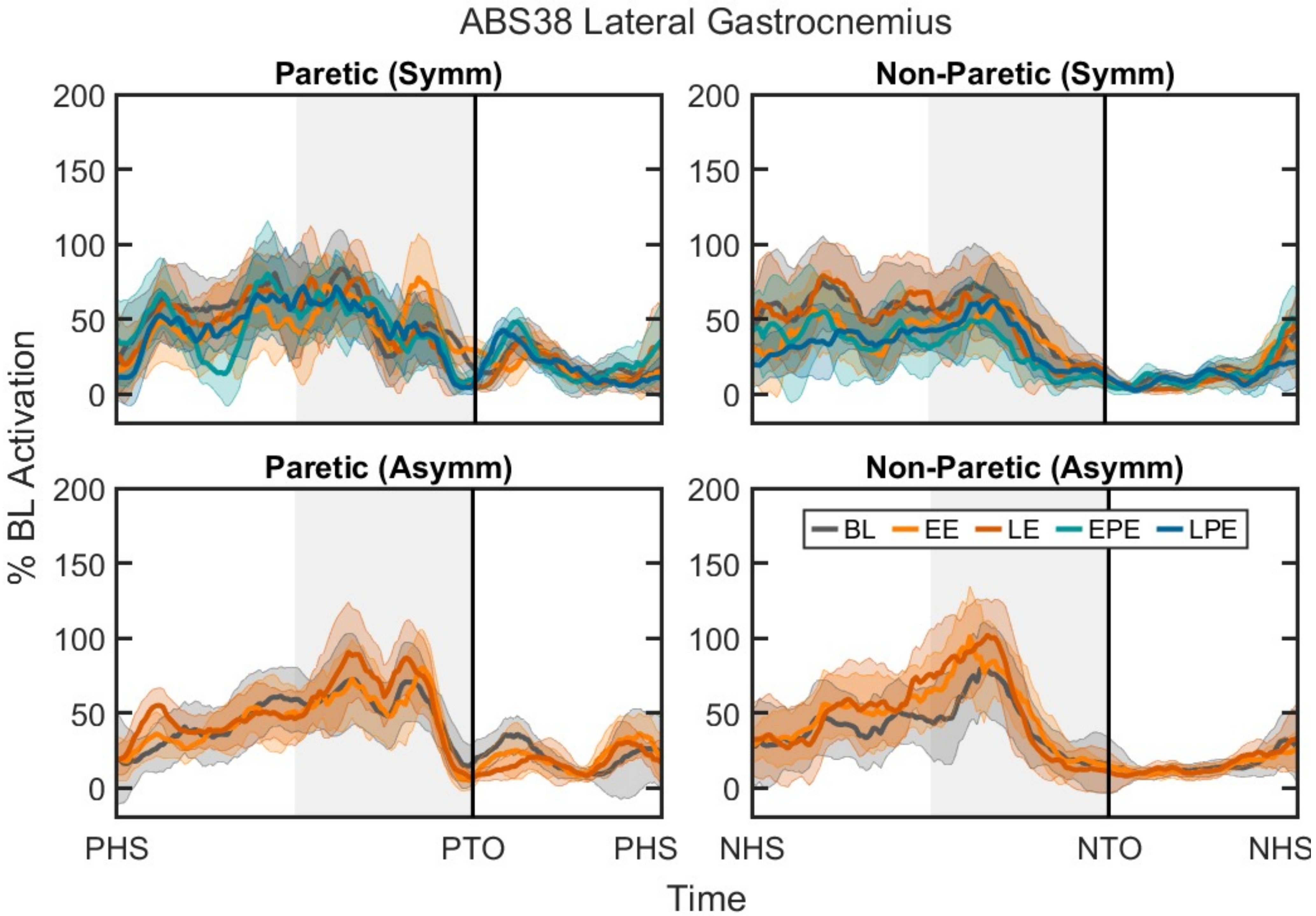
ABS38 Lateral Gastrocnemius
Paretic (Symm)
Non-Paretic (Symm)
Paretic (Asymm)
Non-Paretic (Asymm)
% BL Activation
200
150
100
50
0
BL
EE
LE
EPE
LPE
PHS
PTO
PHS
NHS
NTO
NHS
Time

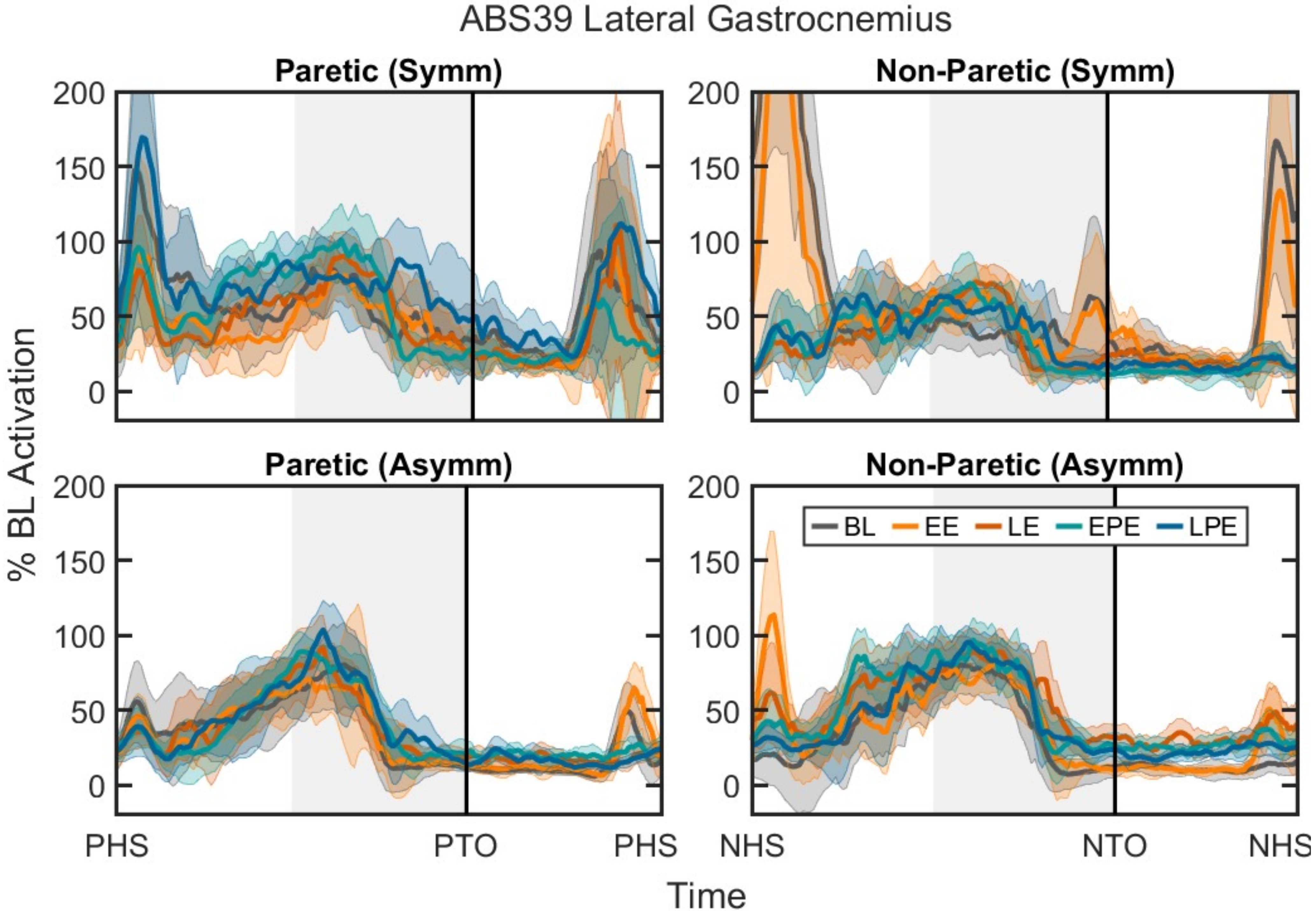
ABS39 Lateral Gastrocnemius
Paretic (Symm)
Non-Paretic (Symm)
Paretic (Asymm)
Non-Paretic (Asymm)
% BL Activation
200
150
100
50
0
BL
EE
LE
EPE
LPE
PHS
PTO
PHS
NHS
NTO
NHS
Time

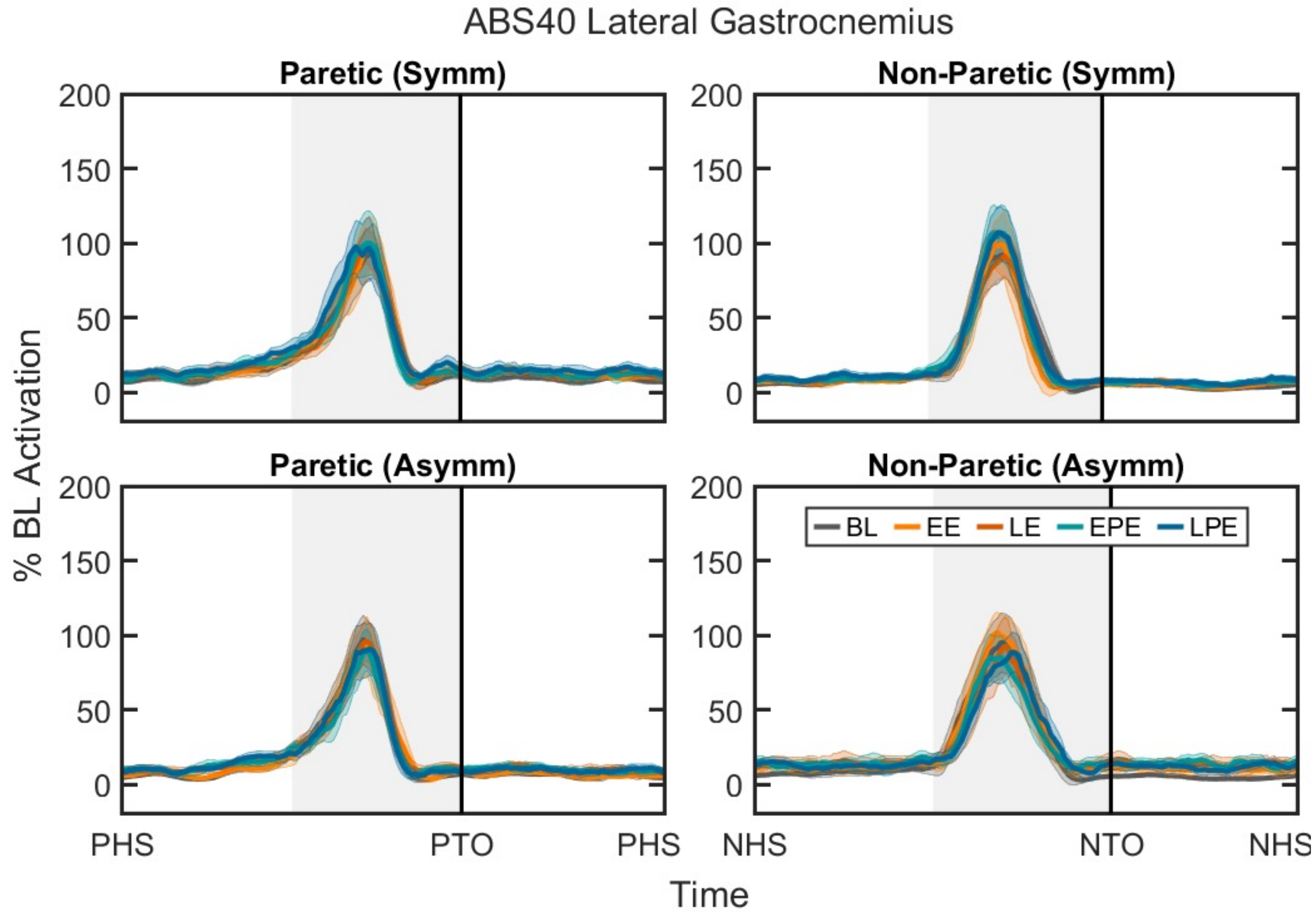
ABS40 Lateral Gastrocnemius
Paretic (Symm)
Non-Paretic (Symm)
Paretic (Asymm)
Non-Paretic (Asymm)
% BL Activation
200
150
100
50
0
BL
EE
LE
EPE
LPE
PHS
PTO
PHS
NHS
NTO
NHS
Time

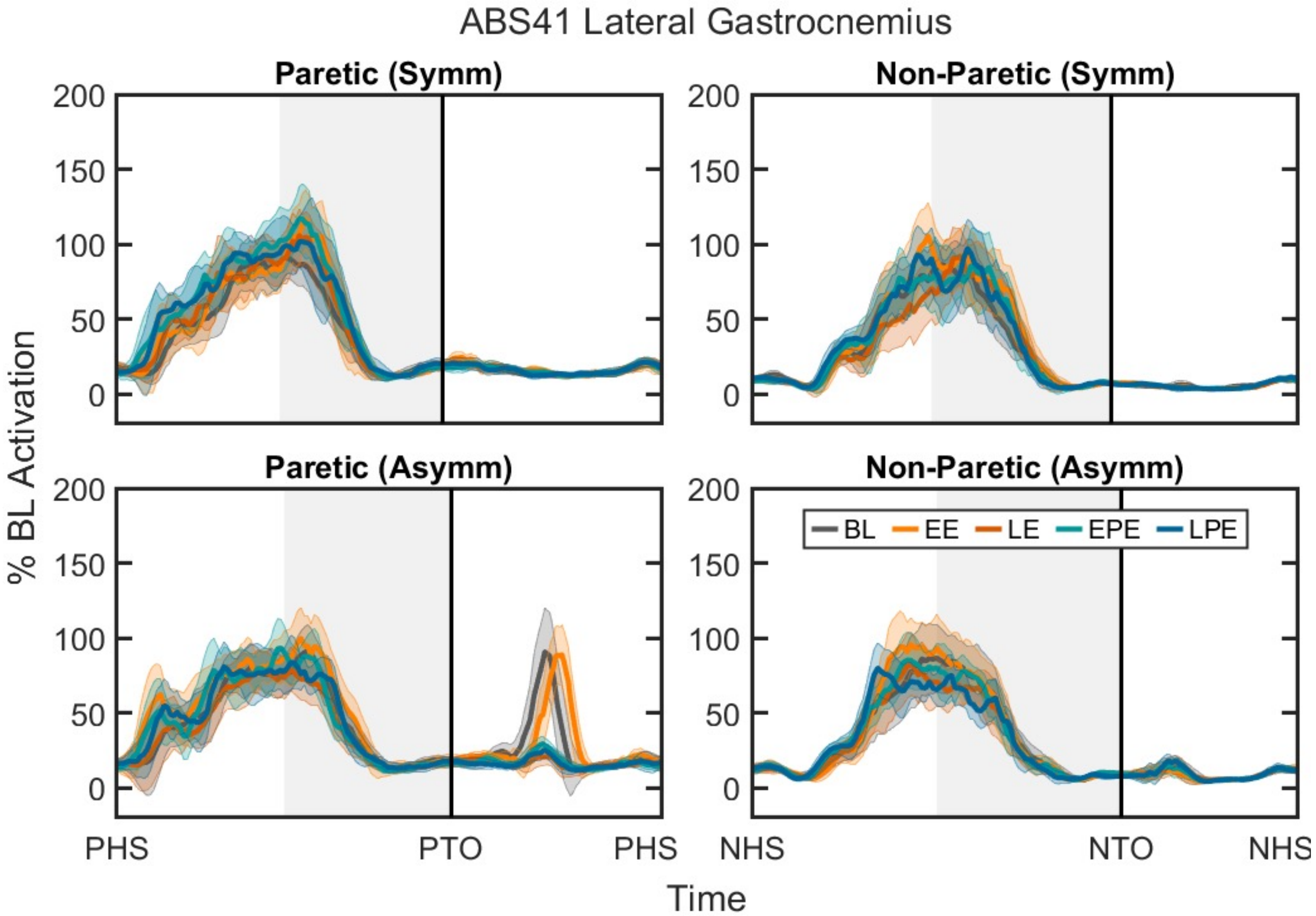
ABS41 Lateral Gastrocnemius
Paretic (Symm)
Non-Paretic (Symm)
Paretic (Asymm)
Non-Paretic (Asymm)
% BL Activation
200
150
100
50
0
BL
EE
LE
EPE
LPE
PHS
PTO
PHS
NHS
NTO
NHS
Time

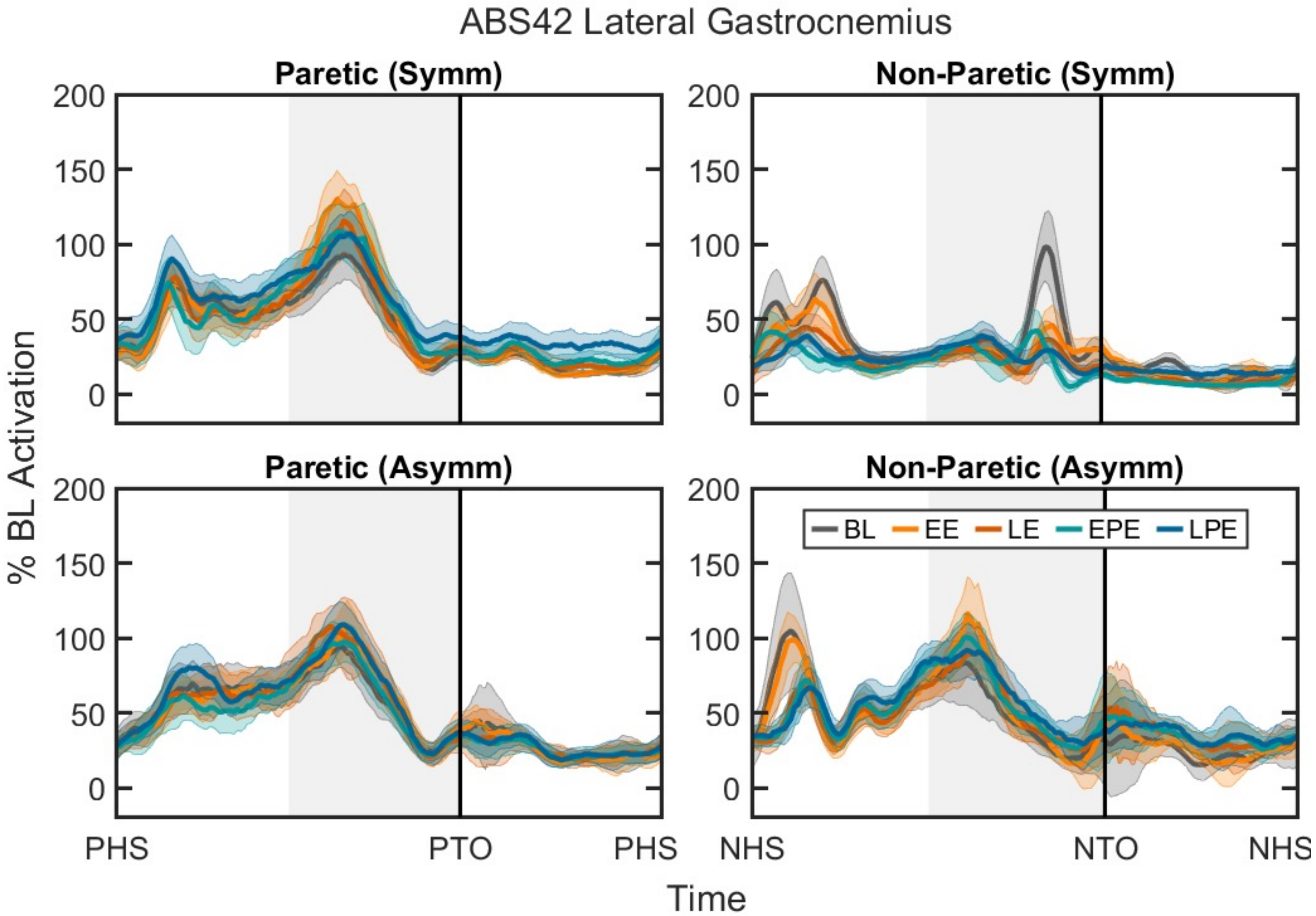
ABS42 Lateral Gastrocnemius
Paretic (Symm)
Non-Paretic (Symm)
Paretic (Asymm)
Non-Paretic (Asymm)
% BL Activation
200
150
100
50
0
BL
EE
LE
EPE
LPE
PHS
PTO
PHS
NHS
NTO
NHS
Time

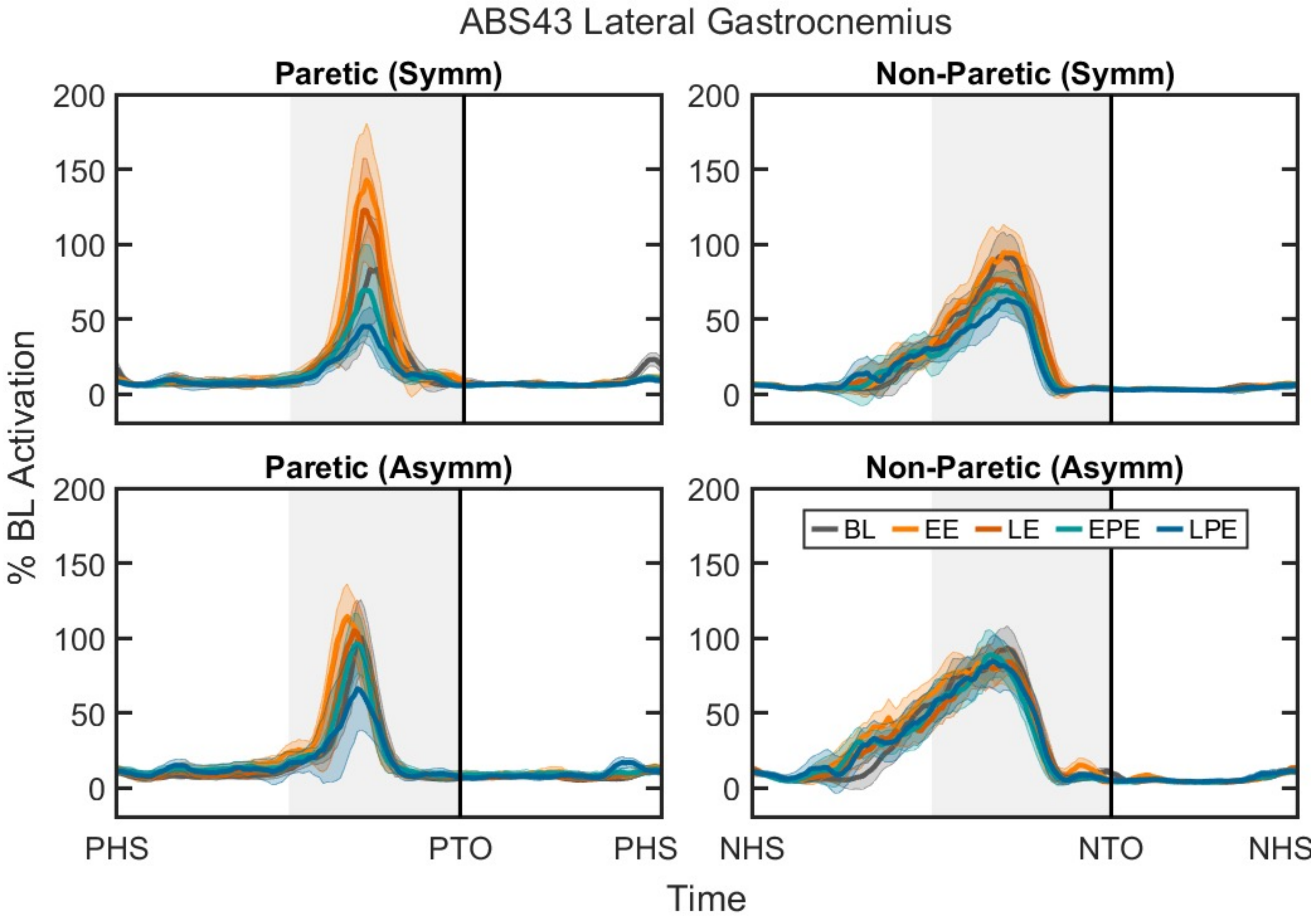
ABS43 Lateral Gastrocnemius
Paretic (Symm)
Non-Paretic (Symm)
Paretic (Asymm)
Non-Paretic (Asymm)
% BL Activation
200
150
100
50
0
BL
EE
LE
EPE
LPE
PHS
PTO
PHS
NHS
NTO
NHS
Time

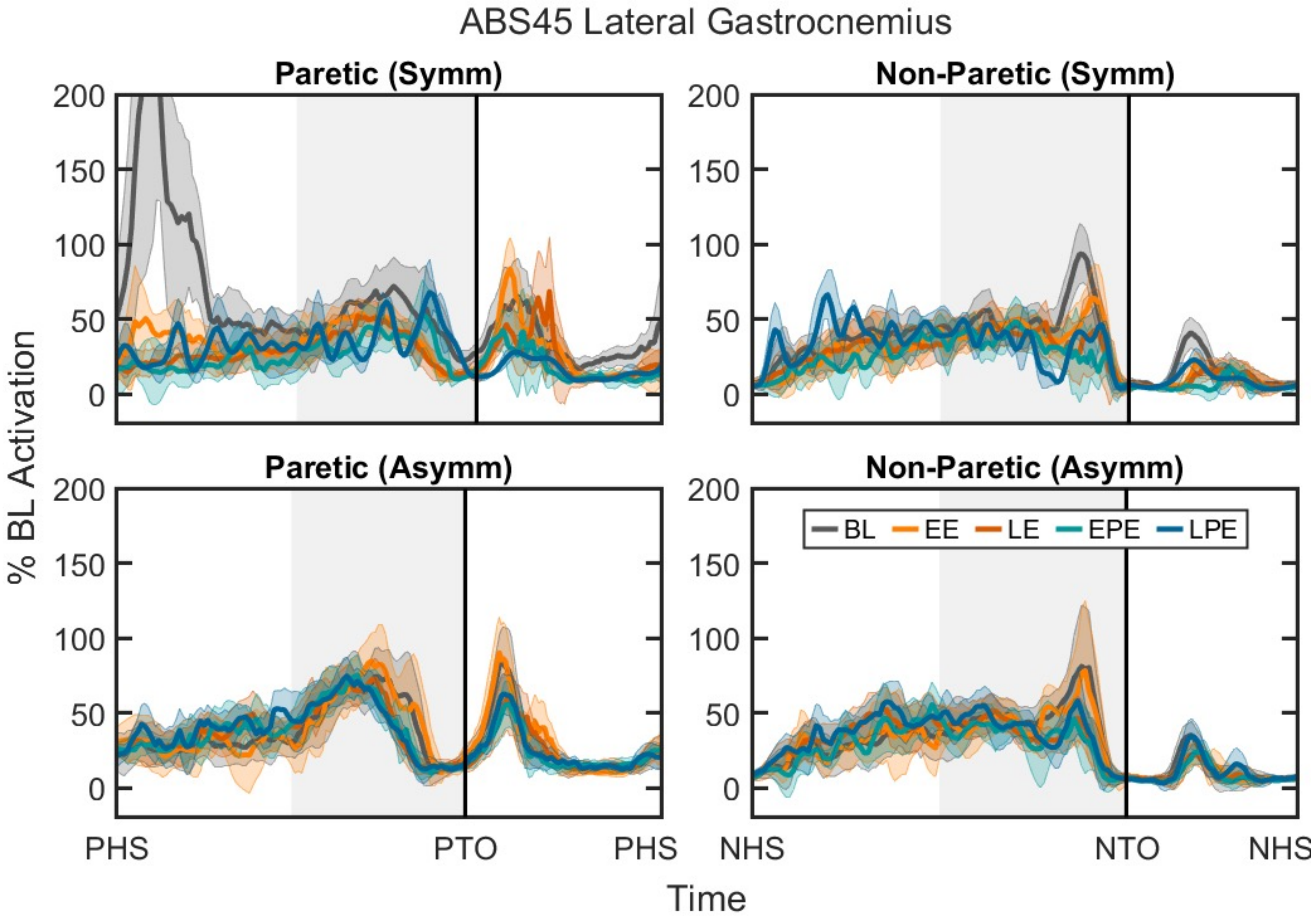

ABS45 Lateral Gastrocnemius
Paretic (Symm)
Non-Paretic (Symm)
Paretic (Asymm)
Non-Paretic (Asymm)
% BL Activation
200
150
100
50
0
BL
EE
LE
EPE
LPE
PHS
PTO
PHS
NHS
NTO
NHS
Time

Medial Gastrocnemius

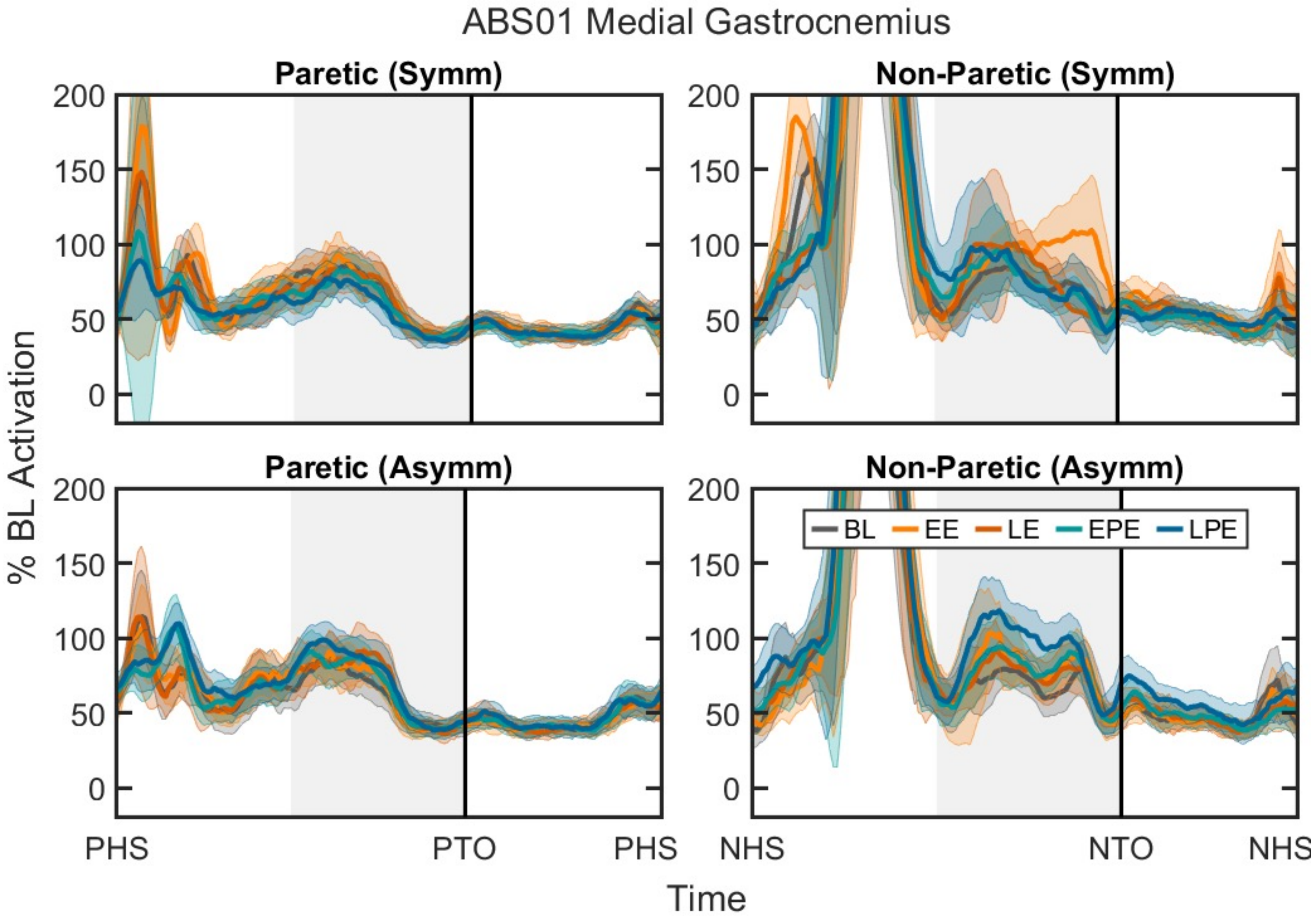
ABS01 Medial Gastrocnemius
Paretic (Symm)
Non-Paretic (Symm)
Paretic (Asymm)
Non-Paretic (Asymm)
% BL Activation
200
150
100
50
0
BL
EE
LE
EPE
LPE
PHS
PTO
PHS
NHS
NTO
NHS
Time

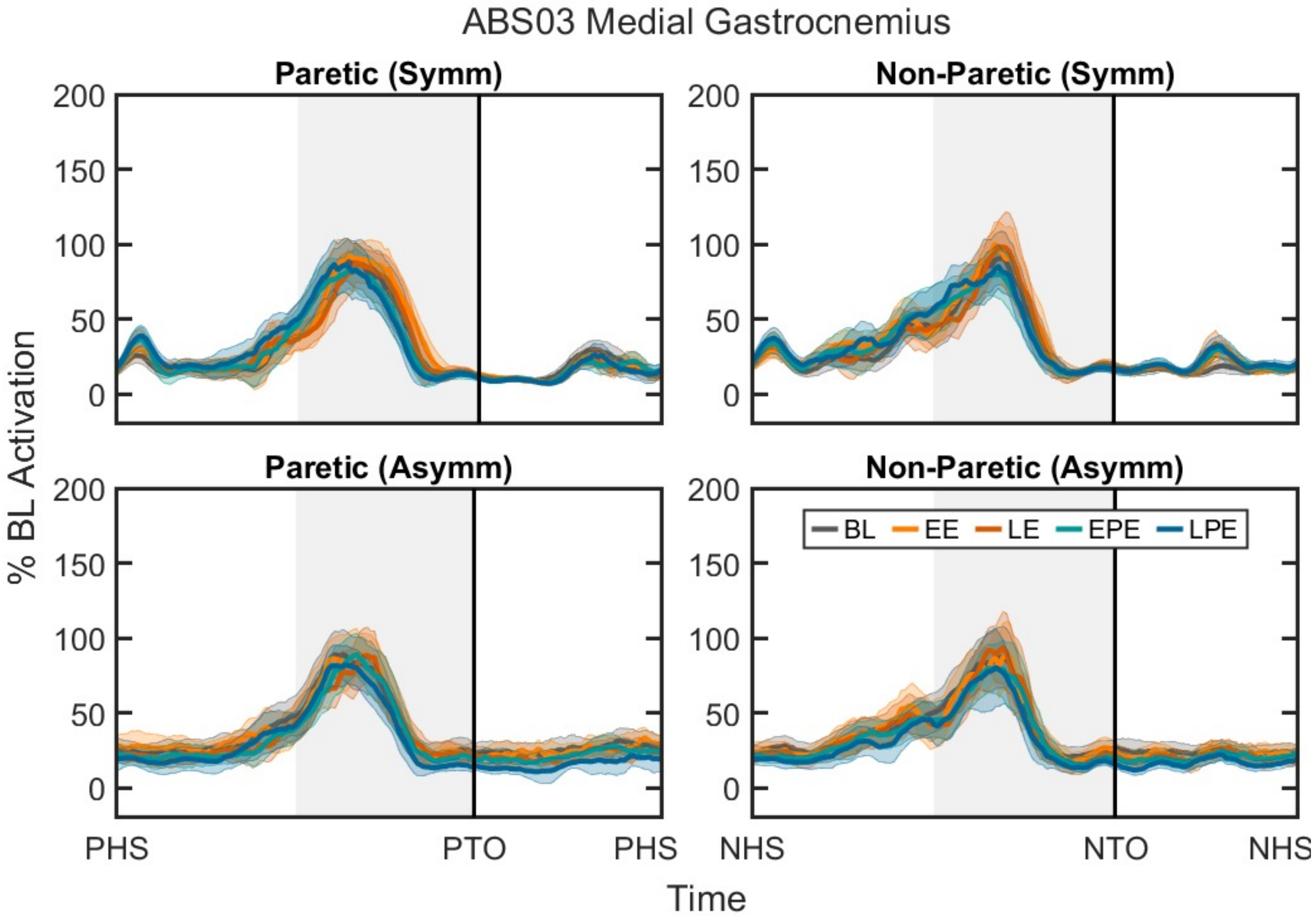
ABS03 Medial Gastrocnemius
Paretic (Symm)
Non-Paretic (Symm)
Paretic (Asymm)
Non-Paretic (Asymm)
% BL Activation
200
150
100
50
0
BL
EE
LE
EPE
LPE
PHS
PTO
PHS
NHS
NTO
NHS
Time

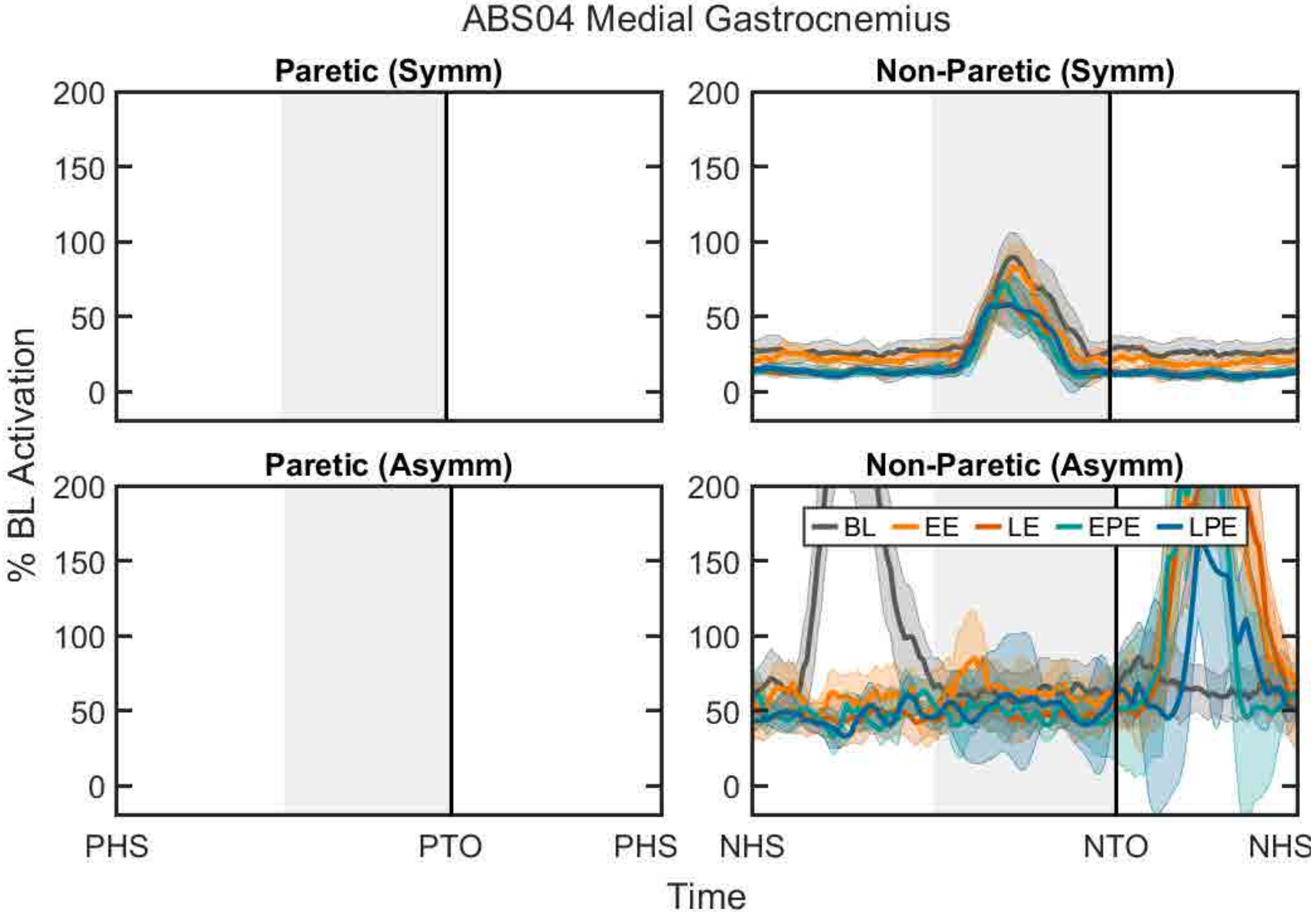
ABS04 Medial Gastrocnemius
Paretic (Symm)
Non-Paretic (Symm)
Paretic (Asymm)
Non-Paretic (Asymm)
% BL Activation
200
150
100
50
0
BL
EE
LE
EPE
LPE
PHS
PTO
PHS
NHS
NTO
NHS
Time

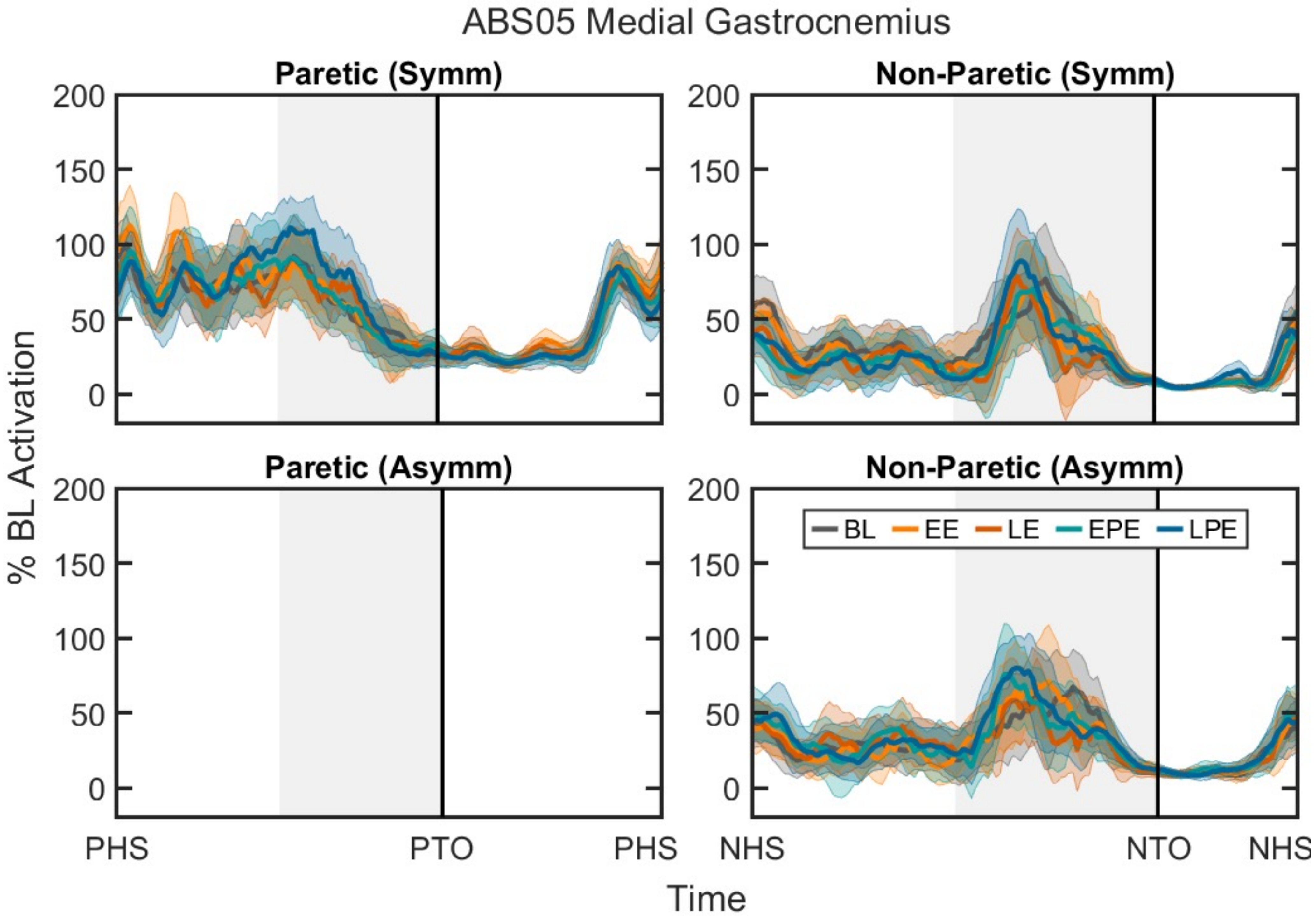
ABS05 Medial Gastrocnemius
Paretic (Symm)
Non-Paretic (Symm)
Paretic (Asymm)
Non-Paretic (Asymm)
% BL Activation
200
150
100
50
0
BL
EE
LE
EPE
LPE
PHS
PTO
PHS
NHS
NTO
NHS
Time

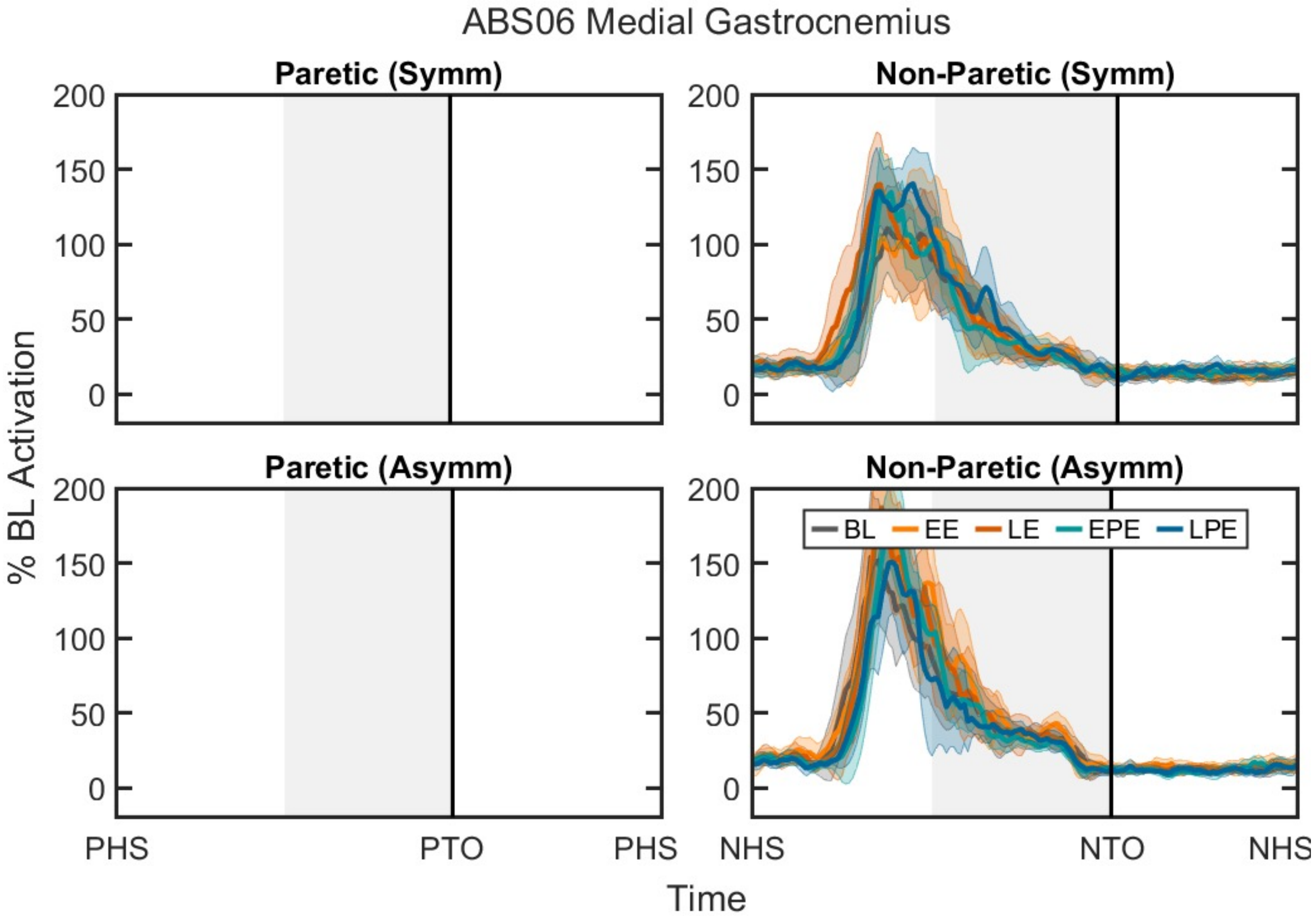

ABS06 Medial Gastrocnemius
Paretic (Symm)
Non-Paretic (Symm)
Paretic (Asymm)
Non-Paretic (Asymm)
% BL Activation
200
150
100
50
0
BL
EE
LE
EPE
LPE
PHS
PTO
PHS
NHS
NTO
NHS
Time

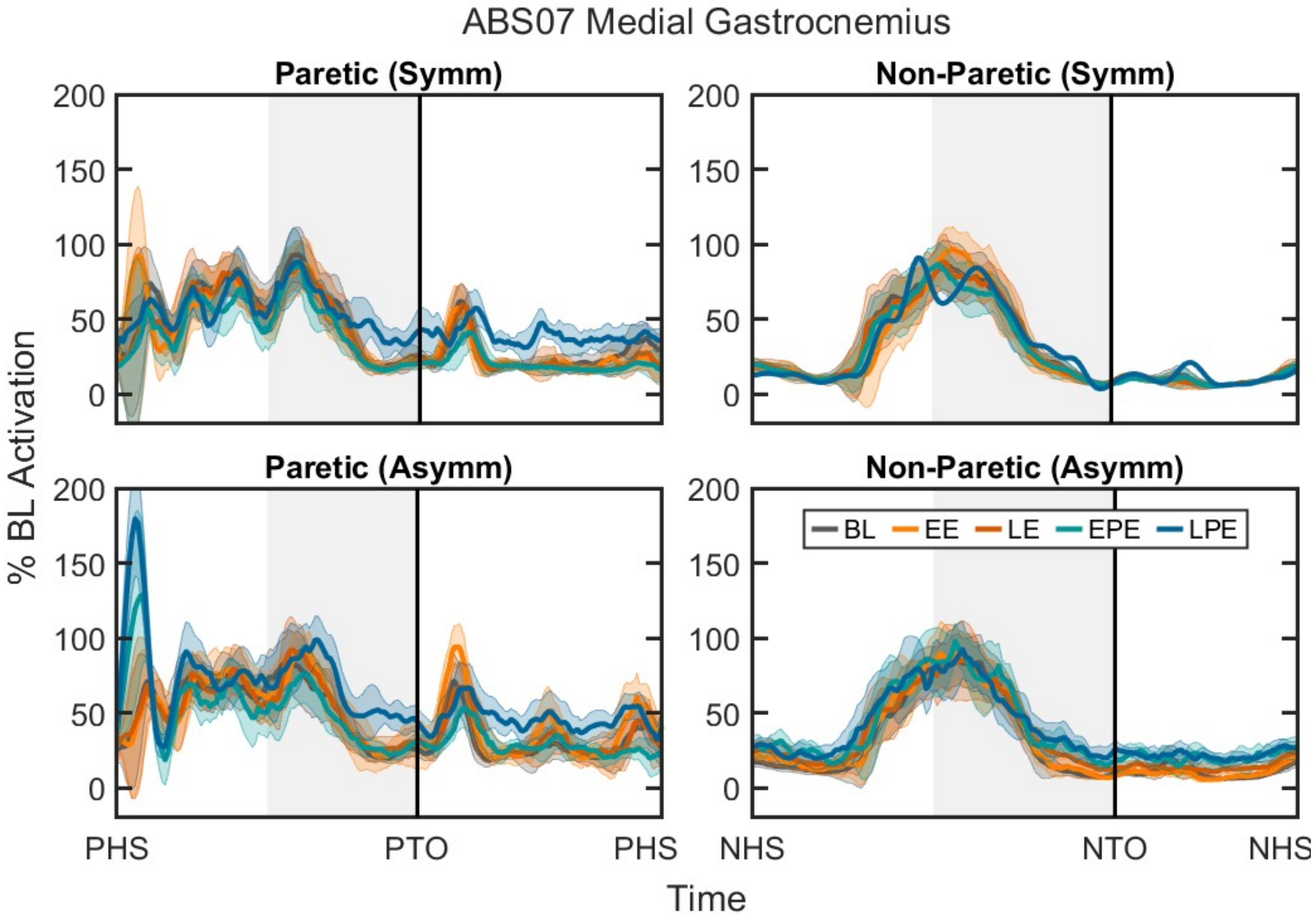
ABS07 Medial Gastrocnemius
Paretic (Symm)
Non-Paretic (Symm)
Paretic (Asymm)
Non-Paretic (Asymm)
% BL Activation
200
150
100
50
0
BL
EE
LE
EPE
LPE
PHS
PTO
PHS
NHS
NTO
NHS
Time

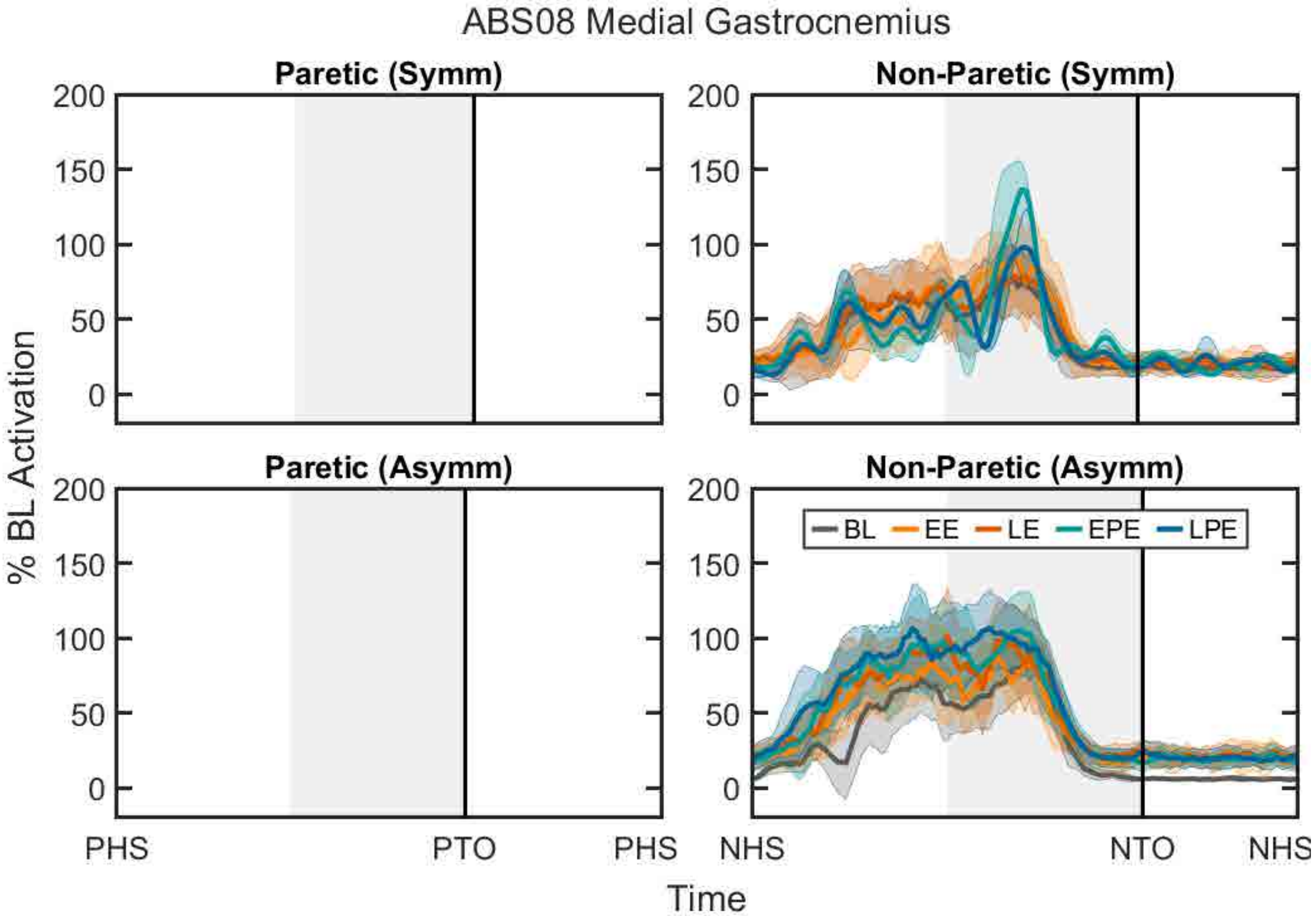

ABS08 Medial Gastrocnemius
Paretic (Symm)
Non-Paretic (Symm)
Paretic (Asymm)
Non-Paretic (Asymm)
% BL Activation
200
150
100
50
0
BL
EE
LE
EPE
LPE
PHS
PTO
PHS
NHS
NTO
NHS
Time

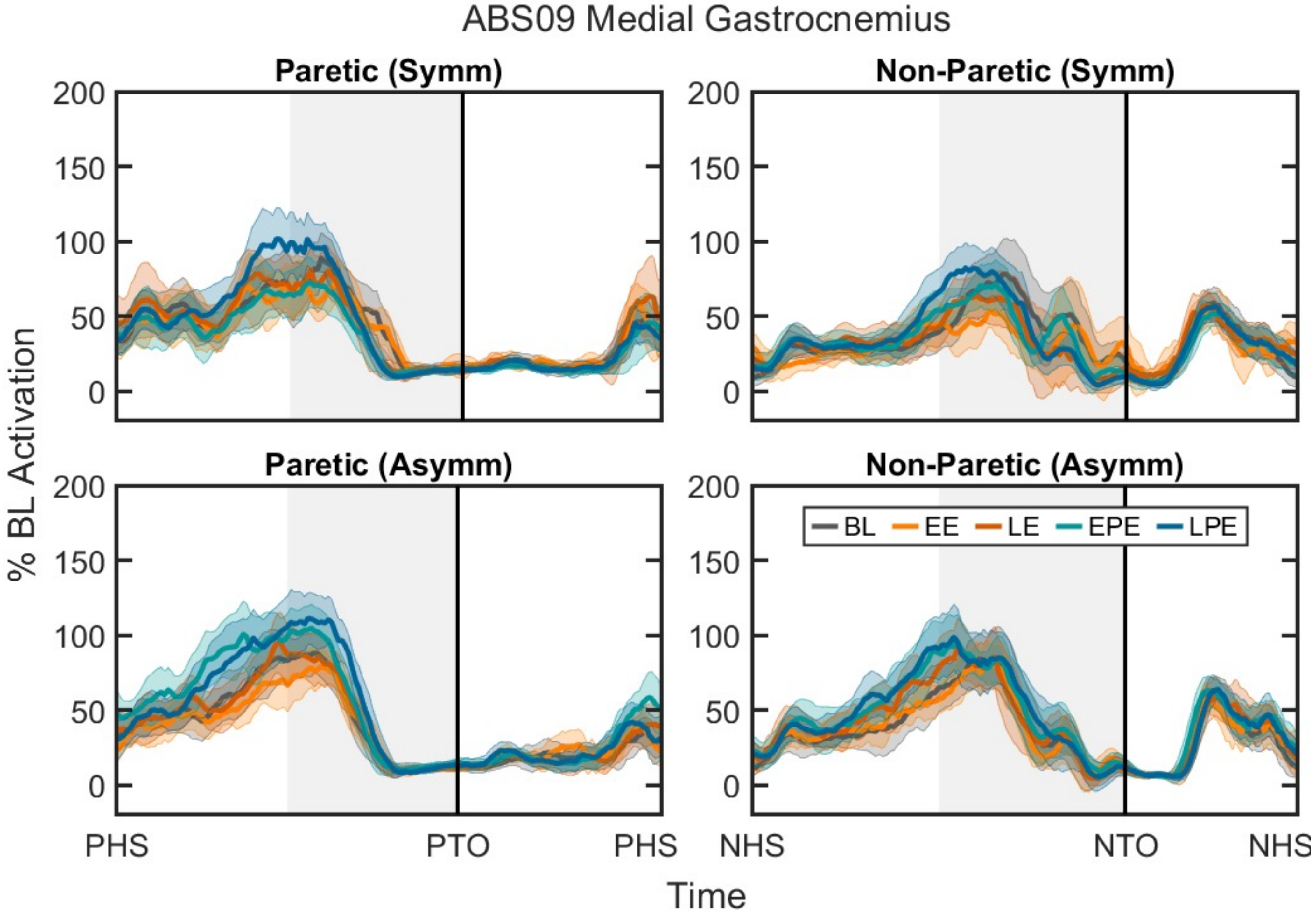

ABS09 Medial Gastrocnemius
Paretic (Symm)
Non-Paretic (Symm)
Paretic (Asymm)
Non-Paretic (Asymm)
% BL Activation
200
150
100
50
0
BL
EE
LE
EPE
LPE
PHS
PTO
PHS
NHS
NTO
NHS
Time

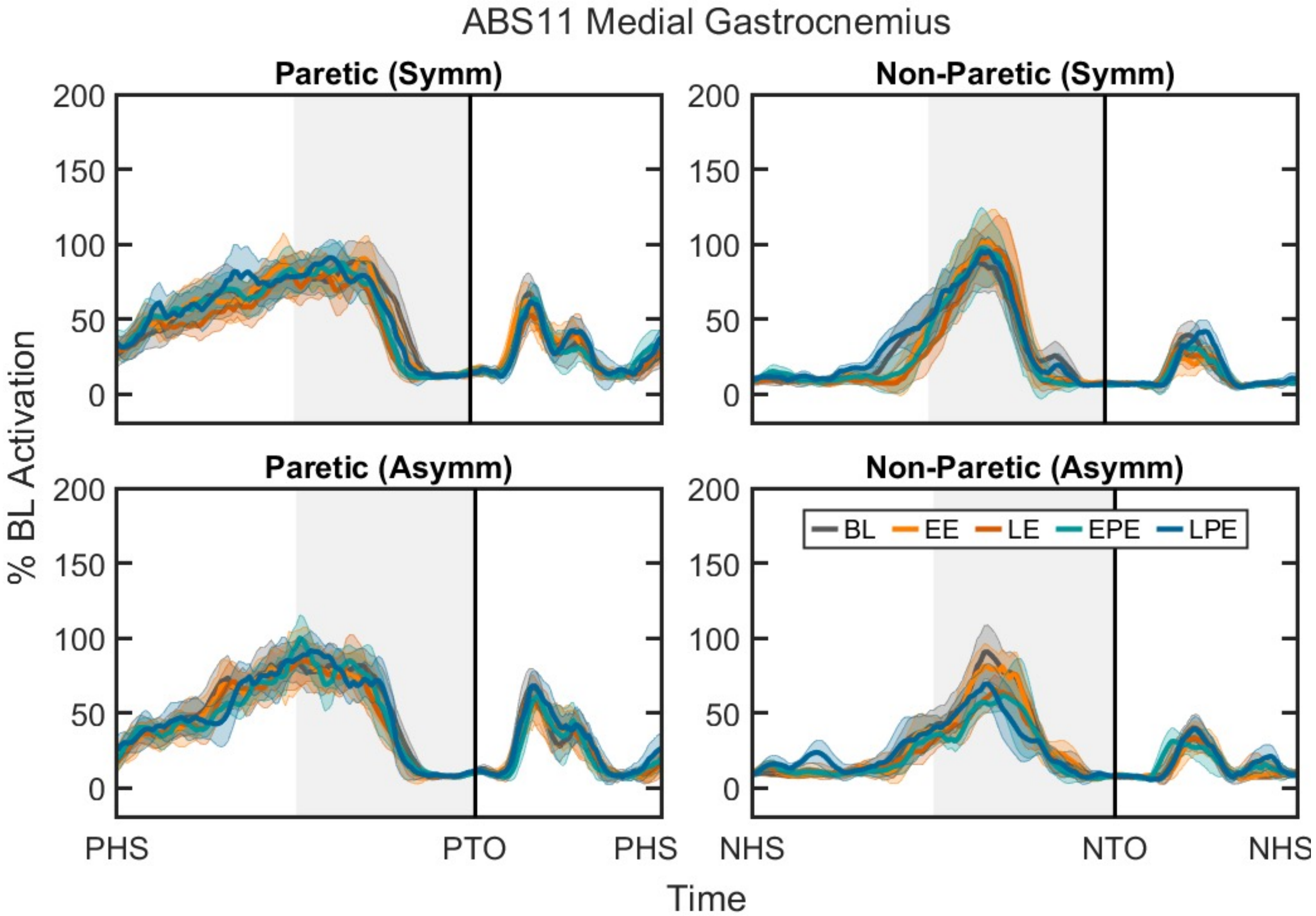

ABS11 Medial Gastrocnemius
Paretic (Symm)
Non-Paretic (Symm)
Paretic (Asymm)
Non-Paretic (Asymm)
% BL Activation
200
150
100
50
0
BL
EE
LE
EPE
LPE
PHS
PTO
PHS
NHS
NTO
NHS
Time

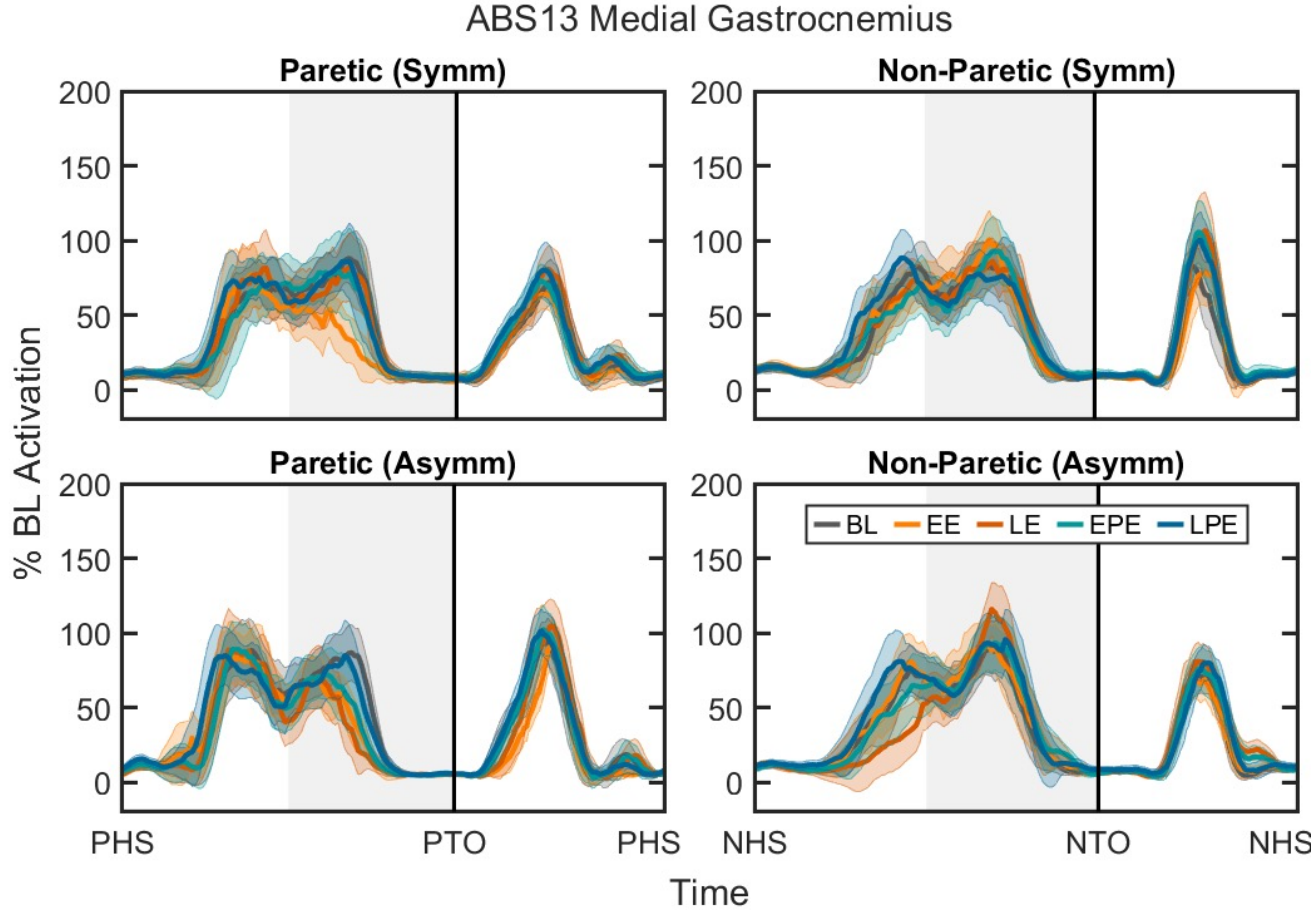
ABS13 Medial Gastrocnemius
Paretic (Symm)
Non-Paretic (Symm)
Paretic (Asymm)
Non-Paretic (Asymm)
% BL Activation
200
150
100
50
0
BL
EE
LE
EPE
LPE
PHS
PTO
PHS
NHS
NTO
NHS
Time

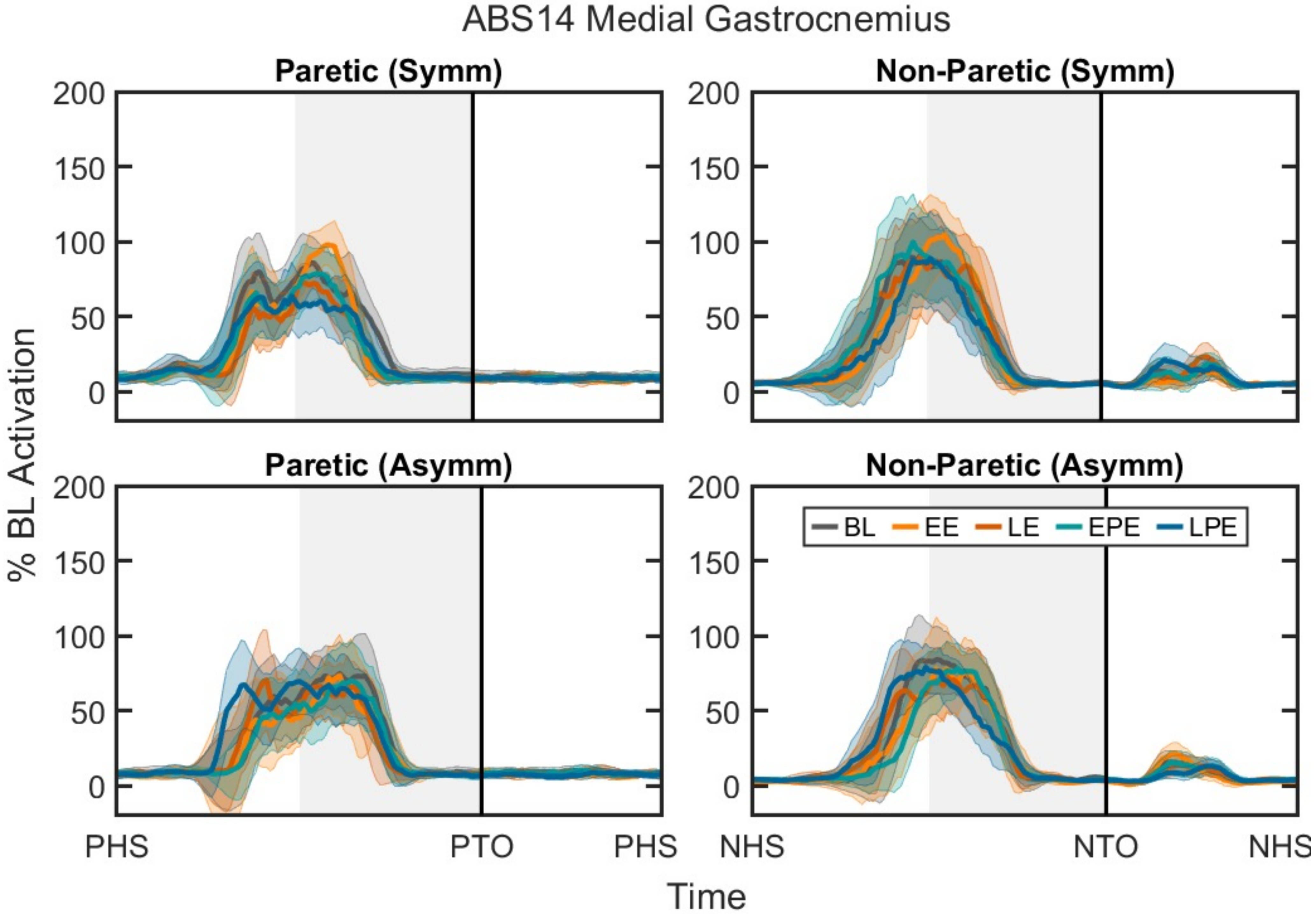
ABS14 Medial Gastrocnemius
Paretic (Symm)
Non-Paretic (Symm)
Paretic (Asymm)
Non-Paretic (Asymm)
% BL Activation
200
150
100
50
0
BL
EE
LE
EPE
LPE
PHS
PTO
PHS
NHS
NTO
NHS
Time

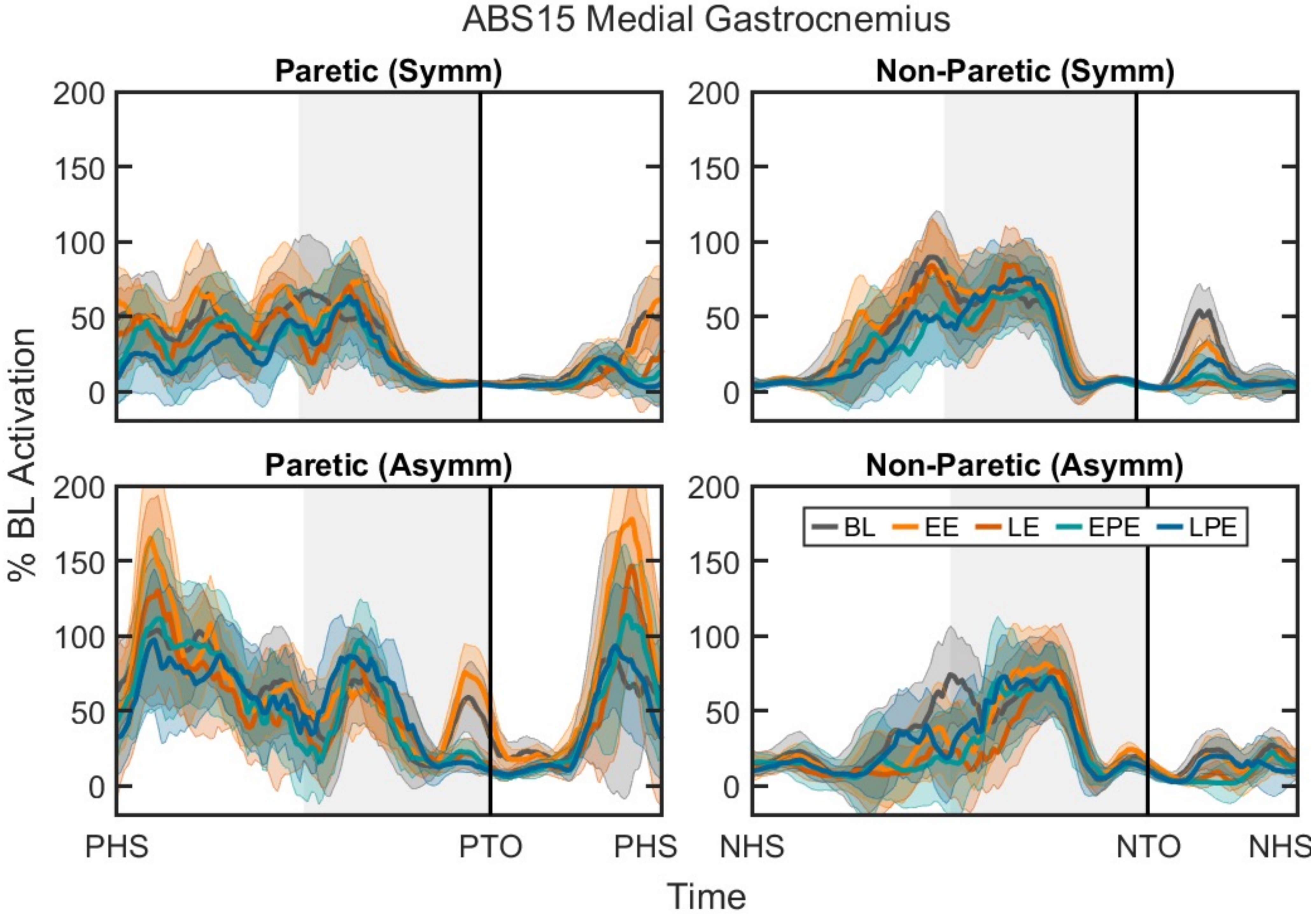

ABS15 Medial Gastrocnemius
Paretic (Symm)
Non-Paretic (Symm)
Paretic (Asymm)
Non-Paretic (Asymm)
% BL Activation
200
150
100
50
0
BL
EE
LE
EPE
LPE
PHS
PTO
PHS
NHS
NTO
NHS
Time

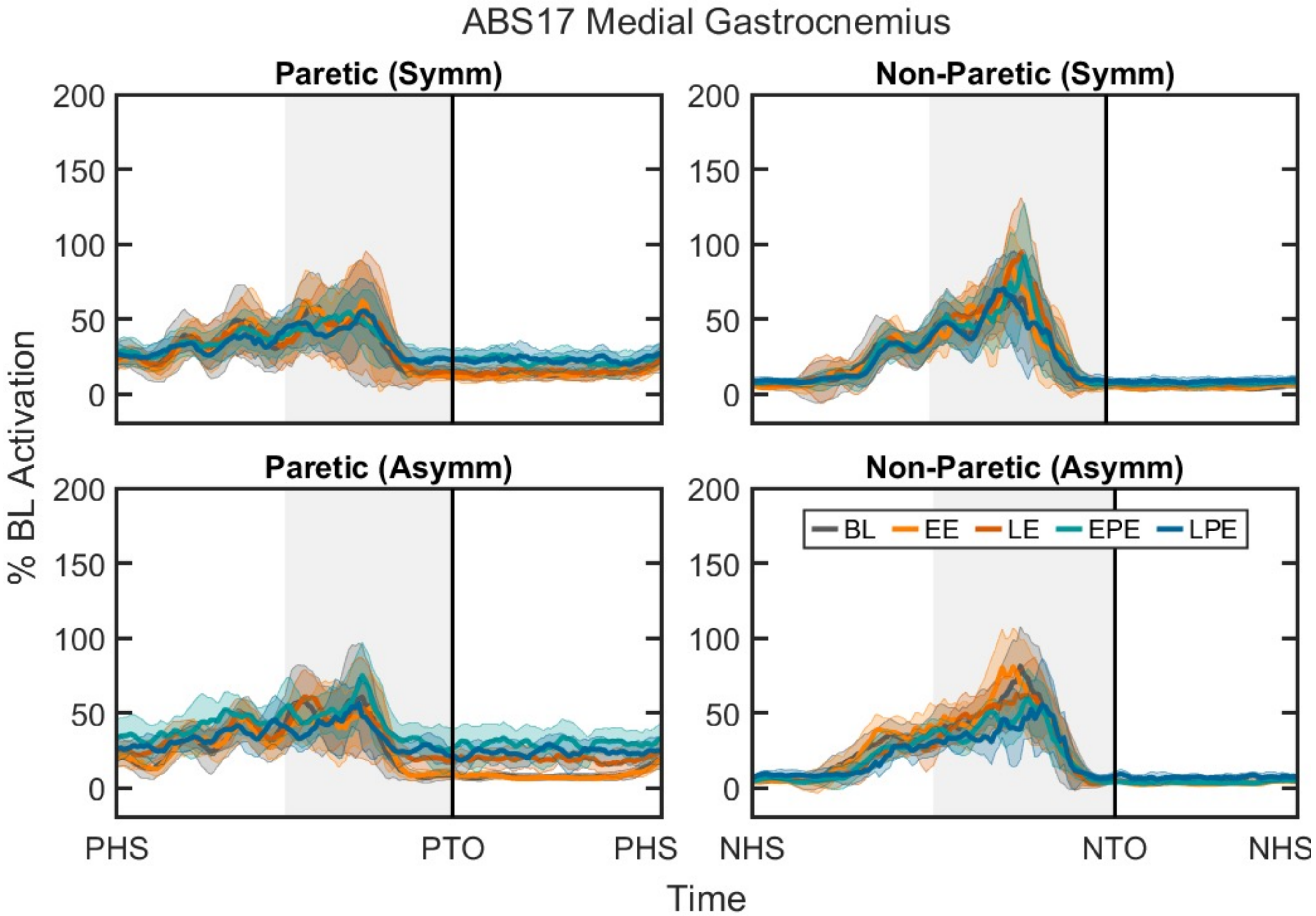

ABS17 Medial Gastrocnemius
Paretic (Symm)
Non-Paretic (Symm)
Paretic (Asymm)
Non-Paretic (Asymm)
% BL Activation
200
150
100
50
0
BL
EE
LE
EPE
LPE
PHS
PTO
PHS
NHS
NTO
NHS
Time

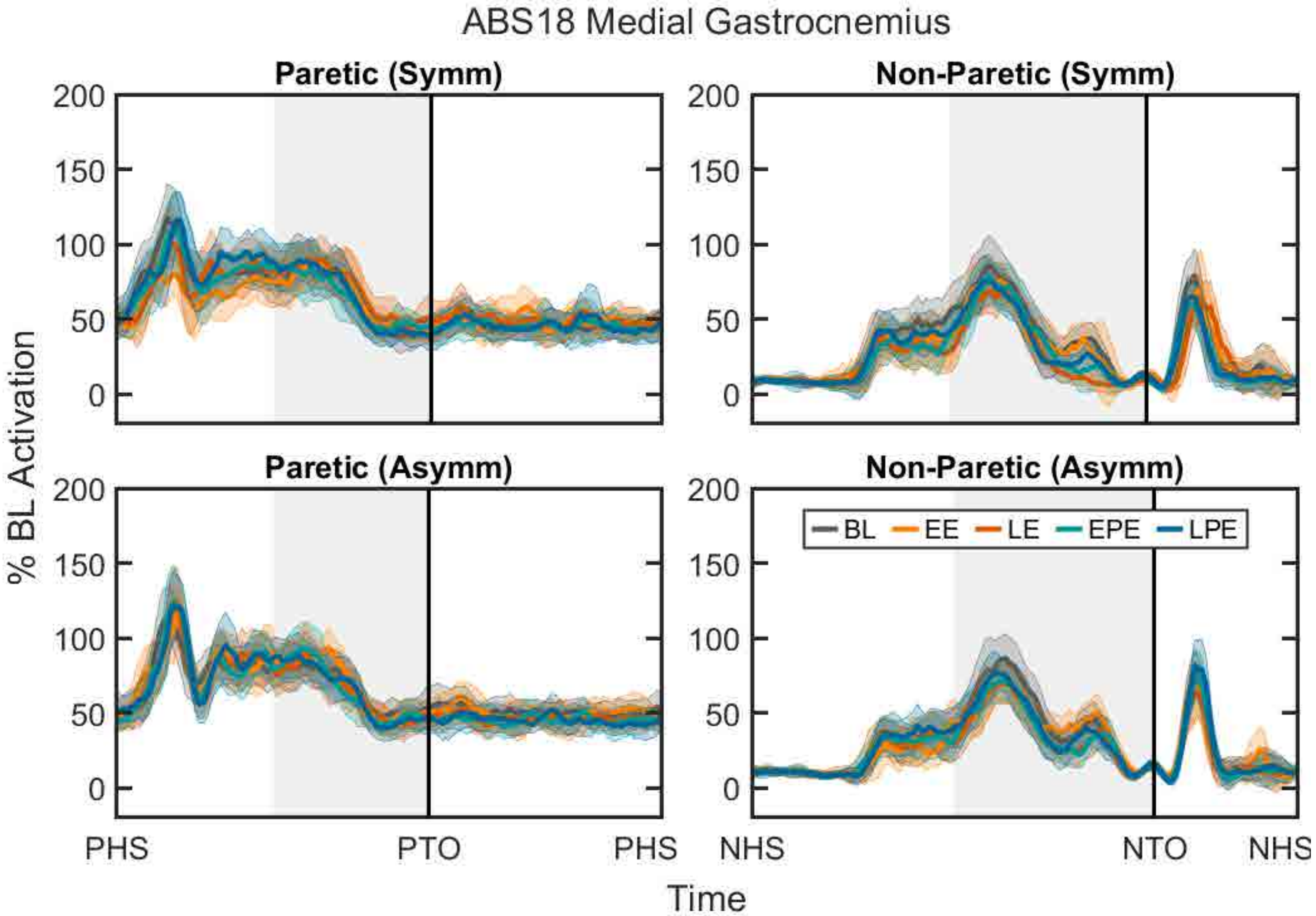
ABS18 Medial Gastrocnemius
Paretic (Symm)
Non-Paretic (Symm)
Paretic (Asymm)
Non-Paretic (Asymm)
% BL Activation
200
150
100
50
0
BL
EE
LE
EPE
LPE
PHS
PTO
PHS
NHS
NTO
NHS
Time

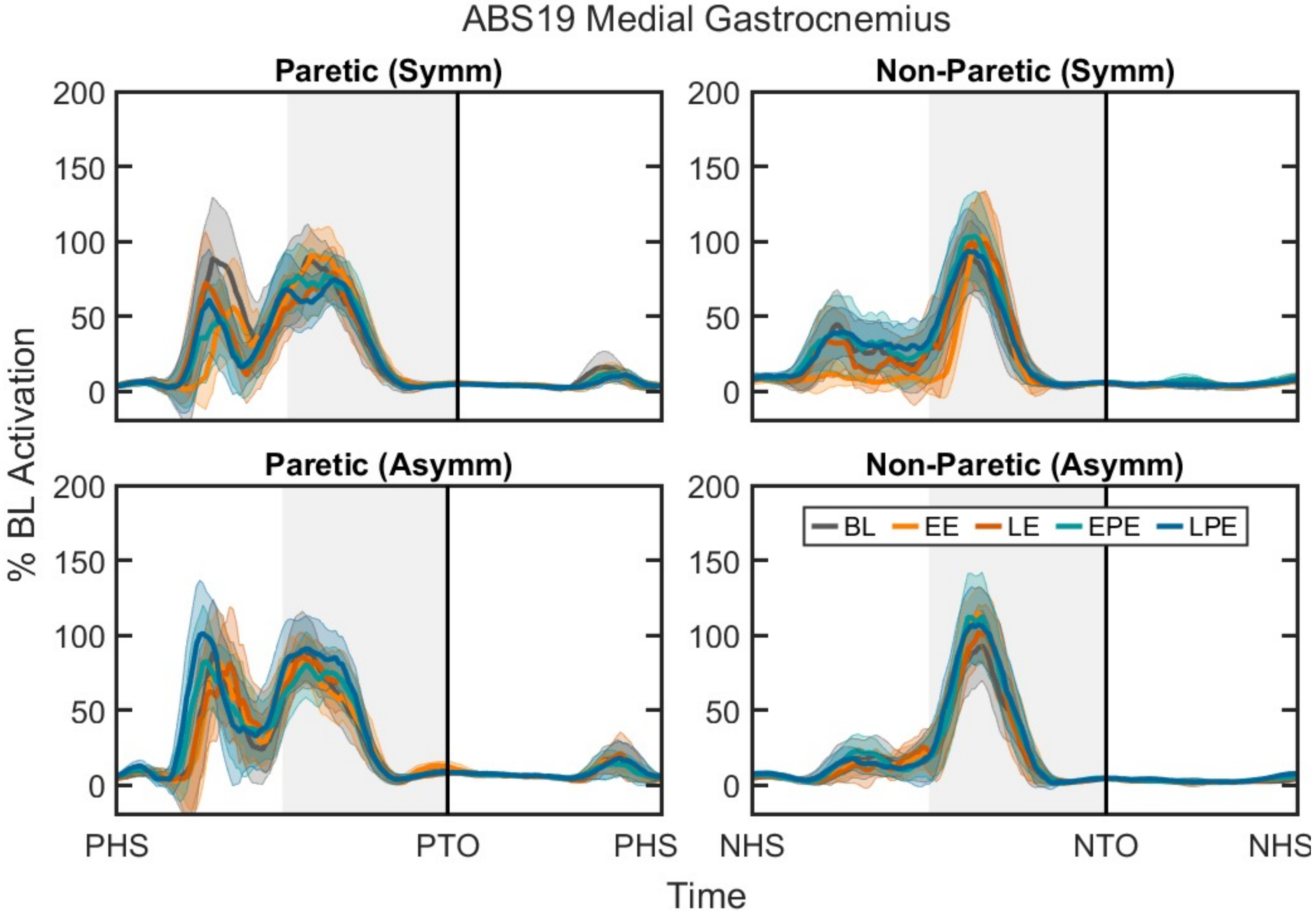
ABS19 Medial Gastrocnemius
Paretic (Symm)
Non-Paretic (Symm)
Paretic (Asymm)
Non-Paretic (Asymm)
% BL Activation
200
150
100
50
0
BL
EE
LE
EPE
LPE
PHS
PTO
PHS
NHS
NTO
NHS
Time

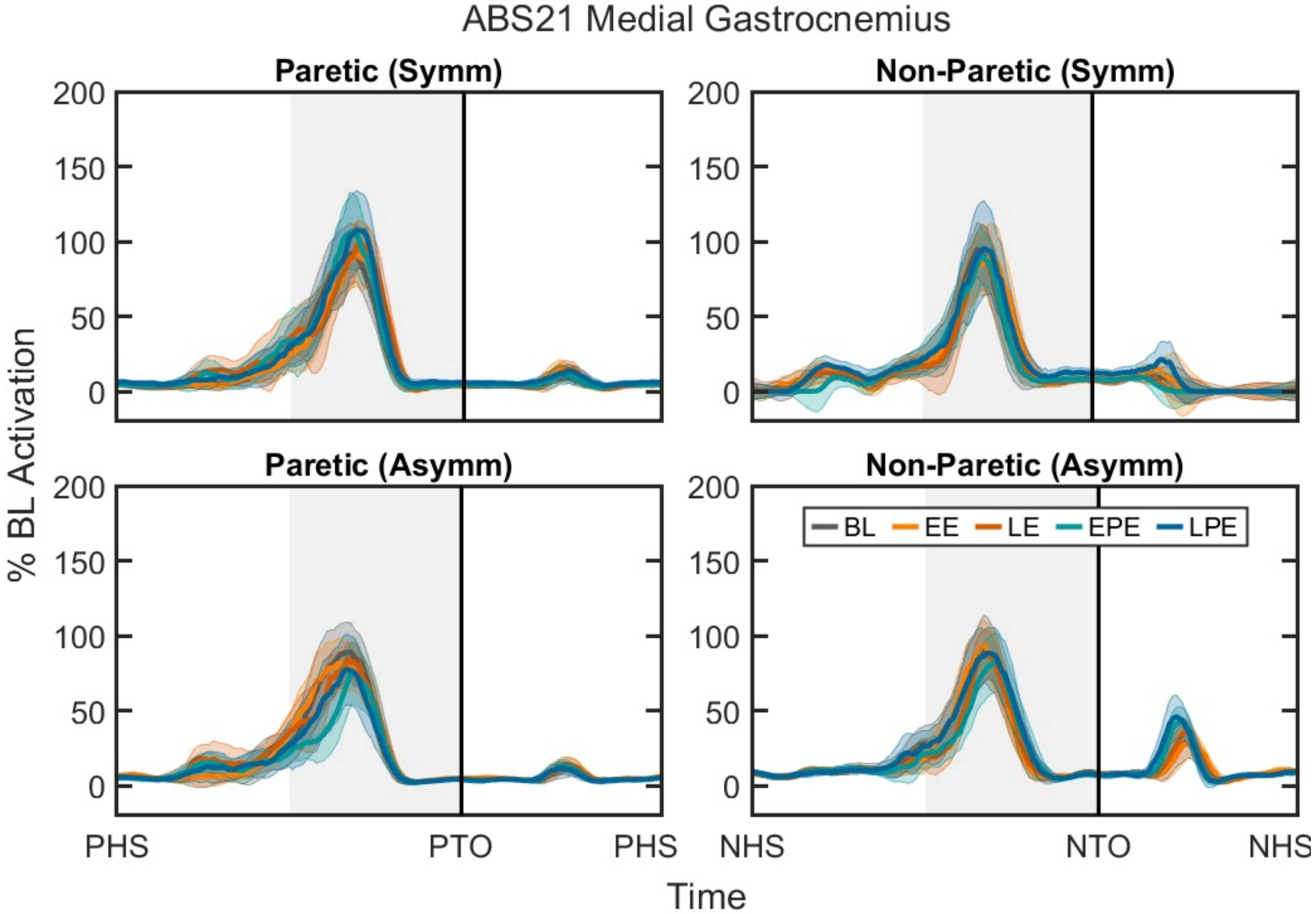
ABS21 Medial Gastrocnemius
Paretic (Symm)
Non-Paretic (Symm)
Paretic (Asymm)
Non-Paretic (Asymm)
% BL Activation
200
150
100
50
0
BL
EE
LE
EPE
LPE
PHS
PTO
PHS
NHS
NTO
NHS
Time

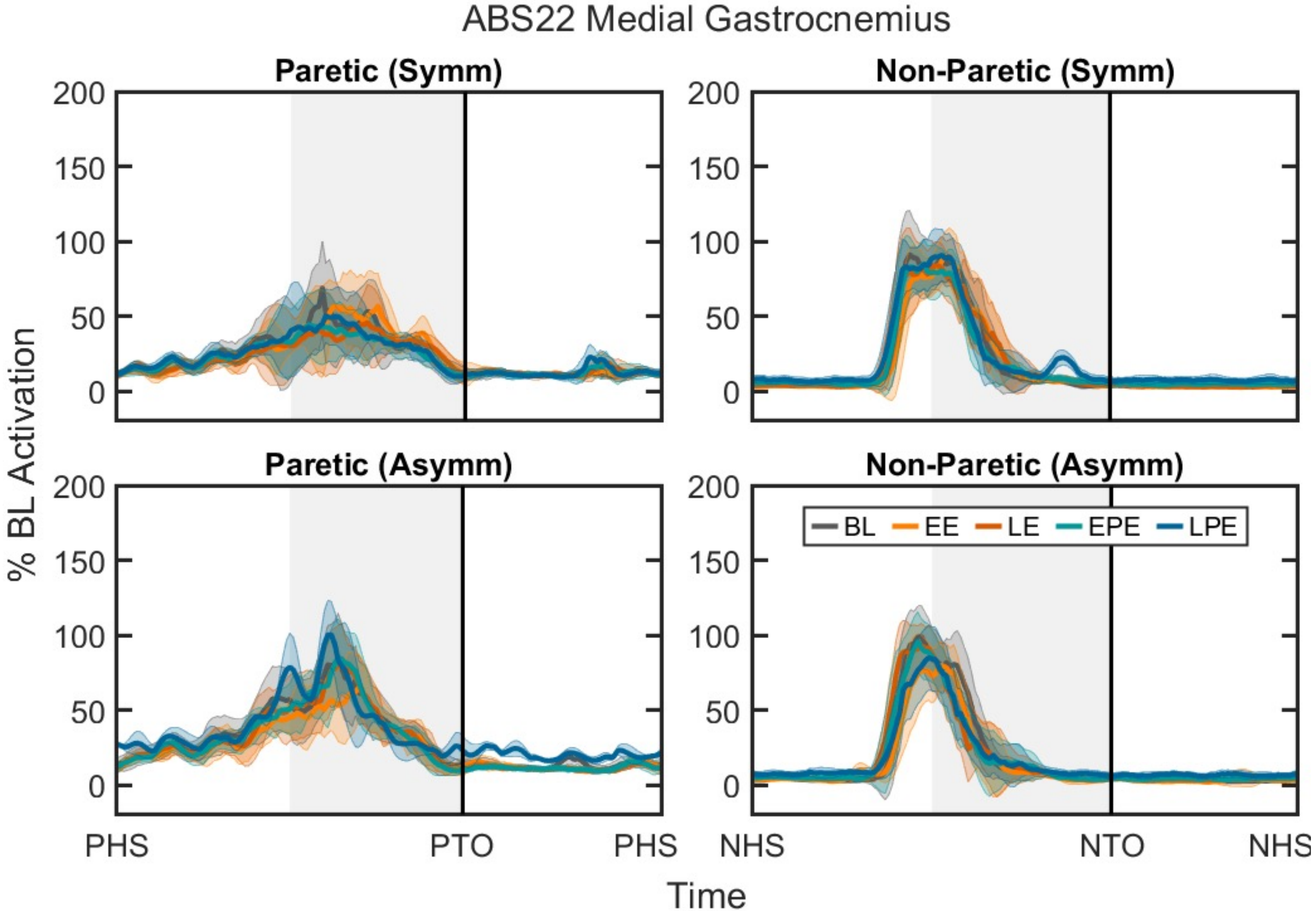
ABS22 Medial Gastrocnemius
Paretic (Symm)
Non-Paretic (Symm)
Paretic (Asymm)
Non-Paretic (Asymm)
% BL Activation
200
150
100
50
0
BL
EE
LE
EPE
LPE
PHS
PTO
PHS
NHS
NTO
NHS
Time

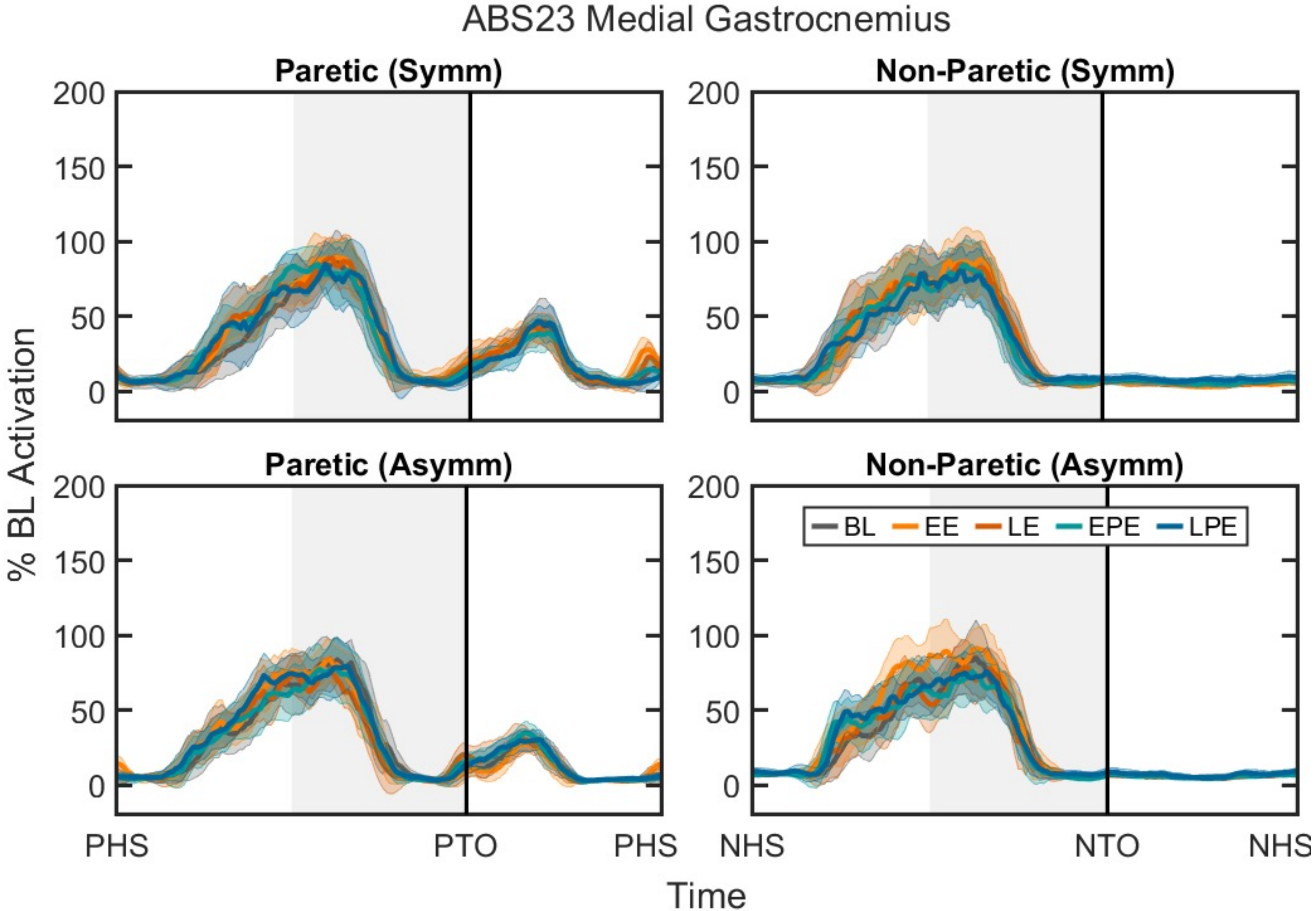

ABS23 Medial Gastrocnemius
Paretic (Symm)
Non-Paretic (Symm)
Paretic (Asymm)
Non-Paretic (Asymm)
% BL Activation
200
150
100
50
0
BL
EE
LE
EPE
LPE
PHS
PTO
PHS
NHS
NTO
NHS
Time

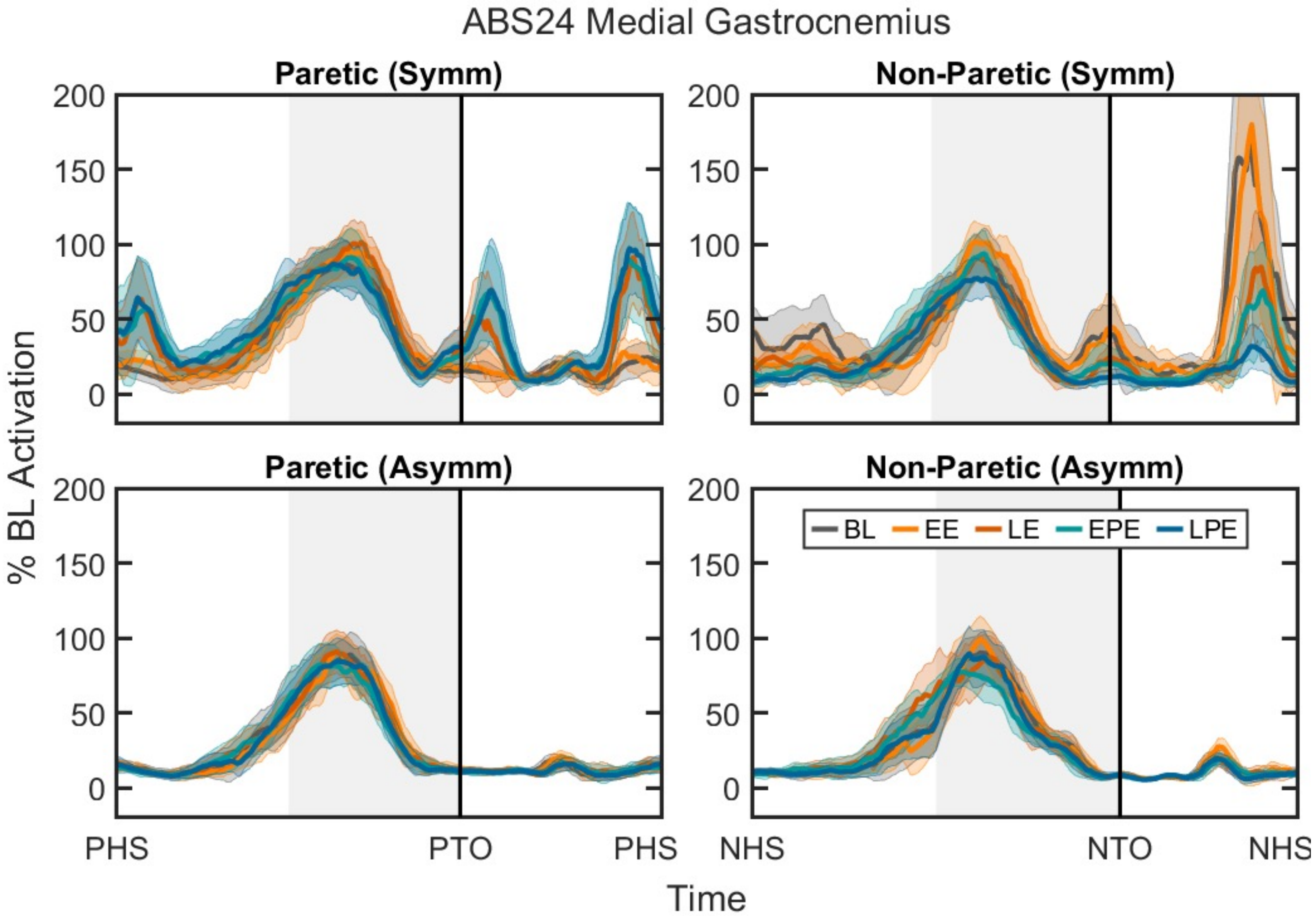
ABS24 Medial Gastrocnemius
Paretic (Symm)
Non-Paretic (Symm)
Paretic (Asymm)
Non-Paretic (Asymm)
% BL Activation
200
150
100
50
0
BL
EE
LE
EPE
LPE
PHS
PTO
PHS
NHS
NTO
NHS
Time

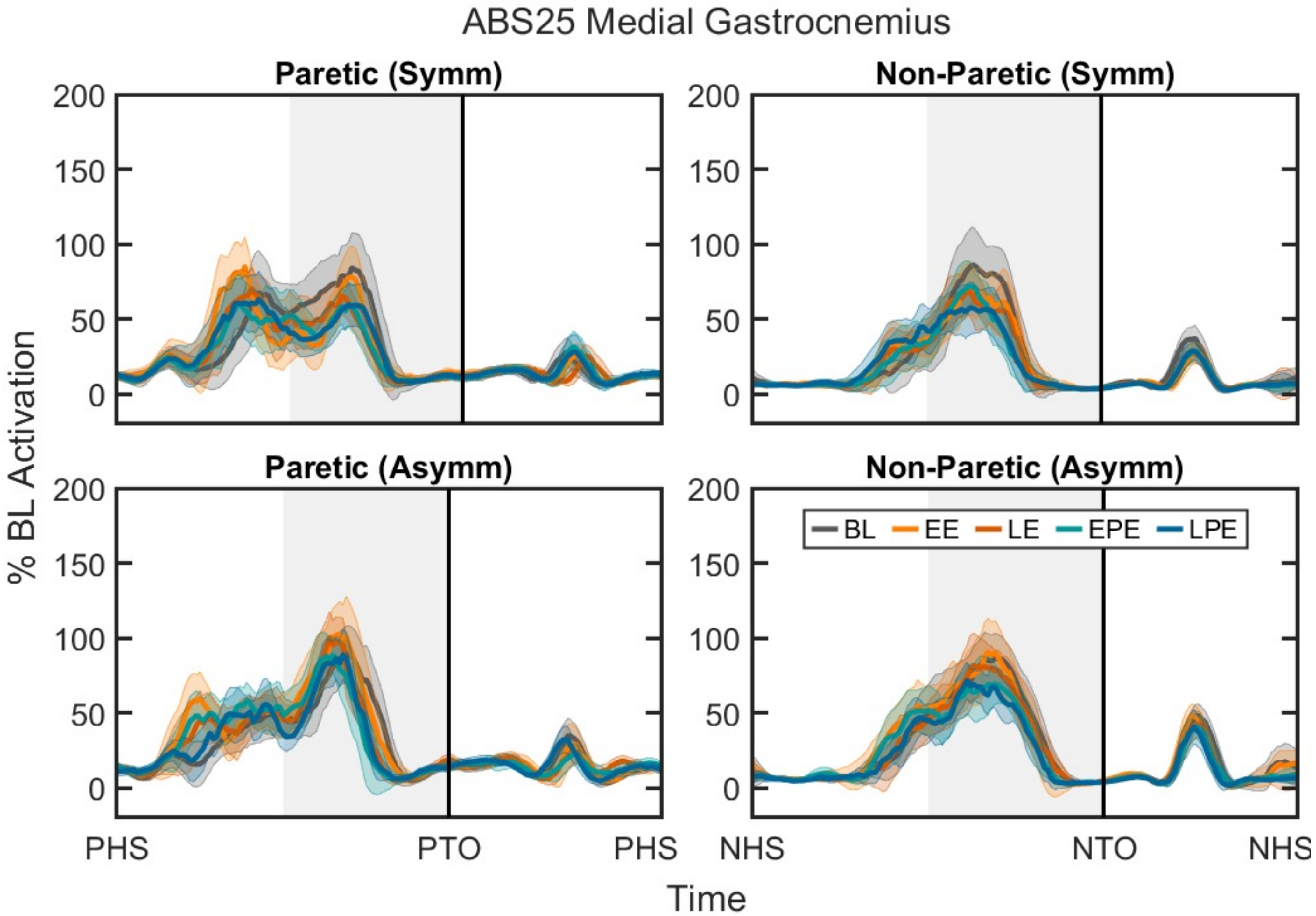
ABS25 Medial Gastrocnemius
Paretic (Symm)
Non-Paretic (Symm)
Paretic (Asymm)
Non-Paretic (Asymm)
% BL Activation
200
150
100
50
0
BL
EE
LE
EPE
LPE
PHS
PTO
PHS
NHS
NTO
NHS
Time

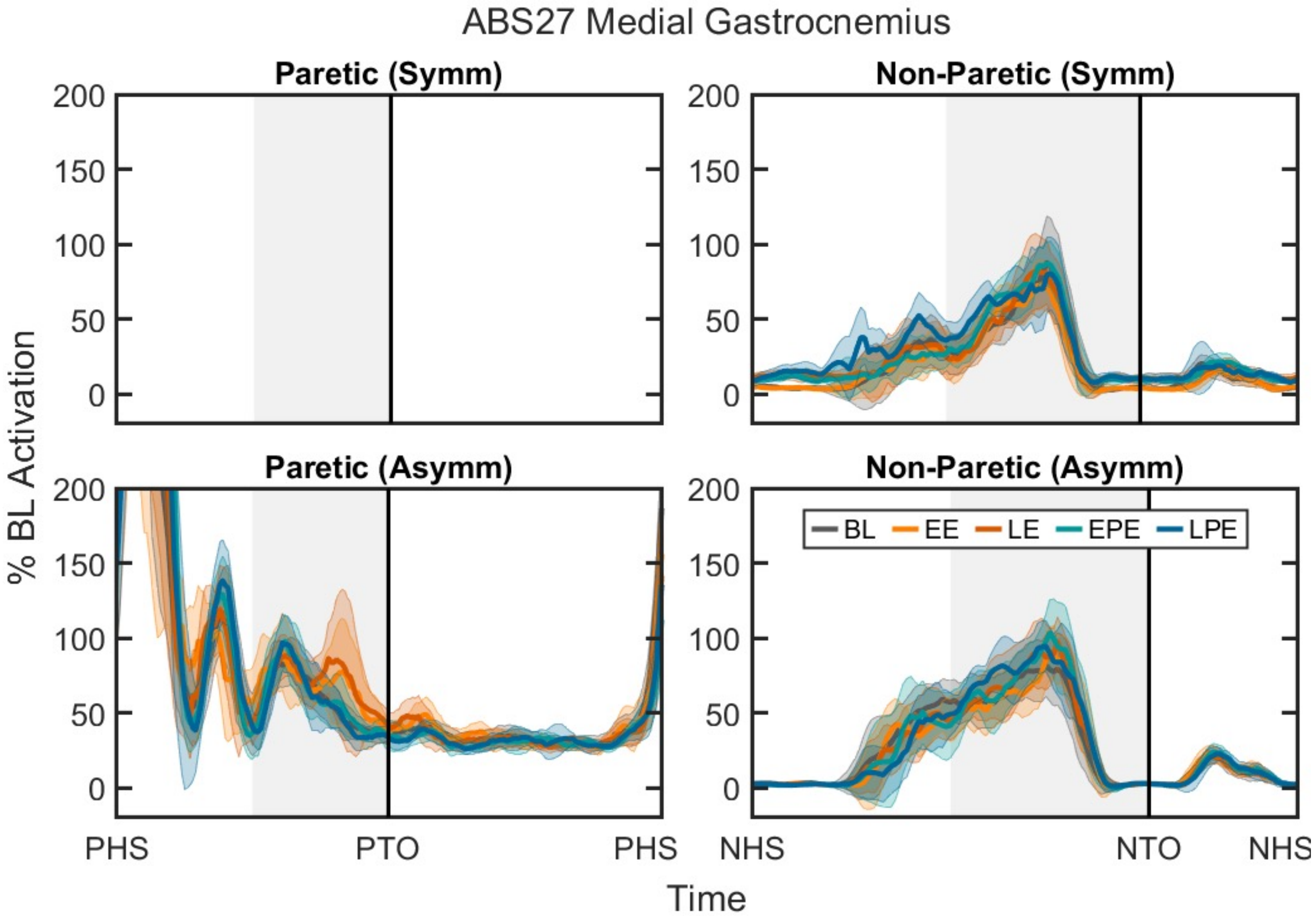
ABS27 Medial Gastrocnemius
Paretic (Symm)
Non-Paretic (Symm)
Paretic (Asymm)
Non-Paretic (Asymm)
% BL Activation
200
150
100
50
0
BL
EE
LE
EPE
LPE
PHS
PTO
PHS
NHS
NTO
NHS
Time

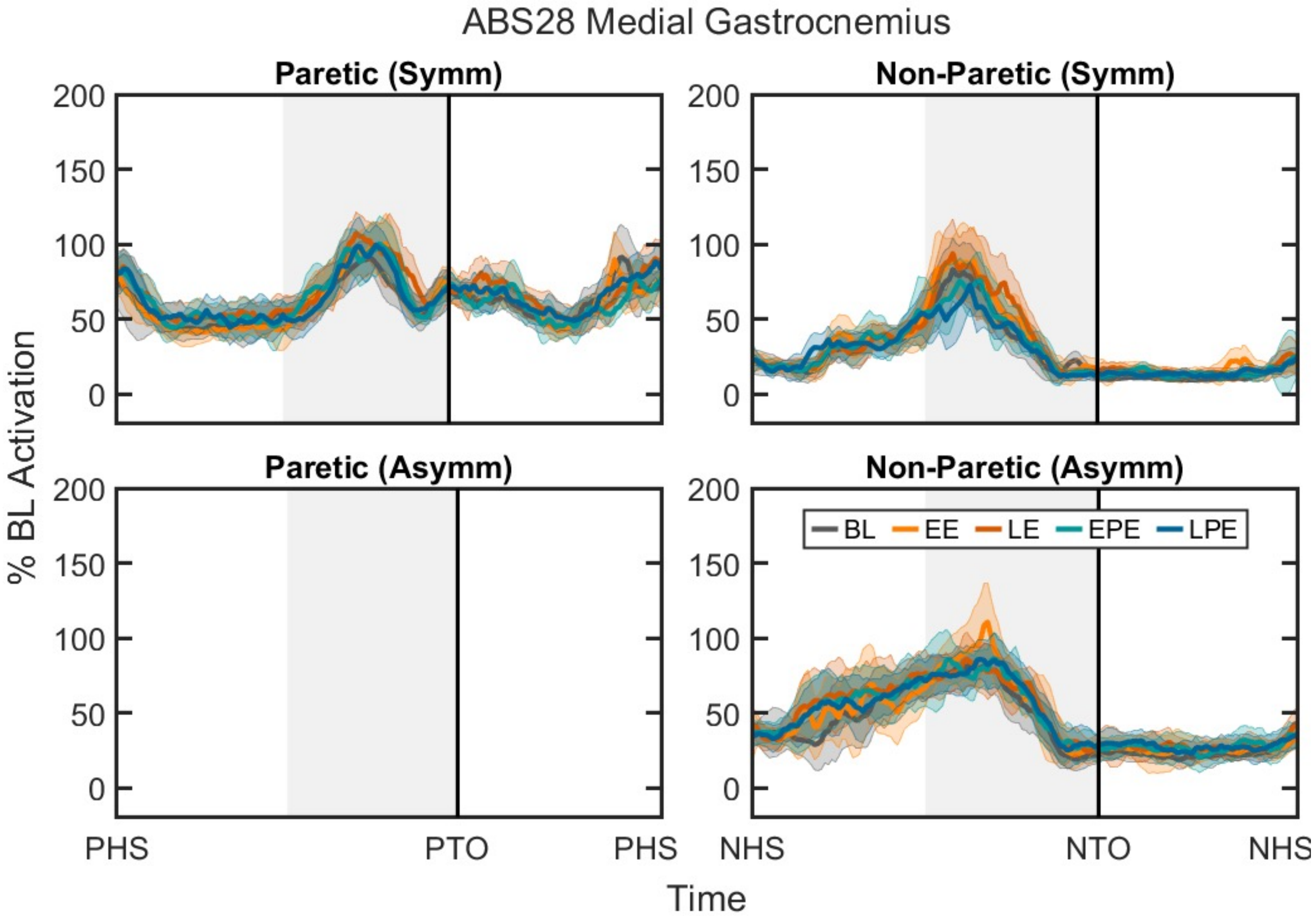

ABS28 Medial Gastrocnemius
Paretic (Symm)
Non-Paretic (Symm)
Paretic (Asymm)
Non-Paretic (Asymm)
% BL Activation
200
150
100
50
0
BL
EE
LE
EPE
LPE
PHS
PTO
PHS
NHS
NTO
NHS
Time

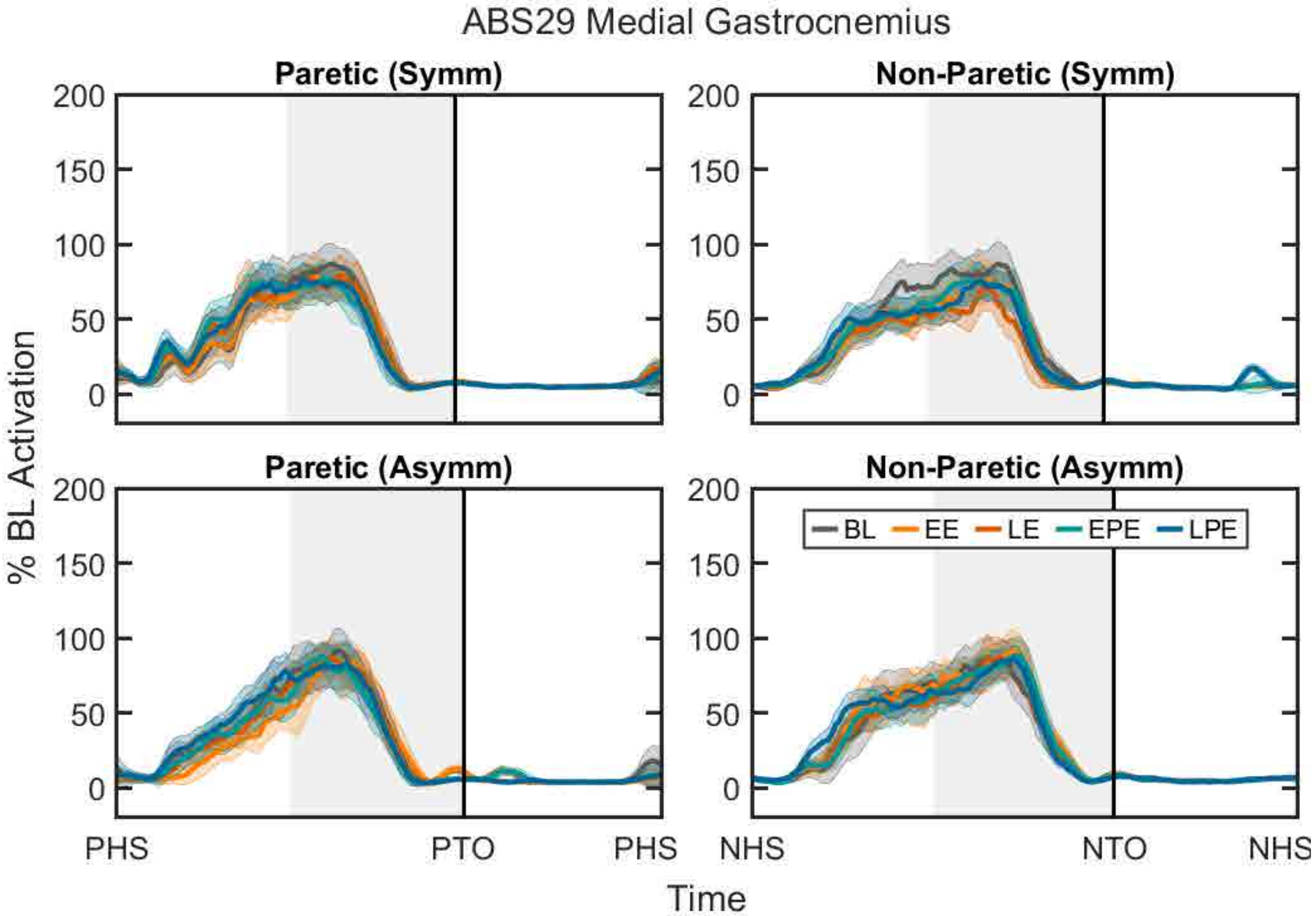
ABS29 Medial Gastrocnemius
Paretic (Symm)
Non-Paretic (Symm)
Paretic (Asymm)
Non-Paretic (Asymm)
% BL Activation
200
150
100
50
0
BL
EE
LE
EPE
LPE
PHS
PTO
PHS
NHS
NTO
NHS
Time

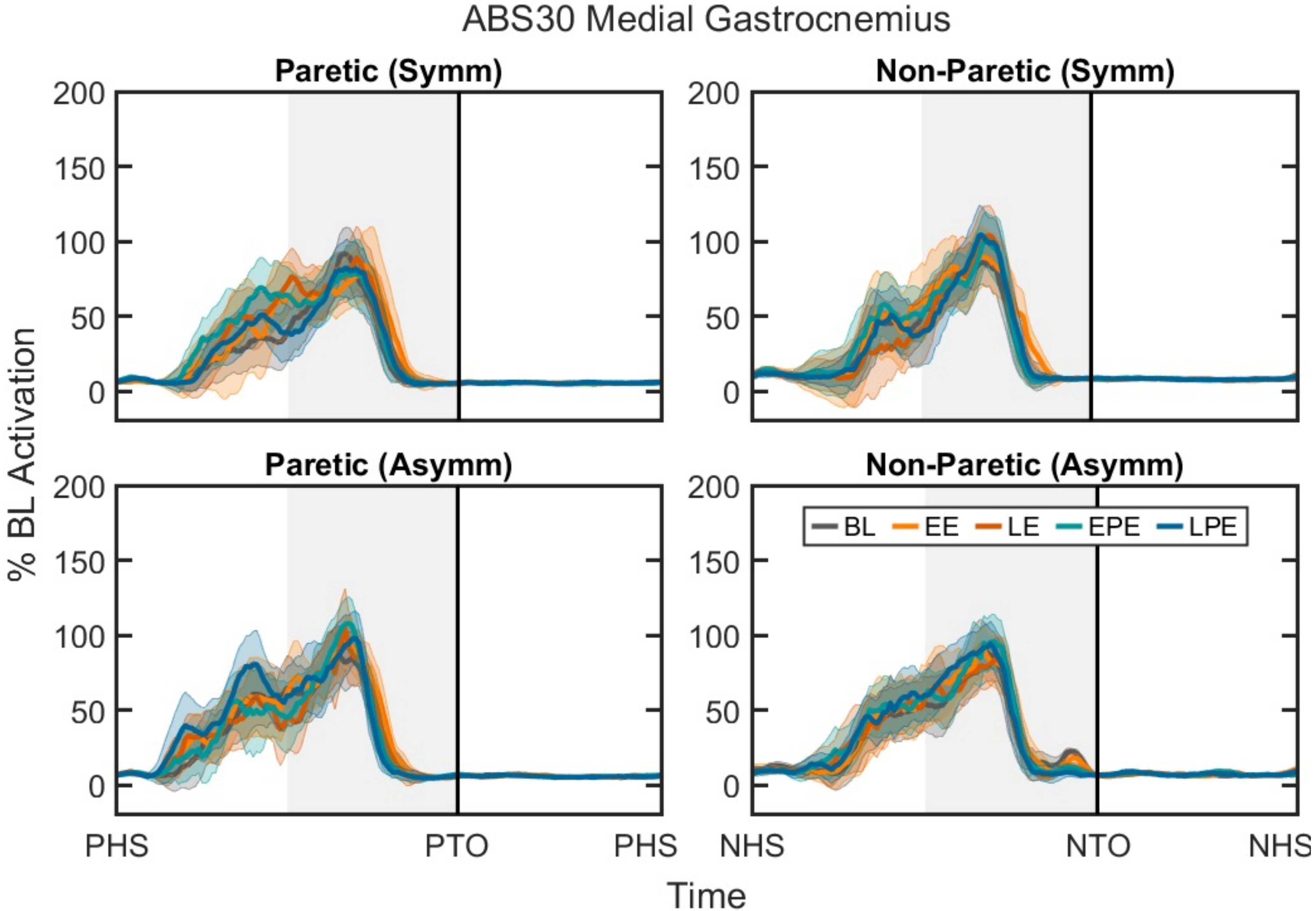
ABS30 Medial Gastrocnemius
Paretic (Symm)
Non-Paretic (Symm)
Paretic (Asymm)
Non-Paretic (Asymm)
% BL Activation
200
150
100
50
0
BL
EE
LE
EPE
LPE
PHS
PTO
PHS
NHS
NTO
NHS
Time

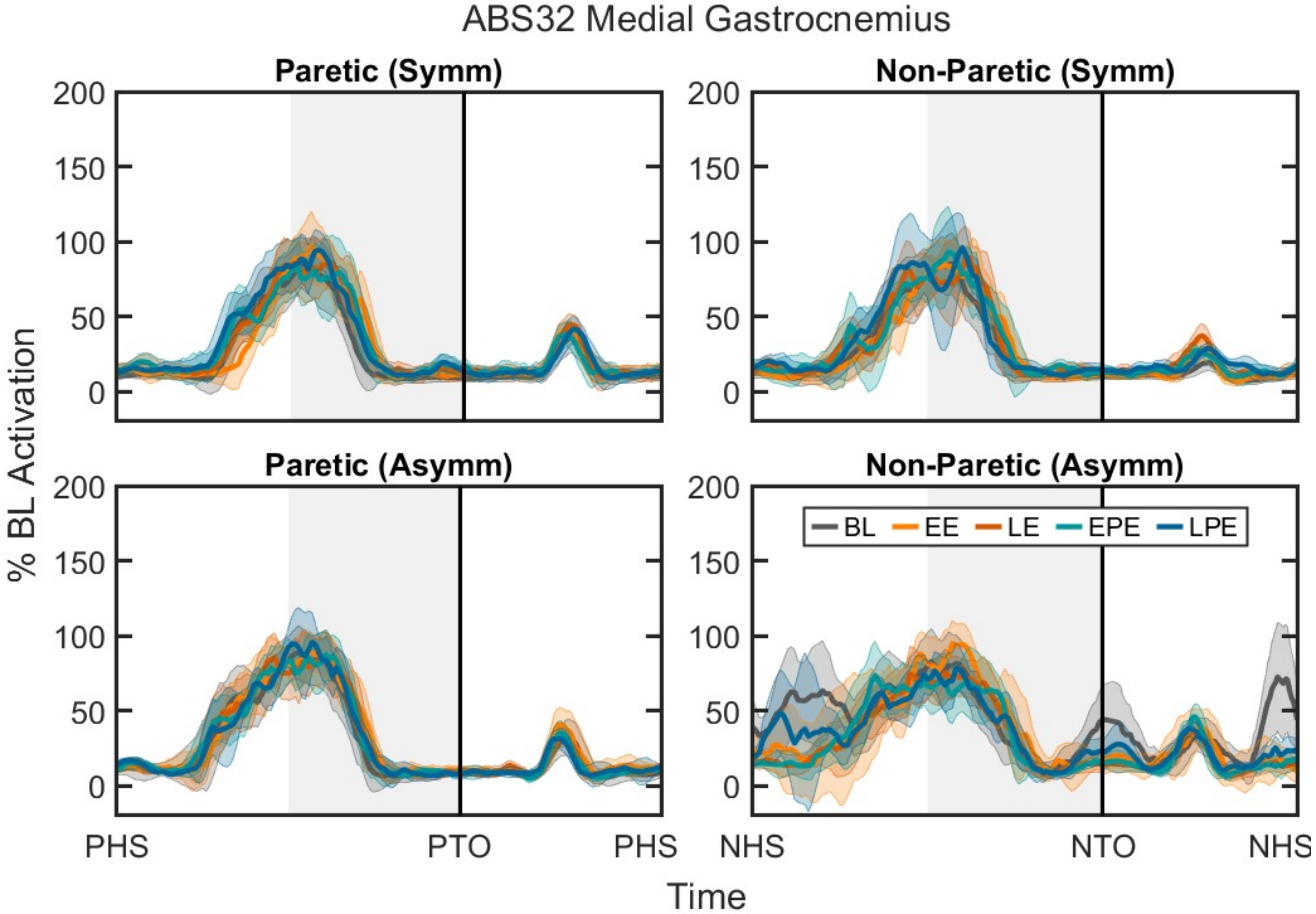
ABS32 Medial Gastrocnemius
Paretic (Symm)
Non-Paretic (Symm)
Paretic (Asymm)
Non-Paretic (Asymm)
% BL Activation
200
150
100
50
0
BL
EE
LE
EPE
LPE
PHS
PTO
PHS
NHS
NTO
NHS
Time

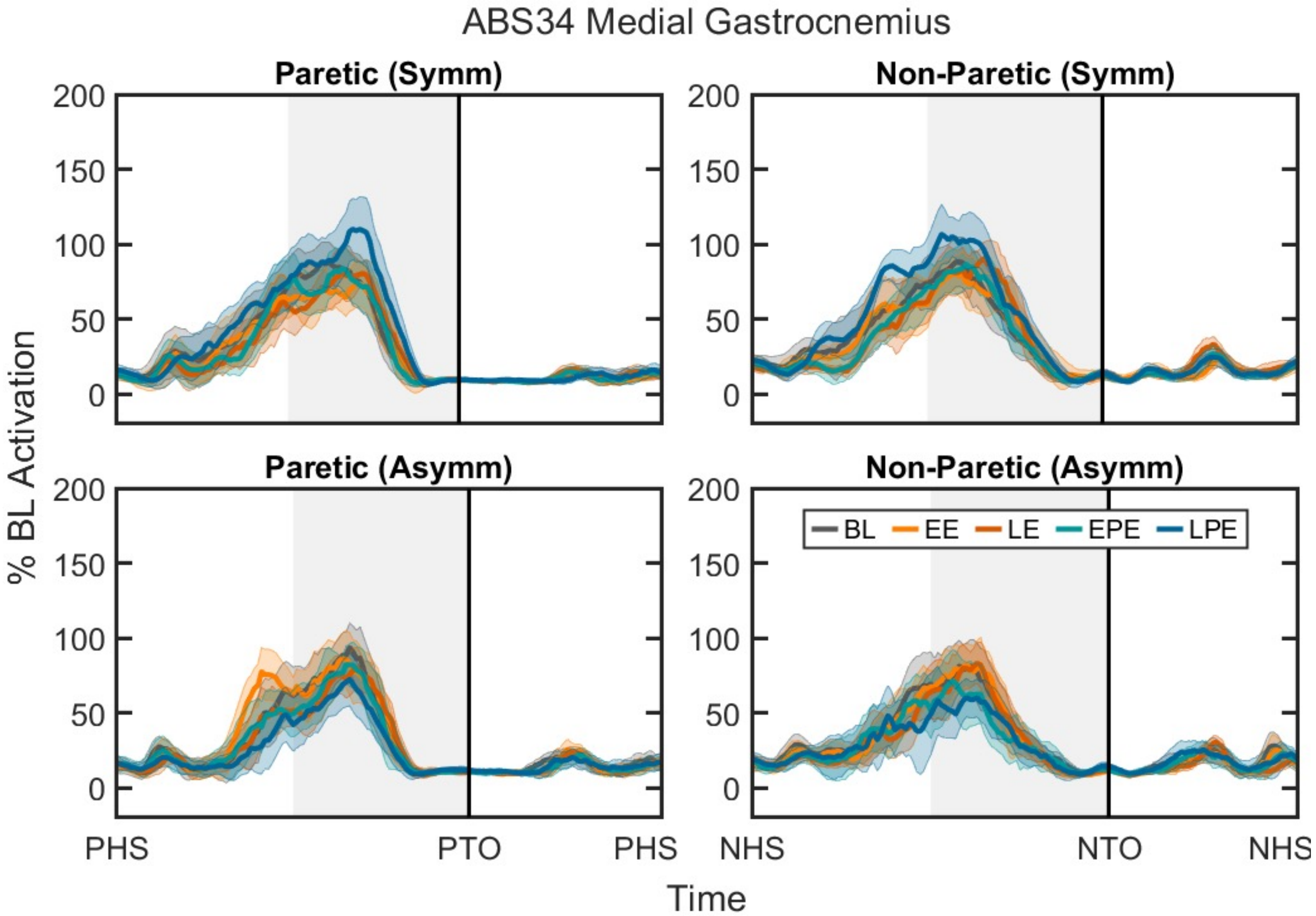
ABS34 Medial Gastrocnemius
Paretic (Symm)
Non-Paretic (Symm)
Paretic (Asymm)
Non-Paretic (Asymm)
% BL Activation
200
150
100
50
0
BL
EE
LE
EPE
LPE
PHS
PTO
PHS
NHS
NTO
NHS
Time

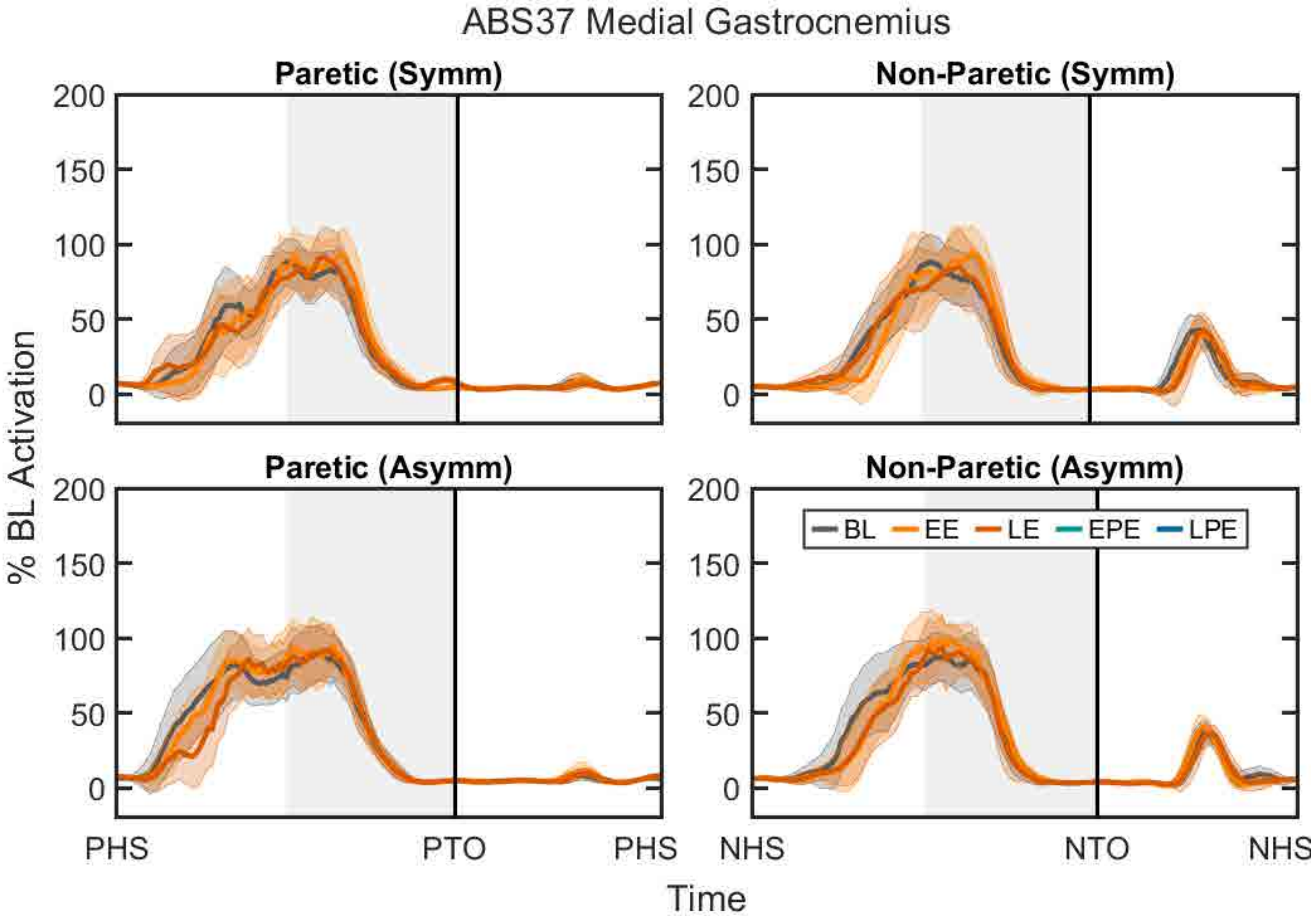

ABS37 Medial Gastrocnemius
Paretic (Symm)
Non-Paretic (Symm)
Paretic (Asymm)
Non-Paretic (Asymm)
% BL Activation
200
150
100
50
0
BL
EE
LE
EPE
LPE
PHS
PTO
PHS
NHS
NTO
NHS
Time

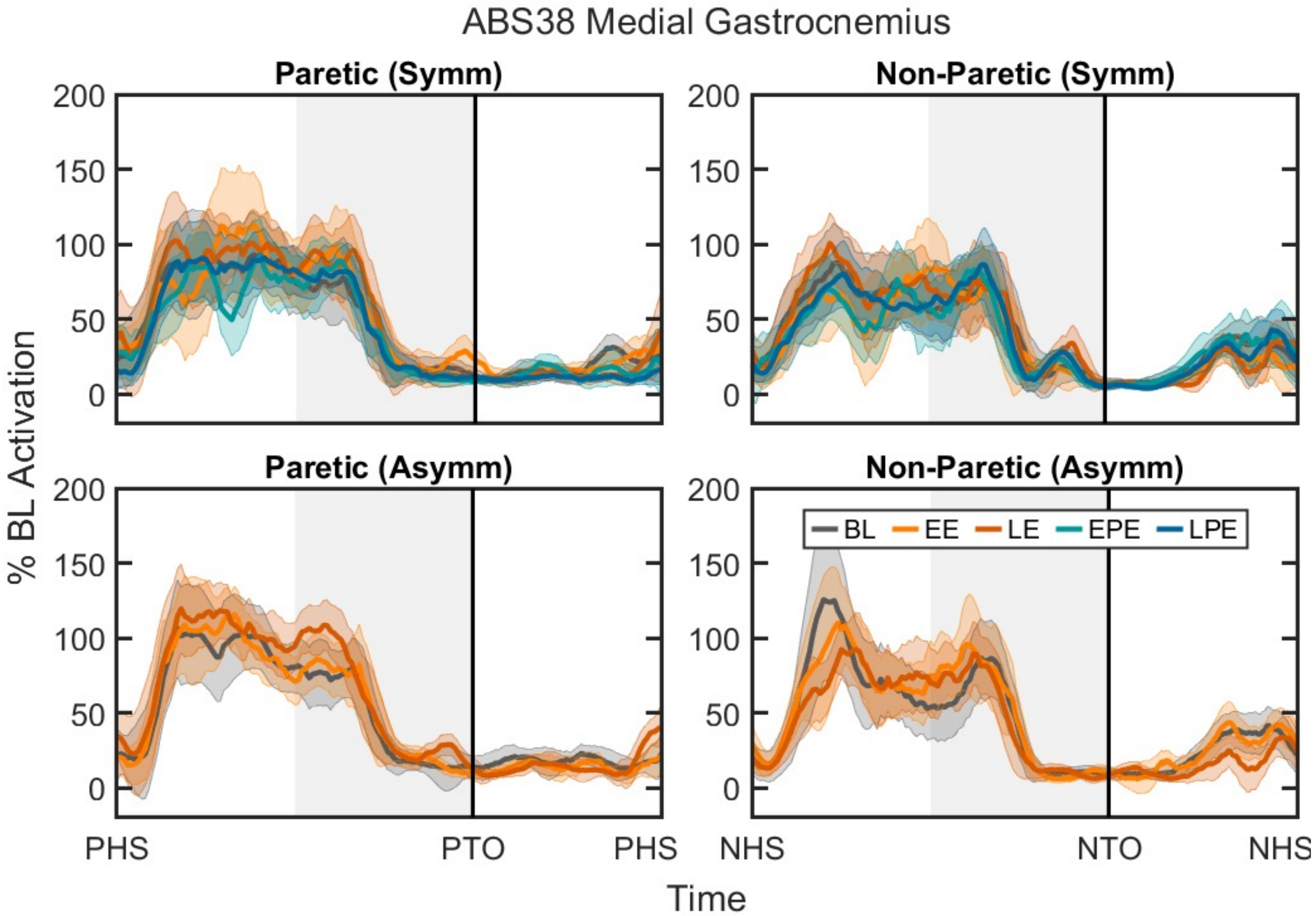

ABS38 Medial Gastrocnemius
Paretic (Symm)
Non-Paretic (Symm)
Paretic (Asymm)
Non-Paretic (Asymm)
% BL Activation
200
150
100
50
0
BL
EE
LE
EPE
LPE
PHS
PTO
PHS
NHS
NTO
NHS
Time

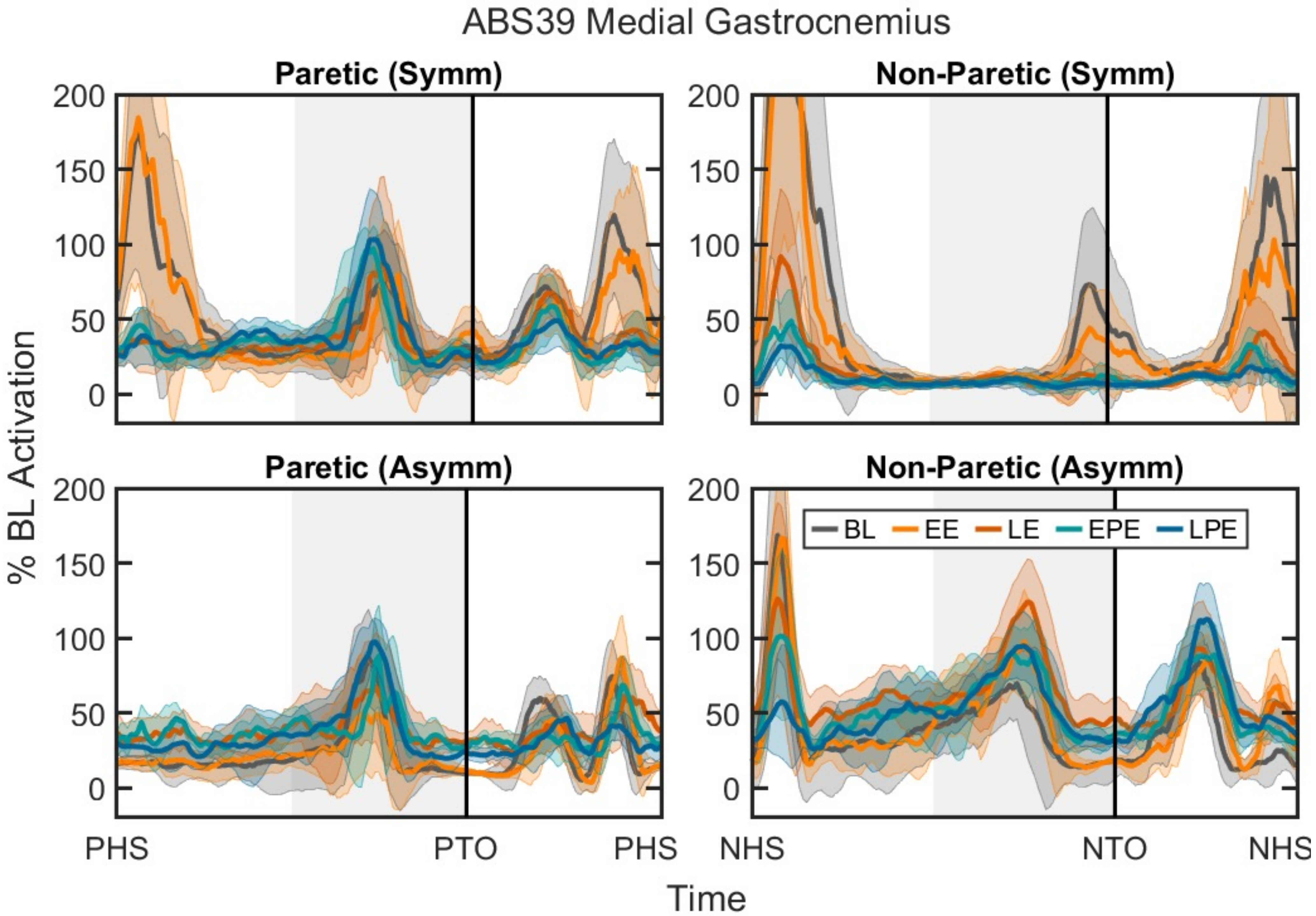

ABS39 Medial Gastrocnemius
Paretic (Symm)
Non-Paretic (Symm)
Paretic (Asymm)
Non-Paretic (Asymm)
% BL Activation
200
150
100
50
0
BL
EE
LE
EPE
LPE
PHS
PTO
PHS
NHS
NTO
NHS
Time

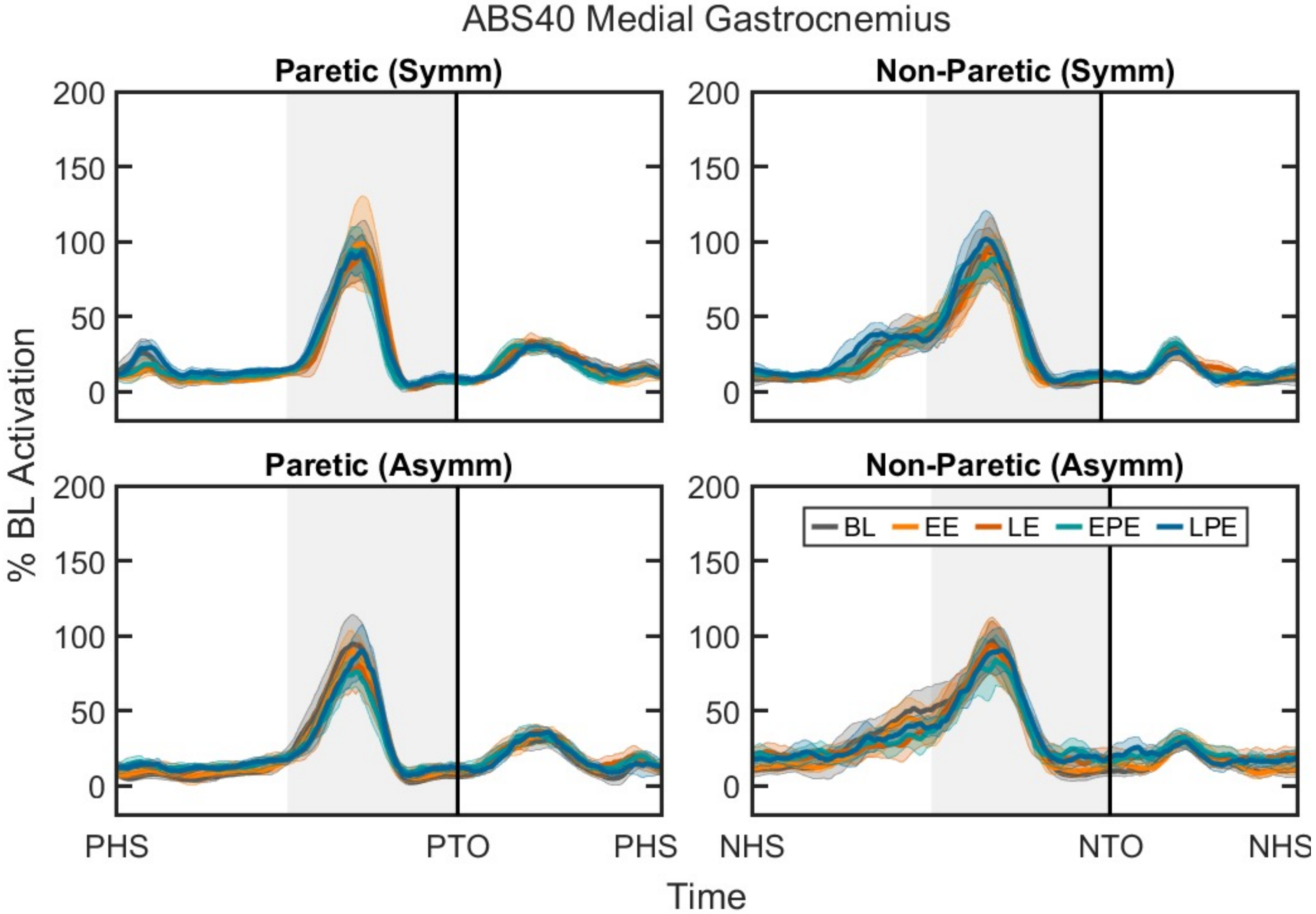
ABS40 Medial Gastrocnemius
Paretic (Symm)
Non-Paretic (Symm)
Paretic (Asymm)
Non-Paretic (Asymm)
% BL Activation
200
150
100
50
0
BL
EE
LE
EPE
LPE
PHS
PTO
PHS
NHS
NTO
NHS
Time

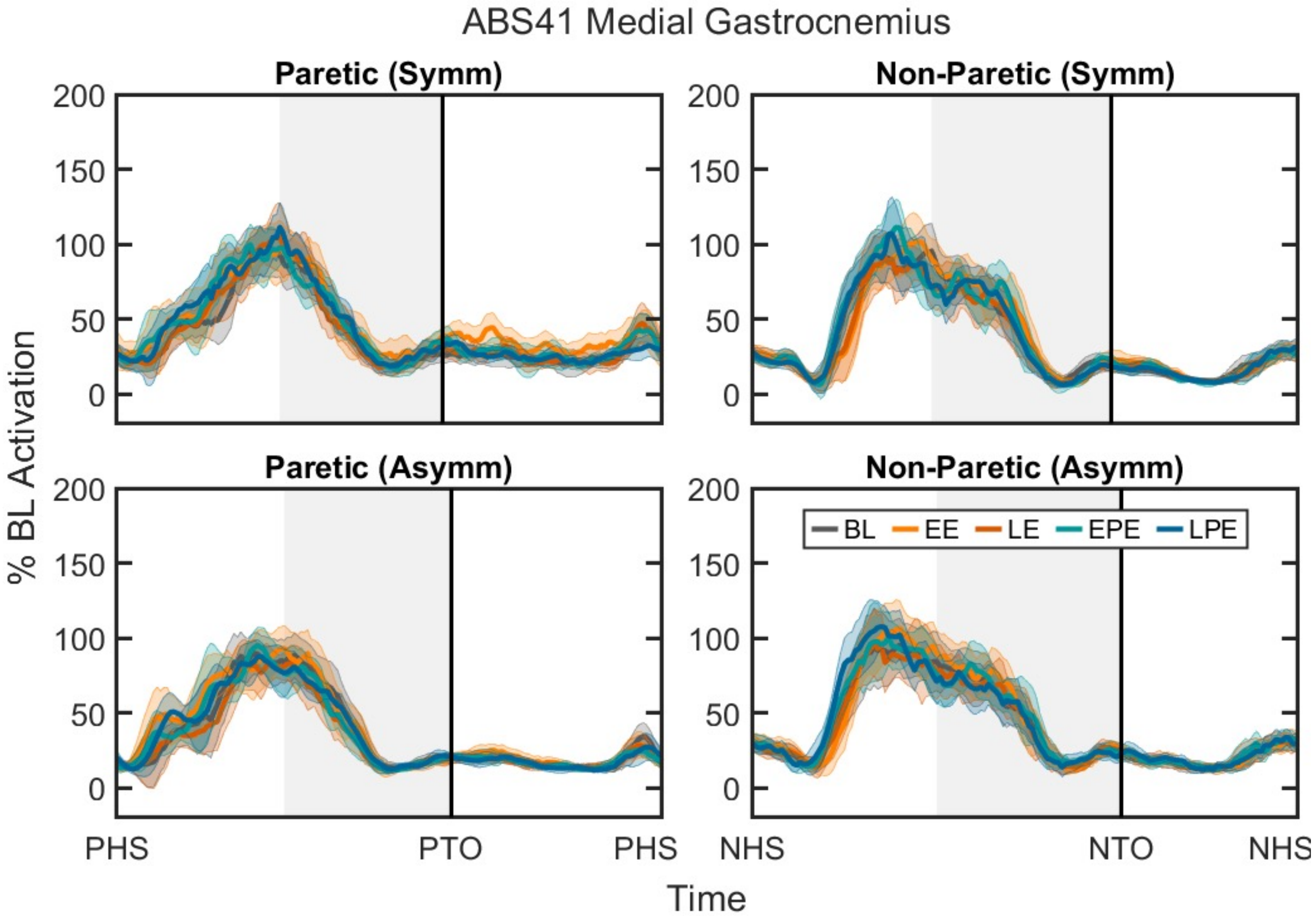
ABS41 Medial Gastrocnemius
Paretic (Symm)
Non-Paretic (Symm)
Paretic (Asymm)
Non-Paretic (Asymm)
% BL Activation
200
150
100
50
0
BL
EE
LE
EPE
LPE
PHS
PTO
PHS
NHS
NTO
NHS
Time

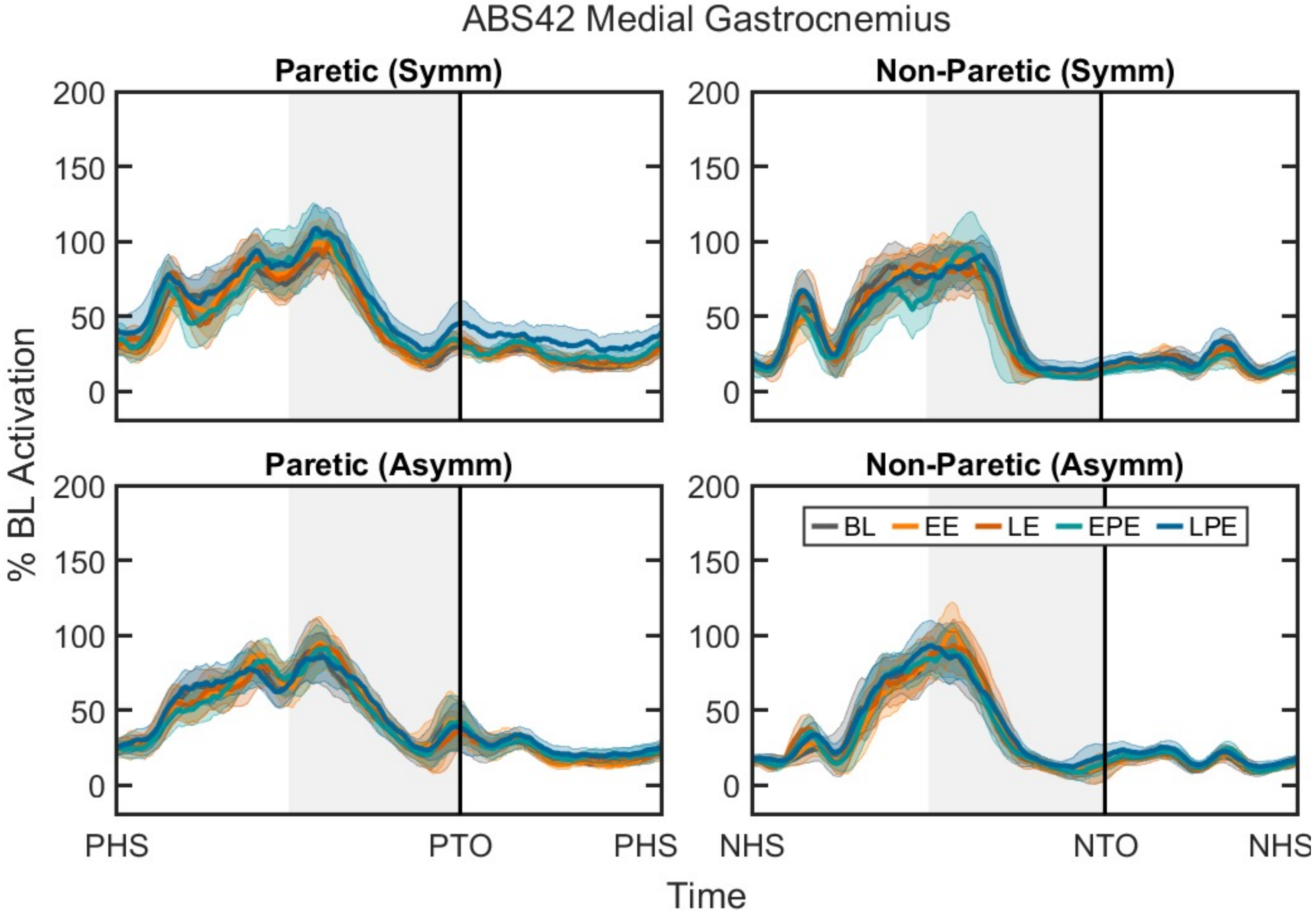
ABS42 Medial Gastrocnemius
Paretic (Symm)
Non-Paretic (Symm)
Paretic (Asymm)
Non-Paretic (Asymm)
% BL Activation
200
150
100
50
0
BL
EE
LE
EPE
LPE
PHS
PTO
PHS
NHS
NTO
NHS
Time

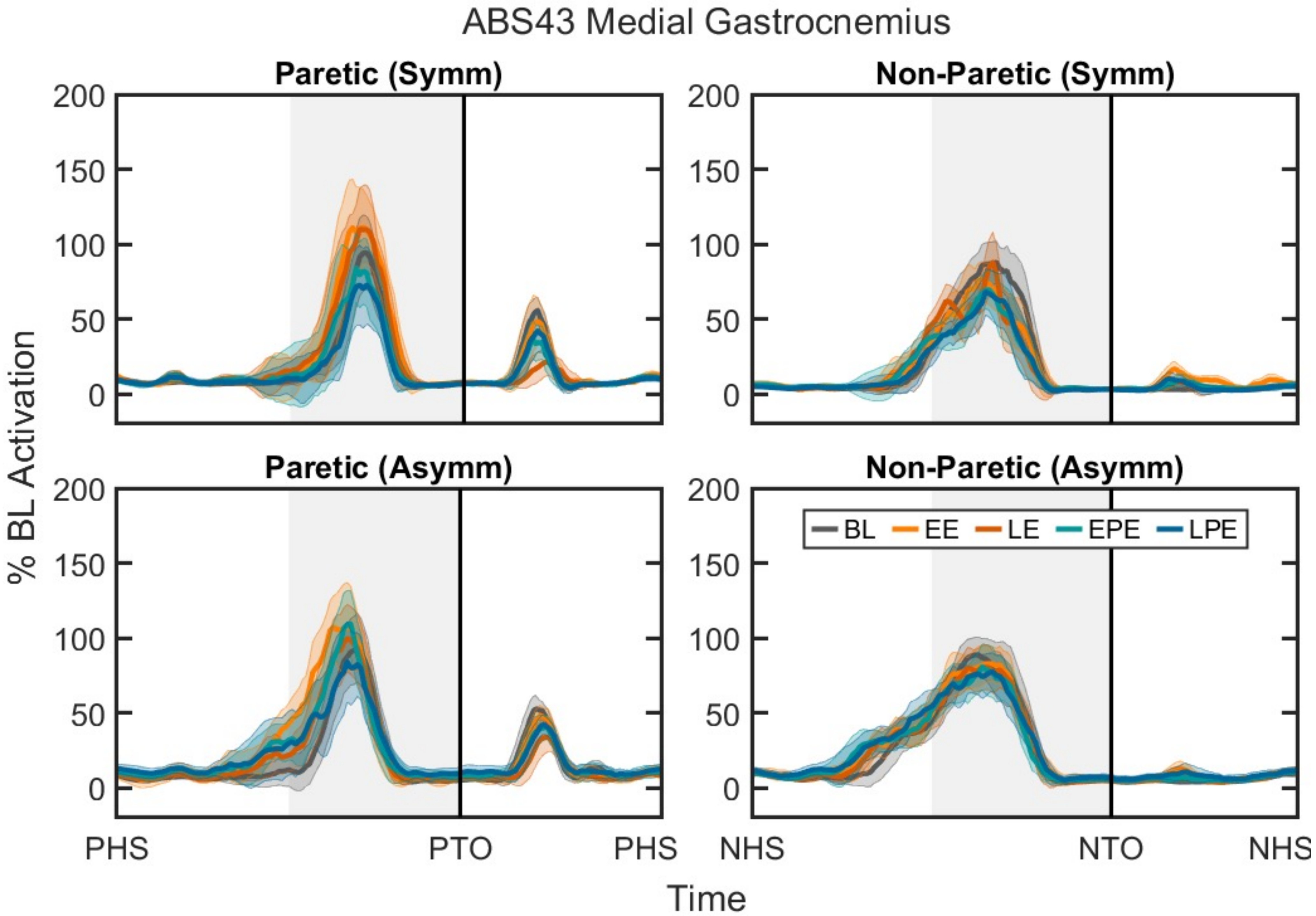
ABS43 Medial Gastrocnemius
Paretic (Symm)
Non-Paretic (Symm)
Paretic (Asymm)
Non-Paretic (Asymm)
% BL Activation
200
150
100
50
0
BL
EE
LE
EPE
LPE
PHS
PTO
PHS
NHS
NTO
NHS
Time

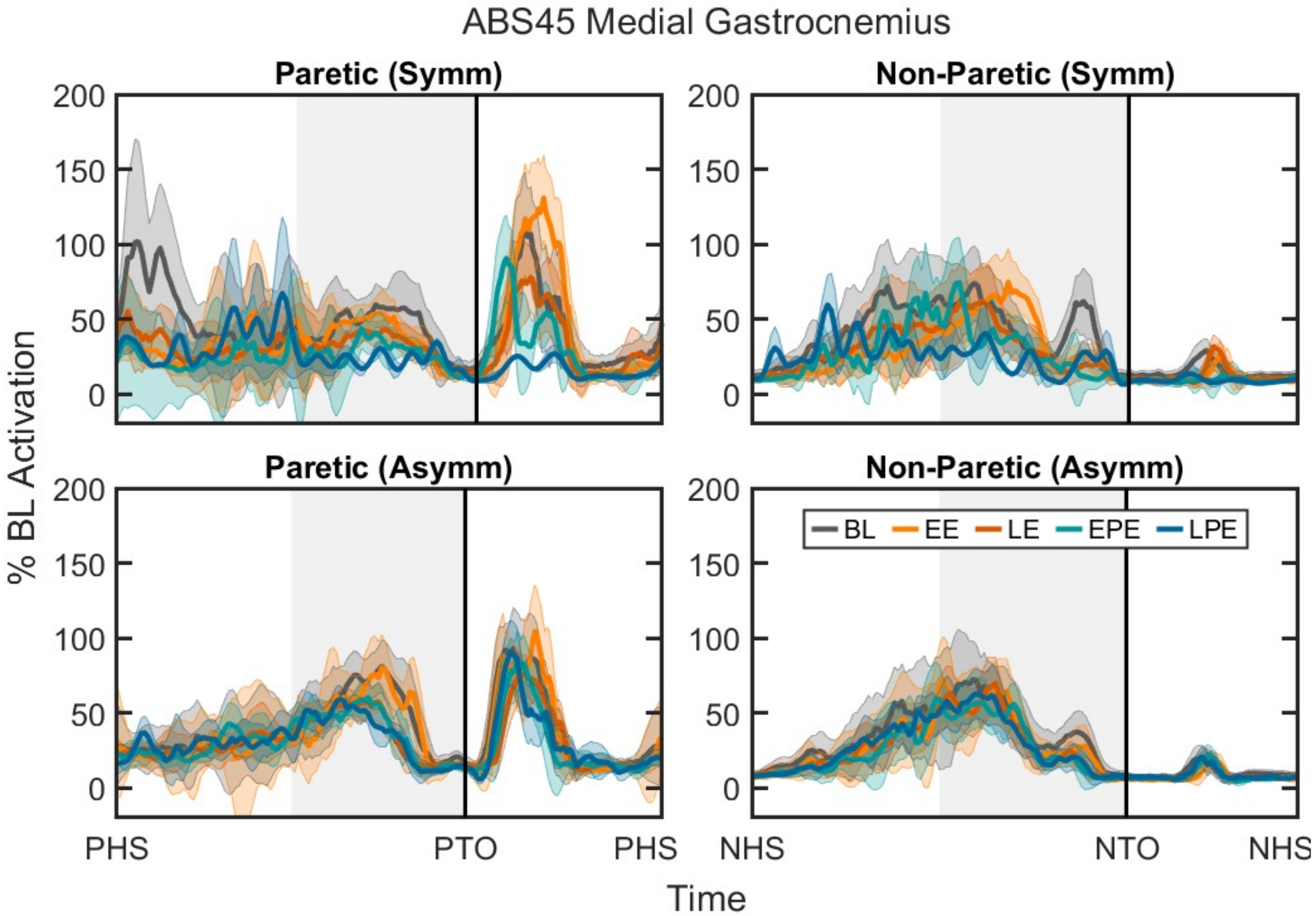

ABS45 Medial Gastrocnemius
Paretic (Symm)
Non-Paretic (Symm)
Paretic (Asymm)
Non-Paretic (Asymm)
% BL Activation
200
150
100
50
0
BL
EE
LE
EPE
LPE
PHS
PTO
PHS
NHS
NTO
NHS
Time

Tibialis Anterior

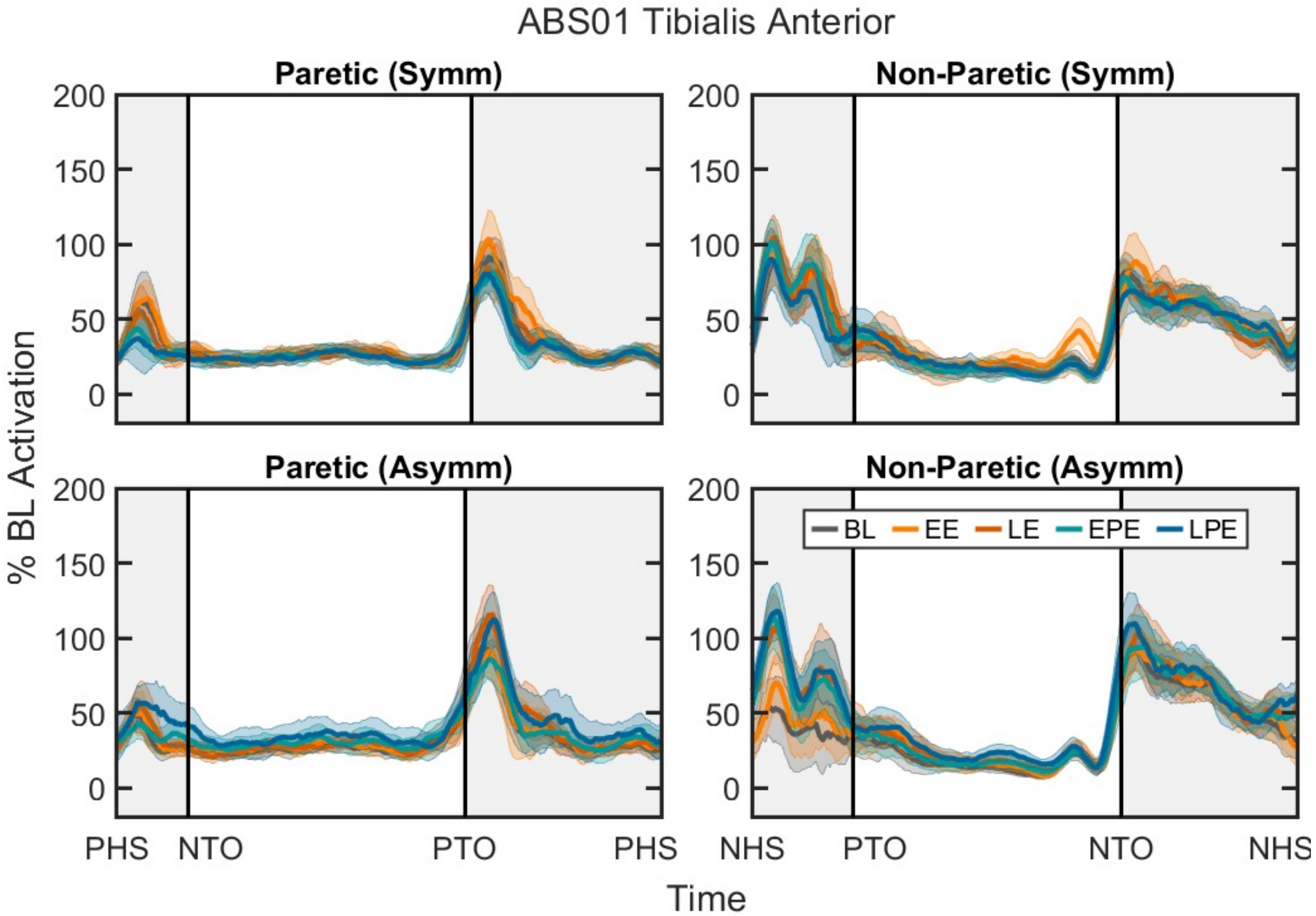
ABS01 Tibialis Anterior
Paretic (Symm)
Non-Paretic (Symm)
Paretic (Asymm)
Non-Paretic (Asymm)
% BL Activation
200
150
100
50
0
BL
EE
LE
EPE
LPE
PHS
NTO
PTO
PHS
NHS
PTO
NTO
NHS
Time

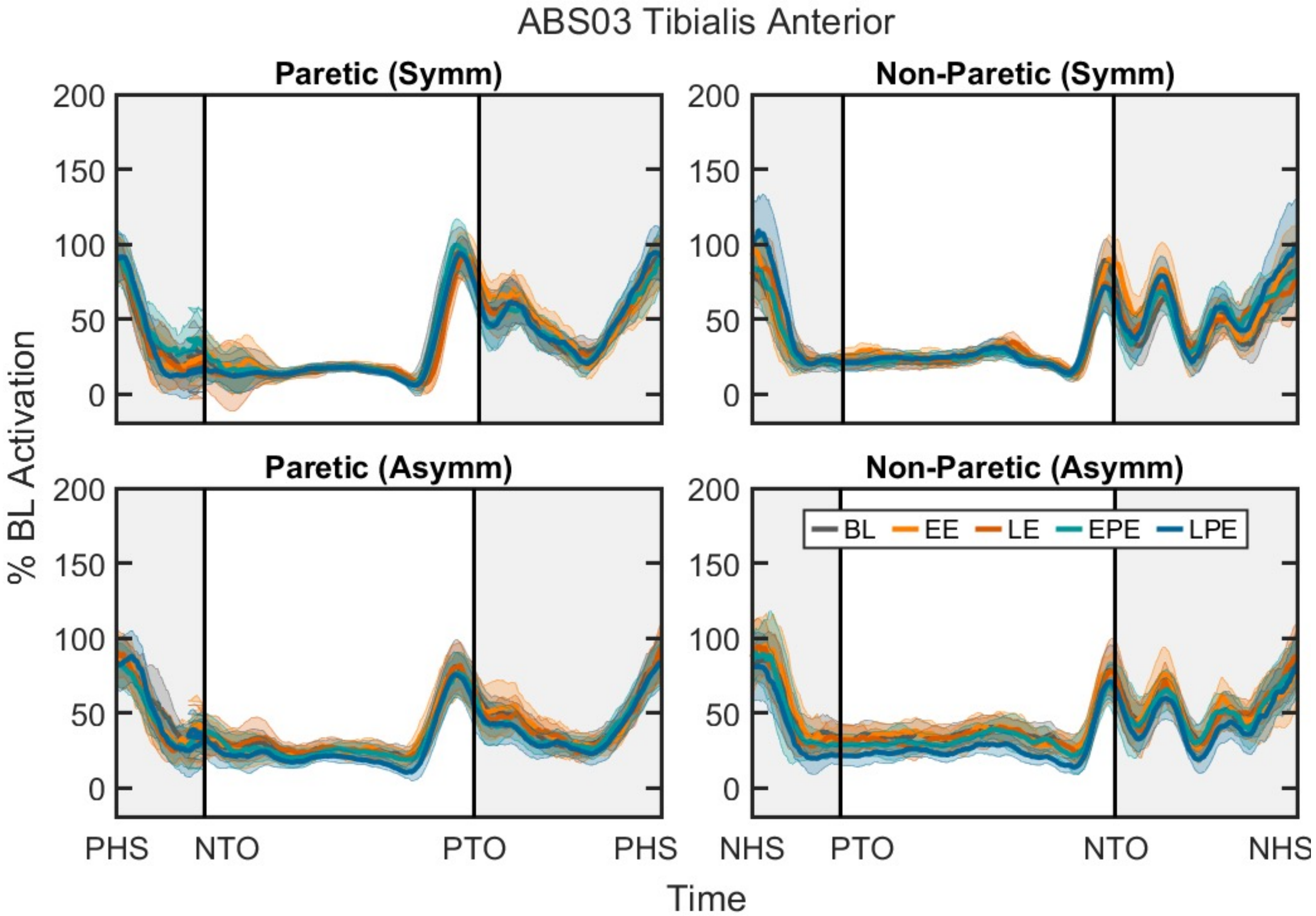

ABS03 Tibialis Anterior
Paretic (Symm)
Non-Paretic (Symm)
Paretic (Asymm)
Non-Paretic (Asymm)
% BL Activation
200
150
100
50
0
BL
EE
LE
EPE
LPE
PHS
NTO
PTO
PHS
NHS
PTO
NTO
NHS
Time

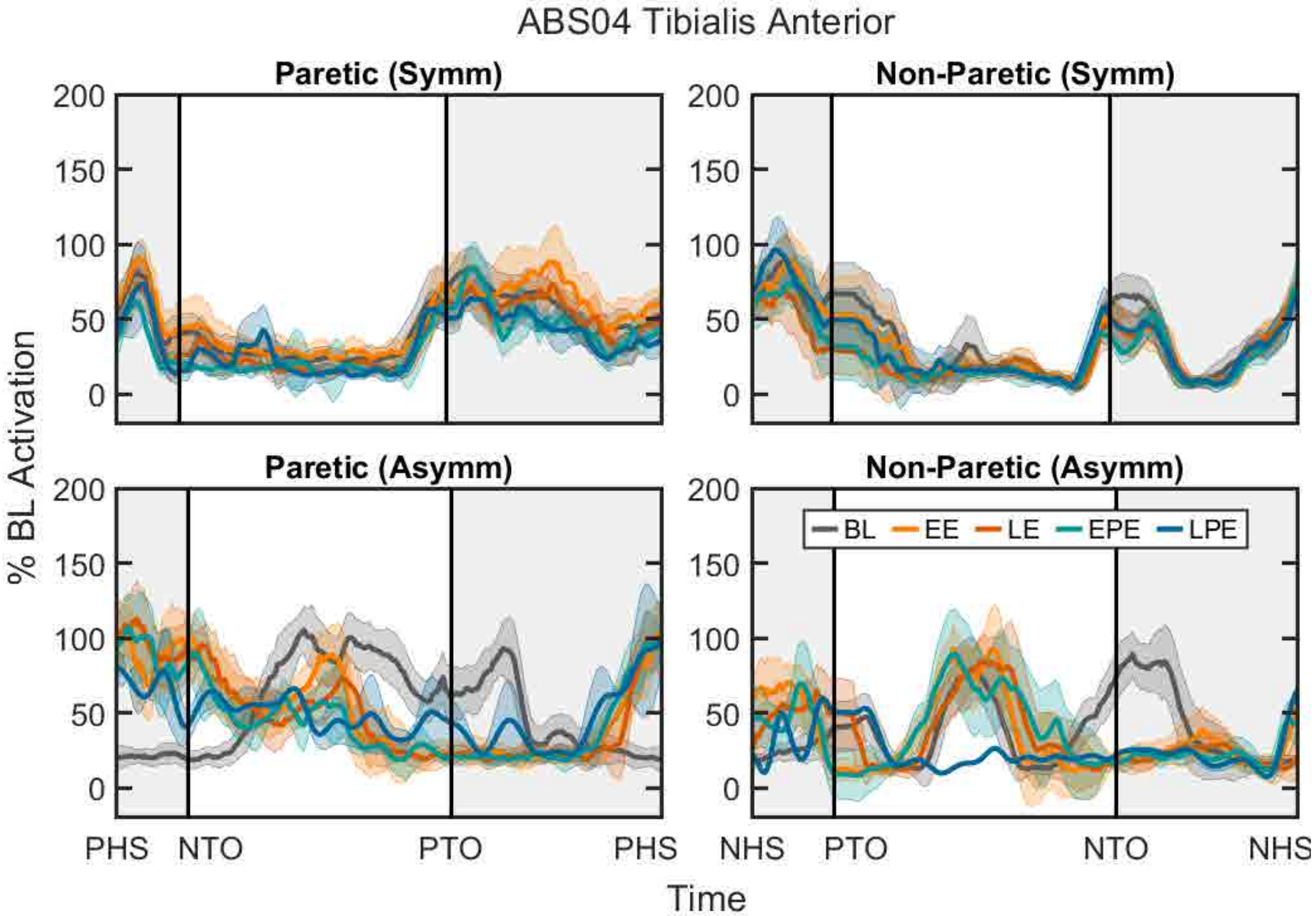
ABS04 Tibialis Anterior
Paretic (Symm)
Non-Paretic (Symm)
Paretic (Asymm)
Non-Paretic (Asymm)
% BL Activation
200
150
100
50
0
BL
EE
LE
EPE
LPE
PHS
NTO
PTO
PHS
NHS
PTO
NTO
NHS
Time

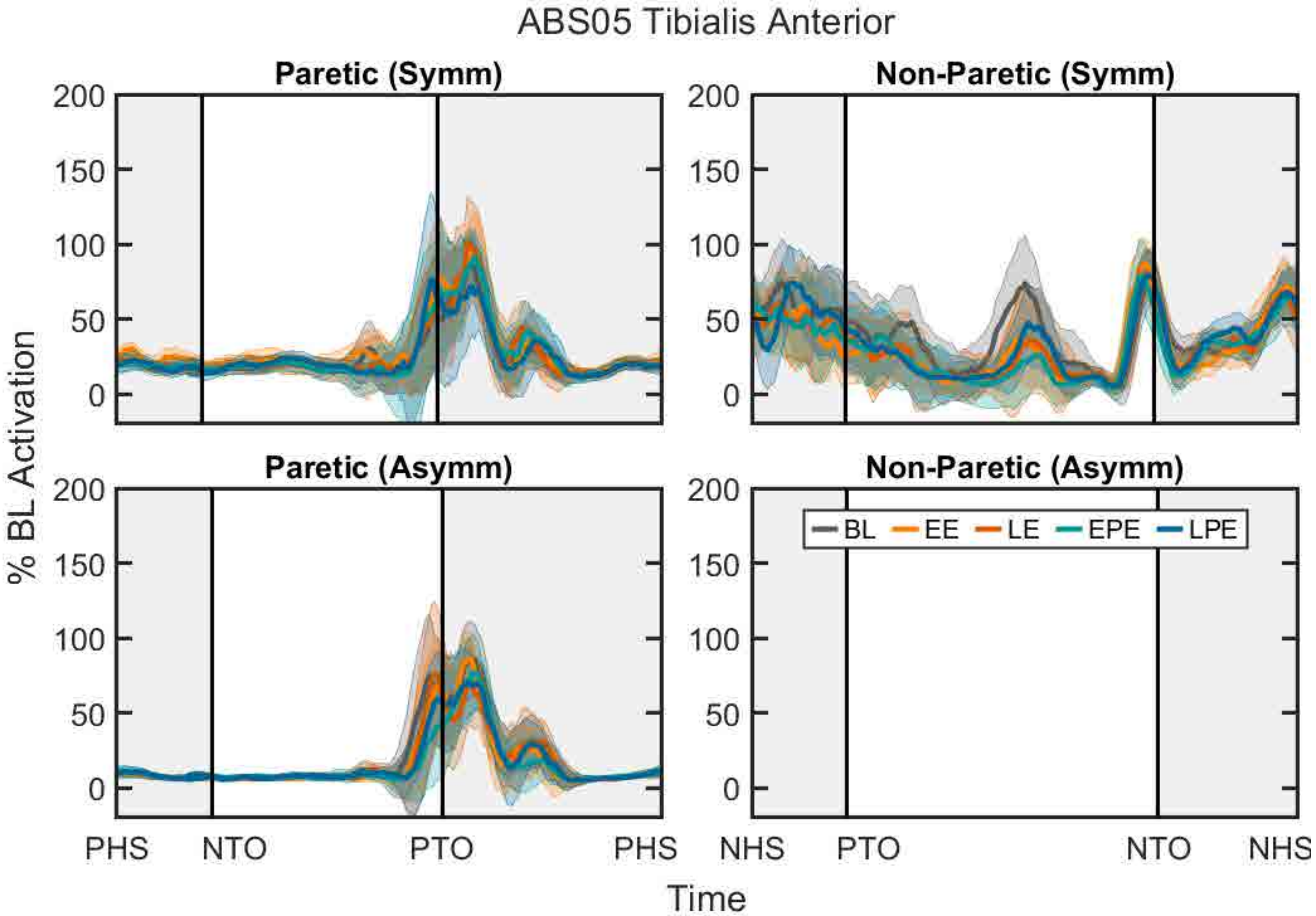
ABS05 Tibialis Anterior
Paretic (Symm)
Non-Paretic (Symm)
Paretic (Asymm)
Non-Paretic (Asymm)
% BL Activation
200
150
100
50
0
BL
EE
LE
EPE
LPE
PHS
NTO
PTO
PHS
NHS
PTO
NTO
NHS
Time

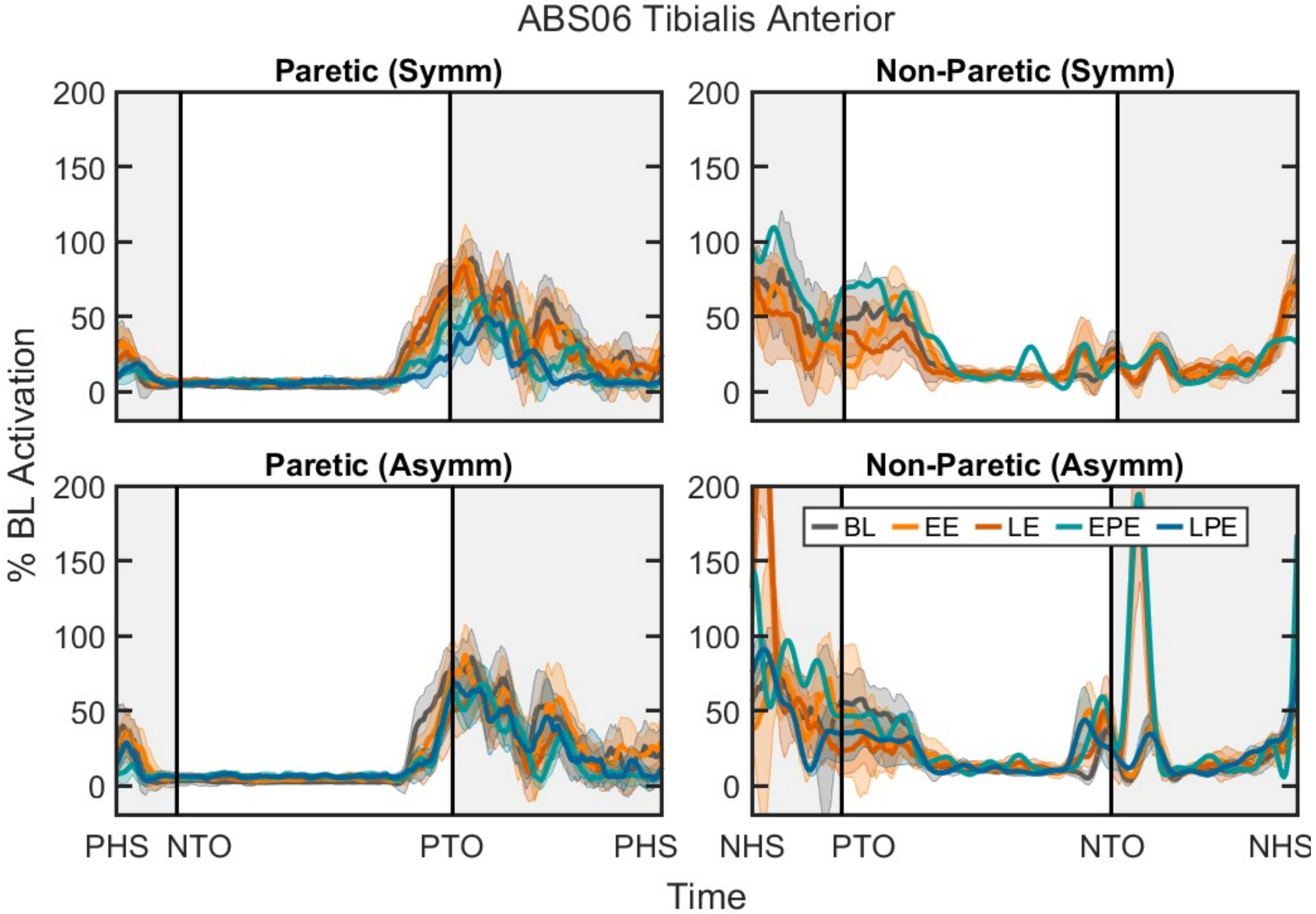
ABS06 Tibialis Anterior
Paretic (Symm)
Non-Paretic (Symm)
Paretic (Asymm)
Non-Paretic (Asymm)
% BL Activation
200
150
100
50
0
BL
EE
LE
EPE
LPE
PHS
NTO
PTO
PHS
NHS
PTO
NTO
NHS
Time

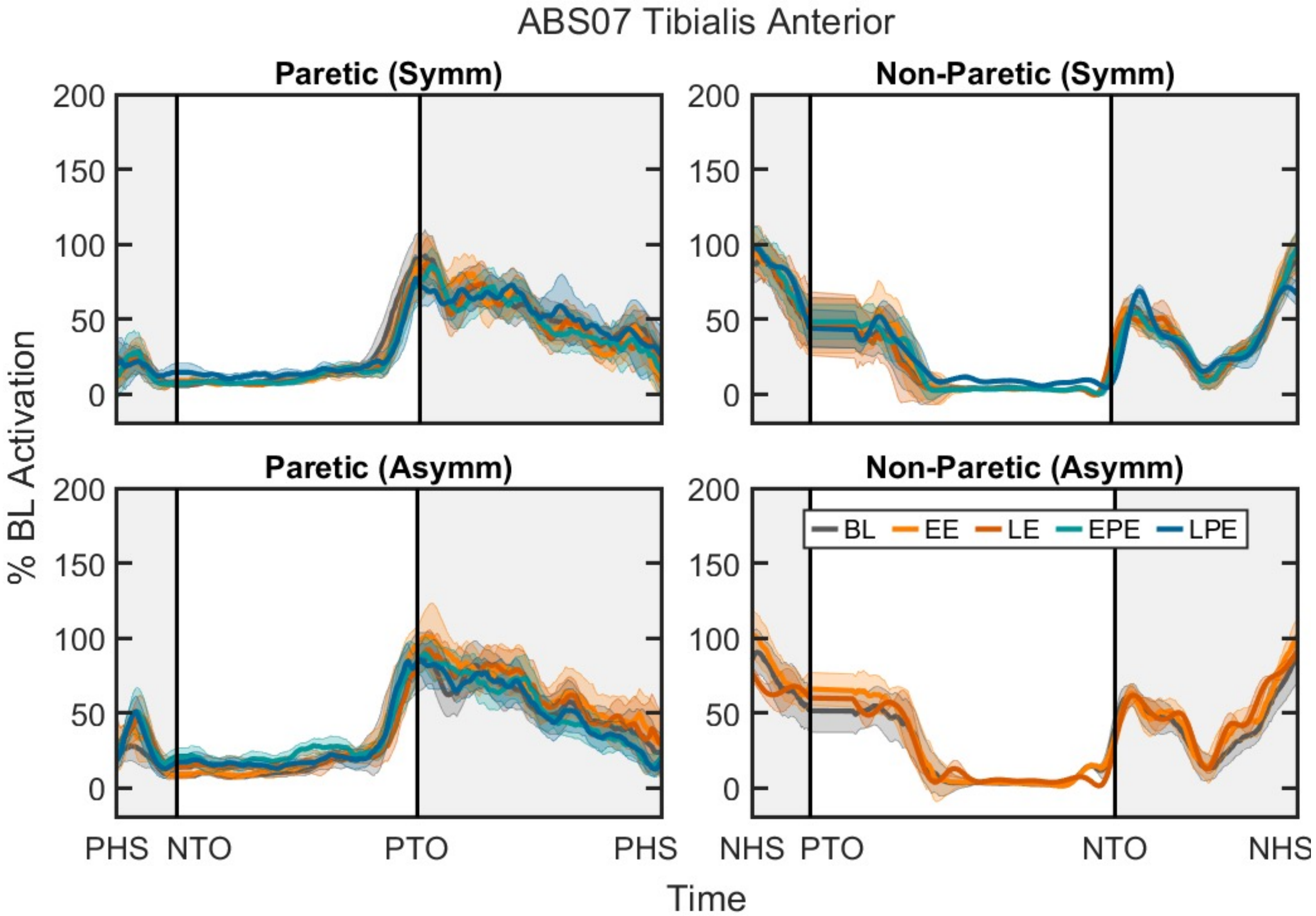
ABS07 Tibialis Anterior
Paretic (Symm)
Non-Paretic (Symm)
Paretic (Asymm)
Non-Paretic (Asymm)
% BL Activation
200
150
100
50
0
BL
EE
LE
EPE
LPE
PHS
NTO
PTO
PHS
NHS
PTO
NTO
NHS
Time

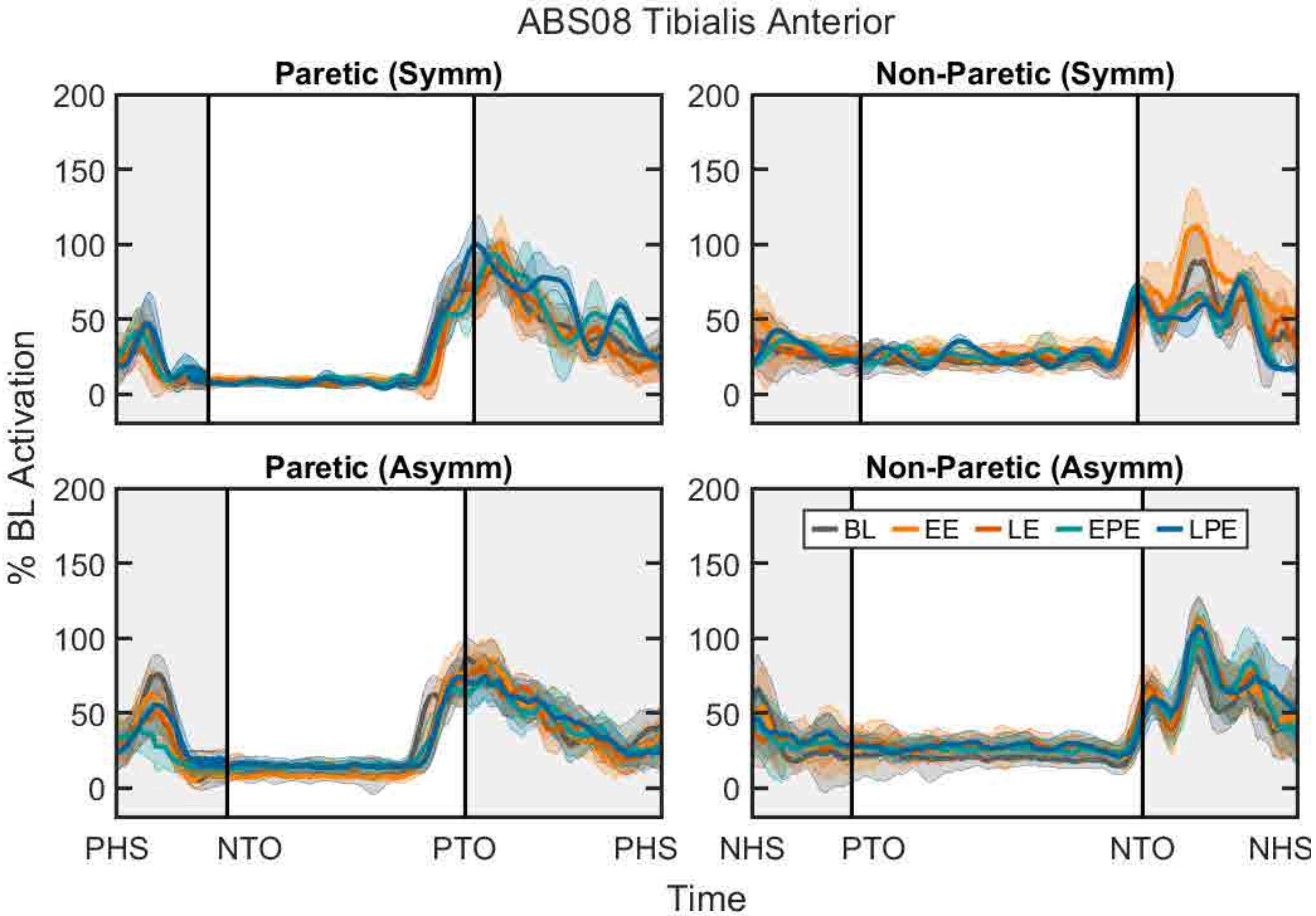

ABS08 Tibialis Anterior
Paretic (Symm)
Non-Paretic (Symm)
Paretic (Asymm)
Non-Paretic (Asymm)
% BL Activation
200
150
100
50
0
BL
EE
LE
EPE
LPE
PHS
NTO
PTO
PHS
NHS
PTO
NTO
NHS
Time

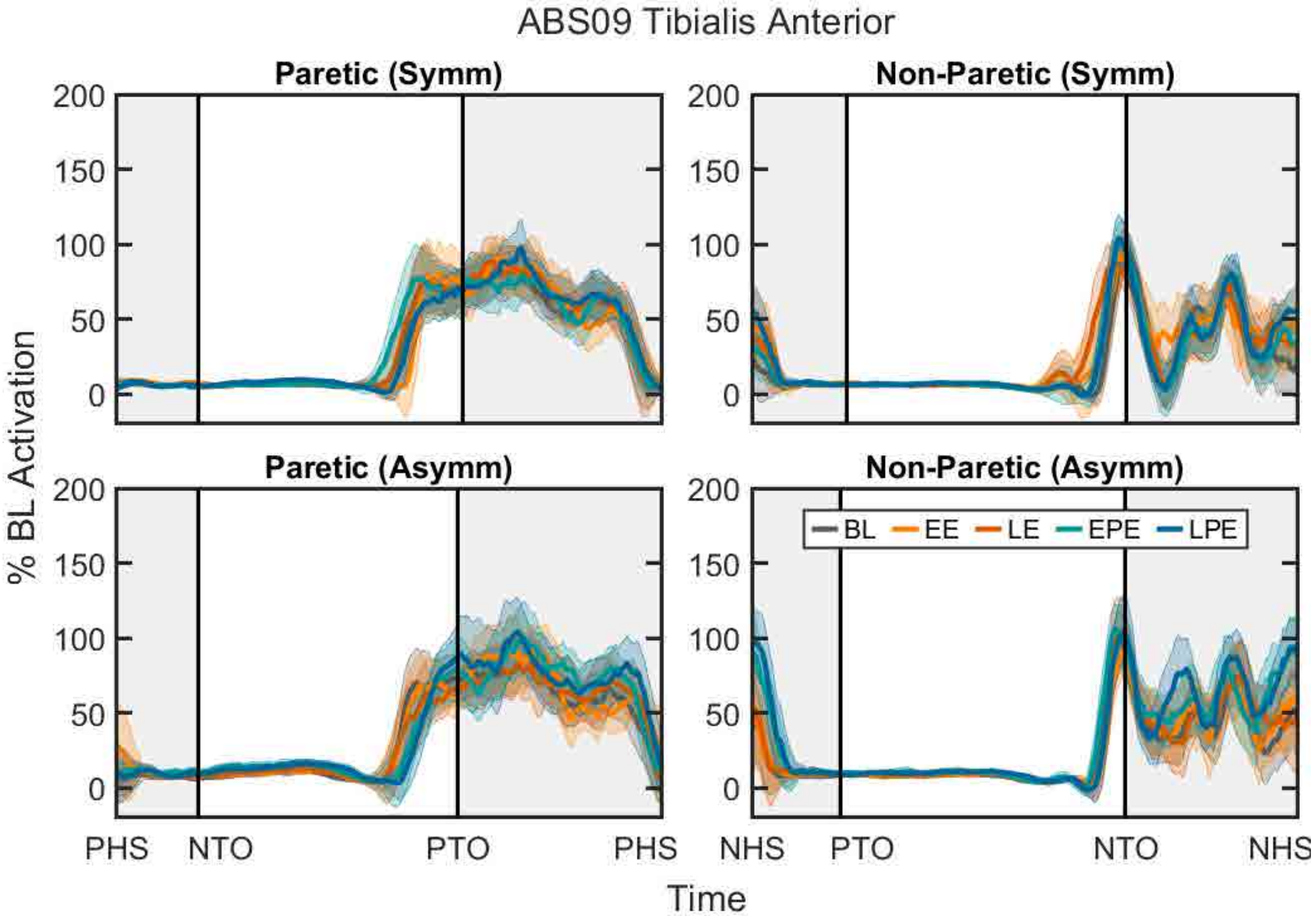
ABS09 Tibialis Anterior
Paretic (Symm)
Non-Paretic (Symm)
Paretic (Asymm)
Non-Paretic (Asymm)
200
150
100
50
0
% BL Activation
BL
EE
LE
EPE
LPE
PHS
NTO
PTO
PHS
NHS
PTO
NTO
NHS
Time

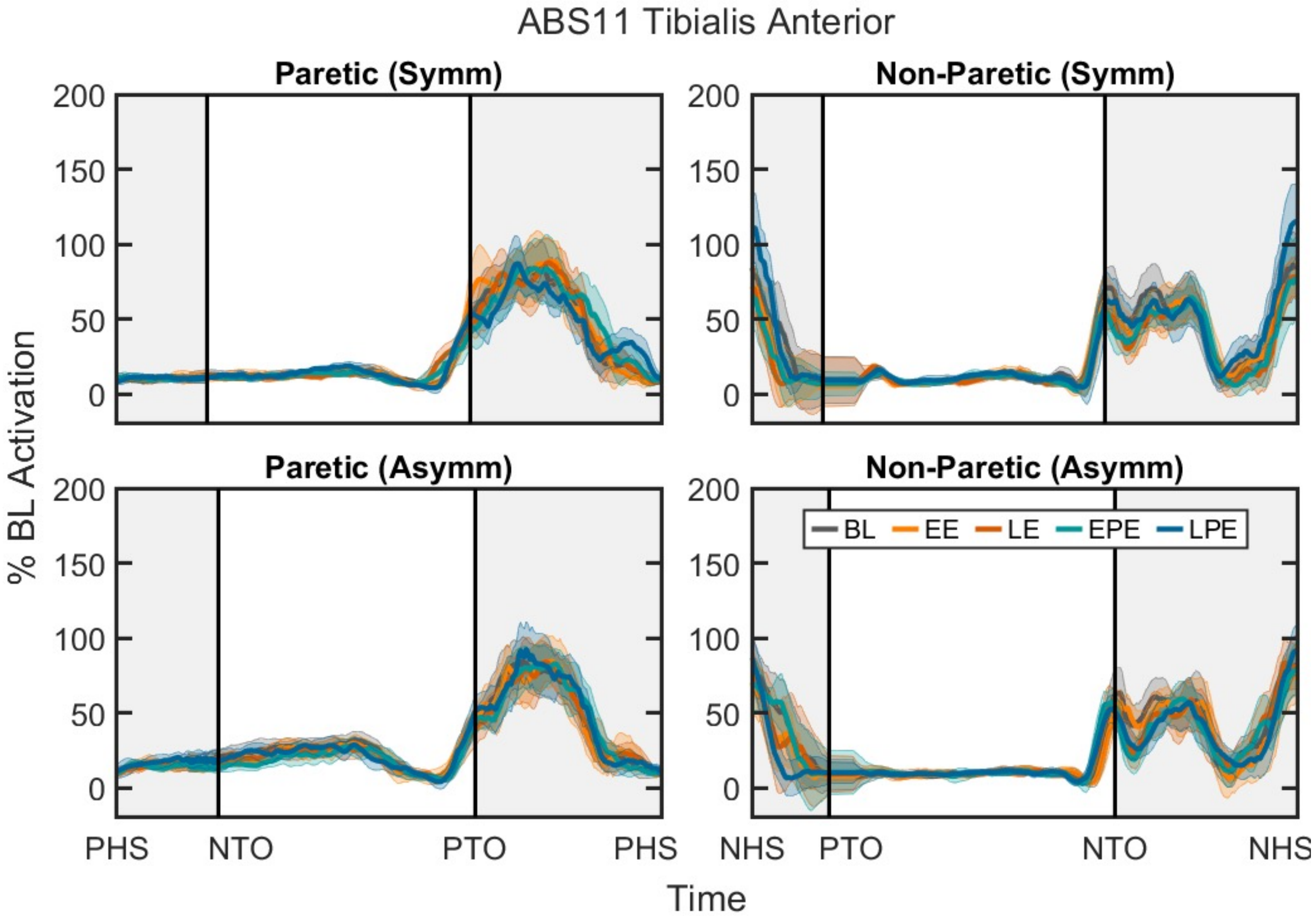
ABS11 Tibialis Anterior
Paretic (Symm)
Non-Paretic (Symm)
Paretic (Asymm)
Non-Paretic (Asymm)
% BL Activation
200
150
100
50
0
BL
EE
LE
EPE
LPE
PHS
NTO
PTO
PHS
NHS
PTO
NTO
NHS
Time

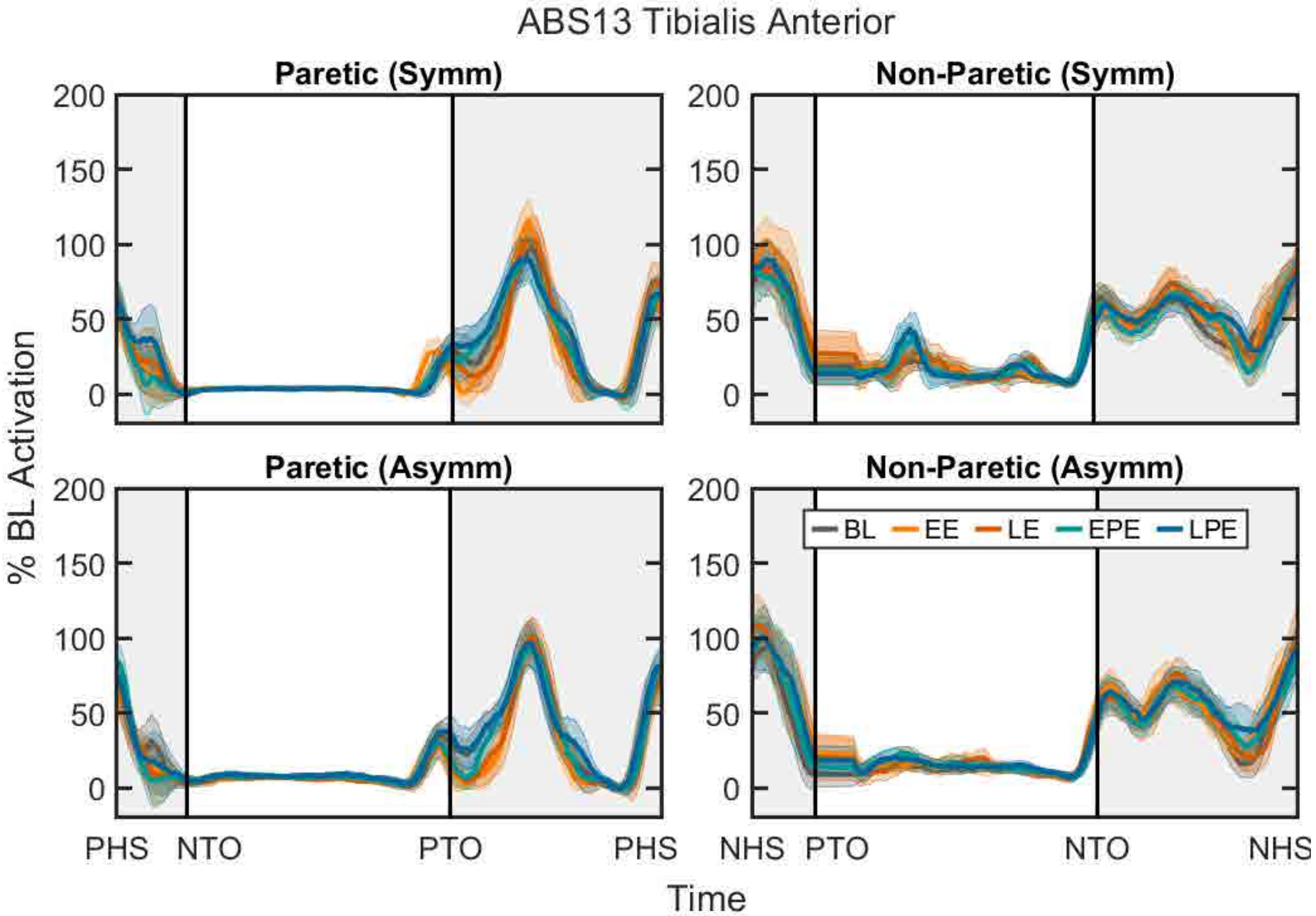
ABS13 Tibialis Anterior
Paretic (Symm)
Non-Paretic (Symm)
Paretic (Asymm)
Non-Paretic (Asymm)
% BL Activation
200
150
100
50
0
BL
EE
LE
EPE
LPE
PHS
NTO
PTO
PHS
NHS
PTO
NTO
NHS
Time

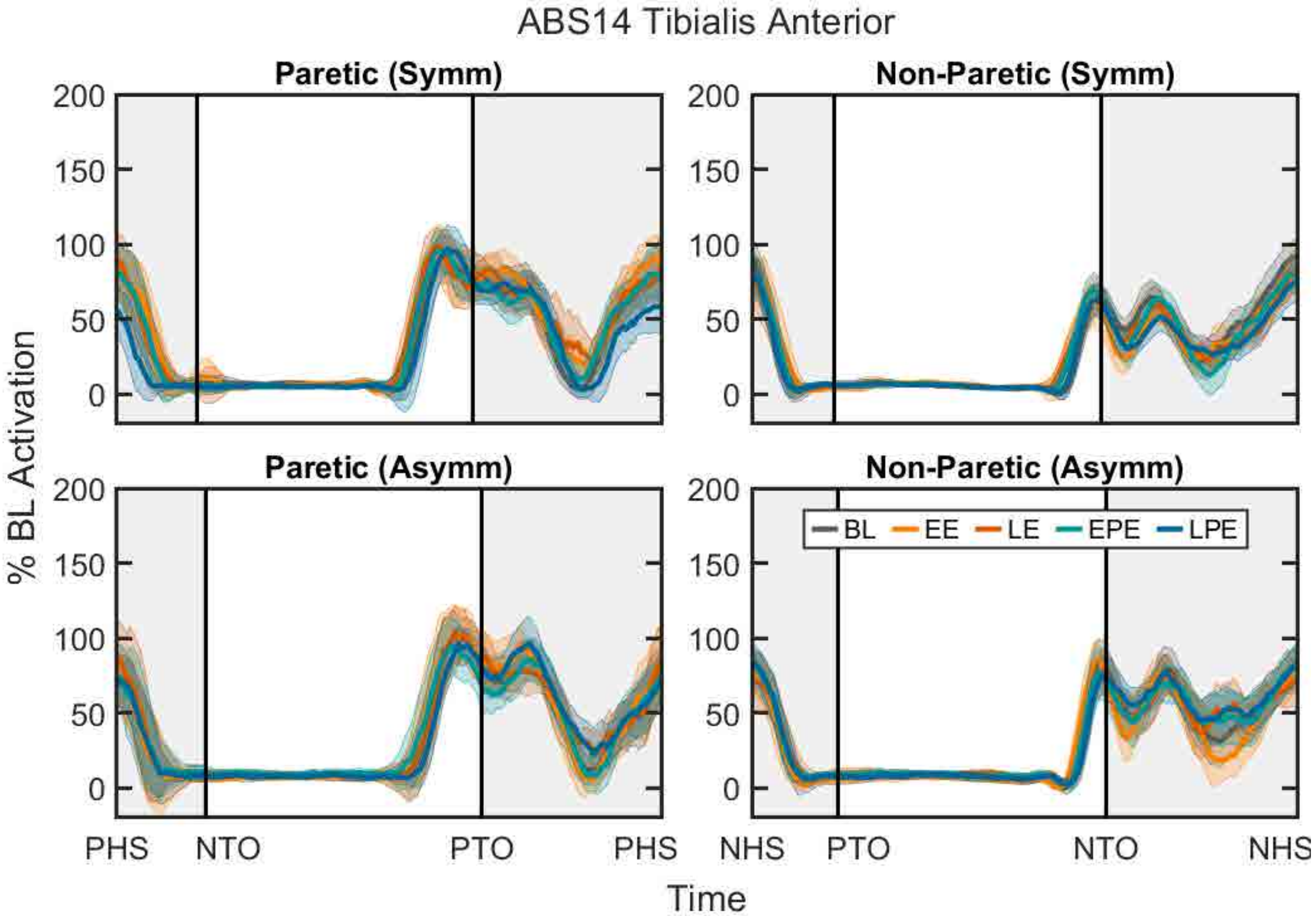

ABS14 Tibialis Anterior
Paretic (Symm)
Non-Paretic (Symm)
Paretic (Asymm)
Non-Paretic (Asymm)
% BL Activation
200
150
100
50
0
BL
EE
LE
EPE
LPE
PHS
NTO
PTO
PHS
NHS
PTO
NTO
NHS
Time

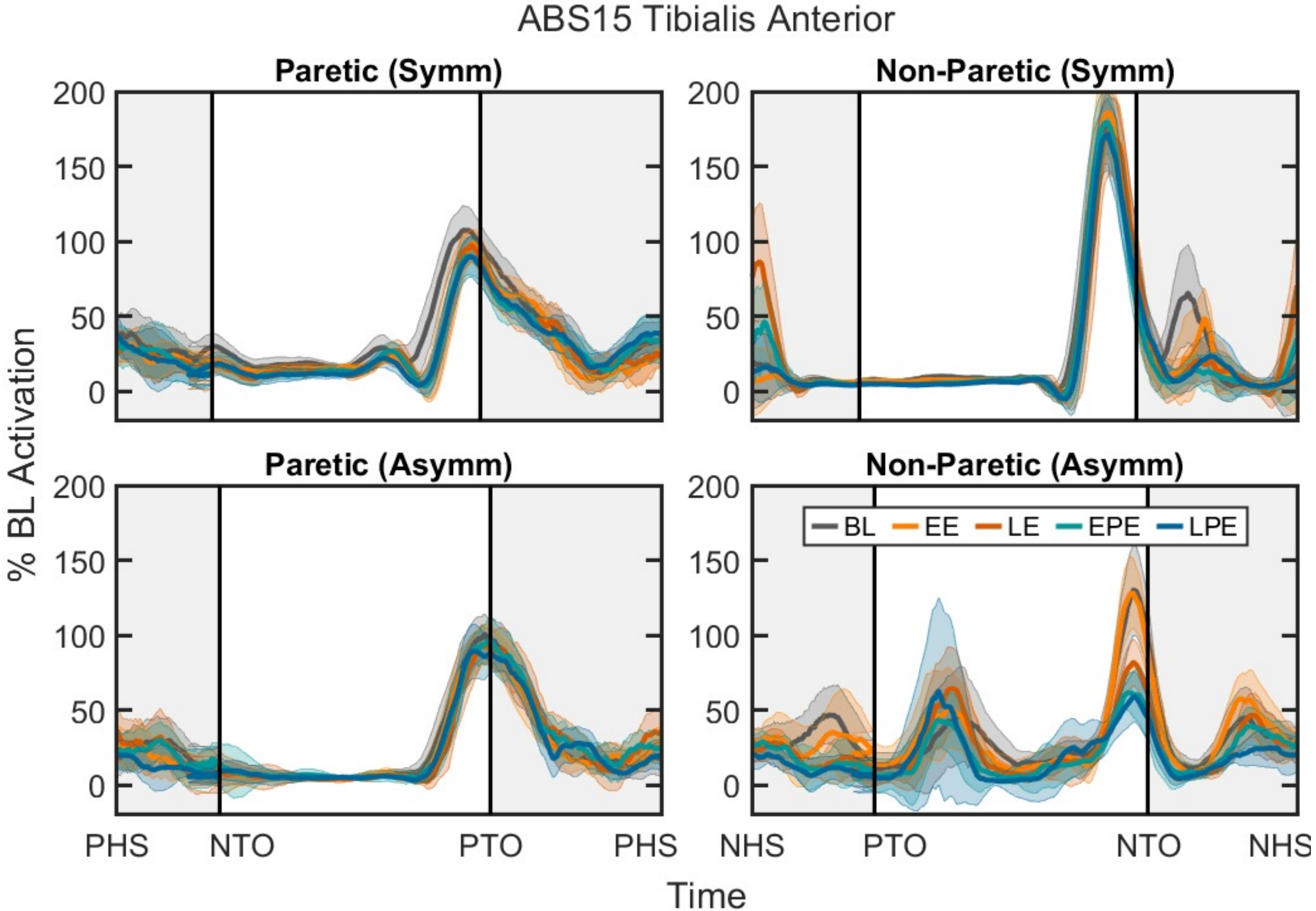
ABS15 Tibialis Anterior
Paretic (Symm)
Non-Paretic (Symm)
Paretic (Asymm)
Non-Paretic (Asymm)
% BL Activation
200
150
100
50
0
BL
EE
LE
EPE
LPE
PHS
NTO
PTO
PHS
NHS
PTO
NTO
NHS
Time

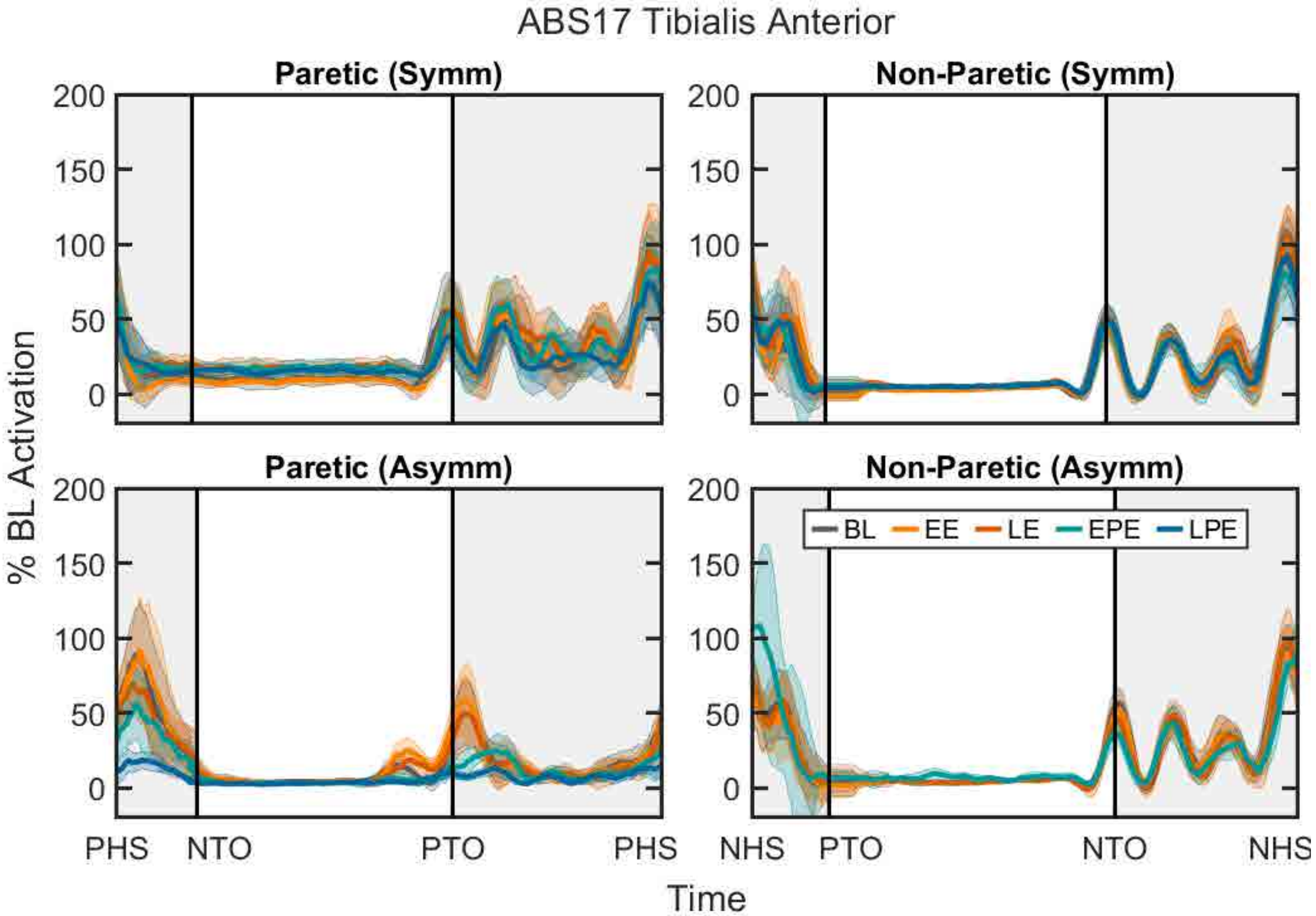

ABS17 Tibialis Anterior
Paretic (Symm)
Non-Paretic (Symm)
Paretic (Asymm)
Non-Paretic (Asymm)
% BL Activation
200
150
100
50
0
BL
EE
LE
EPE
LPE
PHS
NTO
PTO
PHS
NHS
PTO
NTO
NHS
Time

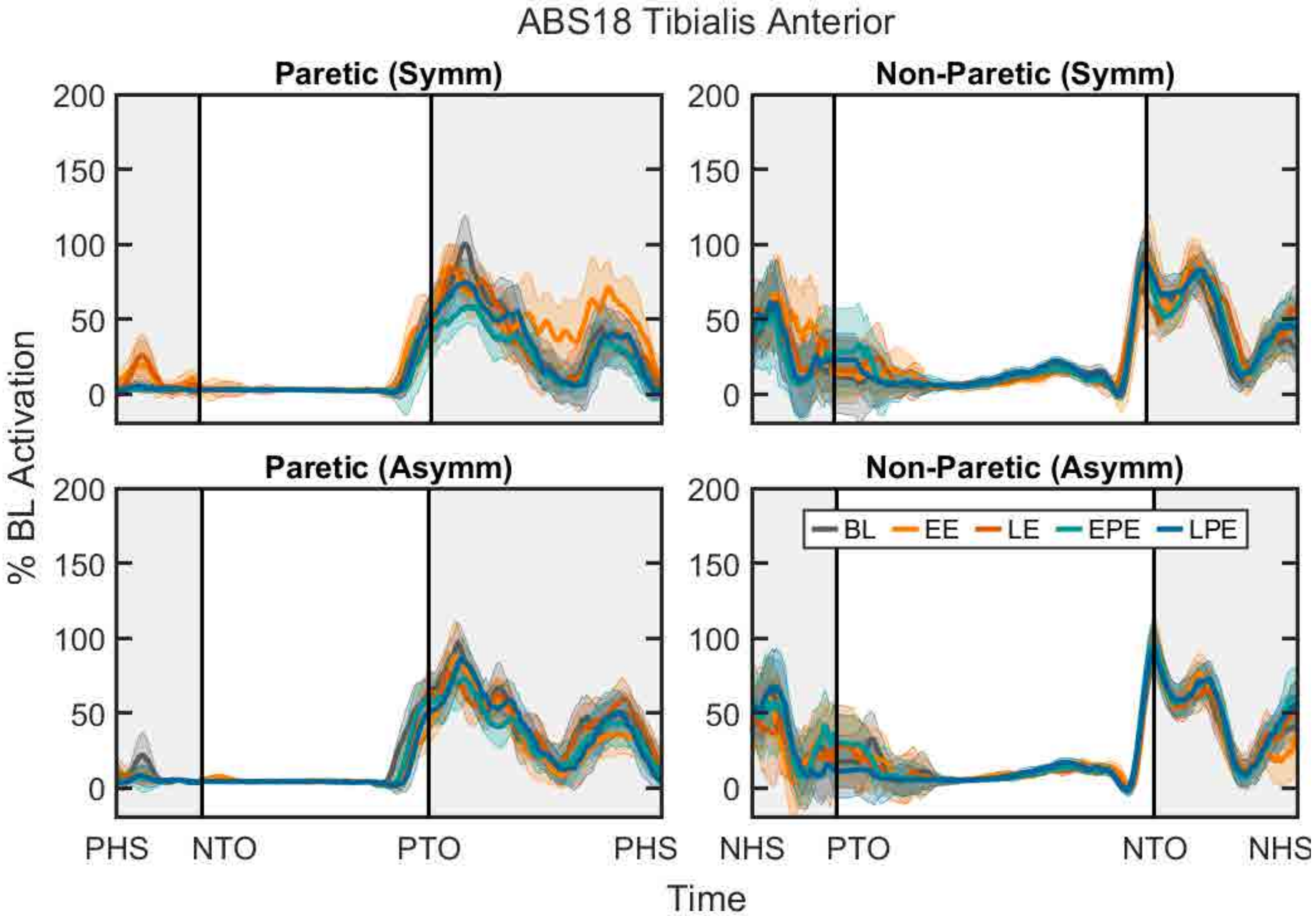

ABS18 Tibialis Anterior
Paretic (Symm)
Non-Paretic (Symm)
Paretic (Asymm)
Non-Paretic (Asymm)
% BL Activation
200
150
100
50
0
BL
EE
LE
EPE
LPE
PHS
NTO
PTO
PHS
NHS
PTO
NTO
NHS
Time

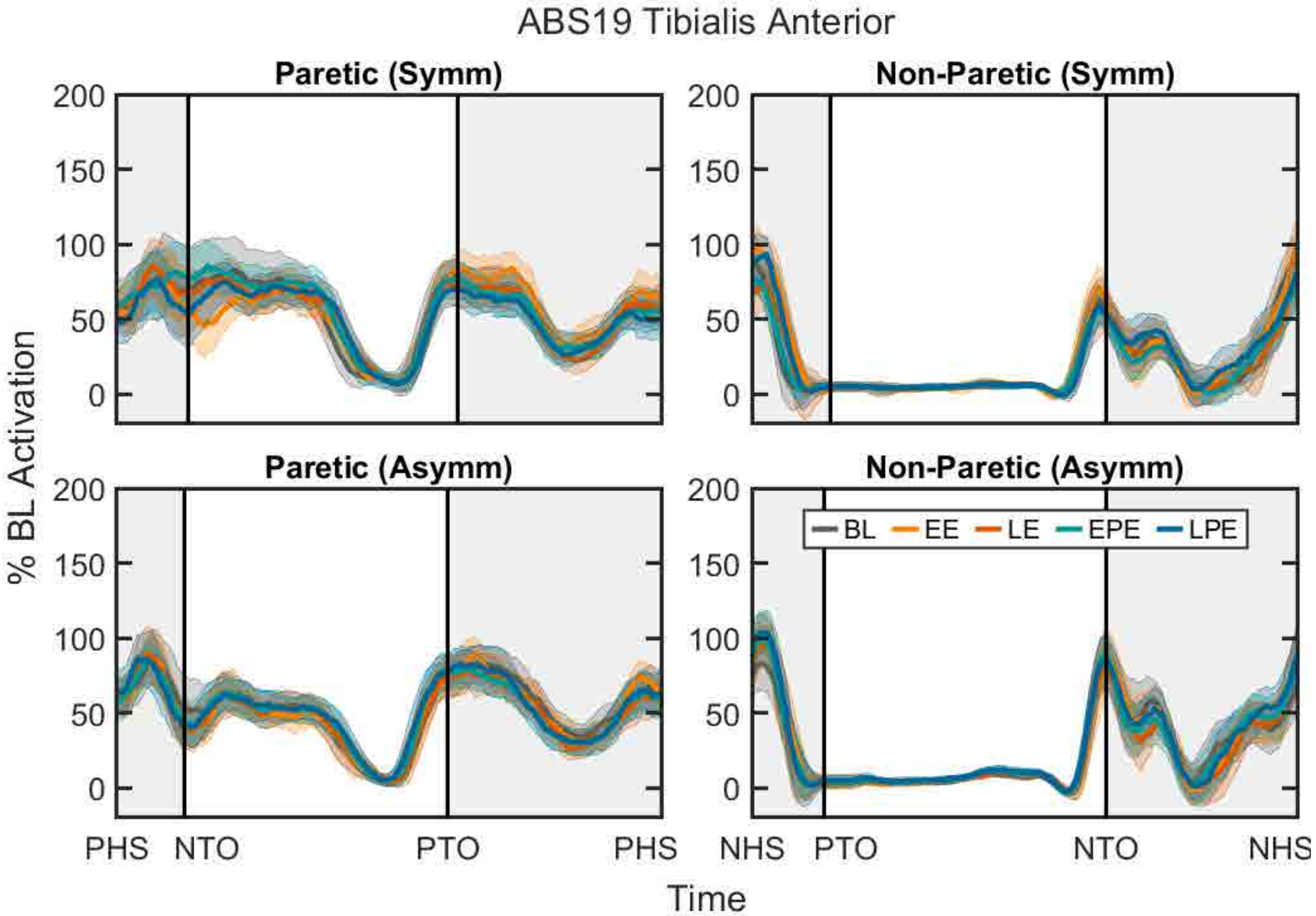

ABS19 Tibialis Anterior
Paretic (Symm)
Non-Paretic (Symm)
Paretic (Asymm)
Non-Paretic (Asymm)
% BL Activation
200
150
100
50
0
BL
EE
LE
EPE
LPE
PHS
NTO
PTO
PHS
NHS
PTO
NTO
NHS
Time

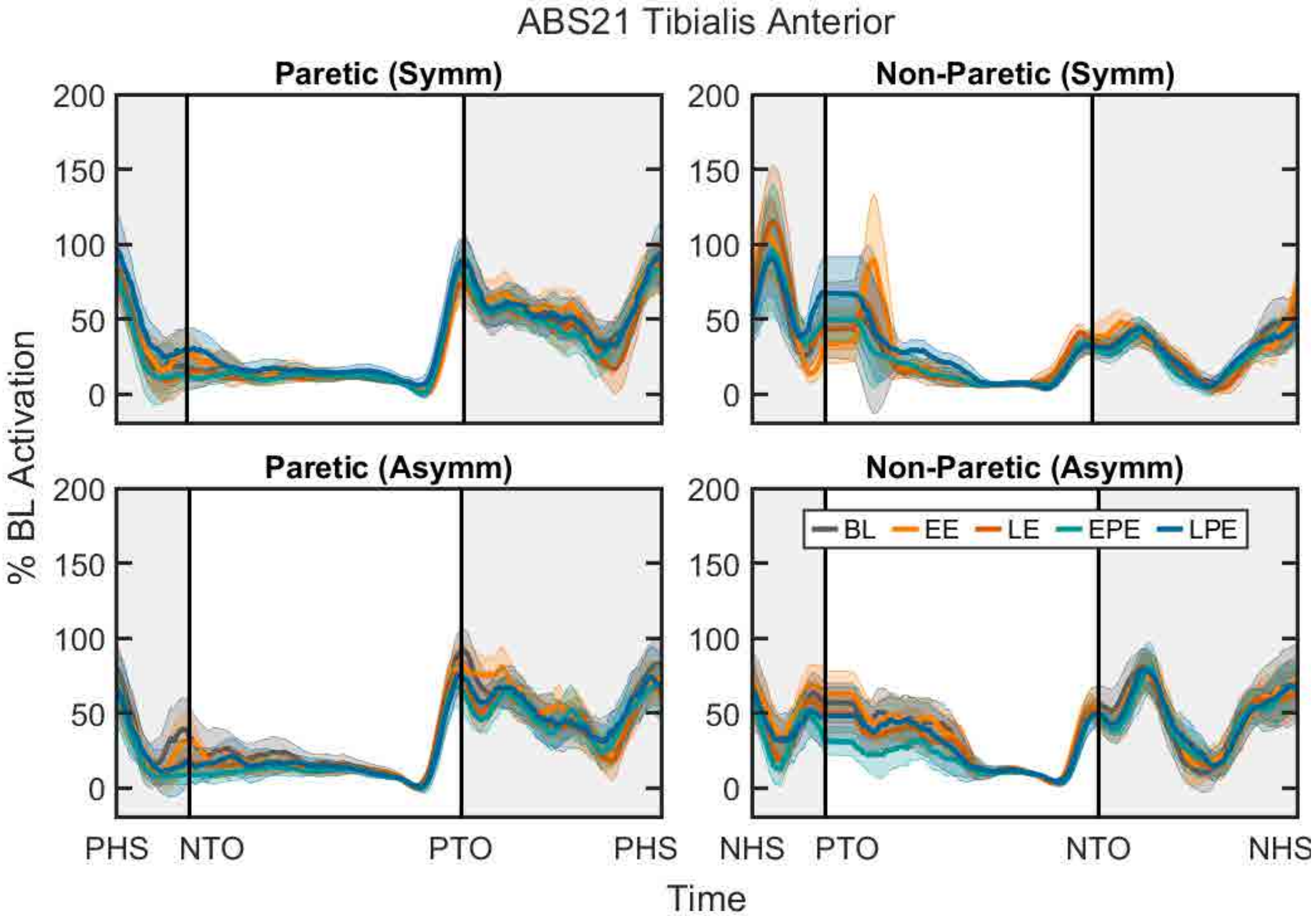
ABS21 Tibialis Anterior
Paretic (Symm)
Non-Paretic (Symm)
Paretic (Asymm)
Non-Paretic (Asymm)
% BL Activation
200
150
100
50
0
BL
EE
LE
EPE
LPE
PHS
NTO
PTO
PHS
NHS
PTO
NTO
NHS
Time

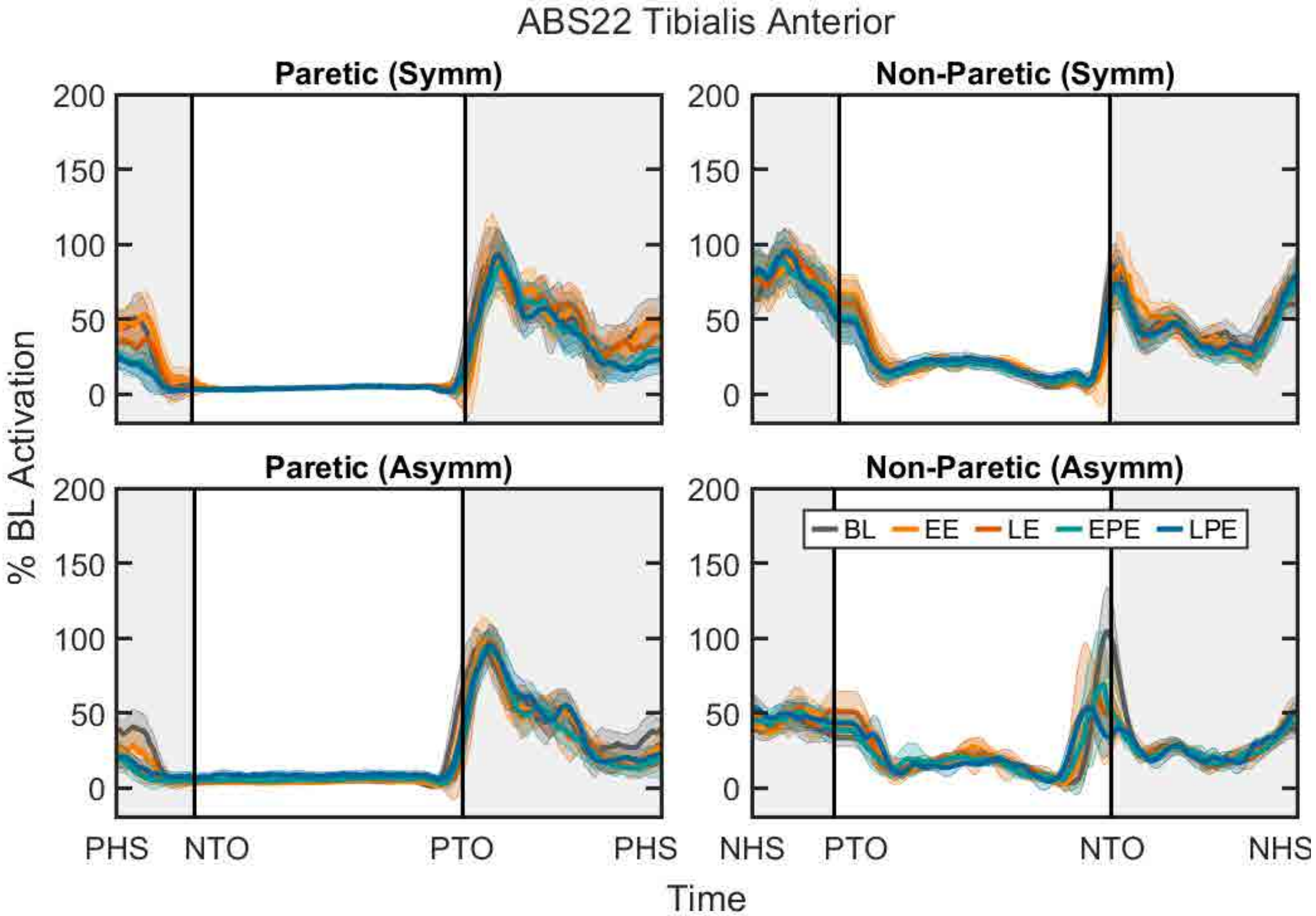
ABS22 Tibialis Anterior
Paretic (Symm)
Non-Paretic (Symm)
Paretic (Asymm)
Non-Paretic (Asymm)
% BL Activation
200
150
100
50
0
BL
EE
LE
EPE
LPE
PHS
NTO
PTO
PHS
NHS
PTO
NTO
NHS
Time

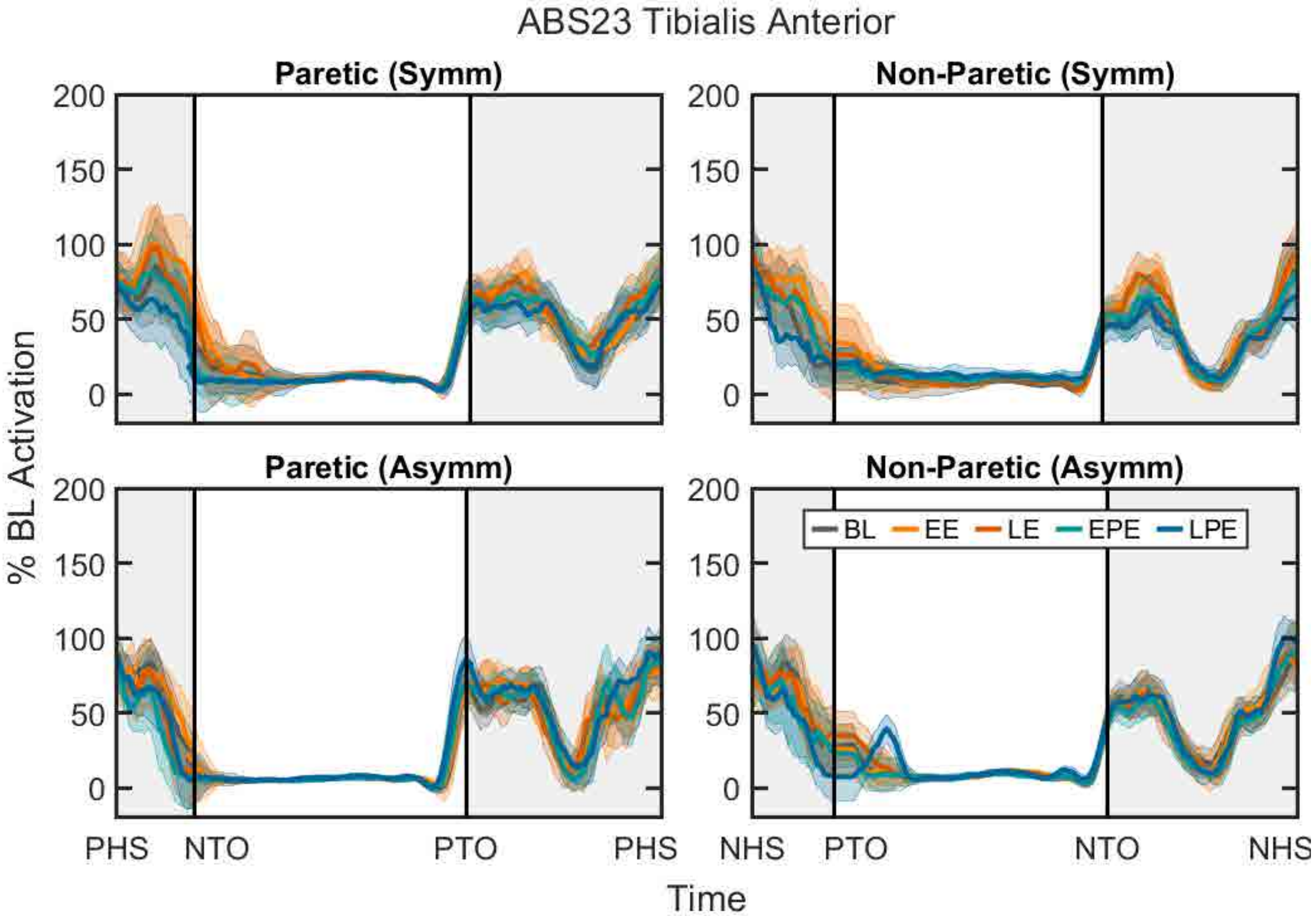

ABS23 Tibialis Anterior
Paretic (Symm)
Non-Paretic (Symm)
Paretic (Asymm)
Non-Paretic (Asymm)
% BL Activation
200
150
100
50
0
BL
EE
LE
EPE
LPE
PHS
NTO
PTO
PHS
NHS
PTO
NTO
NHS
Time

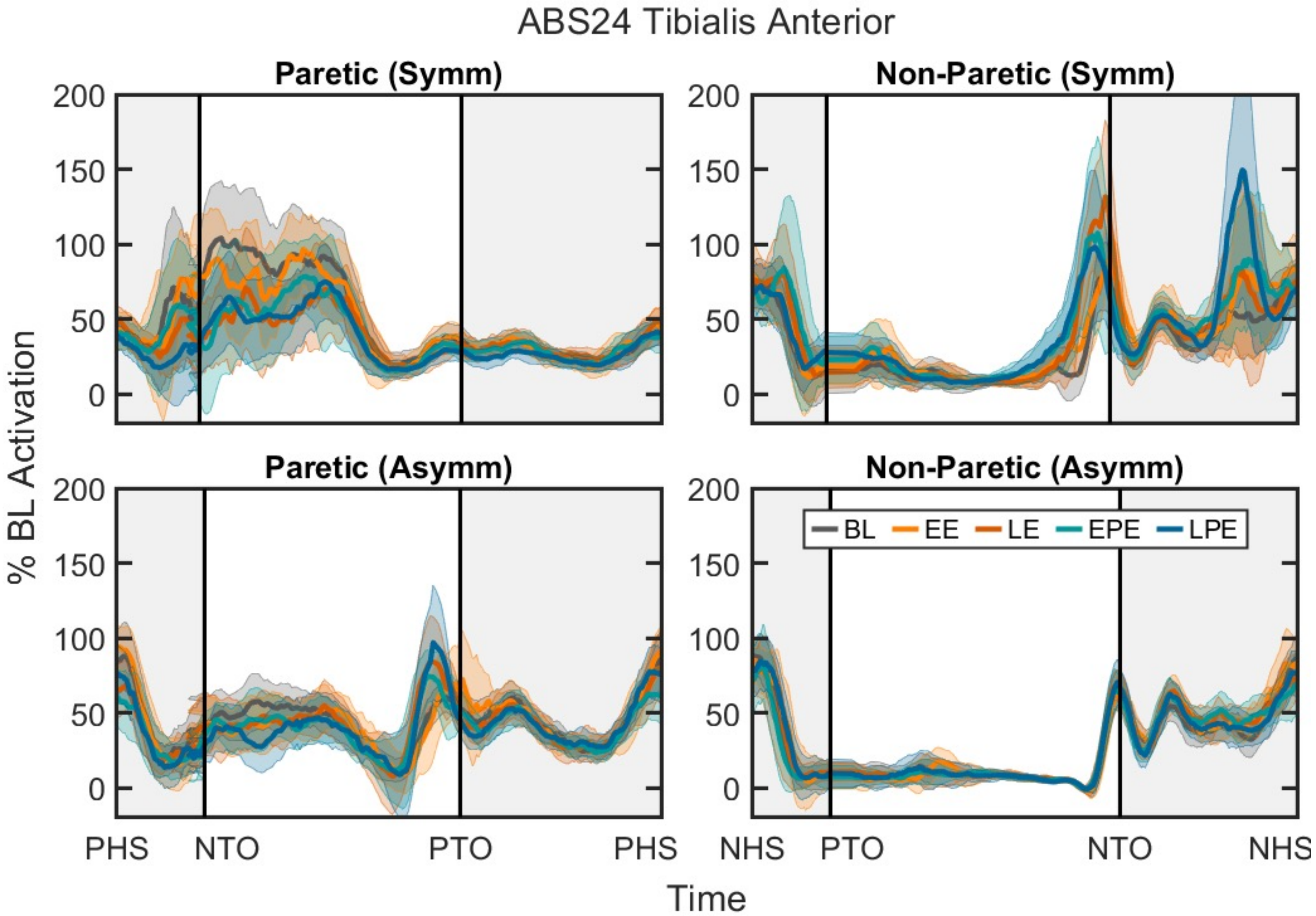

ABS24 Tibialis Anterior
Paretic (Symm)
Non-Paretic (Symm)
Paretic (Asymm)
Non-Paretic (Asymm)
% BL Activation
200
150
100
50
0
BL
EE
LE
EPE
LPE
PHS
NTO
PTO
PHS
NHS
PTO
NTO
NHS
Time

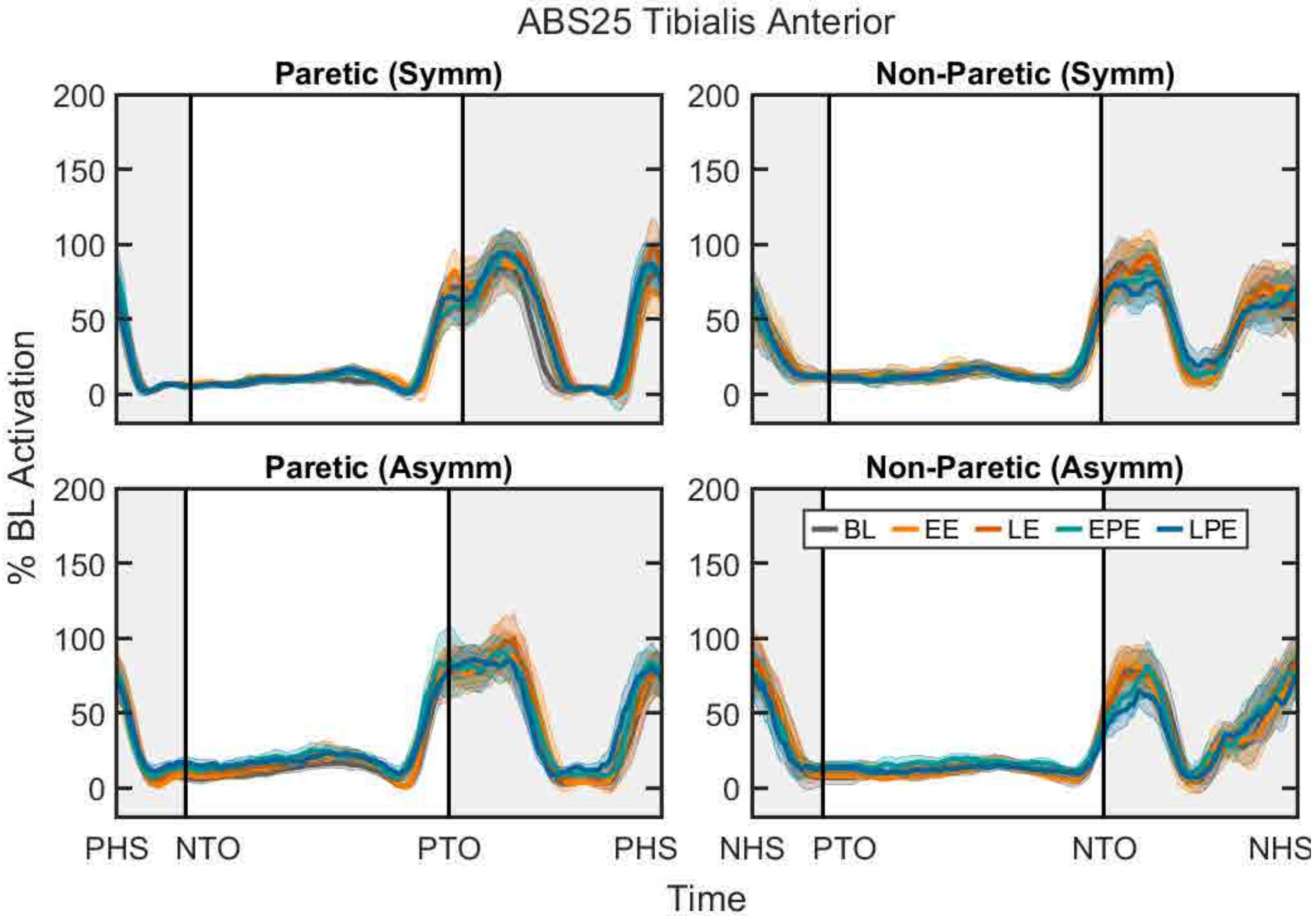

ABS25 Tibialis Anterior
Paretic (Symm)
Non-Paretic (Symm)
Paretic (Asymm)
Non-Paretic (Asymm)
% BL Activation
200
150
100
50
0
BL
EE
LE
EPE
LPE
PHS
NTO
PTO
PHS
NHS
PTO
NTO
NHS
Time

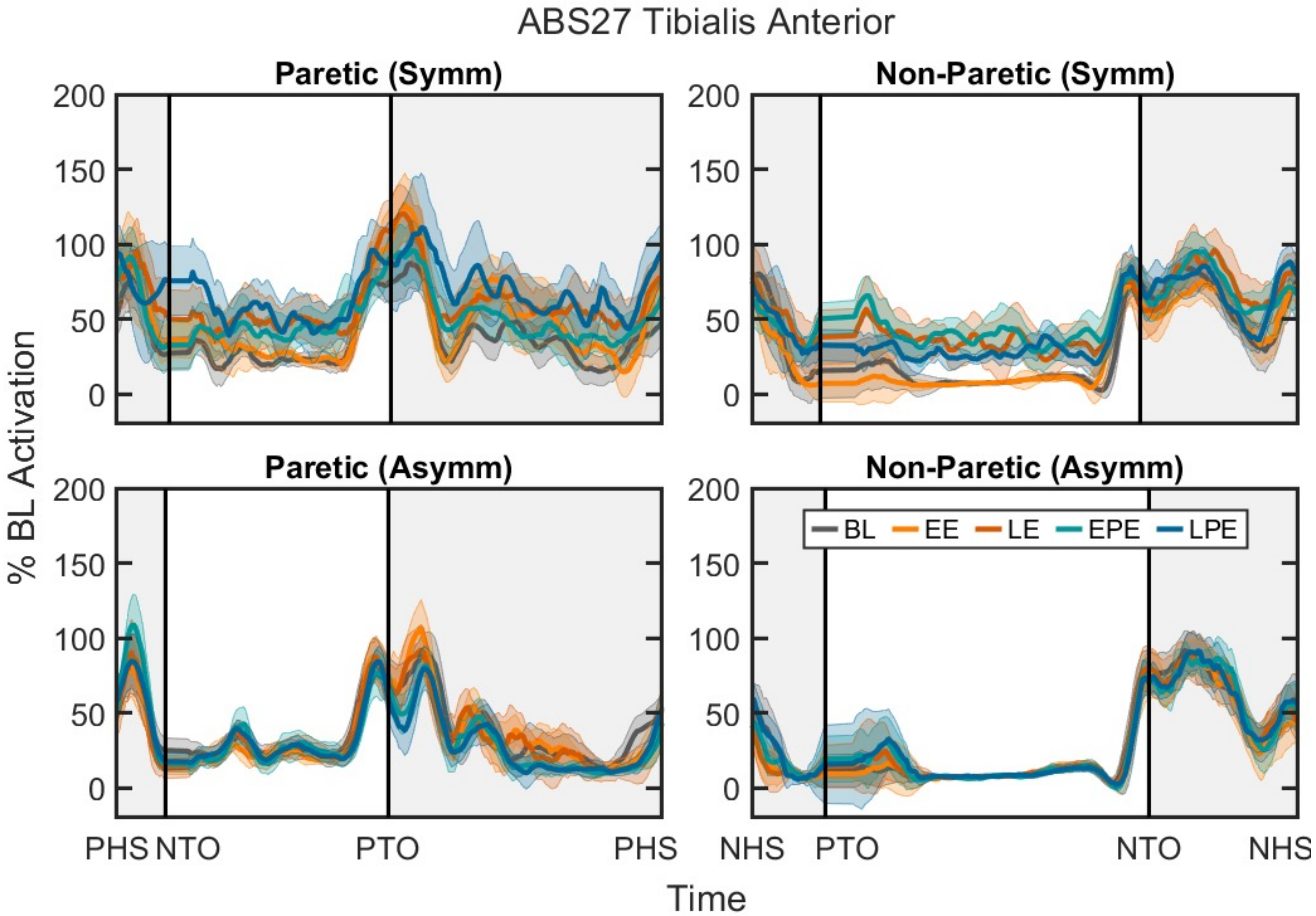

ABS27 Tibialis Anterior
Paretic (Symm)
Non-Paretic (Symm)
Paretic (Asymm)
Non-Paretic (Asymm)
% BL Activation
200
150
100
50
0
BL
EE
LE
EPE
LPE
PHS
NTO
PTO
PHS
NHS
PTO
NTO
NHS
Time

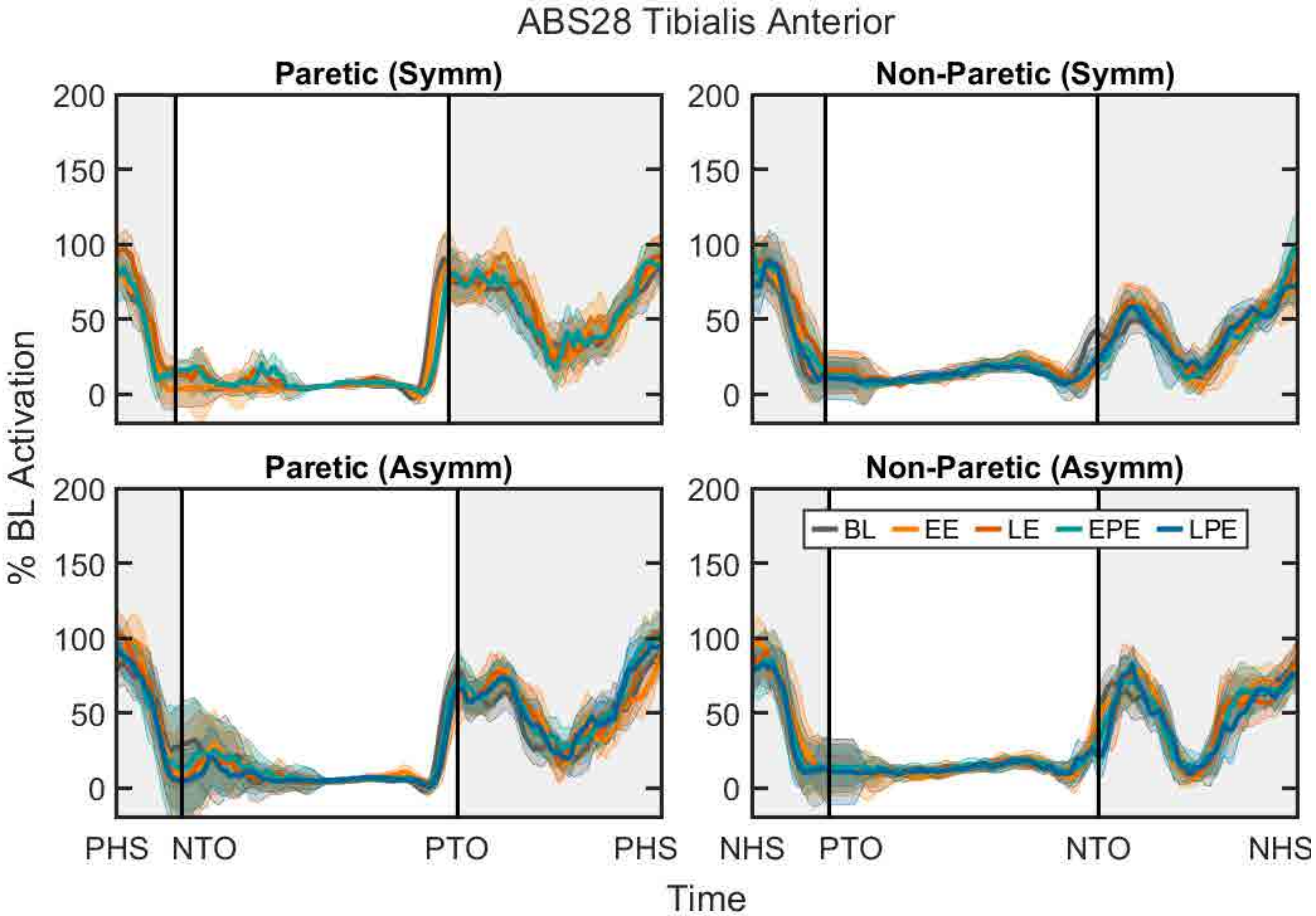

ABS28 Tibialis Anterior
Paretic (Symm)
Non-Paretic (Symm)
Paretic (Asymm)
Non-Paretic (Asymm)
% BL Activation
200
150
100
50
0
BL
EE
LE
EPE
LPE
PHS
NTO
PTO
PHS
NHS
PTO
NTO
NHS
Time

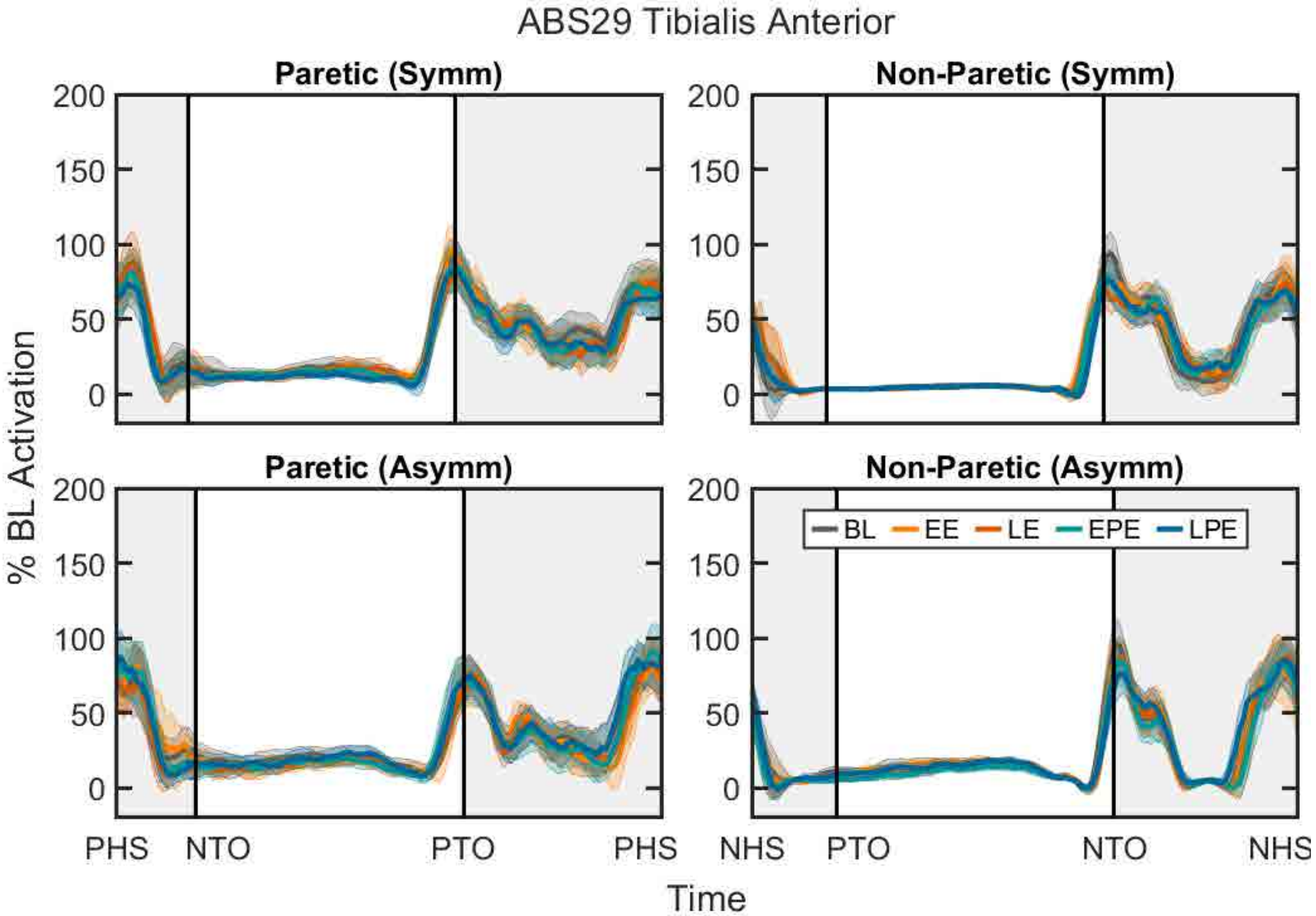

ABS29 Tibialis Anterior
Paretic (Symm)
Non-Paretic (Symm)
Paretic (Asymm)
Non-Paretic (Asymm)
% BL Activation
200
150
100
50
0
BL
EE
LE
EPE
LPE
PHS
NTO
PTO
PHS
NHS
PTO
NTO
NHS
Time

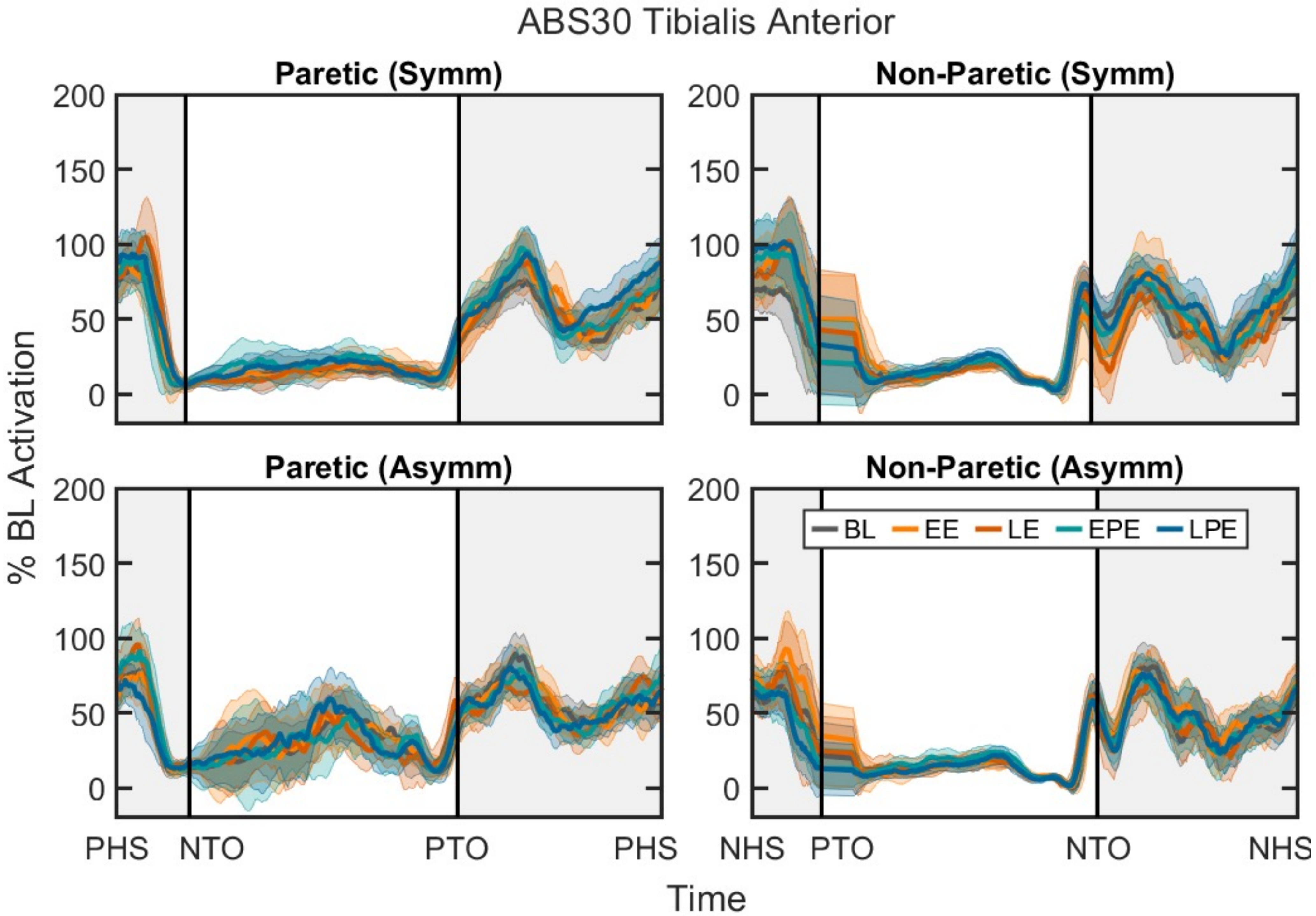
ABS30 Tibialis Anterior
Paretic (Symm)
Non-Paretic (Symm)
Paretic (Asymm)
Non-Paretic (Asymm)
% BL Activation
200
150
100
50
0
BL
EE
LE
EPE
LPE
PHS
NTO
PTO
PHS
NHS
PTO
NTO
NHS
Time

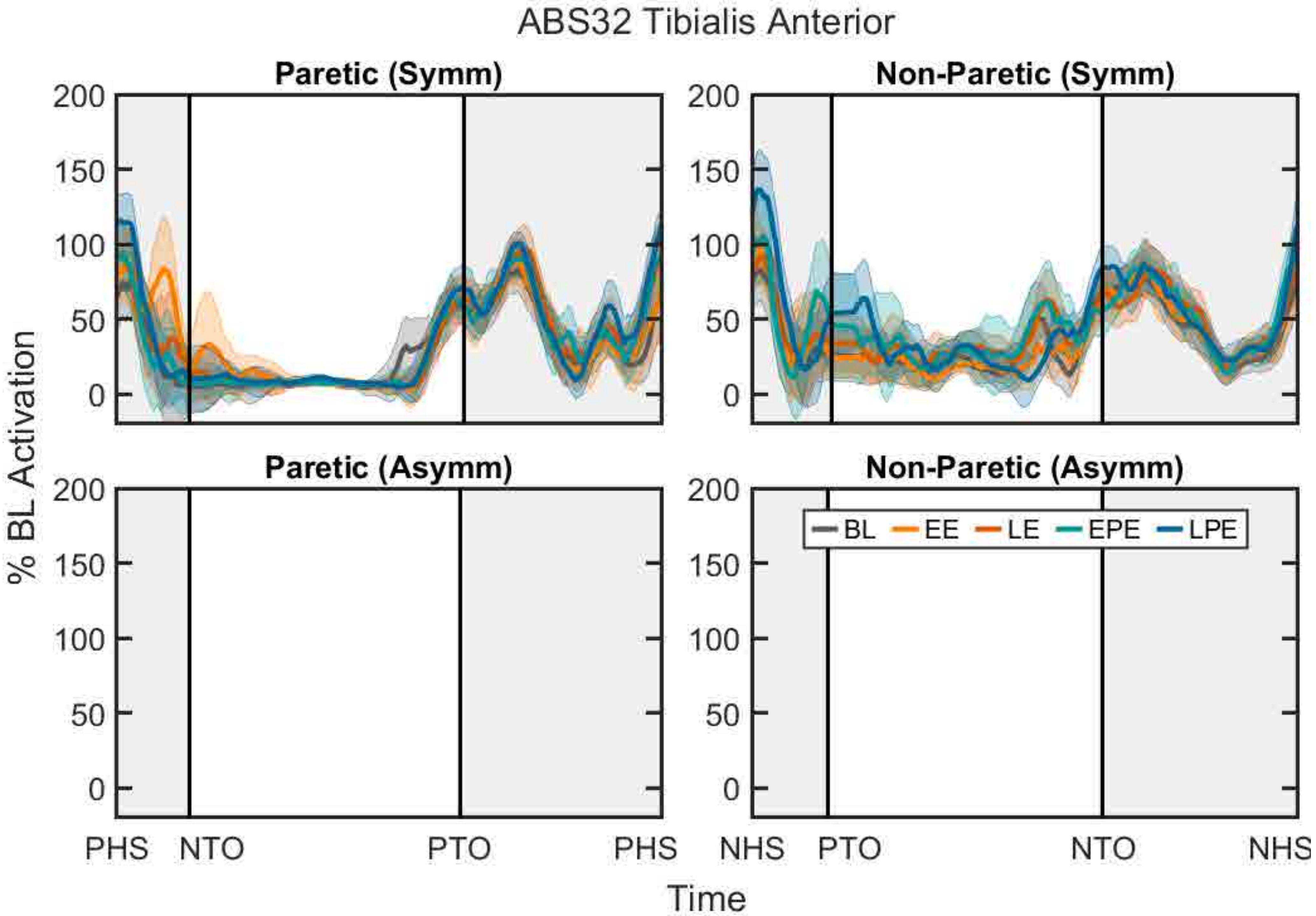
ABS32 Tibialis Anterior
Paretic (Symm)
Non-Paretic (Symm)
Paretic (Asymm)
Non-Paretic (Asymm)
% BL Activation
200
150
100
50
0
BL
EE
LE
EPE
LPE
PHS
NTO
PTO
PHS
NHS
PTO
NTO
NHS
Time

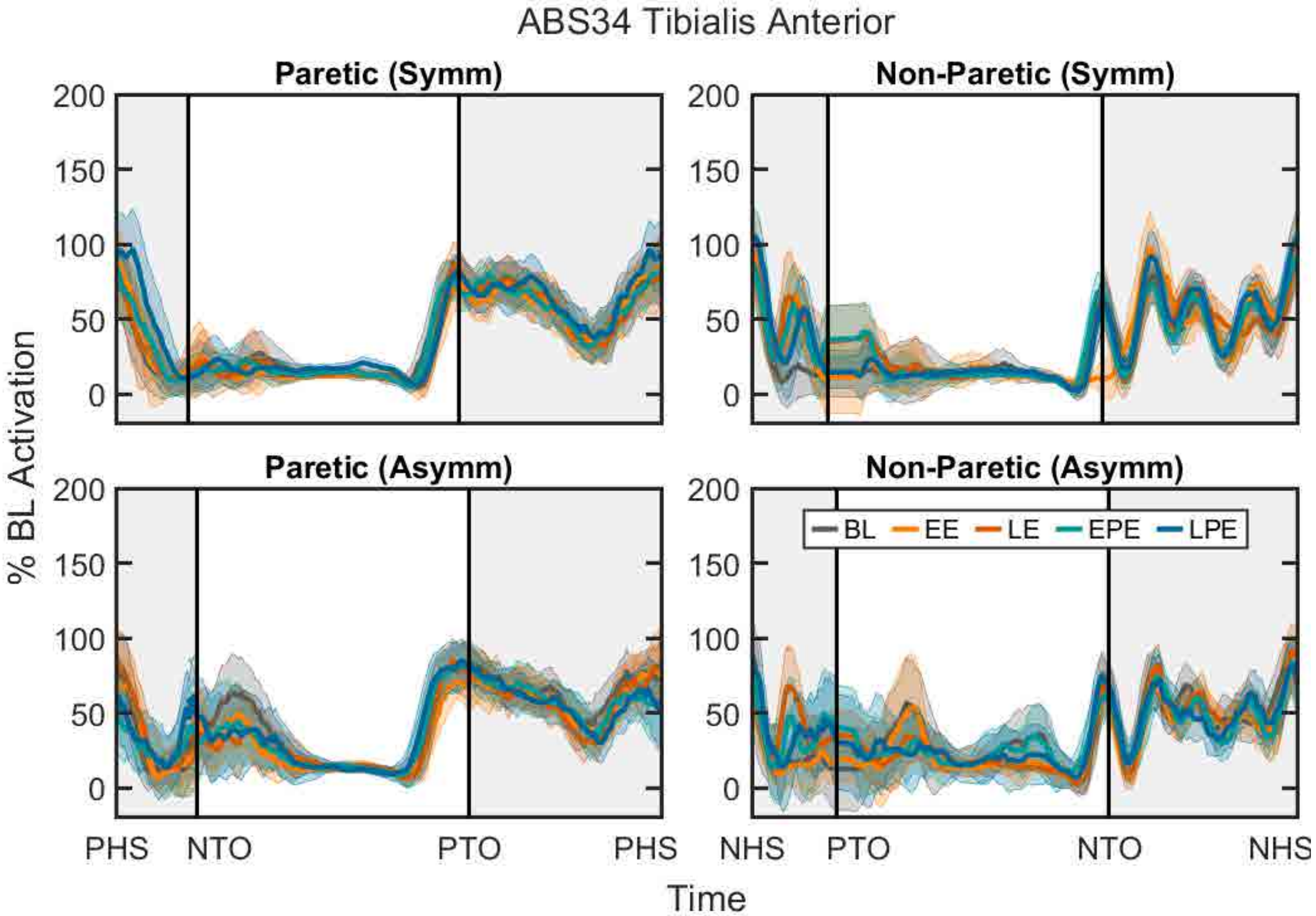
ABS34 Tibialis Anterior
Paretic (Symm)
Non-Paretic (Symm)
Paretic (Asymm)
Non-Paretic (Asymm)
% BL Activation
200
150
100
50
0
BL
EE
LE
EPE
LPE
PHS
NTO
PTO
PHS
NHS
PTO
NTO
NHS
Time

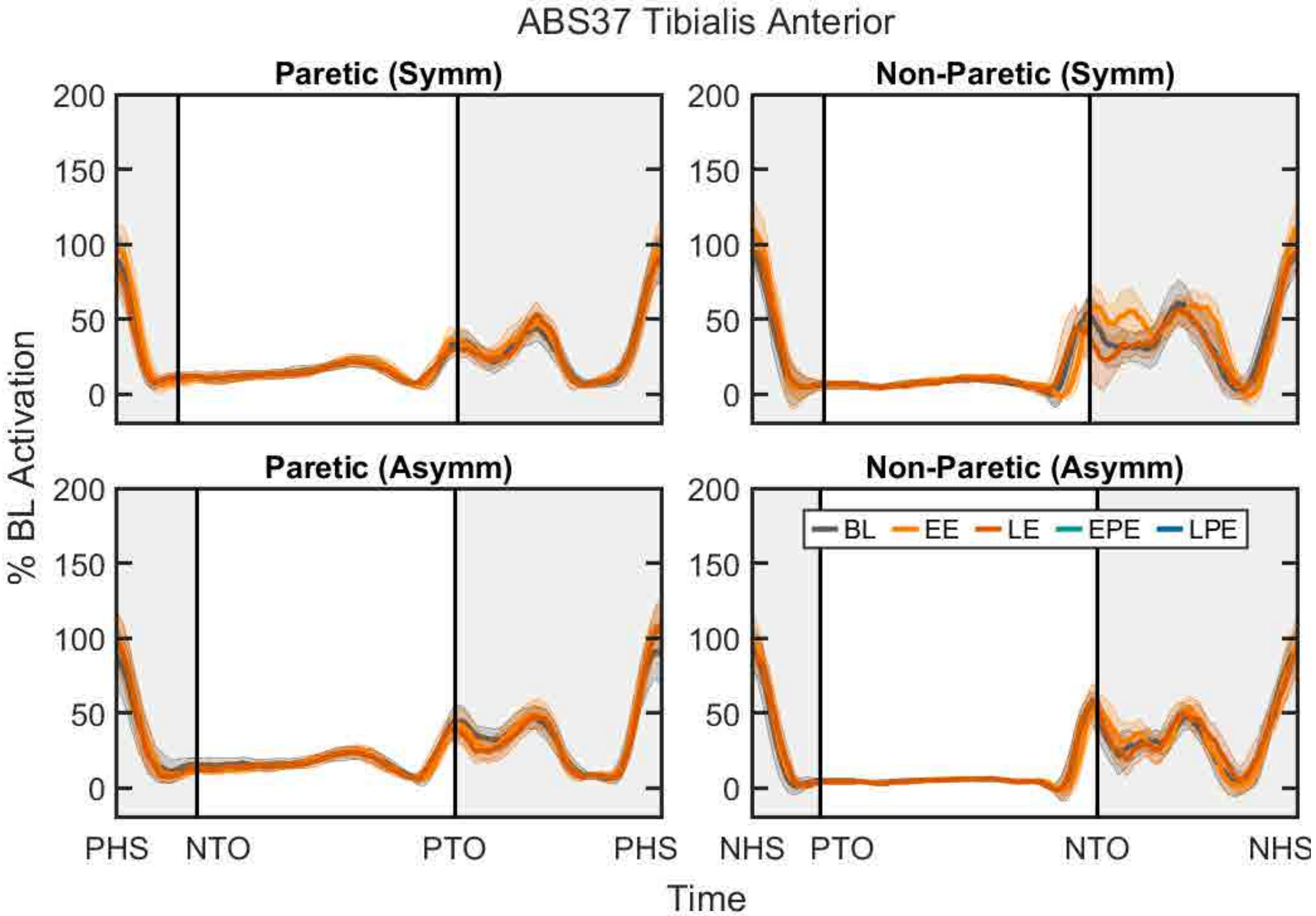
ABS37 Tibialis Anterior
Paretic (Symm)
Non-Paretic (Symm)
Paretic (Asymm)
Non-Paretic (Asymm)
% BL Activation
200
150
100
50
0
BL
EE
LE
EPE
LPE
PHS
NTO
PTO
PHS
NHS
PTO
NTO
NHS
Time

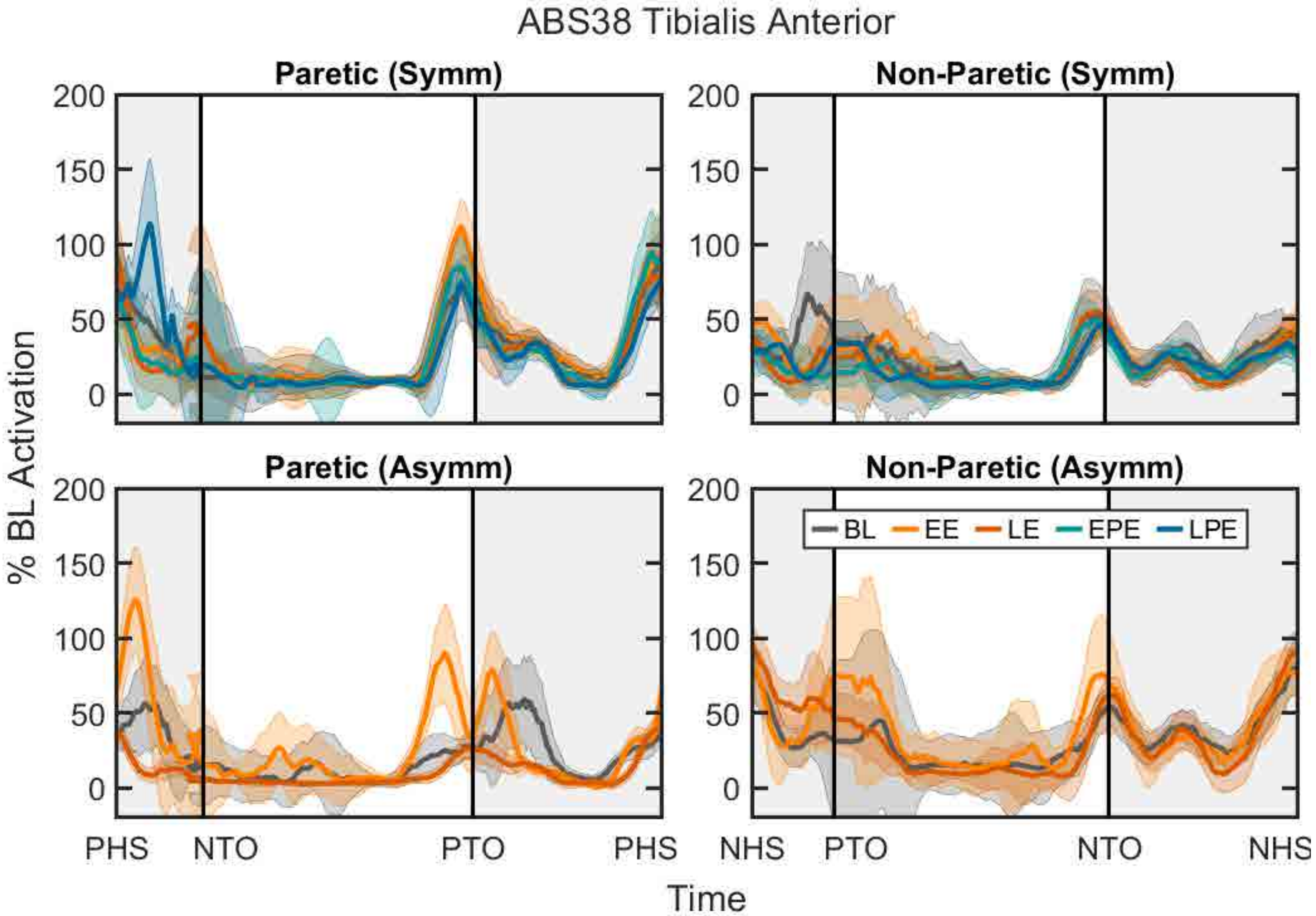
ABS38 Tibialis Anterior
Paretic (Symm)
Non-Paretic (Symm)
Paretic (Asymm)
Non-Paretic (Asymm)
% BL Activation
200
150
100
50
0
BL
EE
LE
EPE
LPE
PHS
NTO
PTO
PHS
NHS
PTO
NTO
NHS
Time

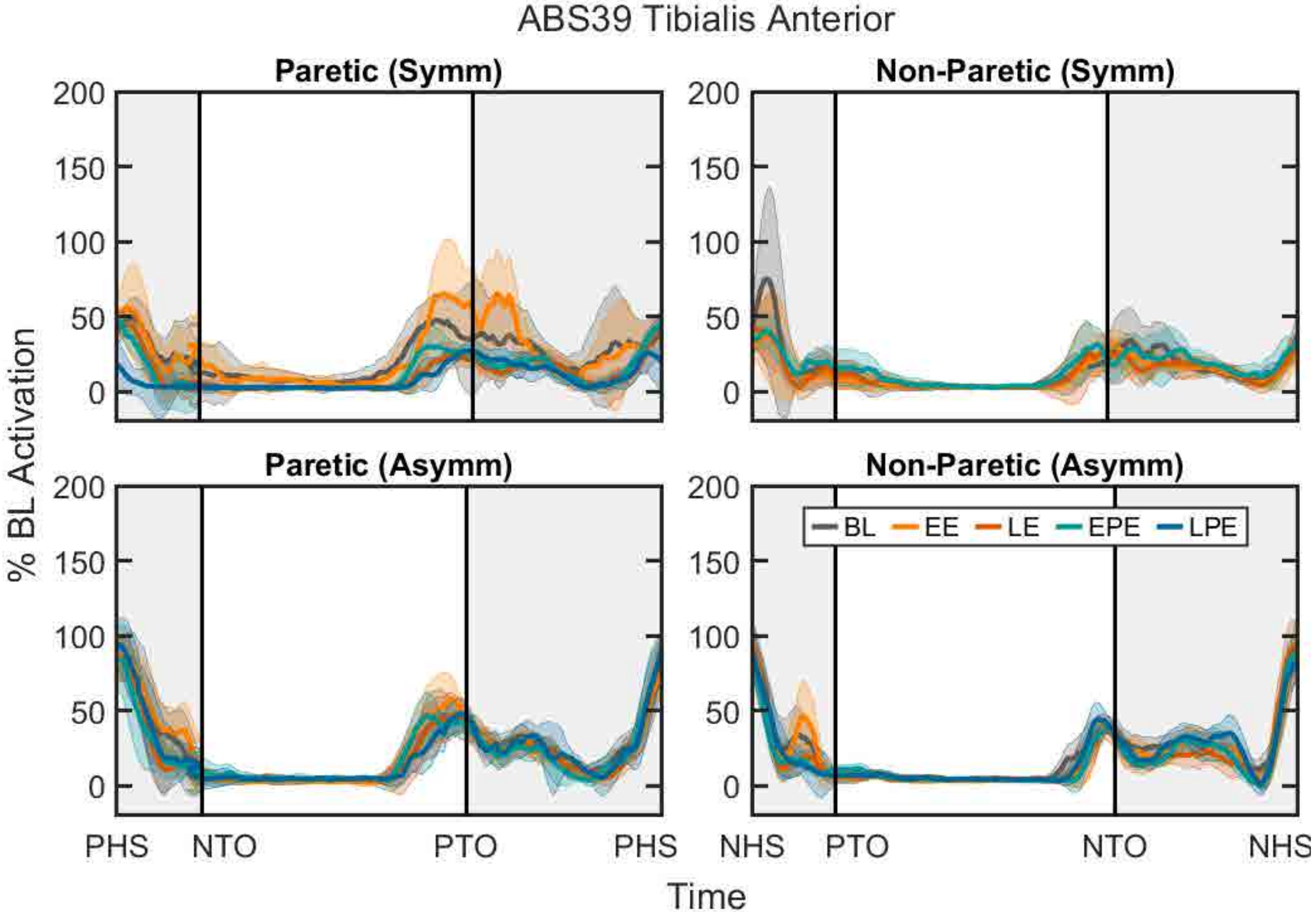

ABS39 Tibialis Anterior
Paretic (Symm)
Non-Paretic (Symm)
Paretic (Asymm)
Non-Paretic (Asymm)
% BL Activation
200
150
100
50
0
BL
EE
LE
EPE
LPE
PHS
NTO
PTO
PHS
NHS
PTO
NTO
NHS
Time

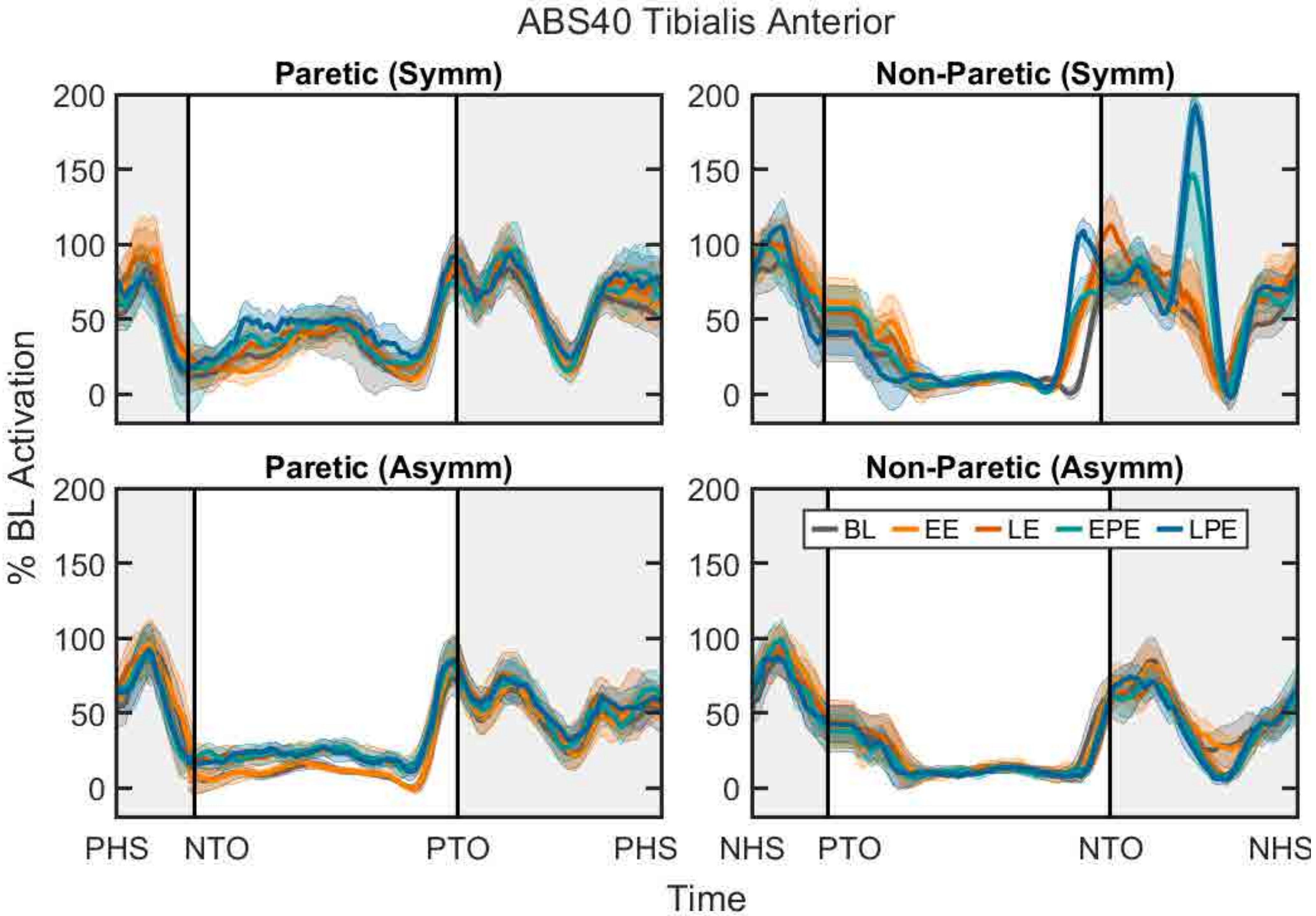
ABS40 Tibialis Anterior
Paretic (Symm)
Non-Paretic (Symm)
Paretic (Asymm)
Non-Paretic (Asymm)
% BL Activation
200
150
100
50
0
BL
EE
LE
EPE
LPE
PHS
NTO
PTO
PHS
NHS
PTO
NTO
NHS
Time

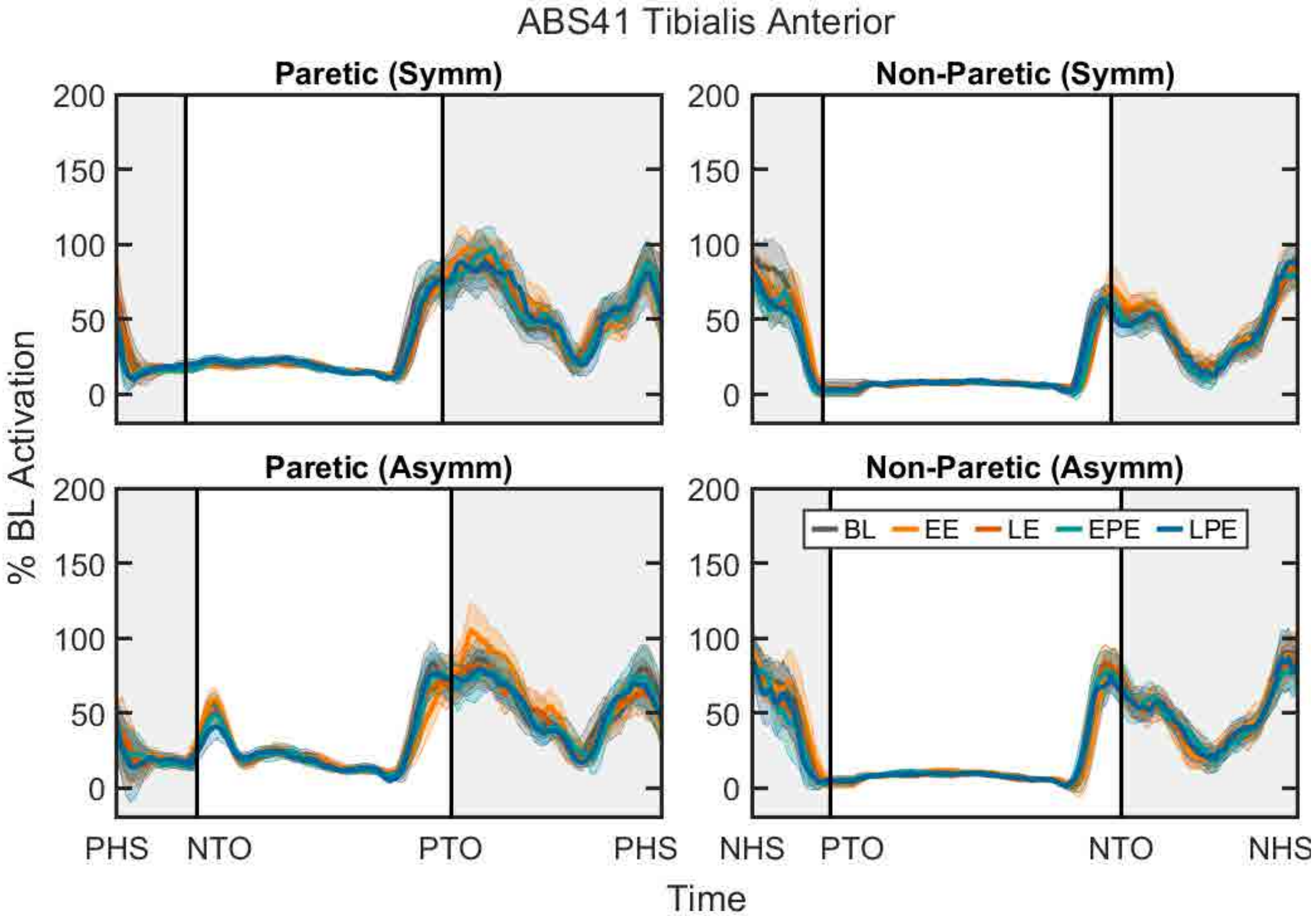
ABS41 Tibialis Anterior
Paretic (Symm)
Non-Paretic (Symm)
Paretic (Asymm)
Non-Paretic (Asymm)
% BL Activation
200
150
100
50
0
BL
EE
LE
EPE
LPE
PHS
NTO
PTO
PHS
NHS
PTO
NTO
NHS
Time

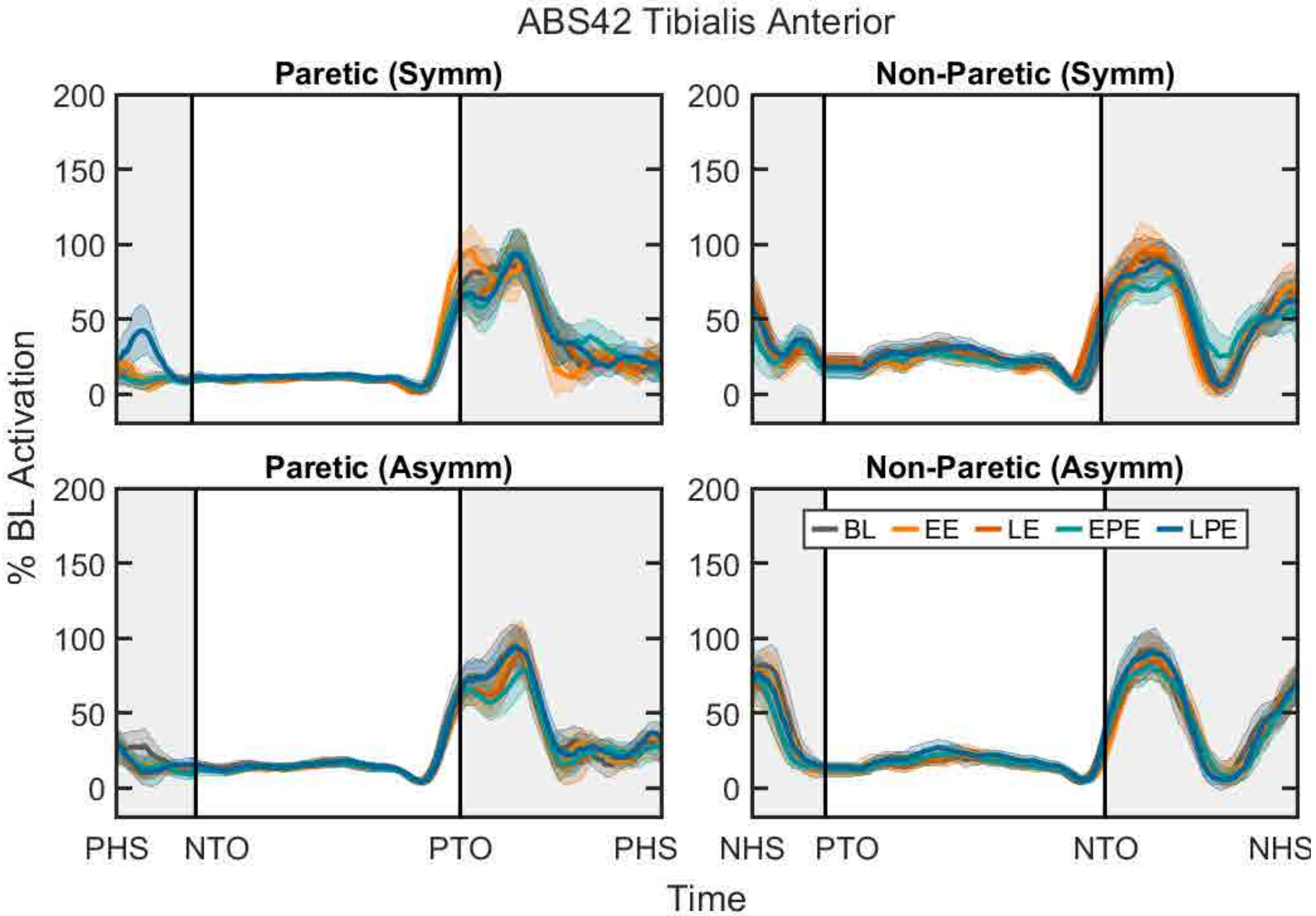
ABS42 Tibialis Anterior
Paretic (Symm)
Non-Paretic (Symm)
Paretic (Asymm)
Non-Paretic (Asymm)
% BL Activation
200
150
100
50
0
BL
EE
LE
EPE
LPE
PHS
NTO
PTO
PHS
NHS
PTO
NTO
NHS
Time

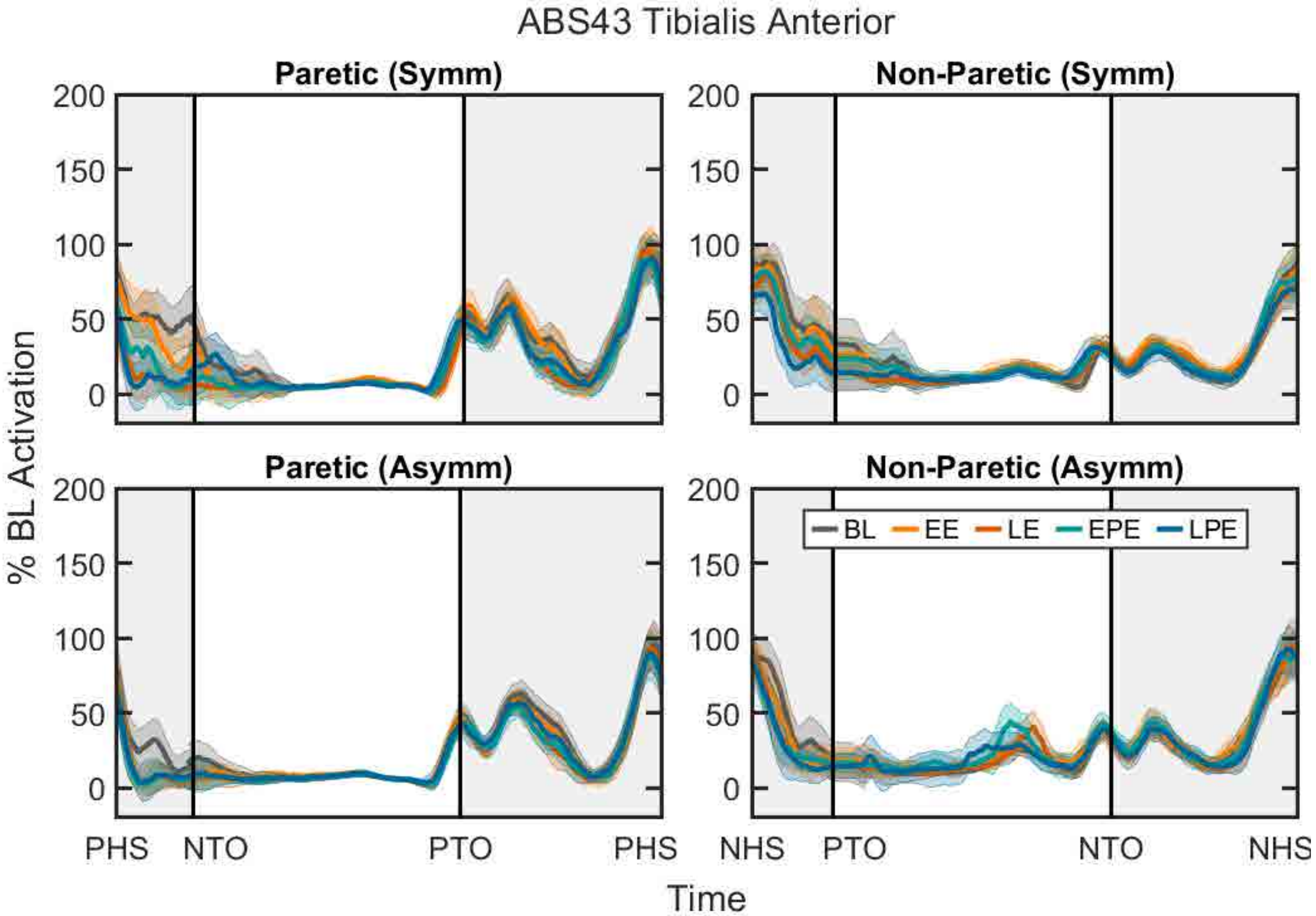
ABS43 Tibialis Anterior
Paretic (Symm)
Non-Paretic (Symm)
Paretic (Asymm)
Non-Paretic (Asymm)
% BL Activation
200
150
100
50
0
BL
EE
LE
EPE
LPE
PHS
NTO
PTO
PHS
NHS
PTO
NTO
NHS
Time

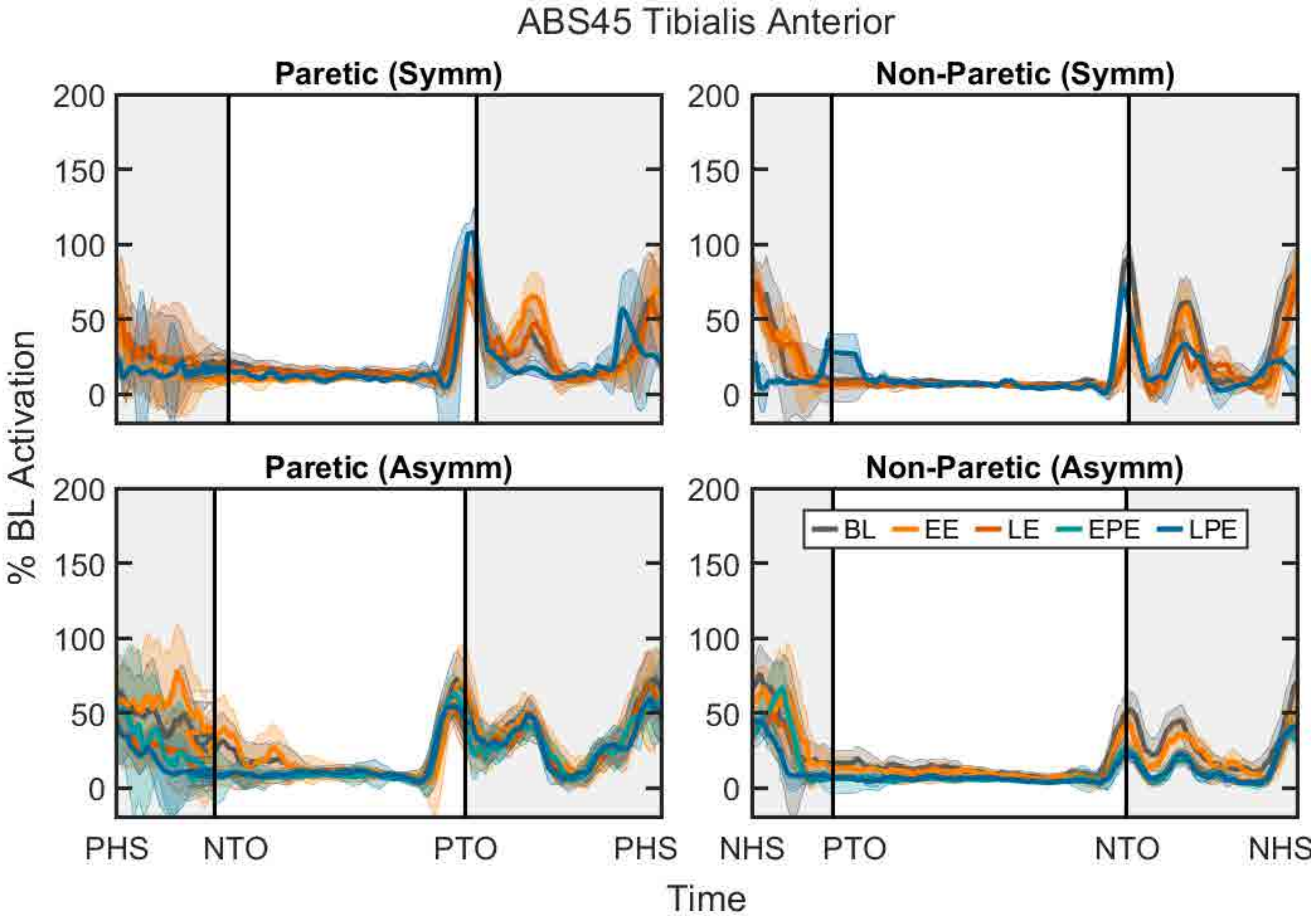
ABS45 Tibialis Anterior
Paretic (Symm)
Non-Paretic (Symm)
Paretic (Asymm)
Non-Paretic (Asymm)
% BL Activation
200
150
100
50
0
BL
EE
LE
EPE
LPE
PHS
NTO
PTO
PHS
NHS
PTO
NTO
NHS
Time
