# Supplementary material for: Effects of Belt Accelerations During Push-Off on Propulsion Mechanics in Individuals Post-Stroke
Source: IEEE Trans Neural Syst Rehabil Eng. Author manuscript; Available in PMC 2026 Apr 27. (PMC13120362; doi:10.1109/TNSRE.2026.3675477)
Supplement: supp1-3675477 [file NIHMS2162889-supplement-supp1-3675477.pdf]

Supplemental Material: Effects of Belt Accelerations During Push-Off on Propulsion Mechanics in Individuals Post-Stroke

Hannah N. Cohen, Tamara Wright, GilHwan Kim, Henry Wright, Darcy S. Reisman, and Fabrizio Sergi, *Member, IEEE*

I. Methods

Validation of Belt Acceleration Controller

To validate the developed controller, six healthy young adults participated in a walking experiment. The participants performed 5 separate walking trials where they walked at a baseline of 0.4, 0.5, 0.6, 0.8, and 1 m/s and experienced 20 consecutive perturbations (magnitude: 5 m/s^2). To measure real-time velocity, motion capture was used to track movement of the belts via three groups of five circular pieces of reflective tape placed on each treadmill belt. Change in belt velocities $v_{L,R}$ relative to baseline v_{BL} were estimated from the recorded motion-capture data. To quantify the "dose" of exposure, the parameter d_R was calculated using the following equation

$$d_R = \int_{t_{HS,L}}^{t_{TO,R}} (v_R - v_{BL}) dt \qquad (S1)$$

A mixed-model ANOVA was fit to the data with velocity as a fixed effect and participant as a random effect to test the hypothesis that d_R would not be significantly different across velocity conditions. There was no significant effect of velocity on displacement (d_R, p = 0.072; d_L, p = 0.853).

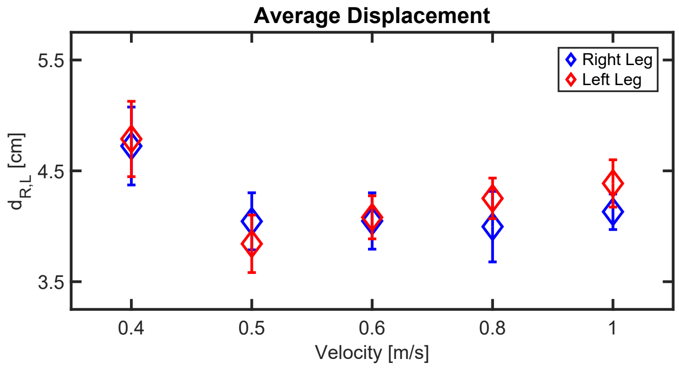

Fig. S1. Average displacement ($d_{R,L}$) $\pm$ one standard error.

II. Results

Effects of Baseline Impairment in the Non-Paretic Leg

In the non-paretic leg, there was a significant interaction between condition and FMLE score on ΔSL during LE (p = 0.004). In the symmetric condition, this relationship was significant with a slope of 0.38 $\pm$ 0.14 cm per unit of baseline FMLE score (p = 0.007), but it was not in the asymmetric condition (slope = -0.17 ± 0.13, p = 0.204). The difference (0.55 $\pm$ 0.18 cm per unit of baseline FMLE score) between the slopes of the two conditions was statistically significant (p = 0.004). The positive change induced in non-paretic SL in less impaired individuals (higher FMLE score) is greater in the symmetric condition than in the asymmetric condition, while the differential effects are smaller in more impaired participants (lower FMLE score)

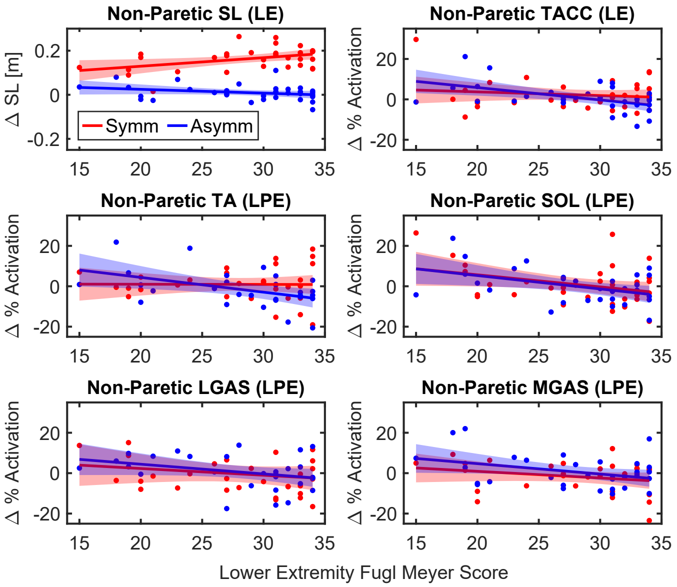

Fig. S2. Relationships between FMLE score and change in outcome measurement from BL to either LE or LPE for both conditions. Outcomes/time points shown had a significant effect of either FMLE or the interaction between condition and FMLE in the non-paretic leg.

The interaction between condition and FMLE score was also significant in the non-paretic TA during LPE (p = 0.031). The change in % TA activation per unit of baseline FMLE score was 0.01 $\pm$ 0.27 (p = 0.970) in the symmetric condition and -0.74 ± 0.29 (p = 0.012) in the asymmetric condition, with a significant difference of 0.75 $\pm$ 0.33 between slopes (p = 0.031). These relationships reflect differential effects of condition on muscle activation depending on baseline

TABLE S1
MIXED MODEL RESULTS: FIXED EFFECTS FOR ALL PARETIC LEG OUTCOMES

**Velocity**	**N par**	**DF Den**	**F Ratio**	**Prob>F**
Condition	1	33.0	0.633	0.432
Timing	2	64.2	4.435	**0.016**
Condition·Timing	2	65.1	0.443	0.643
**Peak AGRF**	**N par**	**DF Den**	**F Ratio**	**Prob>F**
Condition	1	32.9	0.580	0.452
Timing	4	127.6	10.703	**<0.001**
Condition·Timing	4	129.4	0.725	0.577
**Propulsive Impulse**	**N par**	**DF Den**	**F Ratio**	**Prob>F**
Condition	1	33.2	0.237	0.631
Timing	4	128.3	7.604	**<0.001**
Condition·Timing	4	130.0	1.810	0.131
**Soleus**	**N par**	**DF Den**	**F Ratio**	**Prob>F**
Condition	1	31.3	0.617	0.438
Timing	4	129.3	2.892	**0.025**
Condition·Timing	4	125.8	0.094	0.984
**Lateral Gastrocnemius**	**N par**	**DF Den**	**F Ratio**	**Prob>F**
Condition	1	31.3	2.219	0.146
Timing	4	126.2	4.464	**0.002**
Condition·Timing	4	127.1	0.185	0.946
**Medial Gastrocnemius**	**N par**	**DF Den**	**F Ratio**	**Prob>F**
Condition	1	26.4	0.467	0.501
Timing	4	112.9	1.753	0.143
Condition·Timing	4	107.9	0.381	0.822
**Tibialis Anterior**	**N par**	**DF Den**	**F Ratio**	**Prob>F**
Condition	1	32.3	0.228	0.637
Timing	4	127.8	5.245	**<0.001**
Condition·Timing	4	129.0	0.434	0.784
**Tibialis Anterior Co-Contraction**	**N par**	**DF Den**	**F Ratio**	**Prob>F**
Condition	1	32.6	0.445	0.510
Timing	4	131.1	0.975	0.424
Condition·Timing	4	130.2	0.162	0.957
**Trailing Limb Angle**	**N par**	**DF Den**	**F Ratio**	**Prob>F**
Condition	1	26.9	0.138	0.713
Timing	4	105.7	23.099	**<0.001**
Condition·Timing	4	106.6	1.824	0.130
**Stride Length**	**N par**	**DF Den**	**F Ratio**	**Prob>F**
Condition	1	31.4	0.649	0.426
Timing	4	127.3	99.809	**<0.001**
Condition·Timing	4	126.6	0.690	0.600
**Stride Duration**	**N par**	**DF Den**	**F Ratio**	**Prob>F**
Condition	1	33.1	0.844	0.365
Timing	4	130.4	4.198	**0.003**
Condition·Timing	4	130.3	3.058	**0.019**
**Stance Duration**	**N par**	**DF Den**	**F Ratio**	**Prob>F**
Condition	1	33.2	0.842	0.365
Timing	4	130.5	0.881	0.477
Condition·Timing	4	130.4	1.840	0.125

TABLE S2
MIXED MODEL RESULTS: FIXED EFFECTS FOR ALL NON-PARETIC OUTCOMES

**Velocity**	**N par**	**DF Den**	**F Ratio**	**Prob>F**
Condition	1	33.0	0.633	0.432
Timing	2	64.2	4.435	**0.016**
Condition·Timing	2	65.1	0.443	0.643
**Peak AGRF**	**N par**	**DF Den**	**F Ratio**	**Prob>F**
Condition	1	33.1	3.693	0.063
Timing	4	130.0	4.748	**0.001**
Condition·Timing	4	130.7	4.937	**0.001**
**Propulsive Impulse**	**N par**	**DF Den**	**F Ratio**	**Prob>F**
Condition	1	33.2	4.564	**0.040**
Timing	4	130.6	0.738	0.568
Condition·Timing	4	130.6	7.239	**<0.001**
**Soleus**	**N par**	**DF Den**	**F Ratio**	**Prob>F**
Condition	1	32.9	0.182	0.672
Timing	4	130.6	2.703	**0.033**
Condition·Timing	4	129.3	0.250	0.909
**Lateral Gastrocnemius**	**N par**	**DF Den**	**F Ratio**	**Prob>F**
Condition	1	31.7	0.446	0.509
Timing	4	125.3	5.158	**<0.001**
Condition·Timing	4	126.1	0.044	0.996
**Medial Gastrocnemius**	**N par**	**DF Den**	**F Ratio**	**Prob>F**
Condition	1	33.1	4.061	0.052
Timing	4	130.5	3.622	**0.008**
Condition·Timing	4	129.8	1.220	0.305
**Tibialis Anterior**	**N par**	**DF Den**	**F Ratio**	**Prob>F**
Condition	1	31.8	0.008	0.930
Timing	4	121.8	1.655	0.165
Condition·Timing	4	122.2	0.599	0.664
**Tibialis Anterior Co-Contraction**	**N par**	**DF Den**	**F Ratio**	**Prob>F**
Condition	1	31.7	0.041	0.841
Timing	4	108.5	1.817	0.131
Condition·Timing	4	109.4	1.170	0.328
**Trailing Limb Angle**	**N par**	**DF Den**	**F Ratio**	**Prob>F**
Condition	1	24.7	2.928	0.100
Timing	4	72.6	9.427	**<0.001**
Condition·Timing	4	73.4	4.690	**0.002**
**Stride Length**	**N par**	**DF Den**	**F Ratio**	**Prob>F**
Condition	1	30.2	8.196	**0.008**
Timing	4	123.6	33.965	**<0.001**
Condition·Timing	4	124.4	27.180	**<0.001**
**Stride Duration**	**N par**	**DF Den**	**F Ratio**	**Prob>F**
Condition	1	33.1	0.710	0.406
Timing	4	130.3	3.793	**0.006**
Condition·Timing	4	130.3	2.936	**0.023**
**Stance Duration**	**N par**	**DF Den**	**F Ratio**	**Prob>F**
Condition	1	33.2	0.028	0.867
Timing	4	130.6	0.505	0.732
Condition·Timing	4	130.5	1.449	0.221

impairment. While less impaired individuals increased non-paretic TA activation more in the symmetric condition, more impaired individuals increased non-paretic TA activation more in the asymmetric condition.

FMLE score had a significant effect on the change in % activation of non-paretic TACC during exposure, and all three non-paretic plantarflexors post-exposure, independent of condition. The average slopes were -0.39 ± 0.16 for TACC (p = 0.017), -0.62 ± 0.21 for the SOL (p = 0.006), -0.41 ± 0.20 for the LGAS (p = 0.046), and -0.42 ± 0.19 for the MGAS (p = 0.032). Similar to the paretic leg, more impaired participants had greater increases in muscle activation than less impaired participants across both conditions.
